# Structure–activity relationship studies of cyclopropenimines as enantioselective Brønsted base catalysts†
†Electronic supplementary information (ESI) available: Experimental procedures and product characterization data. CCDC 880701. For ESI and crystallographic data in CIF or other electronic format see DOI: 10.1039/c4sc02402h



**DOI:** 10.1039/c4sc02402h

**Published:** 2014-12-19

**Authors:** Jeffrey S. Bandar, Alexandre Barthelme, Alon Y. Mazori, Tristan H. Lambert

**Affiliations:** a Department of Chemistry , Columbia University , 3000 Broadway , New York , NY 10027 , USA . Email: tl2240@columbia.edu

## Abstract

We recently demonstrated that chiral cyclopropenimines are viable Brønsted base catalysts in enantioselective Michael and Mannich reactions. Herein, we describe a series of structure–activity relationship studies that provide an enhanced understanding of the effectiveness of certain cyclopropenimines as enantioselective Brønsted base catalysts. These studies underscore the crucial importance of dicyclohexylamino substituents in mediating both reaction rate and enantioselectivity. In addition, an unusual catalyst CH···O interaction, which provides both ground state and transition state organization, is discussed. Cyclopropenimine stability studies have led to the identification of new catalysts with greatly improved stability. Finally, additional demonstrations of substrate scope and current limitations are provided herein.

## Abstract 2

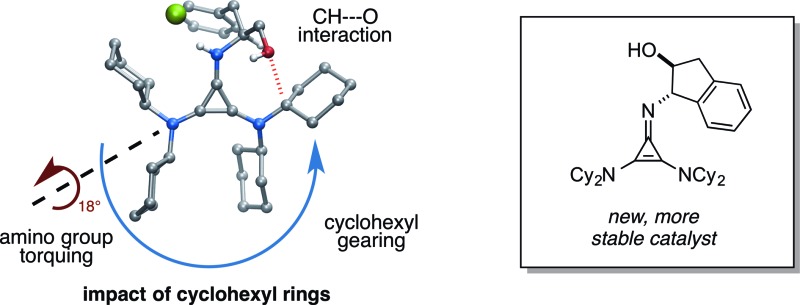
New insights aid in the understanding and design of cyclopropenimine-based asymmetric catalysts.

## Introduction

Brønsted base-mediated deprotonation represents a fundamental mode of HOMO-raising activation, and the anions thus generated readily participate in a range of valuable transformations.^1^ Although chiral Brønsted bases offer the promise of rendering such transformations enantioselective, the field of enantioselective Brønsted base catalysis has not advanced as rapidly as other areas of organocatalysis. This situation is beginning to change, primarily due to the development in recent years of a number of catalysts possessing more potent basicities and useful reactivity profiles.

**Fig. 1:**
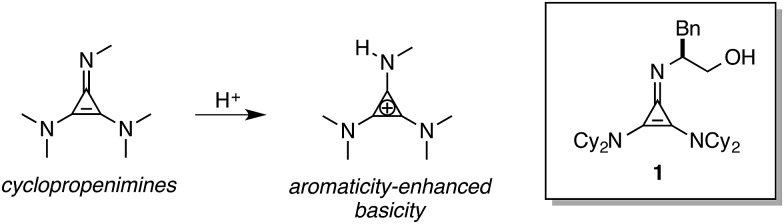
Enantioselective Brønsted base catalysis with cyclopropenimine **1**.

In 2012, we disclosed that cyclopropenimine **1** (Fig. 1) is a highly effective Brønsted base catalyst for enantioselective Michael reactions of the O'Donnell glycine imine.^2^ More recently, we demonstrated that **1** is also particularly effective for enantioselective catalytic Mannich reactions of the same pronucleophile with a variety of imine electrophiles, including those bearing aliphatic substituents.^3^ Cyclopropenimines such as **1** have been found to be strongly Brønsted basic as a result of aromatic stabilization of the conjugate acid. Undoubtedly, the notable potency of catalyst **1** in comparison to related catalysts derived from guanidine or tertiary amine functionality is largely attributed to its stronger basicity. However, in the course of our investigations, it became clear that the remarkable reactivity of cyclopropenimine **1** could not be attributed solely to an increase in catalyst basicity.

Given our earlier observation of some peculiar structure–activity relationships and a desire to further exploit the effectiveness of the cyclopropenimine scaffold for enantioselective Brønsted base catalysis, we have undertaken an in-depth examination of cyclopropenimine **1** and related structures. These studies have revealed a number of important structural elements that are critical to the high efficiency of catalyst **1**, especially regarding the dicyclohexylamino substituents. In addition, the existence of an intramolecular CH···O interaction in the ground state organization of **1** has been identified.^4^ These studies have also led to the discovery of catalysts with significantly improved stability profiles, which should further expand the utility of cyclopropenimines as chiral Brønsted base catalysts.

### Background

For obvious reasons, the position of the acid–base equilibrium between substrate and catalyst provides an effective limit to the scope of any catalytic base. Therefore, much recent research in the area of asymmetric Brønsted base catalysis has focused on the development of catalysts with the capacity to activate a broader range of substrates, either *via* increased basicities or by co-activation through hydrogen-bonding.

As a prime example of this latter approach, bifunctional catalysts pairing tertiary amines with strong hydrogen-bond donors have been vigorously studied.^5^ These bifunctional catalysts induce high selectivity in reactions of many relatively acidic substrate classes, although they show limited success in the activation of less acidic substrates (p*K*_a_ > 17 in DMSO). Accordingly, a number of researchers have explored the installation of more basic functionality with the goal of increasing catalytic activity and thus expanding the range of viable substrate classes.

**Fig. 2:**
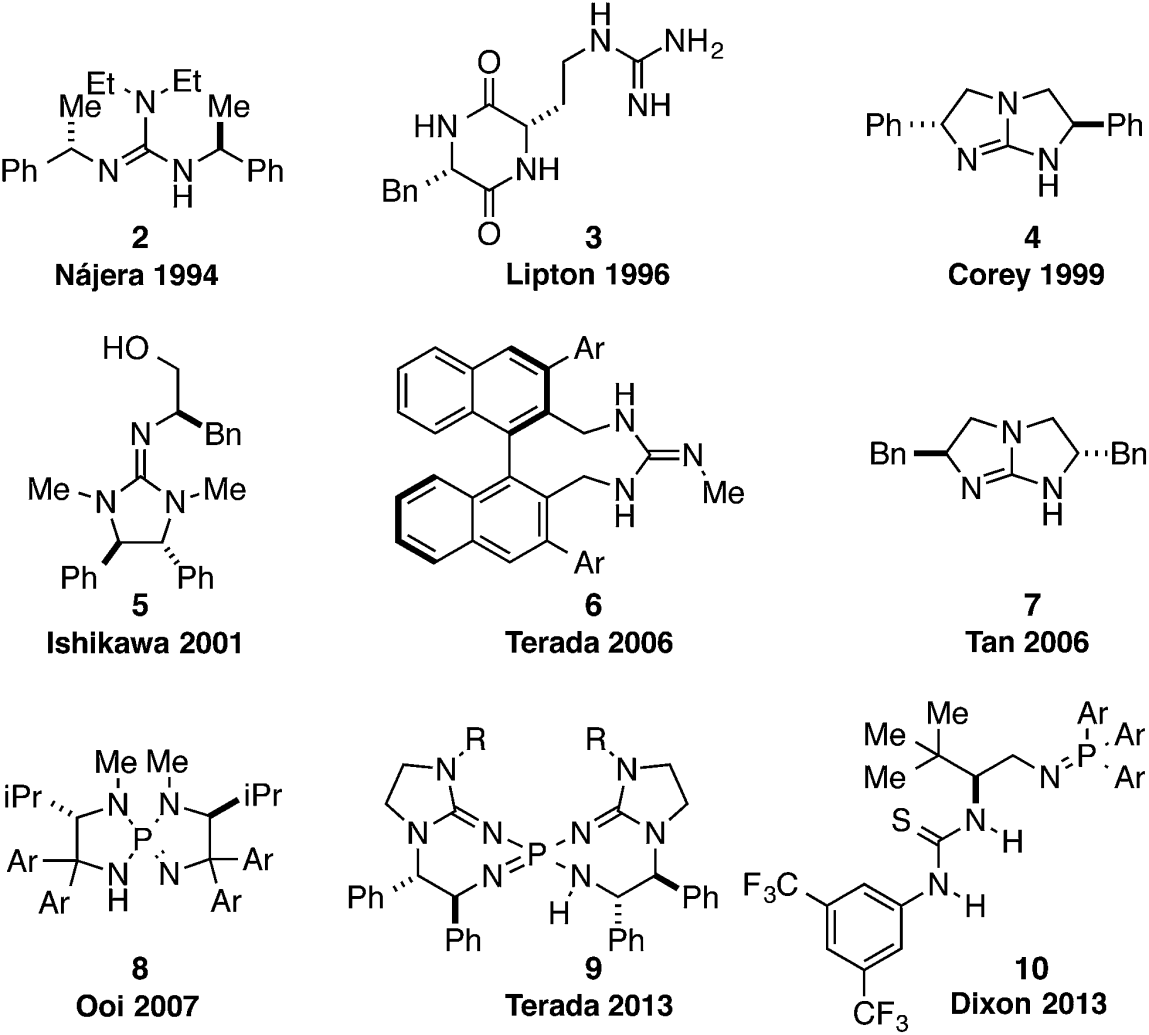
Examples of chiral Brønsted base catalysts with strong basicities.

For example, chiral guanidine **2** was first developed by Nájera in 1994 as an asymmetric catalyst for the addition of nitroalkanes to aldehydes,^6^ albeit with limited selectivities (Fig. 2). Since then, many effective scaffolds have been identified in the area of chiral guanidine catalysis. These include Lipton's dipeptide guanidine **3** (ref. 7) and Corey's *C*_2_-symmetric bicyclic guanidine **4**,^8^ both developed for the asymmetric Strecker reaction. Other important chiral guanidine catalysts include Ishikawa's bifunctional guanidine **5**,^9^ Terada's binaphthyl-based guanidine **6**,^10^ and Tan's modified Corey-type bicyclic guanidine **7**.^11^

The recent development of several catalyst scaffolds with significantly stronger basicities has further enhanced the impact of enantioselective Brønsted base catalysis. In particular, the *P*-spiro chiral bicyclic iminophosphoranes **8**, pioneered by Ooi and Uraguchi have been utilized as catalysts for a range of substrates, including azlactones, phosphonoacetates, dialkylphosphites and nitroalkanes (Fig. 2).^12^ Terada recently reported a highly basic chiral bis(guanidino)iminophosphorane **9** that effectively catalyzes the asymmetric amination of tetralone-like ketones.^13^ Finally, Dixon has developed a class of chiral bifunctional thiourea iminophosphoranes **10** and employed them as catalysts for the asymmetric nitro-Mannich reaction with ketimines.^14^

The chiral cyclopropenimine **1** developed in our lab has also proven to be a highly effective enantioselective Brønsted base catalyst. For example, we reported that **1** catalyzes the highly enantioselective Michael addition of O'Donnell glycine imine **11a** to methyl acrylate in only 5 min under neat conditions, and within 1 hour under reasonable concentration in ethyl acetate (eqn (1)).^2^ We further showed that **1** catalyzes enantioselective Mannich reactions rapidly (eqn (2)) and with a substrate scope surpassing that of established platforms (*i.e.* aliphatic imines).^3^ In both of these cases, the reactivity of **1** was demonstrated to far exceed that of less basic catalysts, a further indication that increased catalyst basicity can lead to significantly improved reaction outcomes.
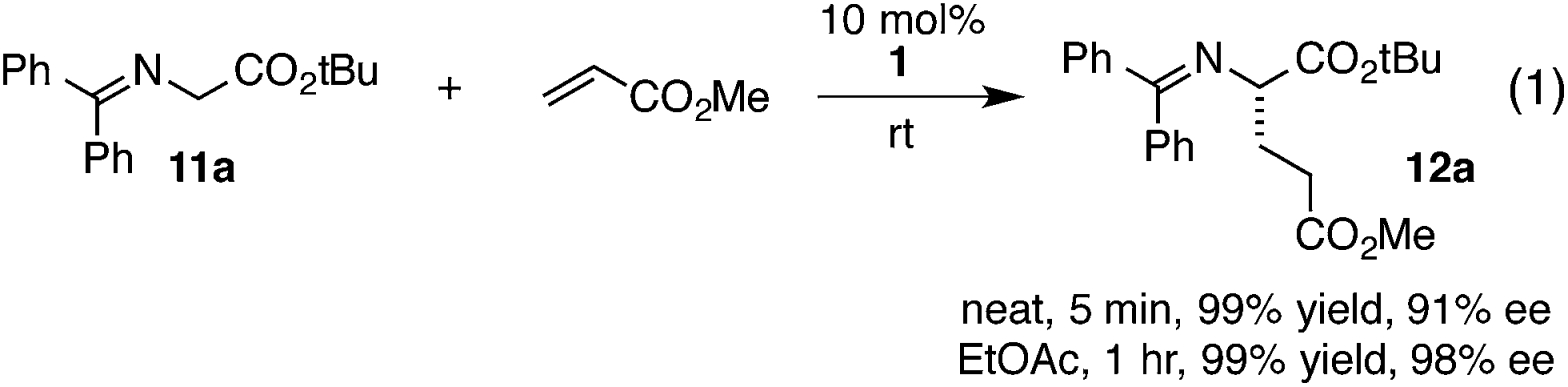
 
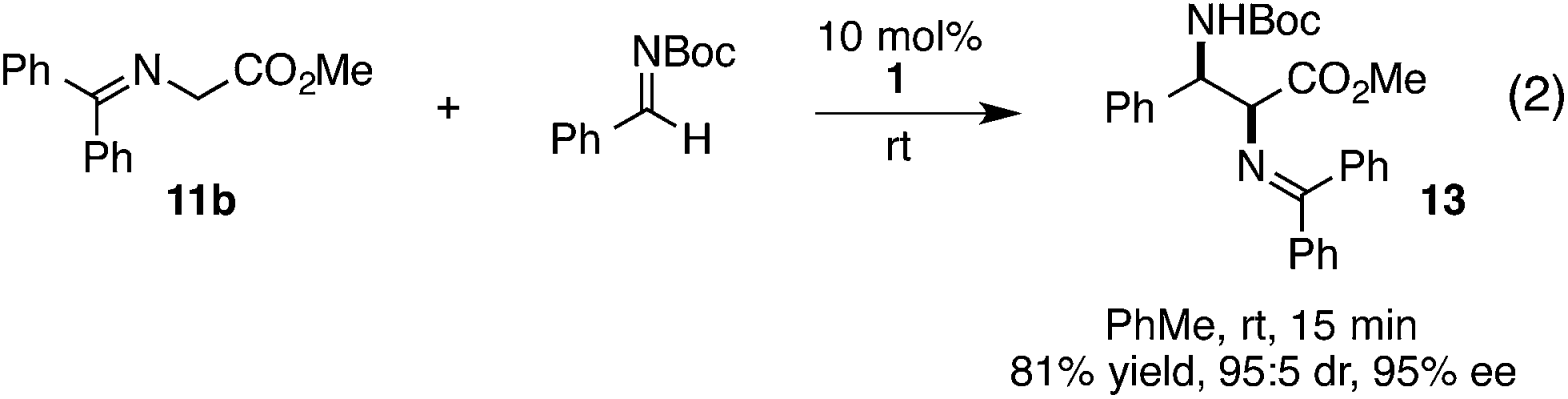


With the goal of further expanding the range of applications of asymmetric cyclopropenimine catalysis, we recently undertook a study of the factors that govern this type of catalysis, with the particular goal of understanding the structural aspects of catalyst **1** that lead to the observed high levels of reactivity and enantioselectivity. In this Article, we disclose our findings from this study along with a full account of the development of chiral cyclopropenimines as a unique platform for enantioselective Brønsted base catalysis.

## Results and discussion

### Catalyst synthesis

To prepare cyclopropenimine catalysts, we relied on the method first reported by Yoshida,^15^ in which amines are added to either tetrachlorocyclopropene or, more conveniently, pentachlorocyclopropane (**14**, eqn (3)).^16^ For example, addition of six equivalents of *N*,*N*-dicyclohexylamine to **14** results in the rapid and quantitative production of **15**, which is an isolable, stable solid that can be stored indefinitely. Large-scale batches (up to 60 g) of **15** have been prepared using this method.

The head group or “imino” nitrogen can be subsequently installed, either in a separate step or *in situ*, by the simple treatment of **15** with an equivalent of the desired chiral primary amine (*e.g.* phenylalaninol). The resulting trisamino-cyclopropenium chlorides are typically crystalline materials that are stable and can be stored without decomposition at room temperature. Using this approach, we have prepared up to 47 g of the salt **1**·HCl in a single run.
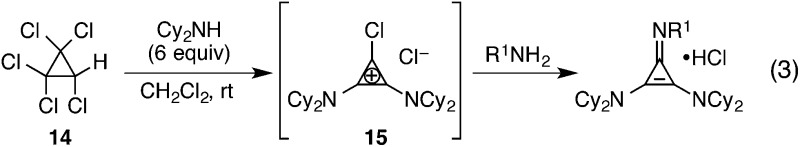


To generate the cyclopropenimine free base, a CH_2_Cl_2_ solution of the salt can be simply washed with 1 M NaOH, and the organic layer then dried and concentrated. The resulting solid is of sufficient purity to be directly used as a catalyst without further purification. For cyclopropenimines with sterically less-demanding amino groups in the 2,3-positions, the higher basicity may necessitate deprotonation with a stronger base, such as NaH or KO*t*Bu.

### The role of catalyst H-bonding

It is well understood that, generally, in order to impart high reactivity and enantioselectivity, a chiral Brønsted base catalyst must incorporate both a basic functionality and an H-bonding moiety. It can be assumed that the H-bonding groups may serve to: (1) activate the pronucleophile by lowering the energy barrier to deprotonation, (2) activate the electrophile *via* LUMO-lowering general acid catalysis, and/or (3) provide two-point organization (along with the conjugate acid of the base functionality) in the enantiodetermining transition state.

**Fig. 3:**
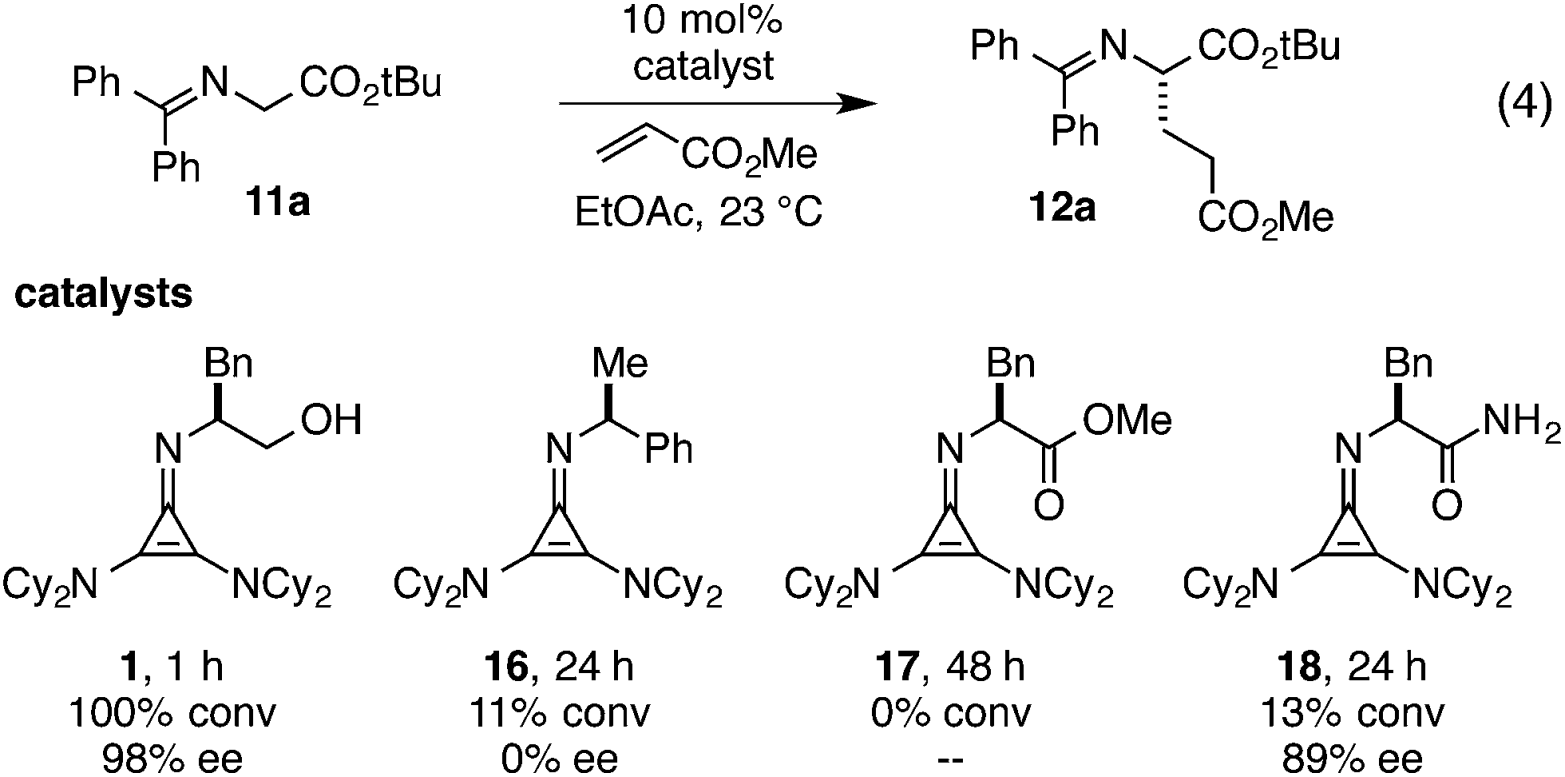
Necessity of H-bonding functionality for high reactivity and enantioselectivity.

Consistent with earlier reports, we also observed the requirement for H-bonding functionality in our cyclopropenimine catalyst systems. As an illustration, while catalyst **1** effected the Michael reaction shown in eqn (4) in 1 h with complete conversion and 98% ee, imines **16** and **17**, lacking H-bonding functionality, resulted in no enantioselectivity and little or no conversion over 24–48 h (Fig. 3). On the other hand, imine **18**, bearing alternative H-bonding functionality, generated product with a significant level of enantioselection, albeit with only poor conversion. Clearly, the hydroxyl group in catalyst **1** plays an essential role in lowering the overall reaction energy barrier and in biasing the competing diastereomeric transition states.

To gain further insight into the role of H-bonding, we examined the reaction conditions employed for the cyclopropenimine-catalyzed enantioselective Michael reaction. Specifically, we were interested in the correlation between enantioselectivity and the solvent dielectric constant.^17^ Table 1 shows ten solvents with dielectric constants ranging from 2.3 to 21. Notably, all solvents with a dielectric constant ≤6.0 resulted in Michael adduct with 98–99% ee, including, notably, triethylamine. With the use of solvents with dielectric constants higher than 6.0, enantioselectivity decreased relatively modestly. These observations suggest that transition state organization is governed by reasonably strong intermolecular forces. Only high dielectric constant solvents, including acetone and especially *n*-butanol, resulted in product with significantly decreased enantioenrichment.

**Table 1:** Solvent screen for cyclopropenimine-catalyzed enantioselective Michael reaction^*a*^

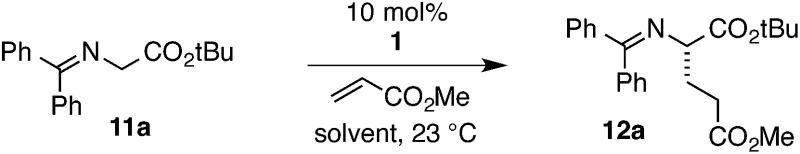					
Entry	Solvent	*ε*	Time (h)	Yield (%)	ee (%)
1	1,4-Dioxane	2.3	8	95	98
2	PhMe	2.4	5	95	99
3	NEt_3_	2.4	6	95	99
4	Et_2_O	4.3	2	95	98
5	EtOAc	6.0	2	86	98
6	THF	7.5	24	95	89
7	CH_2_Cl_2_	9.1	10	95	86
8	1,2-F_2_–C_6_H_4_	14.3	4	95	89
9	*n*-BuOH	17.5	24	45	55
10	Acetone	21	1.5	95	80

^*a*^Conversion determined by ^1^H NMR based on Bn_2_O standard. Enantiomeric excesses (ee) were determined by chiral HPLC. *ε*: dielectric constant.

Further insight into the role of H-bonding came from a screen of the electrophile (Table 2). Notably, an increase in the steric demand of the ester group in the acrylate series led to significant increase in reaction time, although with no impact on selectivity (entries 1 and 2). On the other hand, reaction of trifluoroethyl acrylate, despite its enhanced electrophilicity, was also slower and less selective (entry 3). A reasonable explanation for this observation is that the more electron-deficient ester is a less capable H-bond acceptor, which leads to a less well-organized transition state. Tellingly, acrylonitrile and phenyl vinyl sulfone (entries 4 and 5), which presumably have significantly different H-bonding geometries than the carbonyl-based electrophiles, were found to be substantially less reactive and poorly selective.

**Table 2:** Electrophile screen as a probe for H-bonding interaction^*a*^

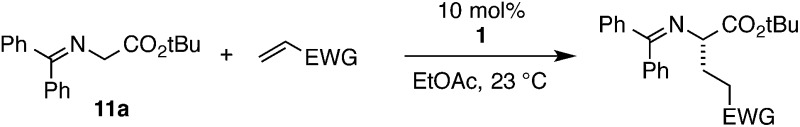					
Entry	EWG	Time (h)	Yield (%)	ee (%)	Attribution
1	CO_2_Et	2.5	96	99	
2	CO_2_ *t*Bu	12	98	99	Increased steric hindrance → slower rate
3	CO_2_CH_2_CF_3_	6	90	58	More electron-withdrawing → slower rate, lower ee
4	CN	30	97	77	Non-carbonyl → slower rate lower ee
5	SO_2_Ph	24	89	41	

^*a*^Yields based on isolated and purified product. Enantiomeric excesses (ee) were determined by chiral HPLC.

We continued to explore the role of H-bonding by examining strategically modified glycine imine substrates (eqn (5)). We found methyl and benzyl glycinate imines to be somewhat more reactive but as selective as the *t*-Bu glycinate imine (**19** and **20**). This result parallels our previous observations in cyclopropenimine-catalyzed Mannich reactions with glycinate imines. Tellingly, the reaction with a nitrile imine substrate, while faster presumably due to increased acidity, resulted in essentially no enantioselectivity (**21**). On the other hand, a substrate in which the imine nitrogen is replaced with carbon had lower reactivity, but resulted in a moderate enantiomeric excess of 70%, indicating that H-bonding to the nitrogen atom in the glycinate imines is not critical for asymmetric induction (**22**).
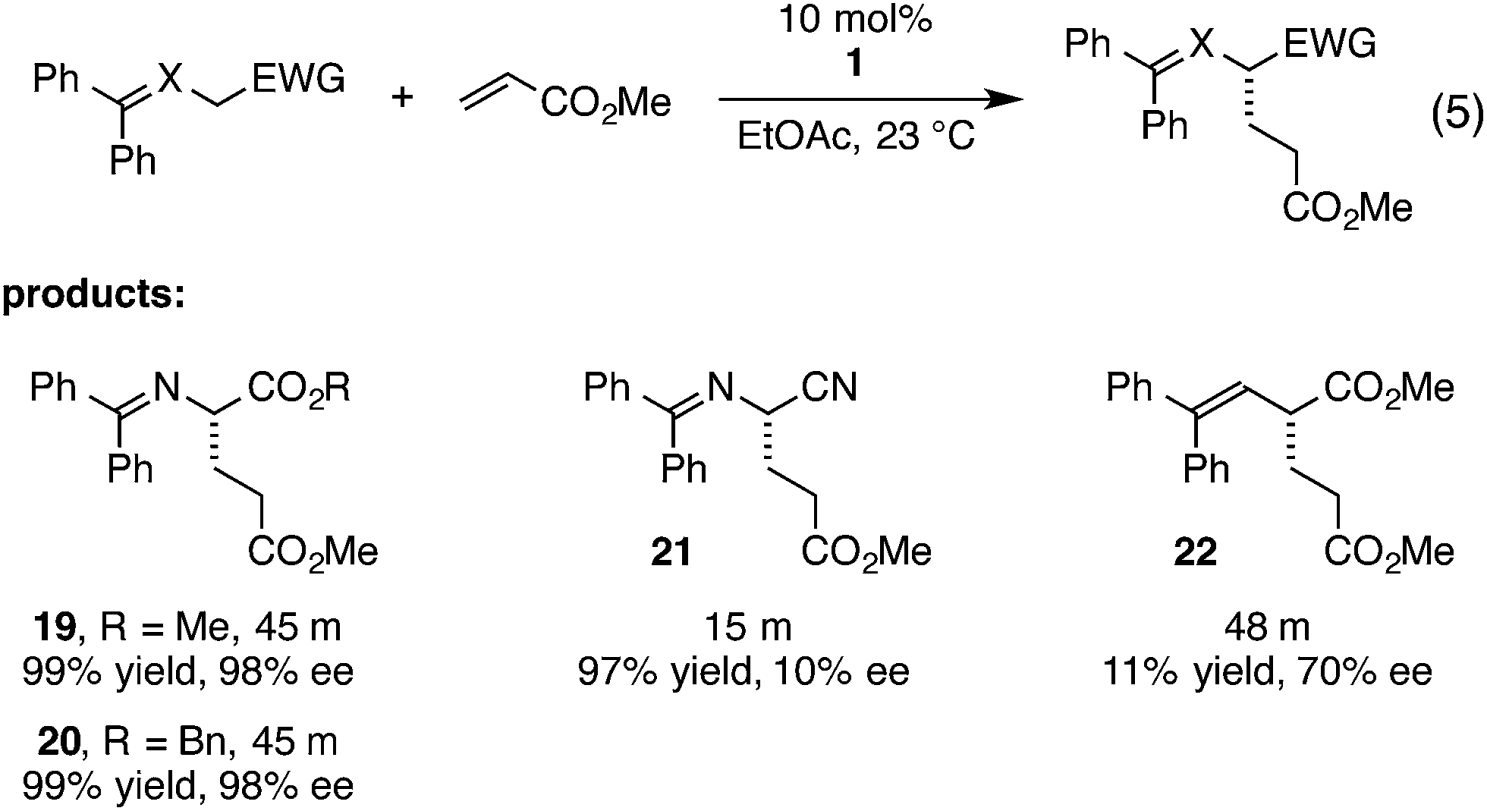


### Conformational requirements of the electrophile

As a probe of the conformational requirements of the Michael acceptor, we compared the performance of two cyclic unsaturated ketones (Table 3). Cyclopent-2-enone was found to undergo addition over the course of 48 h, albeit with a notable lack of diastereoselectivity and enantioselectivity (entry 1). On the other hand, 2-methylenecyclopentanone reacted in only 45 min to furnish the Michael adduct in essentially quantitative yield with 95% ee, albeit as a 1 : 1 mixture of diastereomers (entry 3). This lack of diastereoselection is almost surely due to a nonselective protonation (or keto–enol tautomerization) of the initial conjugate addition intermediate. We propose that the dramatic difference in enantioselection between cyclopent-2-enone and 2-methylenecyclopentenone reflects a marked preference for the electrophile to adopt an s-*cis* conformation in the enantiodetermining transition state, which is in good agreement with the modelled transition states discussed below.

**Table 3:** Comparison of s-*trans vs.* s-*cis* Michael acceptors^*a*^

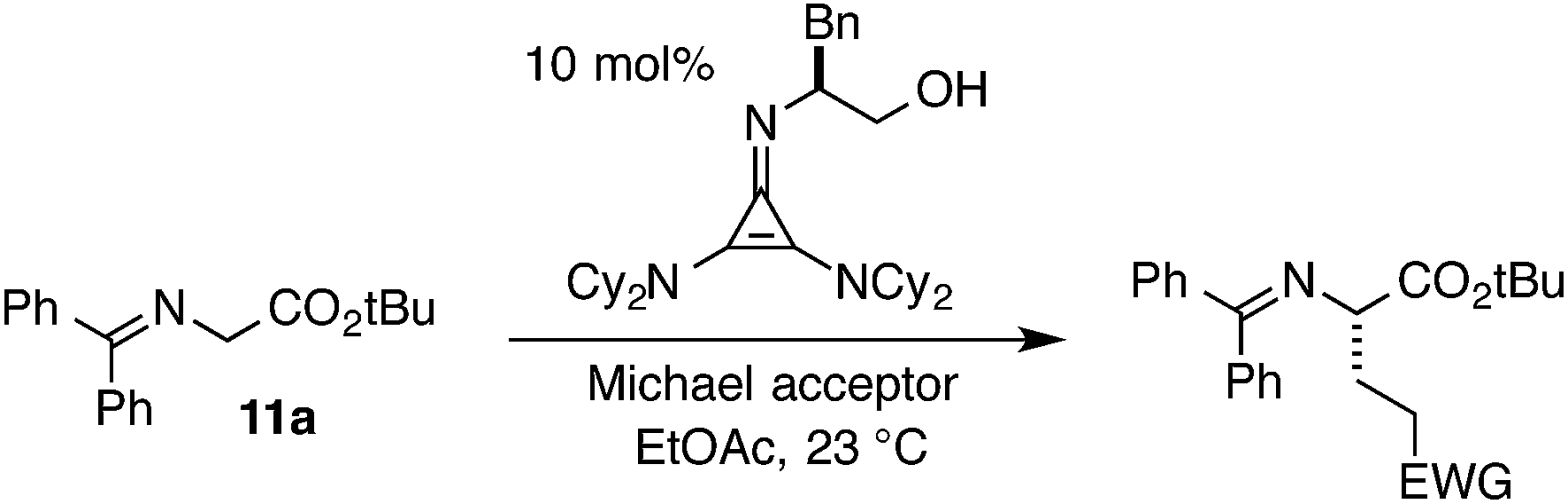						
Entry	Electrophile	Product	Time (h)	Yield (%)	dr	ee (%)
1	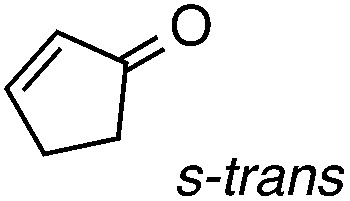	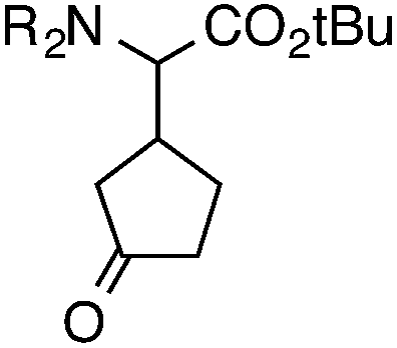	48	88	78 : 22	4/30
2	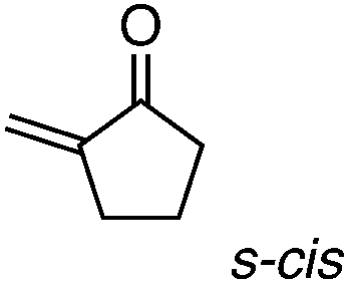	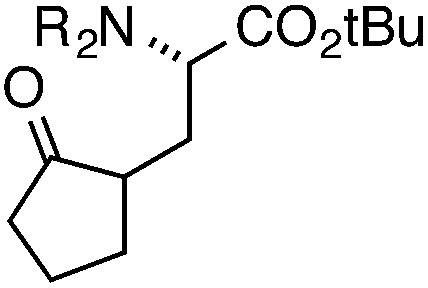	0.75	99	1 : 1	95/95

^*a*^Yields based on isolated and purified product. Enantiomeric excesses (ee) were determined by chiral HPLC.

### The role of the catalyst 2,3-amino substituents

At the outset of this program, we assumed that the 2,3-amino substituents of the cyclopropenimine framework would play a relatively minor role in the operation of these catalysts and that any modifications to these substituents that resulted in an attenuation of basicity would lead to a corresponding decrease in catalyst reactivity. In fact, we have found that the nature of these substituents has a substantial impact on the reactivity and selectivity of these cyclopropenimine catalysts. Most remarkable was the finding that *N*,*N*-dicyclohexylamino groups are critical for the optimal performance of catalyst **1**, despite the fact that these groups lead to cyclopropenimines with lower basicity than those bearing sterically less-demanding groups.
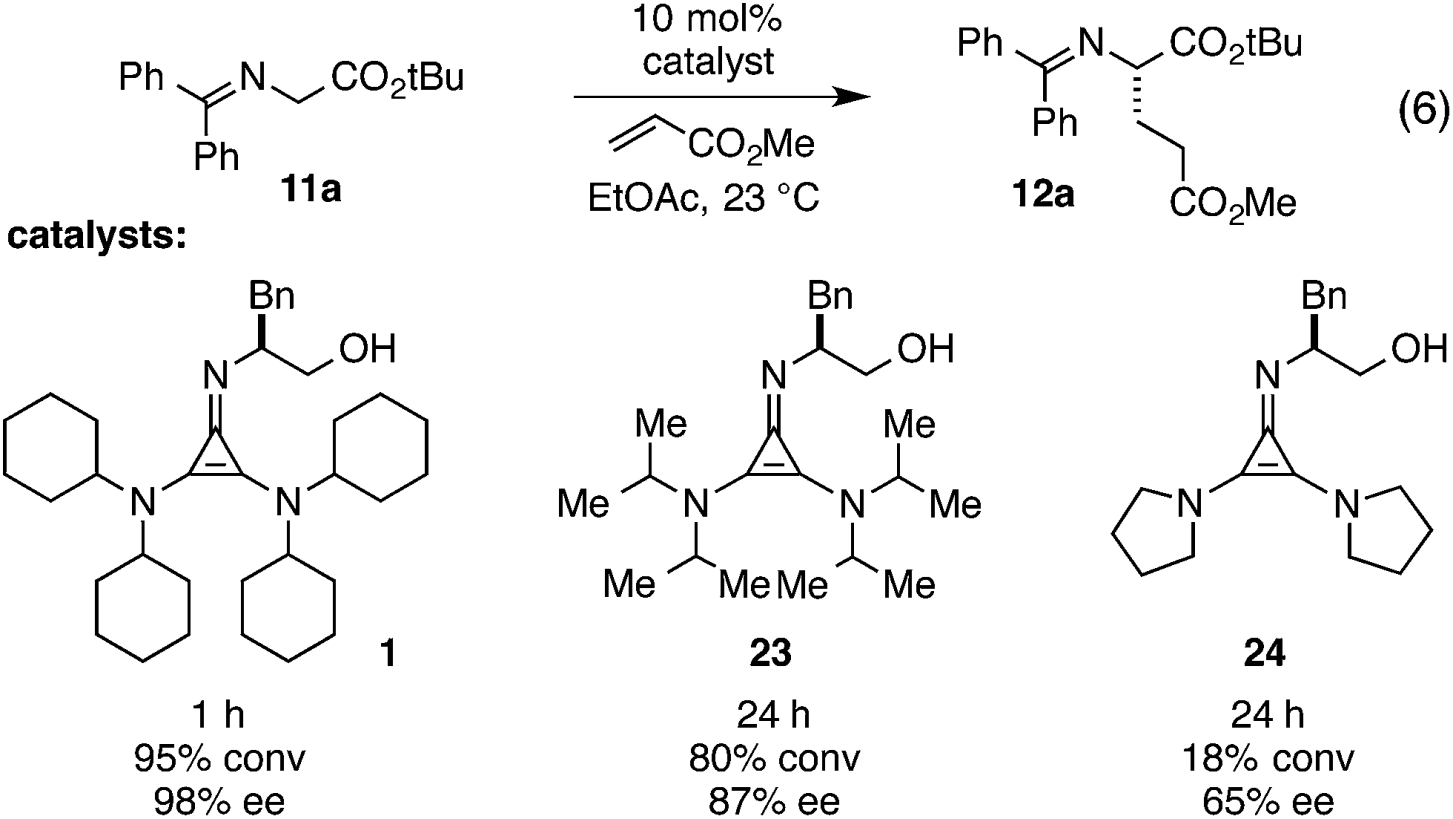


An illustration of the importance of the dicyclohexylamino substituents is shown in eqn (6). Thus while catalyst **1** effects the Michael addition of **11a** to methyl acrylate in 1 hour with 98% ee, the diisopropylamino-bearing catalyst **23** proceeds to only 80% conversion after 24 h and results in significantly reduced enantioselectivity (87% ee). Pyrrolidinyl catalyst **24** is even less reactive, resulting in only 18% conversion over 24 h and low product enantioselectivity.

**Fig. 4:**
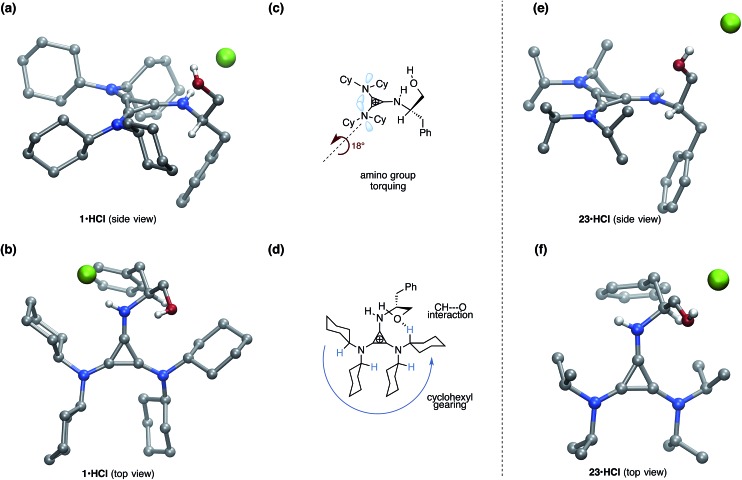
Molecular structure of (a) **1**·HCl side view; (b) **1**·HCl top view; (c) depiction of amino group torquing phenomenon of **1**·HCl; (d) depiction of cyclohexyl gearing effect and key CH···O interaction of **1**·HCl; (e) **23**·HCl side view; and (f) **23**·HCl top view. For (a) and (b) a co-crystallized molecule of H_2_O has been omitted for clarity. For (e) and (f) the hydroxymethyl group was disordered and only one of the crystal forms is shown for clarity. A molecule of co-crystallized benzene was also removed for **23**·HCl. The unmodified structures are included in the ESI file.†

To better understand these results, we obtained single-crystal X-ray structures (Parkin group, Columbia University) of **1**·HCl (Fig. 4a and b) and **23**·HCl (Fig. 4e and f). The structure of **1** differs conformationally from that of **23** in several key ways that can be readily attributed to the difference between the cyclohexyl and isopropyl substituents, and we hypothesize that these differences might manifest in the observed discrepancies in reactivity and selectivity between **1** and **23**. First, it can be seen in Fig. 4a and c that the cyclohexyl rings induce torqueing (∼18°) of the 2,3-amino substituents relative to the cyclopropenium ring, a phenomenon that has been previously reported for the tris(diisopropylamino)cyclopropenium ion.^18^ This torqueing clearly decreases the overlap of the amine lone pairs with the cyclopropenium ring, and thus readily explains the lower basicity of this cyclopropenimine in comparison to the isopropyl-substituted structure, which experiences no such torqueing (Fig. 4e).

**Fig. 5:**
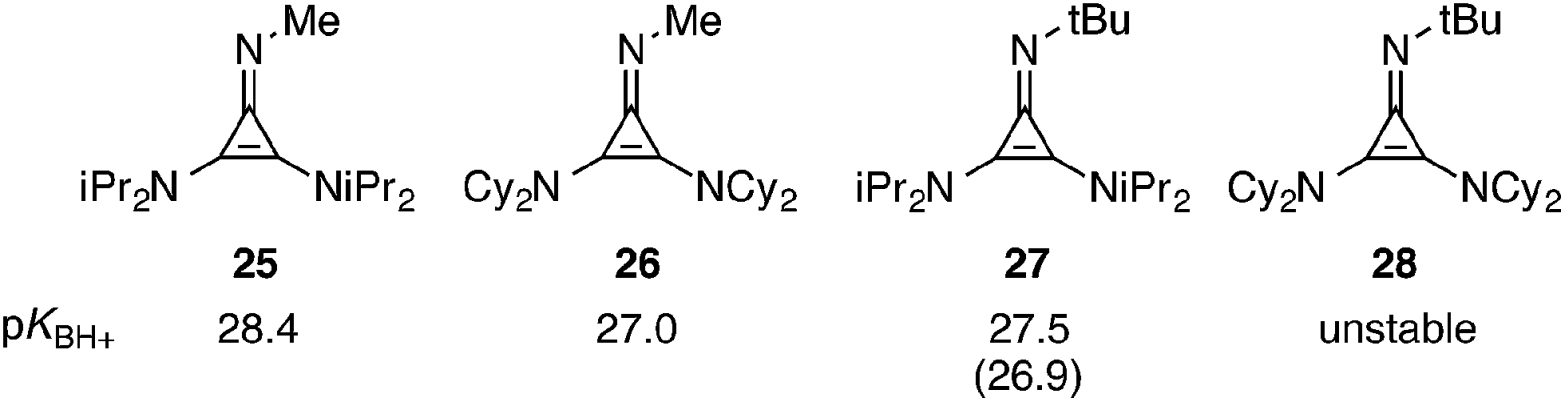
Basicities of representative cyclopropenimines. The p*K*_BH^+^_ values were determined by ^1^H NMR in *d*_3_-MeCN in reference to the P_1_–*t*Bu phosphazene base. The number in parentheses is the value previously determined by an alternative method.

To quantify this difference in basicity, we prepared the N–Me cyclopropenimines **25** and **26** and determined their p*K*_BH^+^_ values by measuring (^1^H NMR) their equilibrium with the known P_1_–*t*Bu phosphazene base (Fig. 5). By this analysis, the isopropyl-substituted imine **25** is 1.5 units more basic than the cyclohexyl-substituted imine **26**. Incidentally, N–Me imine **25** was found to be greater than 1 p*K* unit more basic than the N–*t*-Bu analogue **27**, which suggests there is a notable steric effect of the imino head group on basicity. The basicity of the N–*t*-Bu cyclohexyl-substituted imine **28** could not be determined because it is apparently unstable, presumably due to severe steric conflict.

It is not clear whether the decreased basicity of **1** is in any way responsible for its greater reactivity *versus* **23**, or whether the correlation is merely circumstantial. One possible explanation for why the less basic cyclopropenimine catalyst **1** is more reactive than its isopropyl analogue is that the diminished electron density of the π-system of **1** results in better H-bond donor capability for the N–H function of the protonated catalyst. Since hydrogen bond activation and organization by this N–H group is almost certainly operative in the rate and enantiodetermining transition state for the reactions we have demonstrated,^4^ it stands to reason that reaction rate and enantioselectivity would be correlated to the H-bond donating capacity of this group. While this hypothesis seems reasonable, as discussed below, computational studies^4^ have suggested that the major enantiomer transition state for the Michael reaction shown in eqn (1) involves the glycinate enolate H-bonded to the N–H function of the protonated catalyst, rather than to the O–H group. In this scenario, the increased acidity of the N–H group would *stabilize* the enolate and therefore presumably reduce the reaction rate. Thus we conclude that the amino torqueing phenomenon is not directly responsible for the greater reactivity of **1**, at least insofar as it impacts the electronic nature of the N–H group.

The second notable conformational feature of **1** arising from the cyclohexyl rings can be readily seen in the top view structure (Fig. 4b). Specifically, the cyclohexyl rings are all geared in the same direction, a feature not present in the isopropyl-substituted structure (Fig. 4f). One of the consequences of this gearing effect is that significantly more steric congestion is present next to the N–H function in **1** than in **23**, which could plausibly impact the transition state organization of a substrate H-bonded to this group. However, we do not at this time have evidence, either computational or experimental, for any such impact.

The second and more attributable structural consequence of the cyclohexyl gearing effect is that one of the cyclohexyl rings is predisposed to engage in a CH···O interaction with the hydroxyl of the phenylalaninol substituent, an effect first suggested through computational analysis.^4^ This interaction is apparent in the orientation of the hydroxymethyl substituent in **1** (Fig. 4b) and in the distance between the oxygen and the implied cyclohexyl α-hydrogen (2.51 Å). Although this interaction is undoubtedly weak, the gearing of the cyclohexyl rings clearly predisposes the C–H bond in a favorable orientation, removing much of the entropic penalty that might otherwise offset it. Vetticatt and we propose^4^ that the presence of this interaction provides an important organizational element that contributes to the high levels of enantioselection observed with this catalyst.

Although CH···O hydrogen bonds are weak (∼0.5 kcal mol^–1^), they are known to be an important structural factor in enzymes,^19^ other biomolecules,^20^ and supramolecular complexes.^21^ In addition, such interactions have been proposed in numerous examples in the field of asymmetric catalysis.^22^ These proposed interactions are typically between catalyst and substrate and have been posited primarily to rationalize transition state organization. In contrast, the present case represents an example in which the presence of a CH···O interaction is also part of the ground state structure of the catalyst itself.^23^

Theoretical calculations suggest this interaction is also present in the transition state for the addition of glycine imine to methyl acrylate, with the H···O distance undergoing compression to ∼2.2 Å.^4^ Calculated transition states lacking this interaction were found to be much higher in energy (>10 kcal mol^–1^), lending confidence to this proposal.

### The role of the catalyst chiral substituent

For the final part of our catalyst SAR study, we examined variation of the chiral imino substituent. Interestingly, we found that the size of this group had relatively little impact on catalyst efficiency or enantioselectivity. For example, catalysts derived from phenylalaninol (**1**), alaninol (**29**), and valinol (**30**) operated with relatively minor differences in reaction rate and enantioselectivity (Table 4, entries 1–3). As can be appreciated from the crystal structures in Fig. 4a and b, we believe this chiral substituent is conformationally locked to minimize steric conflict with the dicyclohexylamino substituent, and that this conformation is further stabilized by the CH···O interaction discussed above. From this point of view, it is reasonable that the size of the substituent (Bn, Me, i-Pr) would have minimal impact on catalyst operation. On the other hand, the catalyst derived from phenylglycinol (**31**) was significantly less reactive and selective (entry 4), perhaps due to the electron-withdrawing nature of the phenyl substituent.

**Table 4:** Screen of chiral substituent in the cyclopropenimine-catalyzed enantioselective Michael reaction^*a*^

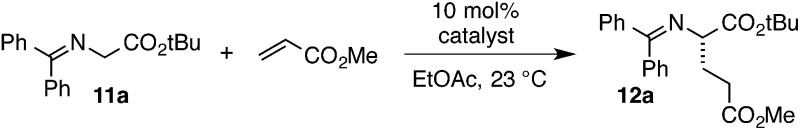
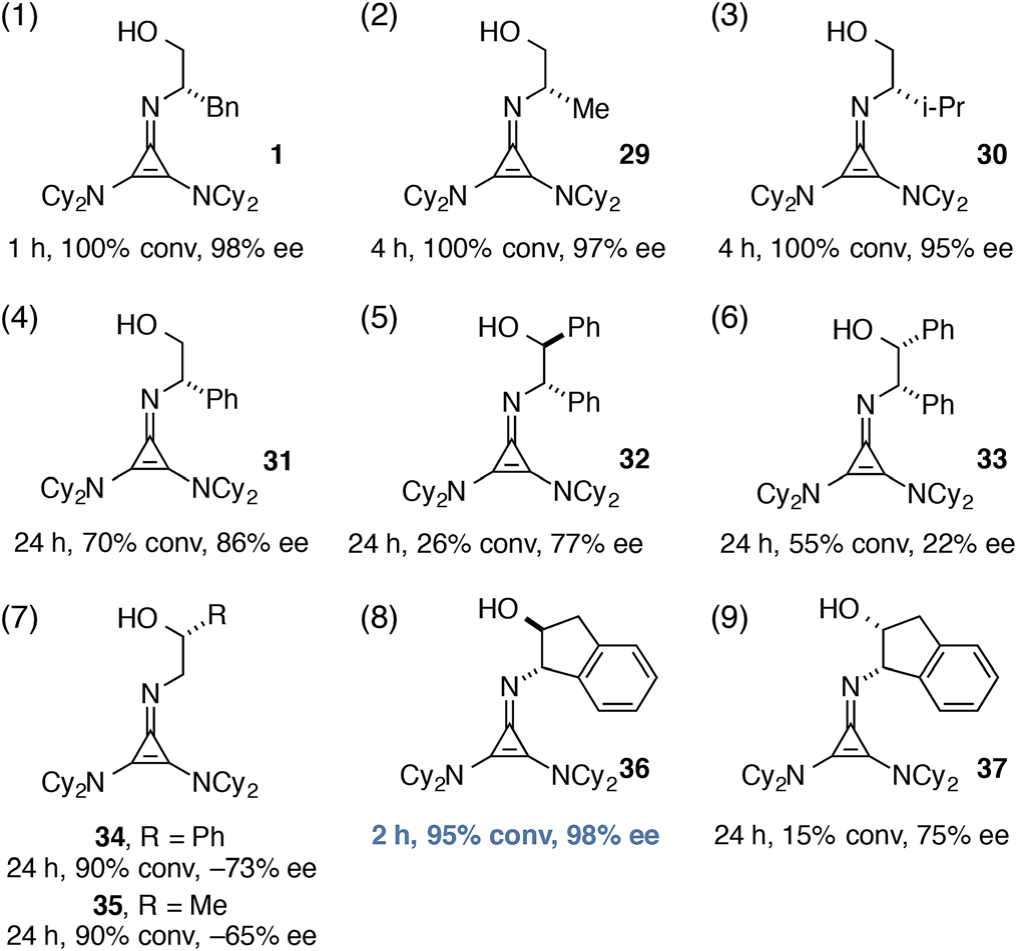

^*a*^Conversion determined by ^1^H NMR based on Bn_2_O standard. Enantiomeric excesses (ee) were determined by chiral HPLC.

A comparison of catalysts bearing vicinal stereocenters, **32** and **33**, revealed divergent matched-mismatched profiles, with **32** being slower but more selective than **33** (entries 5 and 6). Interestingly, use of cyclopropenimine **34**, which bears only a single stereocenter one carbon removed from the imino nitrogen (entry 7, R = Ph), resulted in product with appreciable enantioenrichment despite the lack of an obvious conformational lock. We believe the ability of **34** to induce asymmetric organization is understandable in the context of the CH···O interaction discussed above, which serves as a pseudo-ring that biases the catalyst toward one major conformation. Even the methyl-substituted catalyst **35** induced enantioselectivity of 65% (entry 7, R = Me), a notable level for such a succinct stereochemical motif.

Perhaps the most important finding from this portion of our SAR study was the identification of the 1-aminoindanol-derived cyclopropenimine **36**, the effectiveness of which nearly rivalled that of **1** (entry 8). As will be discussed (see Catalyst stability studies below), the long-term stability of **36** is much improved over **1**, making **36** an important addition to the chiral cyclopropenimine arsenal. Not surprisingly, the *cis*-aminoindanol catalyst **37** was significantly worse in terms of efficiency and selectivity (entry 9).

### Mechanistic rationale

**Fig. 6:**
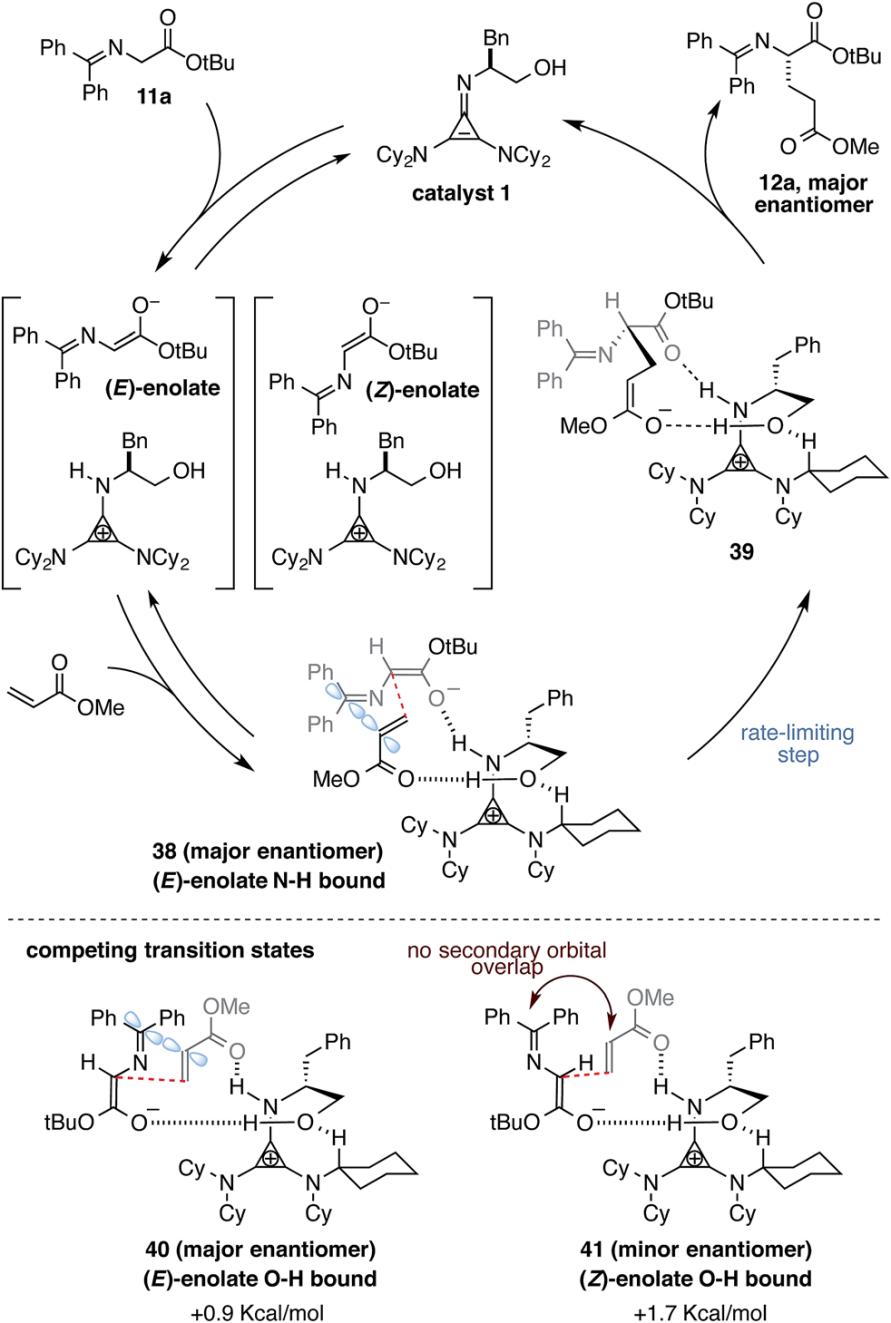
Mechanistic rationale.

Based on the results described above, along with other mechanistic and computational insights, we propose the following model for the operation of catalyst **1** in the enantioselective Michael addition of glycinate **11a** to methyl acrylate (Fig. 6). The first step in the catalytic cycle involves deprotonation of glycinate **11a** by the catalyst **1** to produce either the (*E*)- or (*Z*)-cyclopropenium enolate complex. The organization of these intermediate complexes is not currently known, however, because the *K*_eq_ of proton transfer lies heavily in favor of the starting material, making spectroscopic analysis challenging. Nevertheless, computational studies^4^ have suggested that the lowest-energy enantiodetermining transition state involves the (*E*)-enolate H-bonded to the N–H function of the protonated catalyst, with the acrylate H-bonded to the catalyst hydroxyl group (*cf.* **38**). Natural abundance kinetic isotope analysis^4^ of this reaction has demonstrated conclusively that this conjugate addition step is also rate-limiting. The resulting enolate intermediate **39** is expected to undergo rapid protonation to produce the observed major enantiomeric product **12a** while returning the cyclopropenimine **1** to the catalytic cycle.

Interestingly, calculations suggested that a competing transition state **40**, which is only 0.9 Kcal mol^–1^ higher in energy than **38**, involves the (*E*)-enolate H-bonded to the hydroxyl function and the acrylate activated by the N–H group. Since it also involves addition to the si face of the (*E*)-enolate, this transition state increases the level of enantioselection observed in this reaction. The next lowest energy transition state, **41**, leading to the minor enantiomeric product, involves the same H-bonding organization as **40**, but with the (*Z*)-enolate geometry. As to why **38** and **40** are lower in energy than **41**, the answer is undoubtedly complex. However, from a close analysis of the computed transition states, we hypothesize that **38** and **40** experience a secondary orbital interaction between the acrylate α-carbon and the C=N carbon of the enolate (Fig. 6, blue orbitals), which serves to stabilize the incipient anionic charge on the acrylate fragment. Transition state **41**, which involves the (*Z*)-enolate, is not capable of such an interaction. In any case, the competition between not only enolate geometries but also N–H *vs.* O–H binding modes underscores the complexity of factors that lead to successful enantioselection with this catalyst.

### Catalyst stability studies

During the course of our initial development studies, we noticed a slow but appreciable decrease in the efficiency of catalyst **1** when it was stored in its free base form. Further analysis revealed that **1** undergoes rearrangement to oxazoline **43**, likely *via* intermediate **42** (eqn (7)).
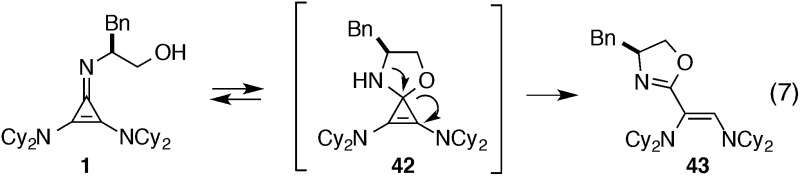


The half-life for this process was found to be approximately 15 days at room temperature (Table 5, entry 1a), and 8 months at –20 °C (entry 1b). As the HCl salt, **1** is essentially indefinitely stable (entry 1c). Under conditions relevant to catalysis (*i.e.* 0.035 M in PhMe), however, **1** decomposes to the inactive species, **43**, with a half-life of only 7 h (entry 1d).^24^ Although the very short reaction times we have observed using catalyst **1** partially mitigate this instability issue, an eye toward broader application of these types of cyclopropenimine catalysts clearly compels the identification of more stable structures. In this regard, while isoleucinol-derived catalyst **30** was found to be significantly less stable than **1**, with a half-life of only 16 h in solid form at rt (entry 2), alaninol-derived catalyst **29** had markedly improved stability, with a half-life of 5 months (entry 3). Since the only obvious trend between catalysts **1**, **30**, and **29** is a correlation of rearrangement rate to substituent size, we speculate that more sterically demanding substituents lead to angle compression, akin to the *gem*-dimethyl (Thorpe-Ingold) effect, which thus accelerates the conversion to intermediate **42** and hence to **43** (eqn (7)).

**Table 5:** Catalyst stability screen^*a*^

Entry	Catalyst	Conditions	*t*_1/2_
1	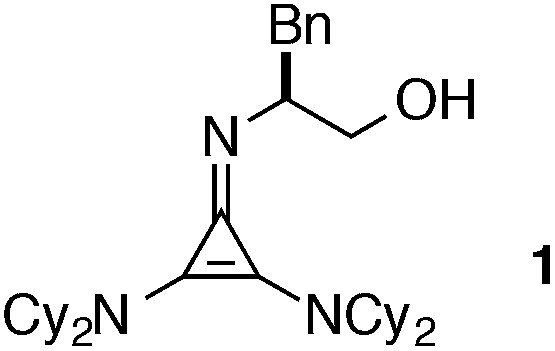	(a) Solid rt	15 days
		(b) Solid –20 °C	8 months
		(c) HCl salt, rt	>5 years
		(d) 0.035 M PhMe	7 h
2	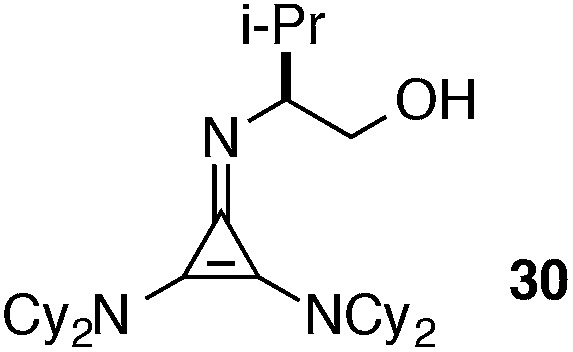	Solid, rt	16 h
3	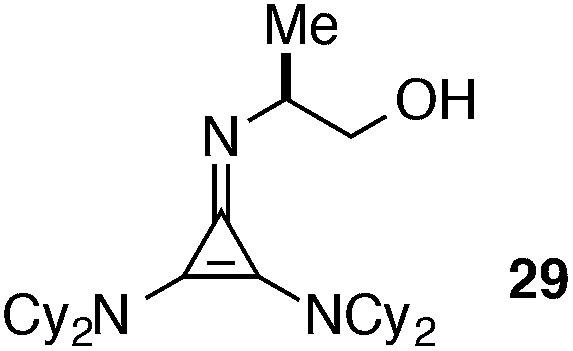	Solid, rt	5 months
4	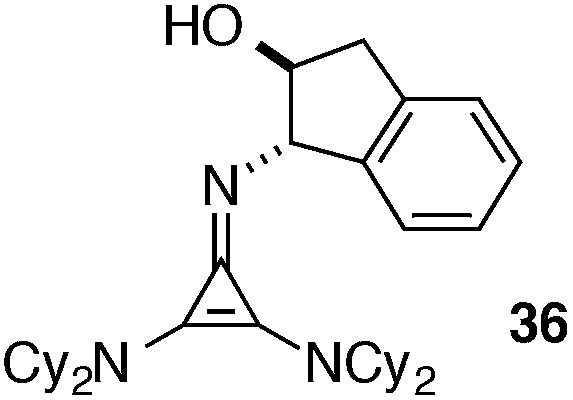	(a) Solid, rt	>5 years
		(b) 0.035 M	36 h
5	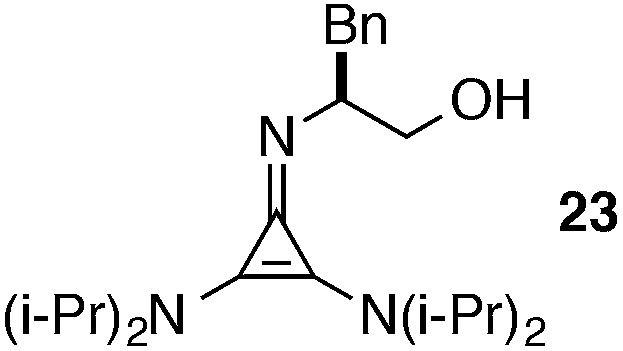	(a) Solid, rt	12 h
		(b) 0.035 M	3 h

^*a*^Decomposition monitored by ^1^H NMR; see ESI for method used for each cyclopropenimine.

**Chart 1:**
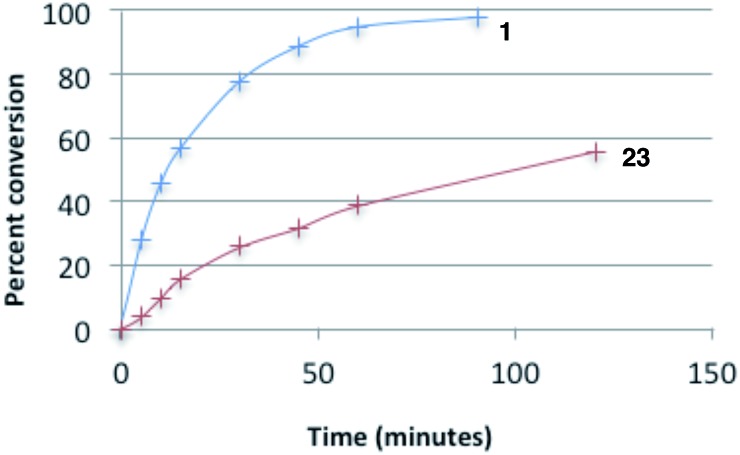
Conversion rates of catalysts **1** and **23** for the Michael reaction of **11a**.

Most notably, indanol-derived catalyst **36**, which showed outstanding performance in the Michael addition reaction (*cf.* Table 4) was found to have significantly improved stability (Table 5, entry 4). As a solid at room temperature, we have observed no decomposition over 3 months, leading to a calculated half-life of at least 5 years (entry 4a). In solution, **36** does undergo decomposition, but with a notably improved half-life of 36 h (entry 4b). Although further efforts to increase catalyst stability are clearly warranted, the improvements offered by structures **29** and **36** make the storage (as free bases) and use of these catalysts significantly more convenient.^25^ Notably, we found the stability of catalyst **23** to be significantly lower than **1** (entries 1 *vs.* 5), further underscoring the benefit of the switch from diisopropylamino to dicyclohexylamino substituents. It is worth noting that although part of the difference in catalyst efficiency observed between cyclopropenimine **1** and **23** (see eqn (6)) can be attributed to the faster decomposition of the latter, catalyst **1** is inherently faster, as can be seen from a comparison of the initial rates of reaction (Chart 1). In fact, the initial rate (10 min) of reaction with the bis(dicyclohexylamino) catalyst **1** is nearly five times as fast as with **23**.

### Michael addition: expanded substrate scope

Finally, we undertook further exploration of the substrate scope of the enantioselective Michael addition of glycine imine **11a** using the new catalyst **36** as shown in Table 6. Notably, dimethyl fumarate participated well in this chemistry to furnish the *anti*-Michael adduct in 93% yield with >20 : 1 dr and 96% ee after 3 h (entry 1). Dimethyl maleate, on the other hand, was essentially unreactive (entry 2). Certain unsaturated ketones were found to be viable substrates for this chemistry, including 4-phenylbut-3-en-2-one (entry 3) and chalcone (entry 4). Heteroaryl products containing furyl (entry 5) or thiophenyl (entry 6) substituents were also accessible in high yield and with high stereoselectivities. A bis-unsaturated acceptor proved to be somewhat poorly reactive with catalyst **36** (entry 5); however, efficient reaction with this substrate was achieved by reverting to catalyst **1**, which resulted in exclusive 1,4-selectivity to produce the β-styrenyl Michael adduct with >20 : 1 dr and 93% ee (entry 7). It should be noted that this 1,4-selectivity contrasts with the 1,6- and 1,8-regioselectivites observed in related reactions.^26^ Finally, β-alkyl substitution in the Michael adduct could be achieved by using an alkylidine malonate electrophile (entry 8). The resulting product was obtained in essentially quantitative yield and with high stereoselectivity.

**Table 6:** Substrate scope studies of cyclopropenimine-catalyzed Michael reaction with catalyst **36** ^*a*^

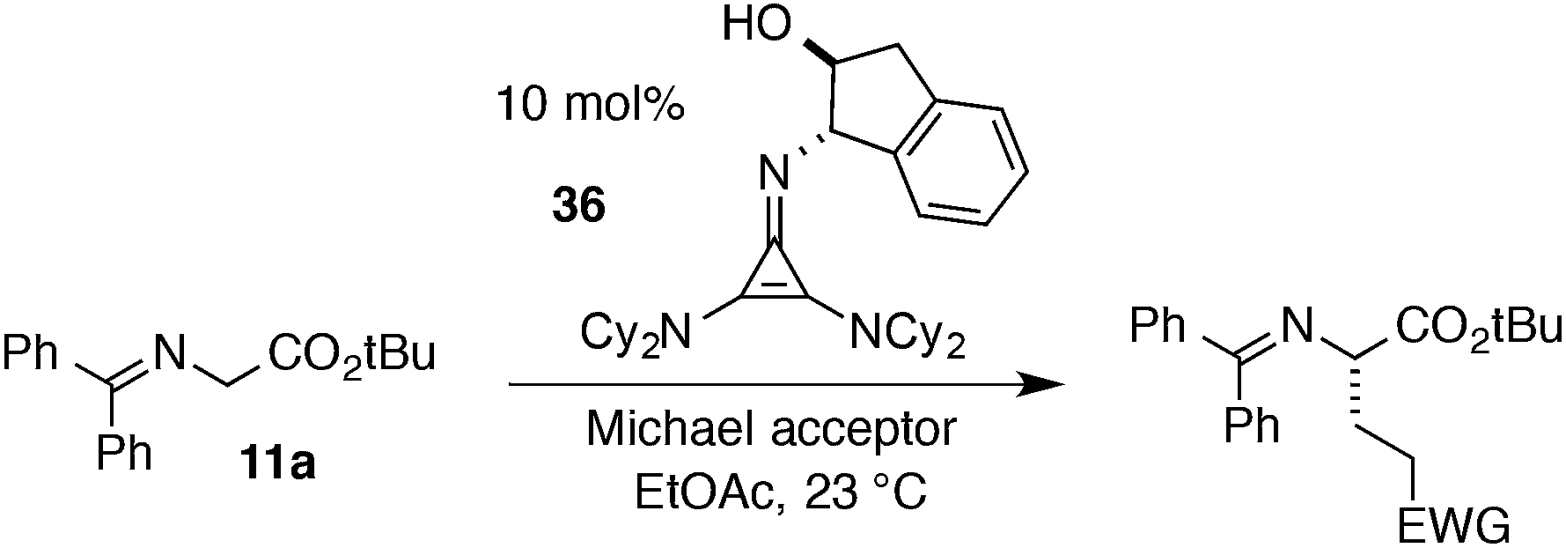						
Entry	Michael acceptor	Product	Time (h)	Yield (%)	dr	ee (%)
1	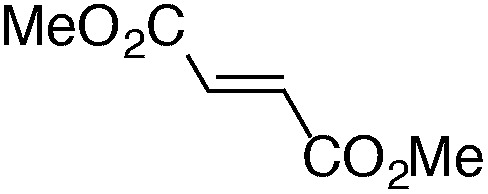	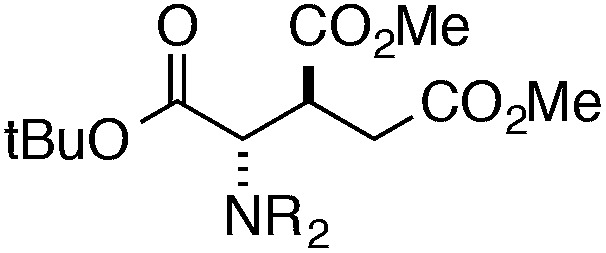	3	94	>20 : 1	96
2	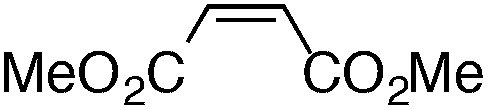	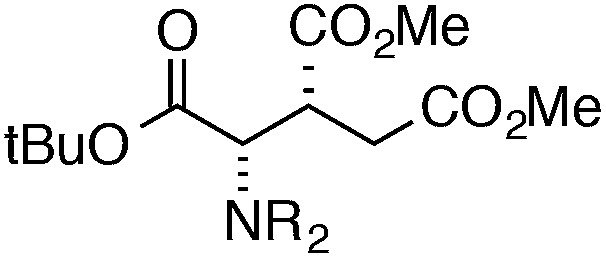	48	<5	—	—
3	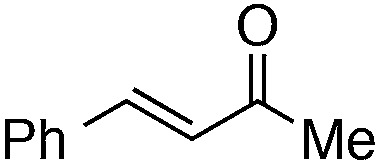	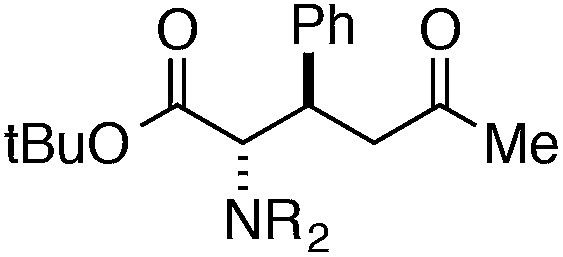	7	99	>20 : 1	91
4	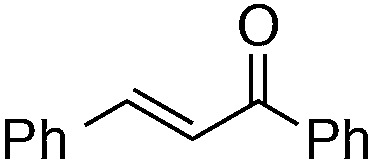	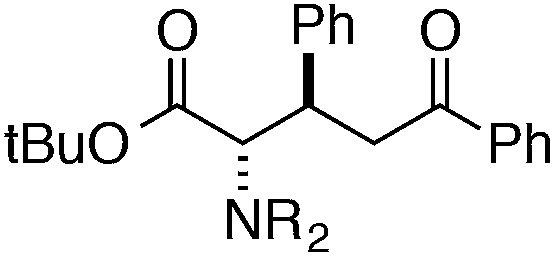	2	99	>20 : 1	96
5	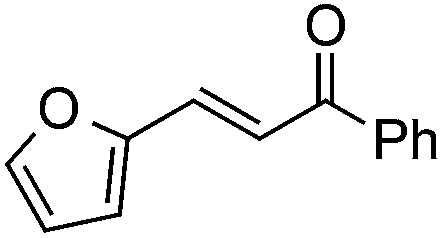	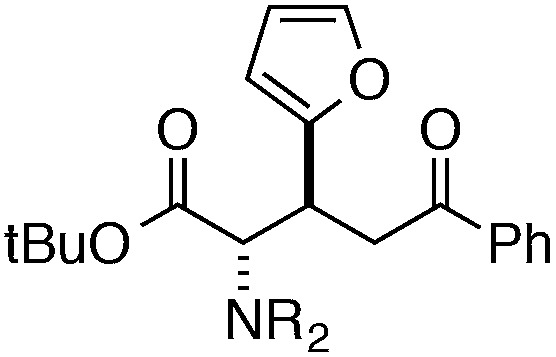	7	99	>20 : 1	96
6	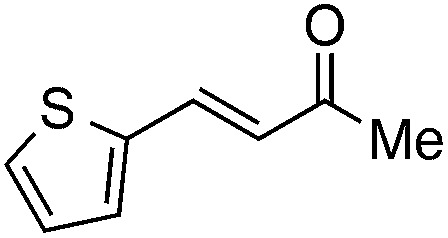	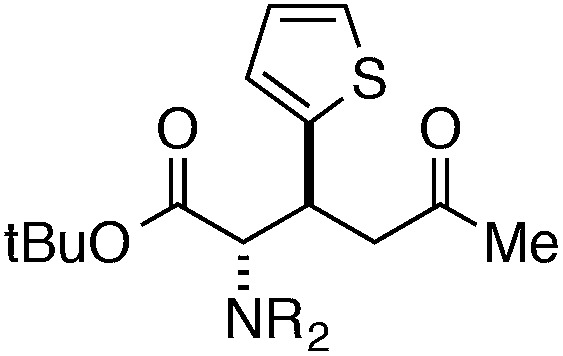	48	94	>20 : 1	88
7^*b*^	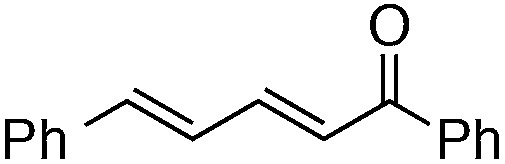	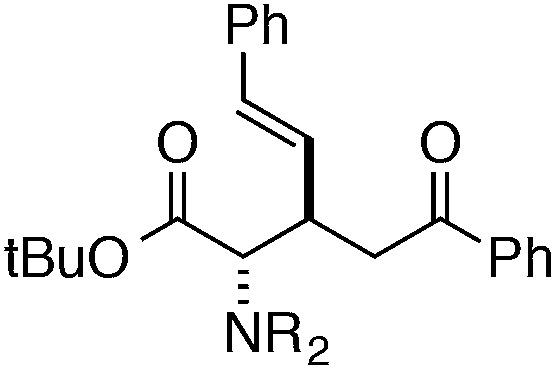	8	63 (with catalyst **1**)	>20 : 1	93
8	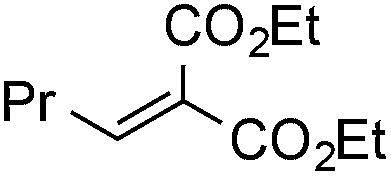	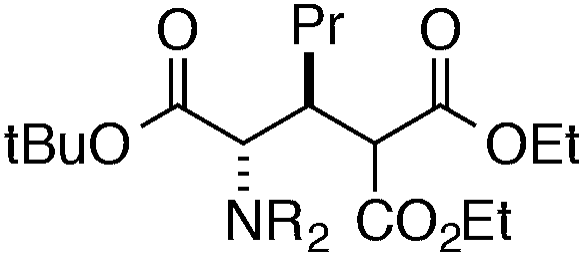	22	99	9 : 1	92

^*a*^Yield based on isolated and purified product. Diastereomeric ratios (dr) and enantiomeric excesses (ee) were determined by chiral HPLC.

^*b*^1,4-Addition : 1,6-addition ratio ≥20 : 1.

## Conclusions

The 2,3-diaminocyclopropenimine framework offers a unique new catalyst platform for enantioselective Brønsted base catalysis. The potent basicity of the cyclopropenimine scaffold clearly contributes to the effectiveness of these catalysts for certain applications. However, as our work has demonstrated, basicity is by no means the sole contributor to catalyst efficiency, as H-bonding ability and other organizational elements also play a crucial role. Most striking is the impact of the dicyclohexylamino substituents on the optimal catalyst efficiency, which serve to modulate both the electronic and conformational properties of the catalyst framework in unexpected ways. Future efforts will be aimed at exploiting the information gained from this work for the development of other chiral cyclopropenimine catalysts and applying these catalysts to enantioselective transformations of high value.

## References

[cit1] (a) Superbases for Organic Synthesis: Guanidines, Amidines, and Phosphazenes and Related Organocatalysts, ed. T. Ishikawa, John Wiley and Sons, 2009.

[cit2] Bandar J. S., Lambert T. H. (2012). J. Am. Chem. Soc..

[cit3] Bandar J. S., Lambert T. H. (2013). J. Am. Chem. Soc..

[cit4] Bandar J. S., Sauer G. S., Wulff W. D., Lambert T. H., Vetticatt M. J. (2014). J. Am. Chem. Soc..

[cit5] Kotke M. and Schreiner P. R., (Thio)urea Organocatalysts, in Hydrogen Bonding in Organic Synthesis, ed. Petri M. Pihko, Wiley-VCH, 2009, pp. 141–251.

[cit6] Chinchilla R., Nájera C., Sánchez-Agulló P. (1994). Tetrahedron: Asymmetry.

[cit7] Iyer M. S., Gigstad K. M., Namdev N. D., Lipton M. (1996). J. Am. Chem. Soc..

[cit8] Corey E. J., Grogan M. J. (1999). Org. Lett..

[cit9] Ishikawa T., Araki Y., Kumamoto T., Seki H., Fukuda K., Isobe T. (2001). Chem. Commun..

[cit10] Terada M., Ube H., Yaguchi Y. (2006). J. Am. Chem. Soc..

[cit11] Leow D., Tan C.-H. (2010). Synlett.

[cit12] Uraguchi D., Sakaki S., Ooi T. (2007). J. Am. Chem. Soc..

[cit13] Takeda T., Terada M. (2013). J. Am. Chem. Soc..

[cit14] Núñez M. G., Farley A. J. M., Dixon D. J. (2013). J. Am. Chem. Soc..

[cit15] Yoshida Z., Tawara Y. (1971). J. Am. Chem. Soc..

[cit16] Tobey S. W., West R. (1966). J. Am. Chem. Soc..

[cit17] Brak K., Jacobsen E. N. (2013). Angew. Chem., Int. Ed..

[cit18] Butchard J. R., Curnow O. J., Pipal R. J., Robinson W. T., Shang R. (2008). J. Phys. Org. Chem..

[cit19] Weiss M. S., Brandl M., Suhnel J., Pal D., Hilgenfeld R. (2001). Trends Biochem. Sci..

[cit20] Zierke M., Smiesko M., Rabbani S., Aeschbacher T., Cutting B., Allain F. H.-T., Schubert M., Ernst B. (2013). J. Am. Chem. Soc..

[cit21] Desiraju G. R. (1996). Acc. Chem. Res..

[cit22] Lippert K. M., Hof K., Gerbig D., Ley D., Hausmann H., Guenther S., Schreiner P. R. (2012). Eur. J. Org. Chem..

[cit23] Armacost K., Acevedo O. (2014). J. Am. Chem. Soc..

[cit24] The rate of decomposition is unaffected by the presence of *tert*-butyl glycinate benzophenone imine

[cit25] After freebase storage at 23 °C for one month cyclopropenimine **1** retained less than 5% of its original activity while cyclopropenimine **36** showed no decrease in reactivity

[cit26] Bernardi L. López-Cantarero J. Niess B. Jørgensen K. A., J. Am. Chem. Soc., 2007, 129 , 5772 , . See also ref. 12 . 17411052 10.1021/ja0707097

