# Supplementary material for: Structure–activity relationship studies of cyclopropenimines as enantioselective Brønsted base catalysts
Source: Chem Sci. 2014 Dec 19;6(2):1537–47. doi: 10.1039/c4sc02402h (PMC4618405; doi:10.1039/c4sc02402h)
Supplement: Supplementary file 1 [file SC-006-C4SC02402H-s001.pdf]

Electronic Supplementary Material (ESI) for Chemical Science.
This journal is © The Royal Society of Chemistry 2014

Supporting Information

Structure-Activity Relationship Studies of Cyclopropenimines as Enantioselective Brønsted Base Catalysts

Jeffrey S. Bandar, Alexandre Barthelme, Alon Y. Mazori and Tristan H. Lambert*

Department of Chemistry, Columbia University, New York, NY 10027

General information. All reactions were performed open to the atmosphere, unless otherwise noted. Methylene chloride, diethyl ether, tetrahydrofuran and toluene were dried using a J.C. Meyer solvent purification system. All other solvents and commercial reagents were used as provided, unless otherwise noted. Flash column chromatography was performed employing 40-63 µm silica gel (SiliaFlash P60 from Silicycle). Thin-layer chromatography (TLC) was performed on silica gel 60 F_{254} plates (EMD). Organic solutions were concentrated using a Buchi rotary evaporator.

^{1}H and ^{13}C NMR spectra were recorded in $CDCl_3$ (except where noted) on Bruker DRX-300, DRX-400 or DRX-500 spectrometers as noted. Data for ^{1}H NMR are reported as follows: chemical shift (δ ppm), multiplicity (s = singlet, br s = broad singlet, d = doublet, t = triplet, q = quartet, dd = doublet of doublets, td = triplet of doublets, m = multiplet), coupling constant (Hz), integration, and assignment. Data for ^{13}C and ^{19}F NMR are reported in terms of chemical shift. Infrared spectra were recorded on a Nicolet Avatar 370DTGS FT-IR. Optical rotations were measured using a Jasco DIP-1000 digital polarimeter. High-resolution mass spectra were obtained from the Columbia University Mass Spectrometry Facility on a JEOL JMSHX110 HF mass spectrometer using FAB+ ionization mode. Low-resolution mass spectrometry (LRMS) was performed on a JEOL JMS-LCmate liquid chromatography spectrometer system using APCl+ ionization technique. High-resolution mass spectra were obtained from the Columbia University Mass Spectrometry Facility on a JEOL JMSHX110 HF mass spectrometer using FAB+ ionization mode HPLC analysis was performed on an Agilent Technologies 1200 series instrument with a Daicel Chiralpak AD-H or OD-H chiral column (25 cm) using the given conditions.

Note: For full details on the synthesis and characterization of compounds **1**, **16**, Table 2 (entries 2, 4 and 5), **23**, **27**, **43**, Table 5 (entry 7) see "Enantioselective Brønsted Base Catalysis with Chiral Cyclopropenimines." Bandar, J. S.; Lambert, T. H. *J. Am. Chem. Soc.* **2012**, *134*, 5552-5555.

Catalyst Syntheses

Cyclcopropenimine 1 catalyst: This is an optimized large-scale procedure for synthesis of the standard cyclopropenimine catalyst **1**. For synthesizing cyclopropenimine derivatives, chloro[bis(dicyclohexylamino)]cyclopropenium chloride was prepared and used as a common starting material as shown below.

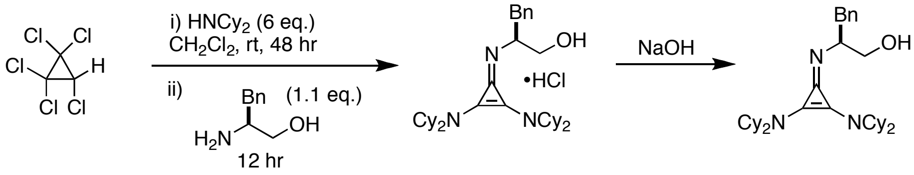

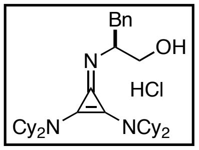

(*S*)-2-(2,3-Bis(dicyclohexylamino)cyclopropenimine)-3-phenylpropan-1-ol hydrochloride (1•HCl). Dicyclohexylamine (108 mL, 541 mmol, 6.0 equiv) was slowly added to a solution of pentachlorocyclopropane[1] (19.3 g, 90.2 mmol, 1.0 equiv) in CH_2Cl_2 (900 mL) in a 3L round bottom flask. A white precipitate formed as the reaction mixture was stirred for a further 48 hr at room temperature. Next, (*S*)-2-amino-3-phenylpropan-1-ol[2] (15.0 g, 99.2 mmol, 1.1 equiv) was added in one portion and the reaction mixture was stirred for an additional 12 hr. The crude reaction mixture was filtered through a celite plug, then washed with 1.0 M HCl (3 x 500 mL), dried with anhydrous sodium sulfate and concentrated *in vacuo* to yield crude cyclopropenimine hydrochloride salt as an off-white solid. Recrystallization from hot ethyl acetate/hexanes (approximately 1/2) yielded pure cyclopropenimine hydrochloride salt as a white solid (38.5 g, 73% yield). ^{1}H NMR (400 MHz, $CDCl_3$) δ 7.35 (d, 9.2 Hz, 1H, N**H**), 7.0-7.15 (m, 5H, Ar**H**), 5.17 (t, 5.6 Hz, 1H, -O**H**), 3.60-3.85 (m, 3H, NC**H**BnC**H₂**OH), 3.10 (m, 4H, NCy**H**), 2.80-3.00 (m, 2H, -C**H₂**Ph), 1.00-1.70 (m, 40H, Cy**H**). ^{13}C NMR (100 MHz, $CDCl_3$) δ 137.8, 129.2, 128.0, 126.2, 116.5, 114.5, 63.7, 61.3, 59.1, 38.4, 32.1, 31.9, 25.4, 24.4. IR (thin film, cm^{-1}) 3175, 2929, 2855, 1498, 1450, 1372, 1317, 1181, 894, 727, 697. $[\alpha]^{20}_D$ = -38.9 (1.0 c, $CHCl_3$). LRMS (APCI+) m/z = 546.43 calcd for $C_{36}H_{56}N_3O$ $[M+]^+$, found 546.98.

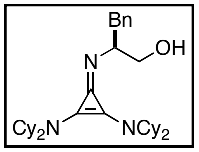

(*S*)-2-(2,3-Bis(dicyclohexylamino)cyclopropenimine)-3-phenylpropan-1-ol (1). Cyclopropenimine freebase was prepared and stored in a freezer on a weekly basis. Pure cyclopropenimine freebase was quantitatively obtained by dissolving the corresponding hydrochloride salt in CH_2Cl_2 and washing the solution with 1.0 M NaOH (3 x), drying with anhydrous sodium sulfate and concentrating *in vacuo*. The cyclopropenimine is obtained as an off-white solid. ^{1}H NMR (400 MHz, $CDCl_3$) δ 7.10-7.25 (m, 5H, Ar**H**), 3.79 (m, 1H, NC**H**BnCH₂OH), 3.40-3.50 (m, 2H, NCHBnC**H₂**OH), 3.00-3.10 (m, 4H, NCy**H**), 2.70-2.85 (m, 2H, -C**H₂**Ph), 1.00-1.90 (m, 40H, Cy**H**). ^{13}C NMR (100 MHz, $CDCl_3$) δ 140.5, 129.7, 129.4, 127.8, 125.5, 65.1, 61.7, 58.3, 41.7, 34.4, 33.1, 32.8, 32.6, 26.3, 26.1, 25.3, 25.2.

General scheme for bis(dicyclohexylamino)-based cyclopropenimines.

[1] Tobey, S. W.; West, R. *J. Am. Chem. Soc.* **1966**, *88*, 2478.
[2] Shi, L.; Chen, L.; Chen, R.; Chen, L. *J. Label Compd. Radiopharm.* **2010**, *53*, 147-151.

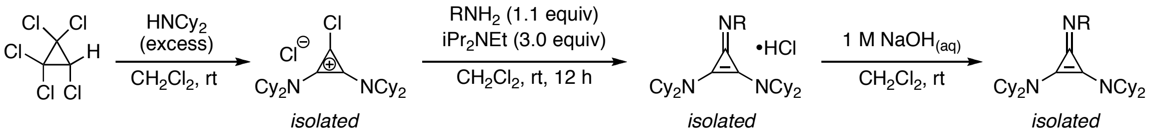

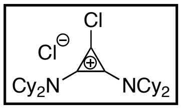

Chloro[bis(dicyclohexylamino)]cyclopropenium chloride (15). Dicyclohexylamine (186 mL, 936 mmol, 6.0 equiv) was slowly added to a solution of pentachlorocyclpropane (33.4 g, 156 mmol, 1.0 equiv) in dichloromethane (1.6 L, 0.1 M). The resulting solution was stirred at room temperature for a further 48 hours (hr) during which time a white precipitate formed. At this time concentrated HCl (200 mL) was added to precipitate excess dicyclohexylamine. The reaction mixture was filtered and the filtrate was washed with 1.0 M HCl (3 x 500 mL and 3 x 250 mL), dried with Na_2SO_4 and concentrated *in vacuo*. The resulting crude solid was washed with hot ethyl acetate (3 x 200 mL) to yield the pure title compound as a white powder (60.0 g, 128 mmol, 82% yield). ^{1}H NMR (400 MHz, $CDCl_3$) δ 3.62 (m, 2H, NCy**H**), 3.44 (m, 2H, NCy**H**), 2.11-1.30 (m, 40H, Cy**H**). ^{13}C NMR (100 MHz, $CDCl_3$) 132.2, 93.5, 65.8, 56.9, 32.7, 30.8, 25.4, 25.2, 24.5, 24.4. IR (thin film, cm^{-1}) 2933, 2851, 1583, 1419, 1324, 1123, 990, 891. LRMS (APCI+) m/z = 413.37; calcd for $C_{37}H_{45}N_2O$ $[M]^+$ 413.35; note: this mass corresponds to hydrolyzed cyclopropenium depicted below:

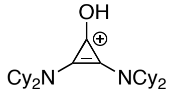

Chemical Formula: $C_{27}H_{45}N_2O^+$
Exact Mass: 413.35

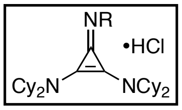

General procedure for cyclopropenimine hydrochloride salt synthesis: Chiral amine (1.10 equiv) was added to a solution of chloro[bis(dicyclohexylamino)]cyclopropenium chloride (1.00 equiv) and *N*,*N*-diisopropylethylamine (2.00-3.00 equiv) in dichloromethane (0.1 M). The reaction solution was stirred for 12 hr before being washed with 1.0 M HCl (3 x reaction volume), dried with Na_2SO_4 and concentrated *in vacuo*. The crude solid material was recrystallized from a hot mixture of specified solvents to yield the pure title compound as a white solid. All cyclopropenimines were fully characterized as their hydrochloride salt.

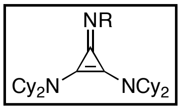

General procedure for cyclopropenimine hydrochloride deprotonation: The corresponding cyclopropenimine hydrochloride salt was dissolved in dichloromethane (approximately 0.2 M) and washed with an equal volume of 1.0 M $NaOH_{(aq)}$ (3 x). The dichloromethane solution was dried with Na_2SO_4 and concentrated *in vacuo*. Notes: (a) the temperature of the rotovap was kept at or below 20 °C; (b) the resulting solid was further dried on a vacuum pump for two hr before use; (c) the catalyst was stored in a freezer while not in use; (d) this process was repeated on a weekly basis as needed. ^{1}H and ^{13}C NMR of the obtained freebase are included below. In certain cases characterization of neutral cyclopropenimines was complicated by: cyclopropene carbons were not always observed

in the ^{13}C NMR, cyclohexane carbons appeared broad in the ^{13}C NMR, and cyclopropenimine decomposition occurred quickly; thus all cyclopropenimines were fully characterized as their hydrochloride salts prior to deprotonation.

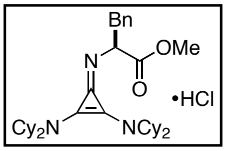

(*S*)-Methyl 2-(2,3-bis(dicyclohexylamino)cyclopropenimine)-3-phenylpropanoate hydrochloride (17•HCl). The general procedure was followed using L-phenylalanine methyl ester hydrochloride (500 mg, 2.32 mmol), chloro[bis(dicyclohexylamino)]cyclopropenium chloride (985 mg, 2.11 mmol) and *N*,*N*-diisopropylethylamine (0.90 mL, 6.33 mmol) in dichloromethane (21 mL). Recrystallization from hot ethyl acetate yielded the title product as a white solid (1.25 g, 2.05 mmol, 97% yield). ^{1}H NMR (400 MHz, $CDCl_3$) δ 8.83 (d, J = 9.0 Hz, 1H, N**H**), 7.45 (d, J = 7.1 Hz, 2H, Ar**H**), 7.26 (t, J = 7.5 Hz, 2H, Ar**H**), 7.22 (t, J = 7.3 Hz, 1H, Ar**H**), 4.45 (m, 1H, NC**H**CO_2Me), 3.95 (dd, J = 9.4 and 14.0 Hz, 1H, C**H**Ph), 3.81 (s, 3H, CO_2CH_3), 3.42 (dd, J = 3.9 and 14.0 Hz, 1H, C**H**Ph), 3.28 (m, 4H, NCy**H**), 1.90-1.25 (m, 40H, Cy**H**). ^{13}C NMR (100 MHz, $CDCl_3$) δ 171.2, 137.1, 129.6, 128.3, 126.7, 115.9, 115.5, 61.2, 59.5, 52.7, 37.0, 32.6, 32.0, 26.0, 25.5, 25.5, 24.9, 24.5. IR (thin film, cm^{-1}) 3223, 2926, 2858, 1740, 1498, 1453, 1372, 1256, 1181, 891. $[\alpha]^{20}_D$ = -34.3 (1.0 c, $CHCl_3$). HRMS (FAB+) m/z = 574.4365; calcd for $C_{37}H_{56}N_3O_2$ $[M]^+$ 574.4367.

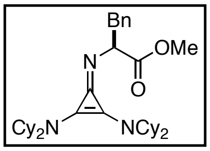

(*S*)-Methyl 2-(2,3-bis(dicyclohexylamino)cyclopropenimine)-3-phenylpropanoate (17). ^{1}H NMR (400 MHz, $CDCl_3$) δ 7.30 (t, J = 7.1 Hz, 2H, Ar**H**), 7.16 (t, J = 7.1 Hz, 2H, Ar**H**), 7.11 (t, J = 7.3 Hz, 1H, Ar**H**), 4.33 (dd, J = 5.2 and 8.2 Hz, 1H, Cp=NC**H**), 3.63 (s, 3H, CO_2CH3), 3.116 (dd, J = 5.3 and 13.1 Hz, 1H, C**H**Ph), 3.12 (m, 4H, NCy**H**), 2.95 (dd, J = 8.4 and 13.0 Hz, 1H, C**H**Ph), 1.80-1.00 (m, 40H, Cy**H**). ^{13}C NMR (100 MHz, $CDCl_3$) δ 176.0, 140.4, 130.0, 127.6, 125.6, 117.6, 67.0, 58.5, 51.4, 42.1, 33.2, 32.7, 26.4, 26.3, 26.2, 25.3.

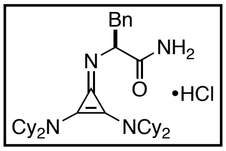

(*S*)-2-(2,3-Bis(dicyclohexylamino)cyclopropenimine)-3-phenylpropanamide hydrochloride (18•HCl). The general procedure was followed using L-phenylalaninamide hydrochloride (500 mg, 3.05 mmol), chloro[bis(dicyclohexylamino)]cyclopropenium chloride (1.30 g, 2.77 mmol) and *N*,*N*-diisopropylethylamine (0.80 mL, 6.33 mmol) in dichloromethane (28 mL). Recrystallization from ethyl acetate/hexanes/dichloromethane yielded the title product as a white solid (1.40 g, 2.51 mmol, 90% yield). ^{1}H NMR (400 MHz, $CDCl_3$) δ 9.5 (s, 1H, N**H**), 8.62 (d, J = 9.8 Hz, 1H, CpN**H**), 7.41 (d, J = 7.2 Hz, 2H, Ar**H**), 7.30-7.18 (m, 3H, Ar**H**), 5.53 (s, 1H, N**H**), 4.16 (m, 1 H, NC**H**$CONH_2$), 3.80 (dd, J = 11.2 and 13.8 Hz, 1H, C**H**Ph), 3.30 (m, 5H, C**H**Ph and NCy**H**), 1.89-1.30 (m, 40H, Cy**H**). ^{13}C NMR (100 MHz, $CDCl_3$) δ 174.0, 137.3, 129.3, 128.0, 126.4, 115.0, 114.6, 64.8, 59.2, 38.4, 31.9, 31.8, 25.3, 25.3, 24.3. IR (thin film, cm^{-1}) 3159, 2928, 2845, 1687, 1537, 1500, 1447, 1373, 1322, 1251, 1180, 895, 577. $[\alpha]^{20}_D$ = 2.3 (1.0 c, $CHCl_3$). HRMS (FAB+) m/z = 559.4360; calcd for $C_{36}H_{55}N_4O$ $[M]^+$ 559.4370.

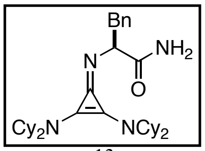

(*S*)-2-(2,3-Bis(dicyclohexylamino)cyclopropenimine)-3-phenylpropanamide (18). ^{1}H NMR (400 MHz, $CDCl_3$) δ 7.28 (m, 2H, Ar**H**), 7.16-7.10 (m, 3H, Ar**H**), 4.26 (dd, J = 4.4 and 6.1 Hz, 1H, Cp=NC**H**), 3.15-2.95 (m, 6H, NCy**H** and C**H₂**Ph), 1.85-1.05 (m, 40H, Cy**H**). ^{13}C NMR (100 MHz, $CDCl_3$) δ 179.4, 139.5, 130.4, 127.2, 126.6, 125.5, 114.7, 111.4, 66.2, 58.8, 57.7, 42.5, 33.3, 33.1, 32.4, 32.1, 26.3, 26.2, 26.1, 25.3.

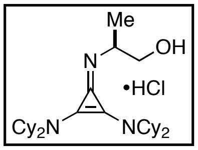

(*S*)-2-(2,3-Bis(dicyclohexylamino)cyclopropenimine)propan-1-ol hydrochloride (29•HCl). The general procedure was followed using (*S*)-2-amino-1-propanol (1.0 mL, 12.8 mmol), chloro[bis(dicyclohexylamino)]cyclopropenium chloride (5.45 g, 11.7 mmol) and *N*,*N*-diisopropylethylamine (3.3 mL, 23.3 mmol) in dichloromethane (117 mL). Recrystallization from ethyl acetate/hexanes yielded the title product as a white solid (4.50 g, 9.58 mmol, 82% yield). ^{1}H NMR (400 MHz, $CDCl_3$) δ 7.78 (d, J = 6.8 Hz, 1H, CpN**H**), 5.44 (s, 1H, O**H**), 3.85 (m, 3H, NC**HCH₂**OH), 3.35 (m, 4H, NCy**H**), 1.95-1.20 (m, 40H, Cy**H**), 1.26 (d, J = 6.2 Hz, 3H, C**H₃**). ^{13}C NMR (100 MHz, $CDCl_3$) δ 116.6, 115.2, 64.5, 59.7, 55.5, 32.4, 25.8, 25.7, 24.7, 19.1. IR (thin film, cm^{-1}) 2927, 2853, 1525, 1491, 1446, 1370, 1252, 1058, 1020, 990, 895, 577. $[\alpha]^{20}_{D}$ = -0.5 (1.0 c, $CHCl_3$). LRMS (APCI+) m/z = 310.36; calcd for $C_{18}H_{35}N_3O$ $[M]^+$ 310.28. HRMS (FAB+) m/z = 470.4121; calcd for $C_{30}H_{52}N_3O$ $[M]^+$ 470.4105.

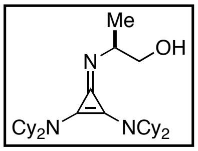

(*S*)-2-(2,3-Bis(dicyclohexylamino)cyclopropenimine)propan-1-ol (29). ^{1}H NMR (400 MHz, $CDCl_3$) δ 3.62-3.55 (m, 2H, C**H**OH and Cp=NC**H**), 3.30 (dd, J = 6.4 and 9.2 Hz, 1H, C**H**OH), 3.17 (m, 4H, NCy**H**), 1.90-1.10 (m, 40H, Cy**H**), 1.10 (d, J = 6.2 Hz, 3H, C**H₃**). ^{13}C NMR (100 MHz, $CDCl_3$) δ 113.7, 67.6, 58.4, 55.8, 32.9, 32.8, 26.3, 26.2, 25.3, 21.0.

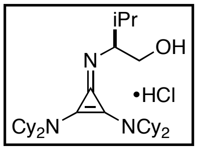

(*S*)-2-(2,3-Bis(dicyclohexylamino)cyclopropenimine)-3-methylbutan-1-ol hydrochloride (30•HCl). The general procedure was followed using (*S*)-2-amino-3-methyl-1-butanol (1.00 g, 9.69 mmol), chloro[bis(dicyclohexylamino)]cyclopropenium chloride (4.12 g, 8.81 mmol) and *N*,*N*-diisopropylethylamine (2.5 mL, 17.6 mmol) in dichloromethane (88 mL). Recrystallization from ethyl acetate/hexanes yielded the title product as a white solid (3.50 g, 7.03 mmol, 80% yield). ^{1}H NMR (400 MHz, $CDCl_3$) δ 7.55 (d, J = 9.0 Hz, 1H, CpN**H**), 5.21 (s, 1H, O**H**), 4.02 (m, 1H, NC**H**CH_2OH), 3.81 (m, 1H, C**H**OH), 3.45 (m, 1H, C**H**OH) 3.45 (m, 4H, NCy**H**), 2.09 (quintet, J = 6.9 Hz, 1H, C**H**Me_2), 1.94-1.20 (m, 40H, Cy**H**), 0.98 (d, J = 6.7 Hz, 6H, C**H₃**). ^{13}C NMR (100 MHz, $CDCl_3$) δ 117.3, 115.0, 65.1, 61.3, 59.4, 32.3, 32.2, 30.6, 25.6, 24.6, 19.5, 18.7. IR (thin film, cm^{-1}) 3226, 2930, 2855, 1501, 1450, 1378, 1177, 894, 731. $[\alpha]^{20}_{D}$ = -30.7 (1.0 c, $CHCl_3$). HRMS (FAB+) m/z = 498.4409; calcd for $C_{32}H_{56}N_3O$ $[M]^+$ 498.4418.

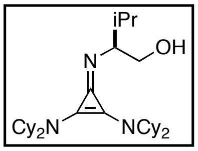

(*S*)-2-(2,3-Bis(dicyclohexylamino)cyclopropenimine)-3-methylbutan-1-ol (30). ^{1}H NMR (400 MHz, $CDCl_3$) δ 3.52 (m, 2H, C**H₂**OH), 3.30 (q, J = 5.8 Hz, 1H, Cp=NC**H**), 3.13 (m, 4H, NCy**H**), 1.85-1.10 (m, 41H, Cy**H** and C**H**Me_2), 0.91 (d, J = 6.4 Hz, 6H, 2 x C**H₃**). ^{13}C NMR (100

MHz, $CDCl_3$) δ 65.8, 63.0, 58.4, 32.9, 32.8, 32.7, 26.2, 25.3, 19.9, 18.8.

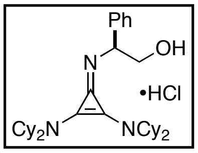

(*S*)-2-(2,3-Bis(dicyclohexylamino)cyclopropenimine)-2-phenylethanol hydrochloride (31•HCl). The general procedure was followed using (*S*)-2-phenylglycinol (500 mg, 3.64 mmol), chloro[bis(dicyclohexylamino)]cyclopropenium chloride (1.55 g, 3.31 mmol) and *N*,*N*-diisopropylethylamine (1. 0 mL, 6.62 mmol) in dichloromethane (33 mL). Recrystallization from ethyl acetate/hexanes/dichloromethane yielded the title product as a white solid (1.89 g, 3.08 mmol, 93% yield). ^{1}H NMR (400 MHz, $CDCl_3$) δ 8.04 (d, J = 5.8 Hz, 1H, CpN**H**), 7.36-7.25 (m, 5H, Ar**H**), 5.95 (t, J = 7.4 Hz, 1H, O**H**), 4.97 (m, 1H, C**H**Ph), 4.05 (m, 1H, C**H**OH), 3.93 (m, 1H, C**H**OH), 3.30 (m, 4H, NCy**H**), 1.95-1.20 (m, 40H, Cy**H**). ^{13}C NMR (100 MHz, $CDCl_3$) δ 139.4, 128.8, 127.7, 126.1, 116.5, 116.0, 65.8, 63.7, 59.6, 32.2, 32.2, 25.7, 25.6, 24.6. IR (thin film, cm^{-1}) 3168, 2928, 2858, 1500, 1447, 1375, 1068, 898, 726, 703. $[\alpha]^{20}_{D}$ = 6.3 (1.0 c, $CHCl_3$). HRMS (FAB+) m/z = 532.4248; calcd for $C_{35}H_{54}N_3O$ $[M]^+$ 532.4261.

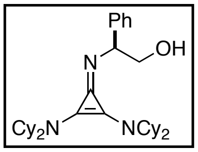

(*S*)-2-(2,3-Bis(dicyclohexylamino)cyclopropenimine)-2-phenylethanol (31). ^{1}H NMR (400 MHz, $CDCl_3$) δ 7.35 (d, J = 7.4 Hz, 2H, Ar**H**), 7.25 (m, 2H, Ar**H**), 7.13 (t, J = 7.3 Hz, 1H, Ar**H**), 4.69 (dd, J = 4.7 and 7.5 Hz, 1H, NC**H**Ph), 3.80 (dd, J = 4.6 and 9.3 Hz, 1H, C**H**OH), 3.39 (t, J = 8.0 Hz, 1H, C**H**OH), 3.07 (br s, 4H, NCy**H**), 1.90-0.95 (m, 40H, Cy**H**). ^{13}C NMR (100 MHz, $CDCl_3$) δ 145.7, 128.0, 127.0, 126.0, 68.7, 64.4, 58.1, 33.1, 32.7, 26.4, 26.2, 26.1, 25.3.

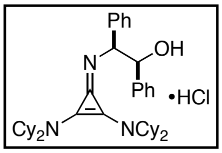

(1*S*,2*S*)-2-(2,3-Bis(dicyclohexylamino)cyclopropenimine)-1,2-diphenylethanol hydrochloride (32•HCl). The general procedure was followed using (*S,S*)-2-amino-1,2-diphenylethanol (500 mg, 2.34 mmol), chloro[bis(dicyclohexylamino)]cyclopropenium chloride (996 mg, 2.13 mmol) and *N*,*N*-diisopropylethylamine (0.60 mL, 4.26 mmol) in dichloromethane (21 mL). Recrystallization from ethyl acetate/hexanes yielded the title product as a white solid (1.33 g, 2.06 mmol, 97% yield). ^{1}H NMR (400 MHz, $CDCl_3$) δ 8.61 (d, J = 8.3 Hz, 1H, CpN**H**), 7.21-7.10 (m, 10H, Ar**H**), 6.26 (m, 1H, C**H**Ph), 5.31 (s, 1H, O**H**), 4.76 (t, J = 8.0 Hz, 1H, C**H**Ph), 3.31 (m, 4H, NCy**H**), 1.90-1.20 (m, 40H, Cy**H**). ^{13}C NMR (100 MHz, $CDCl_3$) δ 141.2, 139.7, 128.2, 127.7, 127.6, 127.5, 127.2, 127.1, 116.3, 115.6, 68.6, 59.5, 32.2, 62.1, 25.6, 25.5, 24.6. IR (thin film, cm^{-1}) 3152, 2927, 2855, 1499, 1447, 1372, 1179, 1056, 700. $[\alpha]^{20}_{D}$= -19.9 (1.0 c, $CHCl_3$). HRMS (FAB+) m/z = 608.4580; calcd for $C_{41}H_{58}N_3O$ $[M]^+$ 608.4574.

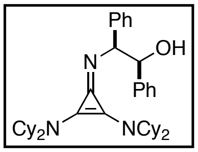

(1*S*,2*S*)-2-(2,3-Bis(dicyclohexylamino)cyclopropenimine)-1,2-diphenylethanol (32). ^{1}H NMR (400 MHz, $CDCl_3$) δ 7.30-7.12 (m, 10H, Ar**H**), 4.60 (d, J = 4.6 Hz, 1H, C**H**Ph), 4.50 (d, J = 4.4 Hz, 1H, C**H**Ar), 3.04 (br s, 4H, NCy**H**), 1.79-1.00 (m, 40H, Cy**H**). ^{13}C NMR (100 MHz, $CDCl_3$) δ 146.2, 144.8, 127.7, 127.6, 127.5, 126.9, 126.6, 126.2, 125.9, 79.2, 70.4, 58.1, 32.6, 26.2, 26.1, 25.3.

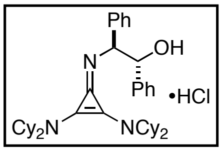

(1*R*,2*S*)-2-(2,3-Bis(dicyclohexylamino)cyclopropenimine)-1,2-diphenylethanol hydrochloride (33•HCl). The general procedure was followed using (1*R*,2*S*)-2-amino-1,2-diphenylethanol (500 mg, 2.34 mmol), chloro[bis(dicyclohexylamino)]cyclopropenium chloride (996 mg, 2.13 mmol) and *N*,*N*-diisopropylethylamine (0.60 mL, 4.26 mmol) in dichloromethane (21 mL). Recrystallization from ethyl acetate/hexanes yielded the title product as a white solid (1.23 g, 2.02 mmol, 95% yield). ^{1}H NMR (400 MHz, $CDCl_3$) δ 7.33 (d, J = 7.2 Hz, 1H, CpN**H**), 7.21-7.00 (m, 10H, Ar**H**), 6.69 (m, 1H, C**H**Ph), 5.68 (s, 1H, O**H**), 5.28 (dd, J = 2.8 and 7.4 Hz, 1H, C**H**Ph), 3.31 (m, 4H, NCy**H**), 1.90-1.20 (m, 40H, Cy**H**). ^{13}C NMR (100 MHz, $CDCl_3$) δ 139.9, 137.6, 127.9, 127.8, 127.6, 127.5, 127.2, 126.7, 115.5, 115.4, 74.5, 66.4, 59.4, 32.3, 25.6, 25.5, 24.7. IR (thin film, cm^{-1}) 3199, 2933, 2855, 1501, 1453, 1375, 1181, 890.1, 740.9. $[\alpha]^{20}_{D}$ = -38.2 (1.0 c, $CHCl_3$). HRMS (FAB+) m/z = 608.4579; calcd for $C_{41}H_{58}N_3O$ $[M]^+$ 608.4574.

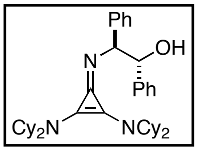

(1*R*,2*S*)-2-(2,3-Bis(dicyclohexylamino)cyclopropenimine)-1,2-diphenylethanol (33). ^{1}H NMR (400 MHz, $CDCl_3$) δ 7.10-6.95 (m, 10H, Ar**H**), 4.94 (m, 2H, 2 x C**H**Ph), 3.21 (br s, 2H, NCy**H**), 2.99 (br s, 2H, NCy**H**), 1.95-1.10 (m, 40H, Cy**H**). ^{13}C NMR (100 MHz, $CDCl_3$) δ 142.9, 142.0, 128.4, 127.1, 127.0, 126.9, 126.2, 126.0, 125.7, 78.1, 69.0, 58.2, 33.1, 32.7, 26.2, 26.1, 25.3.

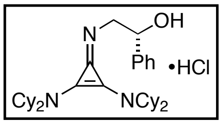

(*R*)-2-(2,3-Bis(dicyclohexylamino)cyclopropenimine)-1-phenylethanol hydrochloride (34•HCl). The general procedure was followed using (*R*)-2-amino-1-phenylethanol (301 mg, 2.20 mmol), chloro[bis(dicyclohexylamino)]cyclopropenium chloride (935 mg, 2.00 mmol) and *N*,*N*-diisopropylethylamine (0.68 mL, 4.00 mmol) in dichloromethane (20 mL). Recrystallization from ethyl acetate/hexanes yielded the title product as a white solid (957 mg, 1.80 mmol, 90% yield). ^{1}H NMR (400 MHz, $CDCl_3$) δ 8.17 (t, J = 5.4 Hz, 1H, CpN**H**), 7.44 (d, J = 7.4 Hz, 2H, Ar**H**), 7.29 (t, J = 7.3 Hz, 2H, Ar**H**), 7.22 (t, J = 7.2 Hz, 1H, Ar**H**), 5.88 (s, 1H, O**H**), 5.15 (d, J = 7.3 Hz, 1H, C**H**Ph), 3.84-3.73 (m, 2H, NC$\mathbf{H_2}$), 3.30 (m, 4H, NCy**H**), 1.90-1.20 (m, 40H, Cy**H**). ^{13}C NMR (100 MHz, $CDCl_3$) δ 142.1, 128.2, 127.4, 126.1, 116.6, 114.5, 72.1, 59.6, 54.3, 32.3, 25.7, 24.5. IR (thin film, cm^{-1}) 3141, 2930, 2855, 1528, 1491, 1447, 1372, 1099, 891. $[\alpha]^{20}_{D}$ = -9.7 (1.0 c, $CHCl_3$). HRMS (FAB+) m/z = 532.4261; calcd for $C_{35}H_{54}N_3O$ $[M]^+$ 532.4261.

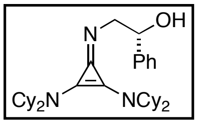

(*R*)-2-(2,3-Bis(dicyclohexylamino)cyclopropenimine)-1-phenylethanol (34). ^{1}H NMR (400 MHz, $CDCl_3$) δ 7.42 (d, J = 7.3 Hz, 2H, Ar**H**), 7.30 (t, J = 7.4 Hz, 2H, Ar**H**), 7.20 (t, J = 7.3 Hz, 1H, Ar**H**), 4.70 (dd, J = 4.0 and 8.8 Hz, 1H, C**H**PhOH), 3.85 (dd, J = 4.2 and 11.6 Hz, 1H, Cp=NC**H**), 3.35 (dd, J = 9.1 and 11.6 Hz, 1H, Cp=NC**H**), 3.12 (m, 4H, NCy**H**), 1.95-1.00 (m, 40H, Cy**H**). ^{13}C NMR (100 MHz, $CDCl_3$) δ 144.0, 128.0, 126.7, 126.0, 73.6, 59.6, 58.4, 32.7, 26.3, 25.3.

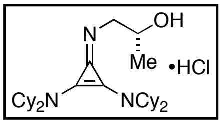

(*R*)-1-(2,3-Bis(dicyclohexylamino)cyclopropenimine)propan-2-ol hydrochloride (35•HCl). The general procedure was followed using (*R*)-1-amino-2-propanol (165 mg, 2.20 mmol), chloro[bis(dicyclohexylamino)]cyclopropenium chloride (935 mg, 2.00 mmol) and *N,N*-diisopropylethylamine (0.68 mL, 4.00 mmol) in dichloromethane (20 mL). Recrystallization from ethyl acetate/hexanes yielded the title product as a white solid (780 mg, 1.66 mmol, 83% yield). ^{1}H NMR (400 MHz, $CDCl_3$) δ 7.94 (s, 1H, CpN**H**), 5.26 (s, 1H, O**H**), 4.23 (m, 1H, C**H**Me), 3.50 (m, 2H, NC**H₂**), 3.31 (m, 4H, NCy**H**), 1.90-1.20 (m, 40H, Cy**H**), 1.23 (d, J = 4.6 Hz, 3H, C**H₃**). ^{13}C NMR (100 MHz, $CDCl_3$) δ 116.9, 114.5, 65.6, 59.6, 54.3, 32.4, 32.3, 25.8, 24.7, 20.8. IR (thin film, cm^{-1}) 3226, 2933, 2858, 1494, 1453, 1375, 1321, 1249, 1181, 894. $[\alpha]^{20}_D$ = -9.9 (1.0 c, $CHCl_3$). HRMS (FAB+) m/z = 470.4117; calcd for $C_{30}H_{52}N_3O$ $[M]^+$ 470.4105.

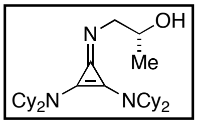

(*R*)-1-(2,3-Bis(dicyclohexylamino)cyclopropenimine)propan-2-ol (35). ^{1}H NMR (400 MHz, $CDCl_3$) δ 3.79 (m, 1H, C**H**OH), 3.59 (dd, J = 3.7 and 11.4 Hz, 1H, Cp=NC**H**), 3.13 (m, 5H, NCy**H** and Cp=NC**H**), 1.90-1.20 (m, 40H, Cy**H**), 1.17 (d, J = 6.1 Hz, 3H, C**H₃**). ^{13}C NMR (100 MHz, $CDCl_3$) δ 67.4, 58.8, 58.4, 32.8, 26.3, 25.4, 20.2.

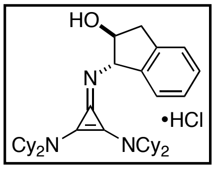

(1*S*,2*S*)-1-(2,3-Bis(dicyclohexylamino)cyclopropenimine)-2,3-dihydro-1*H*-inden-2-ol hydrochloride (36•HCl). The general procedure was followed using (1*S*,2*S*)-*trans*-1-amino-2-indanol (300 mg, 2.01 mmol), chloro[bis(dicyclohexylamino)]cyclopropenium chloride (855 mg, 1.83 mmol) and *N,N*-diisopropylethylamine (0.55 mL, 3.84 mmol) in dichloromethane (18 mL). Recrystallization from ethyl acetate/hexanes yielded the title product as a white solid (1.00 g, 1.72 mmol, 94% yield). ^{1}H NMR (400 MHz, $CDCl_3$) δ 7.72 (d, J = 5.9 Hz, 1H, CpN**H**), 7.21-7.10 (m, 3H, Ar**H**), 6.97 (d, J = 7.4 Hz, 1H, Ar**H**), 6.1 (s, 1H, O**H**), 5.00 (q, J = 9.1 Hz, 1H, C**H**OH), 4.91 (t, J = 6.2 Hz, 1H, C**H**NH), 3.37 (m, 4H, NCy**H**), 3.20 (dd, J = 7.3 and 15.2 Hz, 1H, C**H**Ar), 2.96 (dd, J = 9.9 and 15.0 Hz, 1H, C**H**Ar), 1.90-1.20 (m, 40H, Cy**H**). ^{13}C NMR (125 MHz, $CDCl_3$) δ 140.6, 139.2, 128.3, 126.8, 125.3, 122.4, 116.6, 115.1, 77.9, 68.5, 59.5, 37.1, 32.4, 32.2, 25.6, 25.5, 24.6. IR (thin film, cm^{-1}) 3209, 2926, 2855, 1498, 1447, 1375, 1177, 894, 744. $[\alpha]^{20}_D$ = 79.0 (1.0 c, $CHCl_3$). HRMS (FAB+) m/z = 544.4283; calcd for $C_{36}H_{54}N_3O$ $[M]^+$ 544.4261.

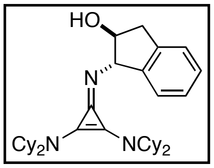

(1*S*,2*S*)-1-(2,3-Bis(dicyclohexylamino)cyclopropenimine)-2,3-dihydro-1*H*-inden-2-ol (36). ^{1}H NMR (400 MHz, $CDCl_3$) δ 7.13 (m, 4H, Ar**H**), 4.74 (d, J = 7.9 Hz, 1H, NC**H**Ar), 4.40 (broad q, J = 7.5 Hz, 1H, C**H**OH), 3.20 (m, 4H, NCy**H**), 3.11 (dd, J = 7.4 Hz, 1H, C**H**Ar), 2.85 (dd, J = 9.7 and 14.7 Hz, 1H, C**H**Ar), 1.90-1.00 (m, 40H, Cy**H**). ^{13}C NMR (100 MHz, $CDCl_3$) δ 138.9, 126.8, 126.5, 124.4, 124.0, 82.0, 73.1, 58.5, 37.3, 32.9, 26.3, 26.1, 25.2.

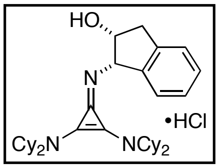

(1*S*,2*R*)-1-(2,3-Bis(dicyclohexylamino)cyclopropenimine)-2,3-dihydro-1*H*-inden-2-ol hydrochloride (37•HCl). The general procedure was followed using (1*S*,2*R*)-*cis*-1-amino-2-indanol (300 mg, 2.01 mmol), chloro[bis(dicyclohexylamino)]cyclopropenium chloride (855 mg, 1.83 mmol) and *N*,*N*-diisopropylethylamine (0.55 mL, 3.84 mmol) in dichloromethane (18 mL). Recrystallization from ethyl acetate/hexanes yielded the title product as a white solid (1.04 g, 1.79 mmol, 98% yield). ^{1}H NMR (400 MHz, $CDCl_3$) δ 7.28-7.15 (m, 4H, Ar**H**), 6.80 (br s, 1H, O**H**), 6.55 (d, J = 8.4 Hz, 1H, CpN**H**), 5.05 (m, 2H, C**H**OH and C**H**NH), 3.35 (m, 4H, NCy**H**), 3.20-3.14 (m, 2H, C**H₂**Ar), 1.90-1.20 (m, 40H, Cy**H**). ^{13}C NMR (100 MHz, $CDCl_3$) δ 141.4, 140.4, 128.6, 126.7, 125.9, 123.6, 115.8, 114.6, 71.7, 63.6, 59.4, 40.0, 32.6, 32.5, 26.1, 25.7, 25.6, 24.8. IR (thin film, cm^{-1}) 3155, 2930, 2858, 1501, 1450, 1372, 1181, 894, 734. $[\alpha]^{20}_D$ = 85.0 (1.0 c, $CHCl_3$). HRMS (FAB+) m/z = 544.4246; calcd for $C_{36}H_{54}N_3O$ $[M]^+$ 544.4261.

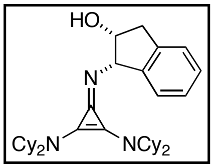

(1*S*,2*R*)-1-(2,3-Bis(dicyclohexylamino)cyclopropenimine)-2,3-dihydro-1*H*-inden-2-ol (37). ^{1}H NMR (400 MHz, $CDCl_3$) δ 7.15 (m, 4H, Ar**H**), 5.00 (d, J = 5.0 Hz, 1H, NC**H**Ar), 4.40 (broad t, J = 3.6 Hz, 1H, C**H**OH), 3.20 (m, 4H, NCy**H**), 3.03 (m, 2H, C**H₂**Ar), 1.90-1.00 (m, 40H, Cy**H**). ^{13}C NMR (100 MHz, $CDCl_3$) δ 147.0, 141.3, 126.7, 126.2, 125.0, 73.6, 67.9, 58.5, 39.5, 33.2, 32.9, 26.4, 26.3, 26.2, 25.3.

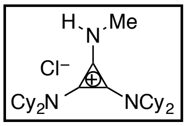

***N*-Methyl-(2,3-bis(dicyclohexylamino)cyclopropenimine hydrochloride (26•HCl).** The general procedure was followed using methylamine (1.1 mL 2.0M in THF, 2.14 mmol), chloro[bis(dicyclohexylamino)]cyclopropenium chloride (1.00 g, 2.14 mmol) and *N*,*N*-diisopropylethylamine (1.12 mL, 6.42 mmol) in dichloromethane (21 mL). Recrystallization from ethyl acetate/hexanes yielded the title product as a white solid (892 mg, 1.93 mmol, 90% yield). ^{1}H NMR (400 MHz, CD_3CN) δ 7.11 (s, 1H, N**H**), 3.36 (tt, J = 3.9 and 12.1 Hz, 4H, NCy**H**), 3.12 (d, J = 4.8 Hz, 3H, NC**H₃**), 1.95-1.20 (m, 40H, Cy**H**). ^{13}C NMR (125 MHz, CD_3CN) δ 117.7, 115.6, 60.1, 33.1, 32.7, 26.5, 25.3. IR (thin film, cm^{-1}) 2925, 2854, 1500, 1447, 1371, 1251, 1182, 1078, 894. HRMS (FAB+) m/z = 426.3839; calcd for $C_{28}H_{48}N_3$ $[M]^+$ 426.3843.

Synthesis of bis(diisopropylamino)-based cyclopropenimines.

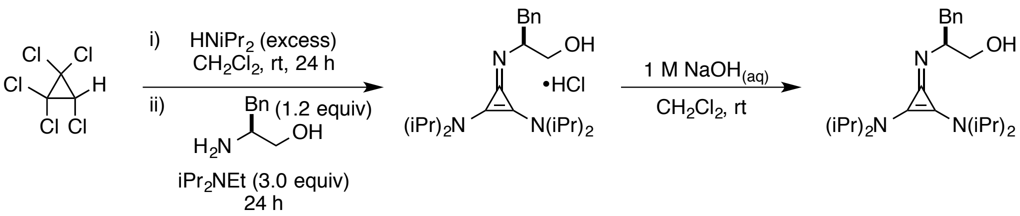

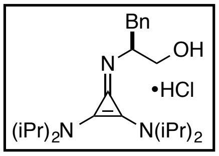

(*S*)-2-(2,3-Bis(diisopropylamino)cyclopropenimine)-3-phenylpropan-1-ol hydrochloride (23•HCl). Diisopropylamine (3.93 mL, 27.9 mmol, 6.0 equiv) was slowly added to a solution of pentachlorocyclopropane[3] (0.57 mL, 4.65 mmol, 1.0 equiv) in CH_2Cl_2 (46 mL, 0.1 M) in a 250 mL round bottom flask. The reaction mixture was stirred for a further 24 hr at room temperature. Next, *N*,*N*-diisopropylethylamine (2.40 mL, 14.1 mmol, 3.0 equiv) and (*S*)-2-amino-3-phenylpropan-1-ol[4] (854 mg, 5.65 mmol, 1.2 equiv) was added in one portion and the reaction mixture was stirred for an additional 24 hr. The crude reaction mixture was then washed with 1.0 M HCl (3 x 100 mL), dried with anhydrous sodium sulfate and concentrated *in vacuo* to yield crude cyclopropenimine hydrochloride salt as an off-white solid. Recrystallization from ethyl acetate/hexanes (approximately 2/1) yielded pure cyclopropenimine hydrochloride salt as a white solid (1.73 g, 4.10 mmol, 88% yield). ^{1}H NMR (400 MHz, $CDCl_3$) δ 7.10-7.25 (m, 5H, Ar**H**), 6.30 (d, 9.6 Hz, N**H**), 3.60-4.0 (m, 8H, NC**H**BnC**H₂**O**H**, NC**H**(CH_3)$_2$), 1.20 (m, 24H, NCH(C**H₃**)$_2$); ^{13}C NMR (100 MHz, $CDCl_3$) δ 137.9, 129.5, 128.5, 126.6, 116.3, 113.7, 64.6, 62.4, 50.5, 38.1, 22.1, 22.0. IR (thin film, cm^{-1}) 3257, 3124, 2971, 2930, 1501, 1457, 1344, 1211, 1136, 1034, 700. $[\alpha]^{20}_D$ = -42.5 (1.0 c, $CHCl_3$). LRMS (APCI+) m/z = 386.32 calcd for $C_{24}H_{40}N_3O$ $[M+]^+$, found 386.31.

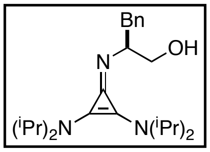

(*S*)-2-(2,3-Bis(diisopropylamino)cyclopropenimine)-3-phenylpropan-1-ol (23). Pure cyclopropenimine freebase was quantitatively obtained by dissolving the corresponding hydrochloride salt in CH_2Cl_2 and washing the solution with 1.0 M NaOH (3 x), drying with anhydrous sodium sulfate and concentrating *in vacuo*. The cyclopropenimine is obtained as an off-white solid. The isolated cyclopropenimine was used immediately after deprotonation. Note: identical spectra were obtained when deprotonated with sodium hydride in acetonitrile. ^{1}H NMR (400 MHz, CD_3CN) δ 7.10-7.30 (m, 5H, Ar**H**), 3.60-3.75 (m, 5H, NC**H**(CH_3)$_2$, NC**H**BnCH$_2$OH), 3.20-3.40 (m, 2H, NCHBnC**H₂**OH), 2.60-2.80 (m, 2H, NCHC**H₂**Ph), 1.20 (d, 6.8 Hz, 24H, NCH(C**H₃**)$_2$); ^{13}C NMR (100 MHz, CD_3CN) δ 141.5, 130.5, 128.8, 128.3, 126.5, 66.6, 64.6, 50.1, 42.3, 22.6, 22.5; $[\alpha]^{20}_D$ = -10.1 (2.0 c, $CHCl_3$).

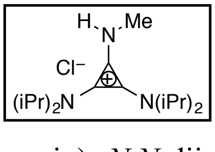

***N*-Methyl-(2,3-Bis(diisopropylamino)cyclopropenimine) (25•HCl).** This cyclopropenimine was prepared in an analogous fashion as directly above using pentachlorocyclopropane (347 mg, 1.62 mmol, 1.0 equiv), *N*,*N*-diisopropylamine (1.36 mL, 9.72 mmol, 6.0 equiv) and methylamine (2.44 mL of 2.0M THF solution, 4.88 mmol, 3.00 equiv). Silica gel column chromotragaphy (20:1 DCM:MeOH) followed by recrystallization from ethyl acetate/hexanes yielded the titled product as an off-white solid (180 mg, 0.596 mmol, 37% yield). ^{1}H NMR (400 MHz, CD_3CN) δ 8.37 (s, 1H, N**H**), 3.83 (quintet, J = 6.8 Hz, 4H, NC**H**Me$_2$), 3.06 (d, J = 4.8 Hz, 3H, NC**H₃**), 1.28 (d, J = 6.8 Hz, 24H, NCH(C**H₃**)$_2$). ^{13}C NMR (100 MHz, CD_3CN) δ 118.1, 114.9, 51.3, 32.7, 22.1. IR (thin film, cm^{-1}) 2929, 2858, 1504, 1451,

---

[3] Tobey, S. W.; West, R. *J. Am. Chem. Soc.* **1966**, *88*, 2478.
[4] Shi, L.; Chen, L.; Chen, R.; Chen, L. *J. Label Compd. Radiopharm.* **2010**, *53*, 147-151.

1370, 1344, 1194, 1155, 1134, 1032, 895. HRMS (FAB+) m/z = 266.2603; calcd for $C_{16}H_{32}N_3$ $[M]^+$ 266.2591.

Synthesis of bis(pyrrolidinol)-based cyclopropenimine.

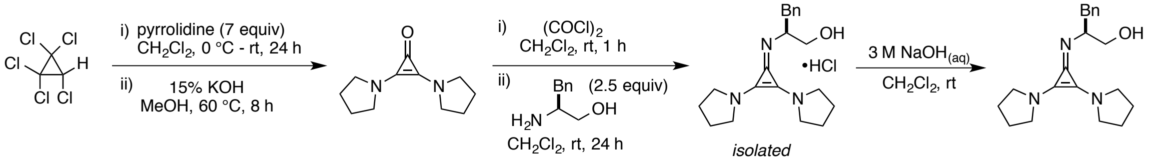

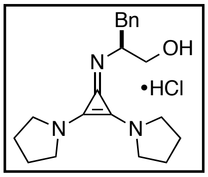

(*S*)-2-(2,3-Bis(pyrrolidino)cyclopropenimine)-3-phenylpropan-1-ol hydrochloride (24•HCl). Pyrrolidine (13.4 mL, 163.3 mmol, 7.0 equiv) was slowly added to a 0 °C solution of pentachlorocyclopropane (5 g, 23.3 mmol, 1.0 equiv) in dichloromethane (230 mL, 0.1 M). After addition the reaction solution was allowed to warm to room temperature and was stirred overnight before direct concentration by rotary evaporator to yield a crude mixture of tris(pyrrolidino)cyclopropenium chloride and pyrrolidine hydrochloride salts.

The crude solid from above was subjected to hydrolysis by dissolution in methanol (90 mL) and mixing with 15% KOH (13.5 g KOH in 90 mL H_2O). The reaction mixture was stirred at 60 °C for 8 h before extraction with dichloromethane (3 x 100 mL). The extracted organics were dried with sodium sulfate and concentrated *in vacuo* to yield 2,3-di(pyrrolidin-1-yl)cycloprop-2-en-1-one. A crude ^{1}H NMR of this material is provided below. This material was not further purified.

Oxallyl chloride (0.27 mL, 3.12 mmol, 1.5 equiv) was added dropwise to a solution of 2,3-di(pyrrolidin-1-yl)cycloprop-2-en-1-one (400 mg, 2.08 mmol, 1.0 equiv) in dichloromethane (21 mL, 1.0 M). After stirring for 1 hr at room temperature the reaction solution was concentrated *in vacuo* and further dried on a vacuum pump for an addition 2 hr. The obtained crude solid was then redissolved in dichloromethane (21 mL, 1.0 M) and (*S*)-2-amino-3-phenylpropan-1-ol (786 mg, 5.20 mmol, 2.5 equiv) was added in one portion. After stirring at room temperature overnight the reaction solution was washed with 1 M HCl (3 x 10 mL), dried with sodium sulfate and concentrated *in vacuo* to a crude solid. Purification by silica gel chromotagraphy (5-10% MeOH/DCM eluent) yielded the title hydrochloride salt as a tan solid (254 mg, 0.702 mmol, 34% yield from cyclopropenone). ^{1}H NMR (500 MHz, $CDCl_3$) δ 8.90 (d, J = 8.5 Hz, 1H, CpN**H**), 7.35 (m, 4H, Ar**H**), 7.19 (t, J = 7.1 Hz, 1H, Ar**H**), 4.86 (s, 1H, O**H**), 3.72 (dd, J = 3.2 and 11.6 Hz, 1H, C**H**OH), 3.56 (m, 1H, C**H**OH), 3.49 (m, 1H, CpNC**H**Bn), 3.45 (br s, 8H, CpNC**H₂** x 4), 2.93 (dd, J = 5.6 and 13.6 Hz, 1H, C**H**Ph), 2.87 (dd, J = 8.7 and 13.6 Hz, 1H, C**H**Ph), 1.91 (quintet, J = 3.5 Hz, -C**H₂**- x 4). ^{13}C NMR (125 MHz, CD_3CN) δ 139.8, 130.6, 129.3, 127.2, 115.7, 114.0, 64.8, 63.4, 51.3, 38.6, 26.3. IR (thin film, cm^{-1}) 3134,

2926, 2871, 1505, 1478, 1453, 1345, 1178, 1047, 744, 702. $[\alpha]^{20}_{D}$ = -28.3 (1.0 c, $CHCl_3$). HRMS (FAB+) m/z = 326.2221; calcd for $C_{20}H_{28}N_3O$ $[M]^+$ 326.2227.

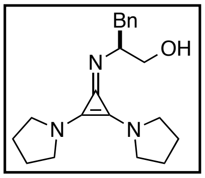

(*S*)-2-(2,3-Bis(pyrrolidino)cyclopropenimine)-3-phenylpropan-1-ol (24). Neutral cyclopropenimine was obtained by dissolving the corresponding hydrochloride salt in CH_2Cl_2 and washing the solution with 3.0 M NaOH (3 x), drying with anhydrous sodium sulfate and concentrating *in vacuo*. The cyclopropenimine is obtained as a brown solid. Note: treatment with sodium hydride or potassium *tert*-butoxide in acetonitrile did not appear to achieve deprotonation (the material obtained from these deprotonation attempts did not have any catalytic activity for the glycinate imine Michael reaction). ^1H NMR (300 MHz, $CDCl_3$) δ 7.40-7.10 (m, 5H, Ar**H**), 3.50-3.40 (m, 2H, C**H₂**OH), 3.28 (br s, 9H, 4 x NC**H₂** and NC**H**Bn), 2.68 (m, 2H, C**H₂**Ph), 1.85 (m, 8H, 4 x –C**H₂**-). ^{13}C NMR (100 MHz, CD_3CN) δ 141.1, 130.5, 129.0, 126.7, 113.3, 65.8, 65.4, 51.1, 40.0, 26.3.

General procedure for catalyst structure-activity relationship study (Figure 3, Equation 6 and Table 3).

Freshly deprotonated cyclopropenimine (0.0169 mmol, 0.1 equiv) and *tert*-butyl glycinate benzophenone Schiff base (50 mg, 0.169 mmol, 1.0 equiv) were dissolved in ethyl acetate (0.48 mL, 0.35 M). Methyl acrylate (46.0 μL, 0.508 mmol, 3.0 equiv) was then added to the vial and the reaction solution was stirred at room temperature. The reaction was monitored by ^1H NMR spectroscopy and conversion was determined by comparing *tert*-butyl integrals of the starting material and product. Upon 95% conversion, or after 24 hr if not complete, the reaction solution was concentrated and the crude material subjected to silica gel column chromatography (1/5 Et_2O/Hexanes) to yield pure product. Enantioselectivities were determined via chiral HPLC: Chiralpak AD-H (Hex/IPA = 97/3, 1.0 mL/min, 254 nm, 23°C), 6.1 min (minor), 6.7 min (major).

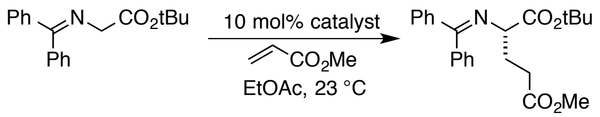

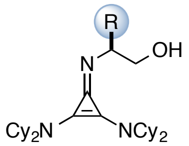

catalyst	R	t (h)	conv. (%)	ee (%)
**1**	Bn	1	95	98
**29**	Ph	24	70	86
**30**	iPr	4	95	95
**31**	Me	4	95	97

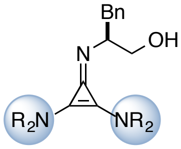

catalyst	R	t (h)	conv. (%)	ee (%)
**23**	$NiPr_2$	24	80	88
**24**	pyrrolidino	24	18	65

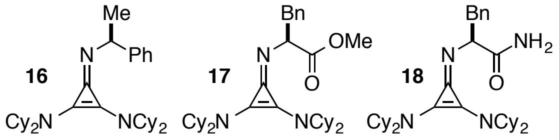

16: 24 h, 11% con, 0% ee
17: 48 h, 0% con
18: 24 h, 13% con, 89% ee

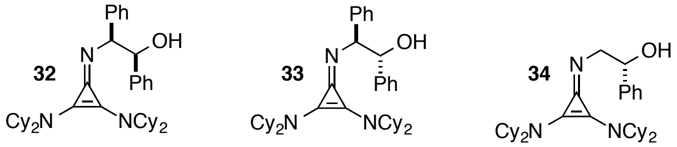

32: 24 h, 26% con, 77% ee
33: 24 h, 55% con, 22% ee
34: 24 h, 90% con, -73% ee

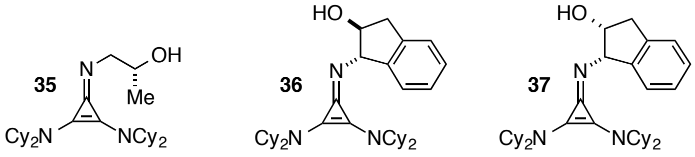

35: 24 h, 95% con, -63% ee
36: 2 h, 95% con, 98% ee
37: 24 h, 15% con, 75% ee

The initial reaction profiles of the above Michael reactions catalyzed by cyclopropenimines **1** and **23**, monitored by crude ^{1}H NMR spectroscopy, are provided in the chart below.

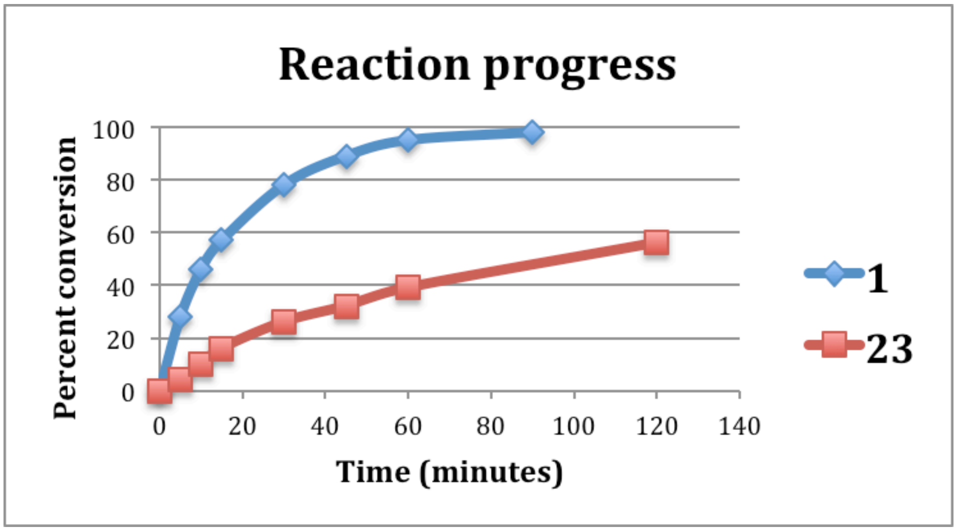

pKa measurements.

Note: Our previous method for determining cyclopropenimine hydrochloride salt pK_a relied on knowing the chemical shifts for both the cyclopropenimine hydrochloride and cyclopropenimine freebase. We recently found that depending on the base used to deprotonate cyclopropenimine hydrochloride salts (e.g. NaH, KOtBu, KH or NaOH) that the observed chemical shifts were rarely consistent. Thus, there was uncertainty associated with the identifying a chemical shift that accurately corresponded to the cyclopropenimine freebase. We therefore base our calculations below on the observed chemical shifts for the reference phosphazene base tBu-P_1(pyrr).

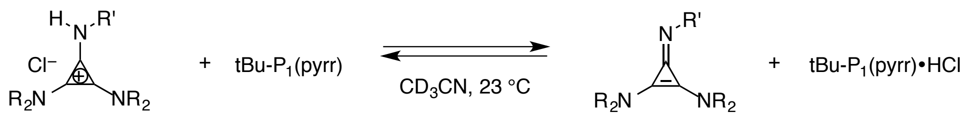

CpIm•HCl **Base** **CpIm** **Base•HCl**

$$K = \frac{[\text{Base•HCl}][\text{CpIm}]}{[\text{Base}][\text{CpIm•HCl}]}$$

$$pK_a(\text{CpIm•HCl}) = pK_a(\text{Base•HCl}) - \log(K)$$

Chemical shifts used:

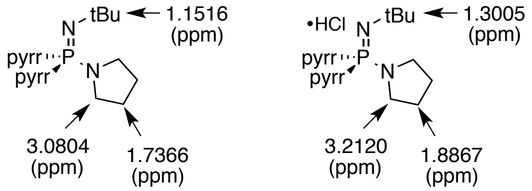

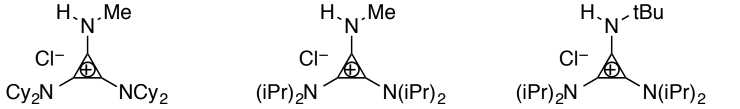

26•HCl **25•HCl** **27•HCl**

	26•HCl	25•HCl	27•HCl
**Representative observed chemical shifts (ppm) for tBu-P₁(pyrr):**	3.196, 1.2713	3.1488, 1.8002	3.1763, 1.8528, 1.2635

Calculated equilibrium constant (K) and pK_a of CpIm•HCl:

Trial	K	pK_a	Trial	K	pK_a	Trial	K	pK_a
1	22.2	27.1	1	0.79	28.5	1	9.0	27.5
2	25.6	27.0	2	1.76	28.2	2	7.3	27.6
3	24.8	27.0	3	0.64	28.6	3	10.0	27.4
Avg	24.2	27.0	Avg	1.06	28.4	Avg	8.8	27.5

General procedure. Cyclopropenimine hydrochloride salts were recrystallized before use. Phosphazene base P_1-t-Bu-tris(tetramethylene) (tBu-P_1(pyrr)) was used from a freshly opened commercial source (Sigma Aldrich Co.). Reference chemical shifts for both the freebase and hydrochloride salts of tBu-P_1(pyrr) were recorded in triplicate; the average chemical shifts are shown above. Stock solutions of cyclopropenimine hydrochloride salts and tBu-P_1(pyrr) in CD_3CN were prepared to insure accurate stoichiometric ratios.

A CD_3CN (0.6 mL) solution containing cyclopropenimine hydrochloride (0.03 mmol, 0.05 M, 1.0 equiv) and tBu-P_1(pyrr) (0.03 mmol, 0.05 M, 1.0 equiv) was prepared and allowed to mix for 15 minutes before obtaining an ^{1}H NMR spectrum (this spectrum did not change over the course of 4 hours). Using the observed chemical shifts of the tBu-P_1(pyrr), the ratio of tBu-P_1(pyrr) to tBu-P_1(pyrr)•HCl, and thus the ratio of CpIm•HCl to CpIm, could be determined. Representative observed chemical shifts of tBu-P_1(pyrr) for these experiments are listed above. Using the equations shown above, with the known pK_{BH+} of tBu-P_1(pyrr) (28.41 in MeCN),[5] an estimated pK_a for the cyclopropenimine hydrochloride salt was determined. Each experiment was ran in triplicate and the

[5] Kaljurand, I.; Ku□tt, A.; Sooväli, L.; Rodima, T.; Mäemets, V.; Leito, I.; Koppel, I. A. *J. Org.Chem.* **2005**, *70*, 1019.

calculated K and pK_a values are shown above. Example ^1H NMR spectra are provided below.

Solvent effect on Glycinate-Michael Additions (Table 1).

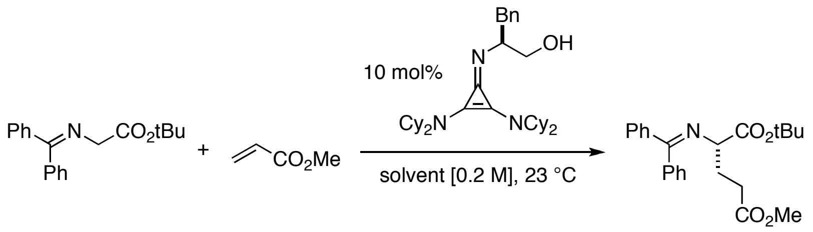

entry	solvent	ε	time (h)	yield (%)	ee (%)
1	1,4-dioxane	2.3	8	95	98
2	PhMe	2.4	5	95	99
3	NEt_3	2.4	6	95	99
4	Et_2O	4.3	2	95	98
5	EtOAc	6.0	2	95	98
6	THF	7.5	24	86	89
7	CH_2Cl_2	9.1	10	95	86
8	1,2-$F_2C_6H_4$	14.3	4	95	89
9	nBuOH	17.5	24	45	55
10	acetone	21	1.5	95	80

Cyclopropenimine (9.2 mg, 0.0169 mmol, 0.1 equiv) and *tert*-butyl glycinate benzophenone Schiff base (50 mg, 0.169 mmol, 1.0 equiv) were dissolved in solvent (0.85 mL, 0.20 M). Methyl acrylate (46 μL, 0.508 mmol, 3.0 equiv) was then added to the vial and the reaction solution was stirred at room temperature. The reaction was monitored by ^1H NMR spectroscopy and conversion was determined by comparing *tert*-butyl integrals of the starting material and product. Upon 95% conversion, or after 24 hr if not complete, the reaction solution was concentrated and the crude material subjected to silica gel column chromatography (1/5 Et_2O/Hexanes) to yield pure product. Enantioselectivities were determined via chiral HPLC: Chiralpak AD-H (Hex/IPA = 97/3, 1.0 mL/min, 254 nm, 23°C), 6.1 min (minor), 6.7 min (major).

General Procedure for Glycinate-Michael Additions with Unsubstituted Acceptors (Table 2).

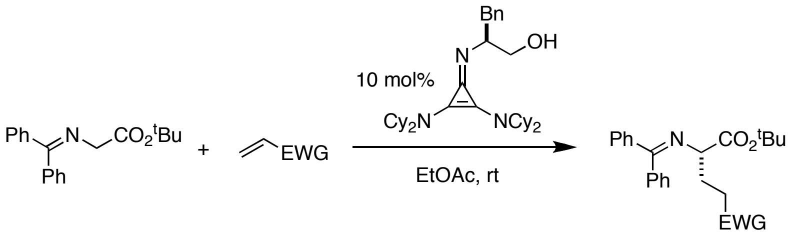

Cyclopropenimine **1** (3.6 mg, 0.00677 mmol, 0.1 equiv) and *tert*-butyl glycinate benzophenone Schiff base (20 mg, 0.0677 mmol, 1.0 equiv) were dissolved in ethyl

acetate (0.2 mL). Michael-acceptor (0.203 mmol, 3.0 equiv) was then added to the vial and the reaction solution was stirred at room temperature. Upon complete consumption of starting material, monitored by ^{1}H NMR spectroscopy, the reaction solution was concentrated and the crude material subjected to silica gel column chromatography (1/4 Et_2O/Hexanes eluent).

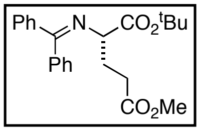

(*S*)-1-*tert*-Butyl-5-methyl 2-(diphenylmethyleneamino)pentanedioate (12a). [6] Isolated as a colorless oil (26 mg, 100% yield). ^{1}H NMR (400 MHz, $CDCl_3$) δ 7.64 (d, 7.2 Hz, 2H, Ar**H**), 7.14-7.45 (m, 8H, Ar**H**), 3.97 (t, 6.4 Hz, **H**$CCO_2{}^tBu$), 3.59 (s, 3H, $CO_2C\mathbf{H_3}$), 2.35-2.45 (m, 2H, -C$\mathbf{H_2}$-), 2.15-2.25 (m, 2H, -C$\mathbf{H_2}$-), 1.44 (s, 9H, C(C$\mathbf{H_3}$)$_3$); ^{13}C NMR (100 MHz, $CDCl_3$) δ 173.7, 170.9, 170.8, 139.6, 136.6, 130.4, 128.9, 128.7, 128.6, 128.1, 127.9, 81.3, 64.9, 51.6, 30.6, 28.8, 28.2. HPLC analysis: Chiralpak AD-H (Hex/IPA = 97/3, 1.0 mL/min, 254 nm, 23°C), 6.1 min (minor), 6.7 min (major), 98% ee.

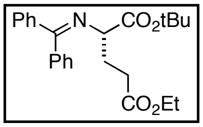

(*S*)-1-*tert*-Butyl 5-ethyl 2-(diphenylmethyleneamino)pentanedioate (Table 2, entry 1).[6] Reaction performed on a 0.169 mmol scale of *tert*-butyl glycinate benzophenone Schiff base. The title product was isolated as a colorless oil (64 mg, 0.162 mmol, 96% yield). ^{1}H NMR (400 MHz, $CDCl_3$) δ 7.64 (d, J = 7.1 Hz, 2H, Ar**H**), 7.48-7.30 (m, 6H, Ar**H**), 7.19 (dd, J = 3.0 and 4.3 Hz, 2H, Ar**H**), 4.05 (t, J = 7.1 Hz, 2H, $CO_2C\mathbf{H_2}CH_3$), 3.96 (t, J = 6.0 Hz, 1H, **H**$CCO_2{}^tBu$), 2.35 (m, 2H, C$\mathbf{H_2}$), 2.22 (m, 2H, C$\mathbf{H_2}$), 1.44 (s, 9H, C(C$\mathbf{H_3}$)$_3$), 1.19 (t, J = 7.1 Hz, 3H, $CO_2CH_2C\mathbf{H_3}$). ^{13}C NMR (100 MHz, $CDCl_3$) δ 173.3, 170.9, 170.7, 139.6, 136.6, 130.4, 128.9, 128.7, 128.6, 128.1, 127.9, 81.3, 65.0, 60.4, 30.9, 28.8, 28.2, 14.3. HPLC analysis: Chiralpak AD-H (Hex/IPA = 97/3, 1.0 mL/min, 254 nm, 23°C), 5.2 min (minor), 5.6 min (major), 99% ee.

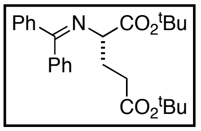

(*S*)-di-*tert*-Butyl 2-(diphenylmethyleneamino)pentanedioate[9] (Table 2, entry 2): Isolated as a colorless oil (28 mg, 98% yield). ^{1}H NMR (400 MHz, $CDCl_3$) δ 7.65 (d, 7.2 Hz, Ar**H**), 7.30-7.50 (m, 6H, Ar**H**), 7.20 (m, 2H, Ar**H**), 3.95 (t, 5.6Hz, 1H, **H**$CCO_2{}^tBu$), 2.15-2.30 (m, 4H, -C$\mathbf{H_2}$C$\mathbf{H_2}$-), 1.44 (s, 9H, C(C$\mathbf{H_3}$)$_3$), 1.39 (s, 9H, C(C$\mathbf{H_3}$)$_3$); ^{13}C NMR (100 MHz, $CDCl_3$) δ 172.6, 171.0, 170.7, 139.7 136.7, 130.4, 128.9, 128.7, 128.6, 128.1, 128.0, 81.2, 80.3, 65.1, 32.1, 29.0, 28.2. HPLC analysis: Chiralpak AD-H (Hex/IPA = 98/2, 0.6 mL/min, 254 nm, 23°C), 9.0 min (minor), 9.6 min (major), 99% ee.

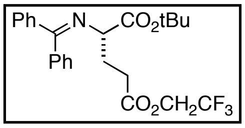

(*S*)-1-*tert*-Butyl 5-(2,2,2-trifluoroethyl) 2-(diphenylmethyleneamino)pentanedioate (Table 2, entry 3).

---

[6] Ma, T.; Fu, X.; Kee, W. K.; Zong, L.; Pan, Y.; Huang, K.W.; Tan, C.H. *J. Am. Chem. Soc.* **2011**, *133*, 2828-2831.

Reaction performed on a 0.169 mmol scale of *tert*-butyl glycinate benzophenone Schiff base. The title product was isolated as a colorless oil (68 mg, 0.151 mmol, 90% yield). ^{1}H NMR (400 MHz, $CDCl_3$) δ 7.62 (d, J = 7.1 Hz, 2H, Ar**H**), 7.45-7.25 (m, 6H, Ar**H**), 7.17 (m, 2H, Ar**H**), 4.34 (m, 2H, $CO_2C\mathbf{H_2}CF_3$), 3.99 (dd, J = 5.0 and 7.3 Hz, 1H, C**H**CO_2R), 2.49 (m, 2H, -C$\mathbf{H_2}$-), 2.24 (m, 2H, -C$\mathbf{H_2}$-), 1.44 (s, 9H, C(C$\mathbf{H_3}$)$_3$). ^{13}C NMR (125 MHz, $CDCl_3$) δ 171.7, 171.0, 170.6, 139.5, 136.5, 130.6, 128.9, 128.8, 128.2, 127.9, 81.5, 64.7, 60.4 (q), 30.2, 28.5, 28.2. ^{19}F NMR (470 MHz, $CDCl_3$) δ 72.8 ppm (s, 3F). IR (thin film, cm^{-1}) 2977, 2936, 1757, 1733, 1627, 1368, 1280, 1143, 973, 693. $[\alpha]^{20}_{D}$ = -40.0 (1.0 c, $CHCl_3$). HRMS (FAB+) m/z = 450.1883; calcd for $C_{24}H_{26}F_3NO_4$ $[M+1]^+$ 450.1814. HPLC analysis: Chiralpak OD-H (Hex/IPA = 98.5/1.5, 1.0 mL/min, 254 nm, 23°C), 5.9 min (major), 7.2 min (minor), 58% ee.

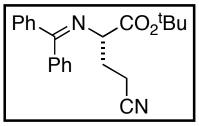

(*S*)-*tert*-Butyl 4-cyano-2-(diphenylmethyleneamino)butanoate [7] **(Table 2, entry 4):** Obtained as a pale yellow oil (23 mg, 98% yield). ^{1}H NMR (400 MHz, $CDCl_3$) δ 7.66 (m, 2H, Ar**H**), 7.30-7.50 (m, 6H, Ar**H**), 7.20 (m, 2H, Ar**H**), 4.05 (m, 1H, **H**C$CO_2{}^t$Bu), 2.20-2.50 (m, 4H, -C$\mathbf{H_2}$C$\mathbf{H_2}$-), 1.44 (s, 9H, C(C$\mathbf{H_3}$)$_3$); ^{13}C NMR (100 MHz, $CDCl_3$) δ 172.1, 170.0, 139.2, 136.1, 130.7, 129.0, 128.9, 128.7, 128.2, 127.5, 119.5, 81.9, 63.8, 29.5, 28.1, 13.9. HPLC analysis: Chiralpak AD-H (Hex/IPA = 98/2, 1.0 mL/min, 254 nm, 23°C), 9.3 min (minor), 11.7 min (major), 77% ee.

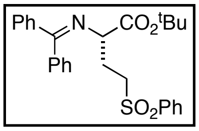

(*S*)-*tert*-Butyl 2-(diphenylmethyleneamino)-4-(phenylsulfonyl)butanoate[8] **(Table 2, entry 5):** Isolated with phenyl vinyl sulfone starting material (88% yield based on benzyl ether standard). ^{1}H NMR (400 MHz, $CDCl_3$) δ 7.90 (m, 2H, Ar**H**), 7.50-7.70 (m, 5H, Ar**H**), 7.30-7.45 (m, 6H, Ar**H**), 7.10 (m, 2H, Ar**H**), 4.00 (m, 1H, **H**C$CO_2{}^t$Bu), 3.10-3.35 (m, 2H, C$\mathbf{H_2}SO_2$Ph), 2.10-2.30 (m, 2H, C$\mathbf{H_2}CH_2SO_2$Ph), 1.37 (s, 9H, C(C$\mathbf{H_3}$)$_3$); ^{13}C NMR (100 MHz, $CDCl_3$) δ 171.5, 169.9, 139.1, 139.0, 136.1, 130.7, 129.4, 128.9, 128.9, 128.7, 128.2, 128.1, 127.7, 81.8, 63.6, 52.9, 28.0, 27.1. HPLC analysis: Chiralpak AD-H (Hex/IPA = 95/5, 1.0 mL/min, 254 nm, 23°C), 12.9 min (minor), 16.8 min (major), 41% ee.

General Procedure for Glycinate-Michael Additions with Substituted Acceptors (Table 3).

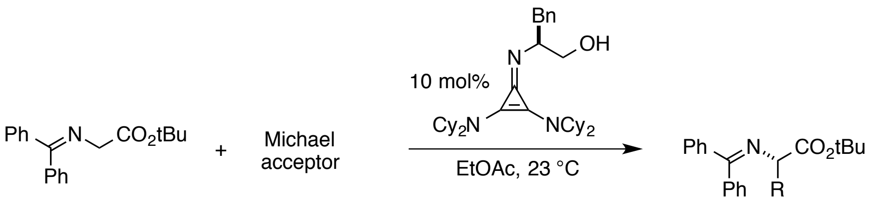

[7] Zhang, F. Y.; Corey, E. J.; *Org. Lett.* **2000**, *2*, 1097-1100.

[8] Tsubogo, T.; Saito, S.; Seki, K.; Yamashita, Y.; Kobayashi, S. *J. Am. Chem. Soc.* **2008**, *130*, 13321-13332.

Cyclopropenimine (9.2 mg, 0.0169 mmol, 0.1 equiv) and *tert*-butyl glycinate benzophenone Schiff base (50 mg, 0.169 mmol, 1.0 equiv) were dissolved in ethyl acetate (0.48 mL, 0.35 M). Michael-acceptor (0.508 mmol, 3.0 equiv) was then added to the vial and the reaction solution was stirred at room temperature. Upon complete consumption of starting material, monitored by ^{1}H NMR spectroscopy, the reaction solution was concentrated and the crude material subjected to silica gel column chromatography (Et_2O/Hexanes eluent as noted). Selectivities were determined via chiral HPLC as noted. Directly below the characterization of these substrates is a list of Michael acceptors that failed to give appreciable yield.

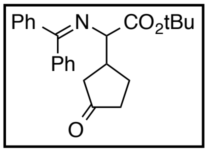

***tert*-Butyl 2-(diphenylmethyleneamino)-2-(3-oxocyclopentyl)ethanoate (Table 3, entry 1).** The general procedure was followed using 2-cyclopentenone (43 μL, 0.508 mmol, 3.0 equiv) as the Michael acceptor. After 48 hr the reaction mixture was purified by silica gel column chromatography (1/6 → 1/4 Et_2O/Hexanes) to yield the title product as a white solid as a mixture of diastereomers (56 mg, 0.148 mmol, 88% yield). ^{1}H NMR (500 MHz, $CDCl_3$) δ 7.62 (m, 2H, Ar**H**), 7.45-7.31 (m, 6H, Ar**H**), 7.15 (m, 2H, Ar**H**), 4.00 (d, J = 5.0 Hz, 1H, minor NC**H**CO_2tBu), 3.92 (d, J = 6.5 Hz, 1H, major NC**H**CO_2tBu), 2.92 (m, 1H, β-CH), 2.31 (m, 2H, -C**H**$_2$-), 2.17 (m, 2H, -C**H**$_2$-), 1.93 (m, 2H, -C**H**$_2$-) 1.45 (s, 9H, major C(C**H**$_3$)$_3$), 1.44 (s, 9H, minor C(C**H**$_3$)$_3$). ^{13}C NMR (125 MHz, $CDCl_3$) δ 171.4, 171.0*, 170.4, 170.3*, 139.4, 136.6, 130.6, 130.6*, 128.9, 128.8, 128.7, 128.2, 127.9, 81.6, 81.5*, 77.4, 69.1, 68.5*, 42.2, 41.0*, 40.3*, 40.1, 38.3, 28.2, 26.4*, 25.5 (* denotes minor diastereomer where observable). IR (thin film, cm^{-1}) 2964, 1743, 1723, 1627, 1283, 1222, 1143, 697. $[\alpha]^{20}_D$ = -7.5 (1.0 c, $CHCl_3$). HRMS (FAB+) m/z = 378.2063; calcd for $C_{24}H_{27}NO_3$ $[M+1]^+$ 378.1991. HPLC analysis: Chiralpak AD-H (Hex/EtOH = 98/2, 1.0 mL/min, 254 nm, 23°C), 7.3 min (minor diastereomer), 7.8 min (major diastereomer), 8.3 (major diastereomer), 9.5 (minor diastereomer), 78:22 dr, 4% ee major diastereomer, 30% ee minor diastereomer.

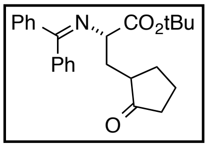

(2*S*)-*tert*-Butyl 2-(diphenylmethyleneamino)-3-(2-oxocyclopentyl)propanoate (Table 3, entry 2). The general procedure was followed using *tert*-butyl glycinate benzophenone Schiff base (25 mg, 0.0846 mmol, 1.0 equiv) and 2-methylenecyclopentanone (24.4 mg, 0.254 mmol, 3.0 equiv) as the Michael acceptor. After 45 min the reaction mixture was purified by silica gel column chromatography (1/4 Et_2O/Hexanes) to yield the title product as a clear oil as a 1:1 mixture of diastereomers (33.0 mg, 0.0843 mmol, 100% yield). ^{1}H NMR (400 MHz, $CDCl_3$) δ 7.64 (m, 2H, Ar**H**), 7.45-7.30 (m, 6H, Ar**H**), 7.18 (m, 2H, Ar**H**), 4.13 (dd, J = 5.6 and 7.4 Hz, 1H, NC**H**CO_2tBu), *3.97 (dd, J = 4.4 and 8.8 Hz, 1H, NC**H**CO_2tBu), 2.50 (m, 1H, C**H**C=O), *2.40 (m, 1H, C**H**C=O), 2.30-1.60 (m, 8H, 4 x –C**H**$_2$-), 1.44 (s, 9H, C(C**H**$_3$)$_3$), *1.43 (s, 9H, major C(C**H**$_3$)$_3$); *denotes diastereomer where observable. ^{13}C NMR (100 MHz, $CDCl_3$) δ 171.4, 171.3, 170.5, 170.4, 139.7, 139.6, 136.7, 136.6, 130.4, 130.4, 128.9, 128.9, 128.8, 128.7, 128.5, 128.1, 127.9, 127.8, 81.2, 81.2, 64.6, 64.1, 46.6, 37.9, 37.8, 34.0, 33.9, 30.6, 29.8, 28.1, 20.9, 20.8. (IR (thin film, cm^{-1}) 2970, 1733, 1661, 1600, 1276, 1153, 697, 638. $[\alpha]^{20}_D$ = -86.0 (0.5 c, $CHCl_3$). HRMS (FAB+) m/z = 392.2224; calcd for $C_{25}H_{29}NO_3$ $[M+1]^+$ 392.2147.

HPLC analysis: Chiralpak AD-H (Hex/IPA = 96/4, 1.0 mL/min, 254 nm, 23°C), 7.1 min (minor), 8.1 min (minor), 8.9 min (major), 9.9 min (major), 94 and 95% ee for the two diastereomers.

Enolates other than *tert*-butyl glycinate benzophenone imine (Equation 5).

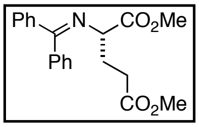

(*S*)-Dimethyl 2-(diphenylmethyleneamino)pentanedioate (19). [9] Cyclopropenimine (11.0 mg, 0.0197 mmol, 0.1 equiv) and methyl glycinate benzophenone imine (50 mg, 0.197 mmol, 1.0 equiv) were dissolved in ethyl acetate (0.56 mL, 0.35 M). Methyl acrylate (54.0 µL, 0.592 mmol, 3.0 equiv) was then added to the vial and the reaction solution was stirred at room temperature. After 45 min the reaction mixture was purified by silica gel column chromatography (1/4 Et_2O/Hexanes) to yield the title product as a colorless oil (67 mg, 0.197 mmol, 100% yield). ^{1}H NMR (400 MHz, $CDCl_3$) δ 7.63 (d, J = 7.1 Hz, 2H, Ar**H**), 7.46-7.32 (m, 6H, Ar**H**), 7.18 (m, 2H, Ar**H**), 4.13 (t, J = 6.3 Hz, 1H, NC**H**CO_2Me), 3.71 (s, 3H, $CO_2C\mathbf{H_3}$), 3.58 (s, 3H, $CO_2C\mathbf{H_3}$), 2.38-2.35 (m, 2H, -C$\mathbf{H_2}$-), 2.26-2.22 (m, 2H, -C$\mathbf{H_2}$-). ^{13}C NMR (100 MHz, $CDCl_3$) δ 173.4, 172.2, 171.3, 139.4, 136.2, 130.6, 129.0, 128.9, 128.7, 128.2, 127.9, 64.2, 52.3, 51.6, 30.5, 28.7. HPLC analysis: Chiralpak OD-H (Hex/IPA = 97/3, 1.0 mL/min, 254 nm, 23°C), 9.6 min (minor), 11.1 min (major), 96% ee.

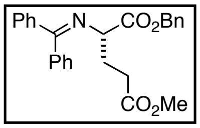

(*S*)-1-Benzyl 5-methyl 2-(diphenylmethyleneamino)pentanedioate (20).[10] Cyclopropenimine (8.3 mg, 0.0152 mmol, 0.1 equiv) and benzyl glycinate benzophenone imine (50 mg, 0.152 mmol, 1.0 equiv) were dissolved in ethyl acetate (0.43 mL, 0.35 M). Methyl acrylate (41.0 µL, 0.455 mmol, 3.0 equiv) was then added to the vial and the reaction solution was stirred at room temperature. After 45 min the reaction mixture was purified by silica gel column chromatography (1/3 Et_2O/Hexanes) to yield the title product as a colorless oil (62 mg, 0.149 mmol, 98% yield). ^{1}H NMR (400 MHz, $CDCl_3$) δ 7.65 (d, J = 7.2 Hz, 2H, Ar**H**), 7.40-7.31 (m, 11H, Ar**H**), 7.11 (m, 2H, Ar**H**), 5.15 (two d, J = 12.4 Hz, 2H, C$\mathbf{H_2}$Ph), 4.16 (t, J = 6.3 Hz, 1H, NC**H**CO_2Bn), 3.58 (s, 3H, $CO_2C\mathbf{H_3}$), 2.38-2.35 (m, 2H, -C$\mathbf{H_2}$-), 2.30-2.25 (m, 2H, -C$\mathbf{H_2}$-). ^{13}C NMR (100 MHz, $CDCl_3$) δ 173.5, 171.5, 139.4, 136.2, 135.9, 130.6, 129.0, 128.8, 128.6, 128.3, 128.2, 128.2, 127.9, 66.7, 64.3, 51.6, 30.5, 28.6. IR (thin film, cm^{-1}) 3028, 2947, 1736, 1624, 1443, 1256, 1160, 1256, 1078, 700. LRMS (APCI+) m/z = 417.26; calcd for $C_{26}H_{25}NO_4$ $[M+1]^+$ 416.18. HPLC analysis: Chiralpak OD-H (Hex/IPA = 97/3, 1.0 mL/min, 254 nm, 23°C), 11.7 min (minor), 12.4 min (major), 97% ee.

---

[9] Saito, S.; Tsubogo, T.; Kobayashi, S. *J. Am. Chem. Soc.* **2007**, *129*, 5364.
[10] Shibuguchi, T.; Mihara, H.; Kuramochi, A.; Sakuraba, S.; Ohshima, T.; Shibasaki, M. *Angew. Chem. Int. Ed.* **2006**, *45*, 4635.

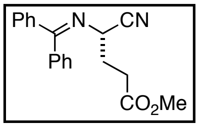

(*S*)-Methyl 4-cyano-4-(diphenylmethyleneamino)butanoate (21). Cyclopropenimine (24.8 mg, 0.0454 mmol, 0.1 equiv) and 2-(diphenylmethyleneamino)ethanenitrile (100 mg, 0.454 mmol, 1.0 equiv) were dissolved in ethyl acetate (1.30 mL, 0.35 M). Methyl acrylate (123 μL, 1.36 mmol, 3.0 equiv) was then added to the vial and the reaction solution was stirred at room temperature. After 15 min the reaction mixture was purified by silica gel column chromatography (1/4 Et_2O/Hexanes) to yield the title product as a colorless oil (135 mg, 0.441 mmol, 97% yield). ^1H NMR (400 MHz, $CDCl_3$) δ 7.65 (m, 2H, Ar**H**), 7.52-7.42 (m, 4H, Ar**H**), 7.35 (t, J = 7.8 Hz, 2H, Ar**H**), 7.20 (dd, J = 2.7 and 7.7 Hz, 2H, Ar**H**), 4.35 (t, J = 6.4 Hz, 1H, C**H**CN), 3.61 (s, 3H, $CO_2C\mathbf{H_3}$), 2.60-2.51 (m, 2H, -C$\mathbf{H_2}$-), 2.24-2.22 (m, 2H, -C$\mathbf{H_2}$-). ^{13}C NMR (100 MHz, $CDCl_3$) δ 173.8, 172.6, 138.3, 135.1, 131.4, 129.5, 129.1, 128.3, 127.4, 119.1, 51.9, 51.8, 30.0, 29.9. IR (thin film, cm^{-1}) 3066, 2953, 1726, 1624, 1443, 1215, 1153, 731, 697. $[\alpha]^{20}_D$ = -28.4 (1.0 c, $CHCl_3$). HRMS (FAB+) m/z = 307.1444; calcd for $C_{19}H_{18}N_2O_2$ $[M+1]^+$ 307.1368. HPLC analysis: Chiralpak OD-H (Hex/IPA = 98.5/1.5, 0.7 mL/min, 254 nm, 23°C), 19.3 min (minor), 20.2 min (major), 10% ee.

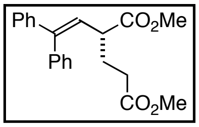

(*R*)-Dimethyl 2-(2,2-diphenylvinyl)pentanedioate (22). Cyclopropenimine (54 mg, 0.099 mmol, 0.1 equiv) and methyl 4,4-diphenylbut-3-enoate (250 mg, 0.99 mmol, 1.0 equiv) were dissolved in ethyl acetate (2.8 mL, 0.35 M). Methyl acrylate (270 μL, 2.97 mmol, 3.0 equiv) was then added to the vial and the reaction solution was stirred at room temperature. After 48 hr the reaction mixture was purified by silica gel column chromatography (1/9 Et_2O/Hexanes) to yield the title product as a colorless oil (37 mg, 0.109 mmol, 11% yield). Unreacted starting material methyl 4,4-diphenylbut-3-enoate was also isolated (195 mg, 0.77 mmol, 78% recovery). ^1H NMR (500 MHz, $CDCl_3$) δ 7.48-7.19 (m, 10H, Ar**H**), 6.02 (d, J = 10.5 Hz, 1H, C=C**H**), 3.70 (s, 3H, $CO_2C\mathbf{H_3}$), 3.57 (s, 3H, $CO_2C\mathbf{H_3}$), 3.25 (m, 1H, C=CHC**H**CO_2Me), 2.28 (m, 2H, C$\mathbf{H_2}CO_2$Me), 2.07 and 1.94 (m, 2H, -C$\mathbf{H_2}$-). ^{13}C NMR (125 MHz, $CDCl_3$) δ 117.2, 173.3, 145.2, 141.7, 139.2, 129.9, 128.5, 128.3, 127.7, 127.6, 127.5, 125.5, 52.1, 51.7, 31.5, 27.9. IR (thin film, cm^{-1}) 2950, 1733, 1494, 1440, 1205, 1157, 765, 700. $[\alpha]^{20}_D$ = -141.4 (0.5 c, $CHCl_3$). LRMS (APCI+) m/z = 339.25; calcd for $C_{21}H_{21}O_4$ $[M+1]^+$ 338.14. HPLC analysis: Chiralpak OD-H (Hex/IPA = 98/2, 0.8 mL/min, 254 nm, 23°C), 10.5 min (minor), 11.1 min (major), 71% ee.

General Procedure for Glycinate-Michael Additions with Substituted Acceptors (Table 6).

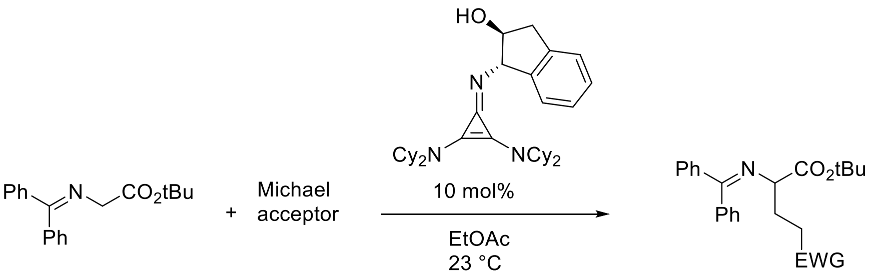

Cyclopropenimine (9.2 mg, 0.0169 mmol, 0.1 equiv) and *tert*-butyl glycinate benzophenone Schiff base (50 mg, 0.169 mmol, 1.0 equiv) were dissolved in ethyl acetate (0.48 mL, 0.35 M). Michael-acceptor (0.508 mmol, 3.0 equiv) was then added to the vial and the reaction solution was stirred at room temperature. Upon complete consumption of starting material, monitored by ^{1}H NMR spectroscopy, the reaction solution was concentrated and the crude material subjected to silica gel column chromatography (Et_2O/Hexanes eluent as noted). Selectivities were determined via chiral HPLC as noted. Directly below the characterization of these substrates is a list of Michael acceptors that failed to give appreciable yield.

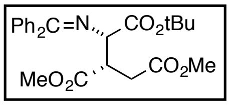

(1*S*,2*S*)-1-*tert*-Butyl 2,3-dimethyl 1-(diphenylmethyleneamino)propane-1,2,3-tricarboxylate (Table 6, entry 1). The general procedure was followed using dimethyl fumarate (73 mg, 0.508 mmol, 3.0 equiv) as the Michael acceptor. After 2.5 hr the reaction mixture was purified by silica gel column chromatography (1/10 → 1/4 Et_2O/Hexanes) to yield the title product as a clear oil (70 mg, 0.159 mmol, 94% yield). ^{1}H NMR (500 MHz, $CDCl_3$) δ 7.60 (m, 2H, Ar**H**), 7.45-7.31 (m, 6H, Ar**H**), 7.15 (m, 2H, Ar**H**), 4.42 (d, J = 5.0 Hz, 1H, NC**H**CO_2tBu), 3.65 (s, 3H, CO_2C**H$_3$**), 3.63 (m, 1H, C**H**CO_2Me), 3.60 (s, 3H, CO_2C**H$_3$**) 3.10 (dd, J = 9.6 and 17.0 Hz, 1H, C**H**CO_2Me), 2.68 (dd, J = 3.9 and 17.0 Hz, 1H, C**H**CO_2Me), 1.44 (s, 9H, C(C**H$_3$**)$_3$); ^{13}C NMR (125 MHz, $CDCl_3$) δ 172.9, 172.7, 172.2, 169.3, 139.4, 136.1, 130.6, 129.1, 128.9, 128.4, 128.1, 128.0, 82.1, 66.2, 52.1, 51.9, 44.7, 32.4, 28.1. IR (thin film, cm^{-1}) 2977, 2950, 1733, 1624, 1443, 1222, 1150, 697. $[\alpha]^{20}_{D}$= -96.5 (1.0 c, $CHCl_3$). HRMS (FAB+) m/z = 440.2073; calcd for $C_{25}H_{29}NO_6$ $[M+1]^+$ 440.1995. HPLC analysis: Chiralpak AD-H (Hex/IPA = 98/2, 1.0 mL/min, 254 nm, 23°C), 12.4 min (minor), 25.9 min (major), 96% ee.

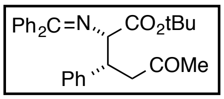

(2*S*,3*R*)-*tert*-Butyl 2-(diphenylmethyleneamino)-5-oxo-3-phenylhexanoate (Table 6, entry 3).[6] The general procedure was followed using benzylideneacetone (73 mg, 0.508 mmol, 3.0 equiv) as the Michael acceptor. After 7 hr the reaction mixture was purified by silica gel column chromatography (1/20 → 1/4 Et_2O/Hexanes) to yield the title product as a white solid (74 mg, 0.169 mmol, quantitative yield). ^{1}H NMR (400 MHz, $CDCl_3$) δ 7.66 (m, 2H, Ar**H**),

7.35-7.10 (m, 11H, Ar**H**), 6.70 (d, J = 7.0 Hz, 2H, Ar**H**), 4.05 (d, J = 7.0 Hz, 1H, NC**H**CO_2tBu), 3.98 (m, 1H, PhC**H**), 3.15-3.01 (m, 1H, C**H$_2$**COMe), 2.07 (s, 3H, C**H$_3$**), 1.31 (s, 9H, C(C**H$_3$**)$_3$); ^{13}C NMR (125 MHz, $CDCl_3$) δ 207.5, 171.2, 170.0, 141.3, 139.4, 136.3, 130.5, 128.9, 128.7, 128.5, 128.3, 128.3, 128.1, 127.6, 126.8, 81.4, 70.9, 45.4, 44.7, 30.4, 28.0. IR (thin film, cm^{-1}) 2981, 1719, 1621, 1361, 1147, 697. LRMS (APCI+) m/z = 442.20; calcd for $C_{29}H_{31}NO_3$ $[M+1]^+$ 442.23. HPLC analysis: Chiralpak AD-H (Hex/IPA = 95/5, 1.0 mL/min, 254 nm, 23°C), 6.4 min (minor), 8.1 min (major), 91% ee.

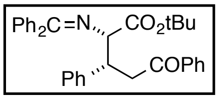

(2*S*,3*R*)-*tert*-Butyl 2-(diphenylmethyleneamino)-5-oxo-3,5-diphenylpentanoate (Table 6, entry 4).[6] The general procedure was followed on a slightly smaller scale using cyclopropenimine (3.6 mg, 0.00677 mmol, 0.1 equiv), *tert*-butyl glycinate benzophenone Schiff base (20 mg, 0.0677 mmol, 1.0 equiv) and *trans*-chalcone (42, 0.203 mmol, 3.0 equiv) in ethyl acetate (0.20 mL, 0.35 M). After 1.5 hr the reaction mixture was purified by silica gel column chromatography (1/9 → 1/4 Et_2O/Hexanes) to yield the title product as a clear oil (85 mg, 0.169 mmol, quantitative yield. ^{1}H NMR (400 MHz, $CDCl_3$) δ 8.00 (d, 7.2 Hz, 2H, Ar**H**), 7.70 (d, 7.2 Hz, 2H, Ar**H**), 7.30-7.60 (m, 9H, Ar**H**), 7.10-7.20 (m, 5H, Ar**H**), 6.70 (m, 2H, Ar**H**), 4.20 (m, 2H, NC**H**$CO_2{}^t$Bu, C**H**Ph), 3.60-3.80 (m, 2H, C**H$_2$**COPh), 1.33 (s, 9H, C(C**H$_3$**)$_3$); ^{13}C NMR (125 MHz, $CDCl_3$) δ 198.8, 171.2, 170.1, 141.5, 139.5, 137.3, 136.4, 132.9, 130.4, 129.0, 128.7, 128.6, 128.5, 128.3, 128.3, 128.2, 128.1, 127.6, 126.7, 81.4, 44.9, 40.1, 28.0. HPLC analysis: Chiralpak AD-H (Hex/IPA = 95/5, 1.0 mL/min, 254 nm, 23°C), 8.8 min (minor), 11.0 min (major), 96% ee.

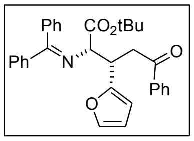

tert-butyl (2S,3S)-2-((diphenylmethylene)amino)-3-(furan-2-yl)-5-oxo-5-phenylpentanoate (Table 6, entry 5). The general procedure was followed using (*E*)-3-(furan-2-yl)-1-phenylprop-2-en-1-one[11] (101 mg, 0.508 mmol, 3.0 equiv) as the Michael acceptor. After 7 hr the reaction mixture was purified by silica gel column chromatography (1/10 Et_2O/Hexanes) to yield the title product as a clear oil (83 mg, 0.167 mmol, 99%). ^{1}H NMR (300 MHz, $CDCl_3$) δ 8.04 (d, J = 5.2 Hz, 2H, Ar**H**), 7.68 (d, J = 8.4 Hz, 2H, Ar**H**), 7.64-7.30 (m, 10H, Ar**H**), 7.21 (m, 1H, Ar**H**), 6.93 (m, 1H, Ar**H**), 6.21 (m, 1H, Ar**H**), 6.00 (d J = 3.2 Hz, 1H, Ar**H**), 4.35 (m, 2H, C**H**C**H**CH_2COPh), 3.78 (m, 1H, C**H$_a$**COPh**)**, 3.58 (m, 1H, C**H$_b$**COPh**)**, 1.41 (s, 9H, C(C**H$_3$**)$_3$). ^{13}C NMR (125 MHz, $CDCl_3$) 198.3, 171.5, 169.8, 154.9, 145.0, 139.6, 137.1, 136.3, 132.9, 130.3, 128.9, 128.5, 128.3, 128.2, 128.0, 127.7, 110.2, 106.5, 81.5, 68.71, 38.46, 27.97. IR (thin film, cm^{-1}) 1729, 1658, 1447, 1277, 11150, 756, 701. LRMS (APCI+) m/z = 494.26; calcd for $C_{30}H_{40}NO_6$ $[M]^+$ 494.23. HPLC analysis: Chiralpak AD-H (Hex/IPA = 95/5, 1.0 mL/min, 254 nm, 23°C), 5.5 min (minor diastereomer), 6.1 (minor diastereomer), 9.0 (major diastereomer) 10.3 min (major diastereomer), 20:1 dr, 96% ee major diasteroisomer. Note: other diastereomer are visible in HPLC trace and account for <5% of the material.

[11] Songbay, L.; Liebskind, L.; *J. Am. Chem. Soc.* **2008**, 130, 6918.

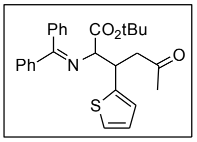

tert-butyl 2-((diphenylmethylene)amino)-5-oxo-3-(thiophen-2-yl)hexanoate (Table 6, entry 6). The general procedure was followed using (*E*)-4-(thiophen-2-yl)but-3-en-2-one[12] (77 mg, 0.508 mmol, 3.0 equiv) as the Michael acceptor. After 22 hr the reaction mixture was purified by silica gel column chromatography (1/20 → 1/4 Et_2O/Hexanes) to yield the title product as a yellow oil (71 mg, 0.159 mmol, 94%). ^{1}H NMR (400 MHz, $CDCl_3$) δ 7.70 (m, 2H, Ar**H**), 7.45-7.30 (m, 6H, Ar**H**), 7.10 (dd, J = 1.2 Hz and 5.2 Hz, 1H, Ar**H**), 6.89-6.79 (m, 4H, Ar**H**), 4.33 (m, 1H, CH_3COCH_2C**H**), 4.13 (d, J = 4.0 Hz, 1H, NC**H**CO_2tBu), 3.24 (dd, J = 9.6 Hz and 16.8 Hz, 1H, COCHC**H**$_a$), 3.12 (dd, J = 8.0 Hz and 16.8 Hz, 1H, COCHC**H**$_b$), 2.15 (s, 3H, C**H**$_3$CO), 1.40 (s, 9H, C(C**H**$_3$)$_3$). ^{13}C NMR (125 MHz, $CDCl_3$) δ 206.9, 171.7, 169.6, 144.76, 139.4, 136.3, 130.4, 1289.0, 128.4, 128.2, 128.0, 127.5, 126.4, 125.2, 123.9, 81.6, 70.7, 46.5, 39.8, 30.4, 27.9. IR (thin film, cm^{-1}) 1726, 1624, 1445, 1367, 1283, 1149, 754, 697.LRMS (APCI+) m/z = 448.67; calcd for $C_{27}H_{30}NO_3S$ $[M+1]^+$ 448.60. HPLC analysis: Chiralpak AD-H (Hex/IPA = 95/5, 1.0 mL/min, 254 nm, 23°C), 6.1 min (major diastereomer), 7.4 (major diastereomer), 8.7 (minor diastereomer) 9.9 min (minor diastereomer), >20:1 dr, 88% ee major diasteroisomer. Note: other diastereomer are visible in HPLC trace and account for <5% of the material.

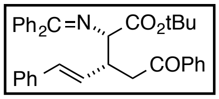

(2*S*,3*R*,*E*)-*tert*-Butyl 2-(diphenylmethyleneamino)-3-(2-oxo-2-phenylethyl)-5-phenylpent-4-enoate (Table 6, entry 7). The general procedure was followed using (2*E*,4*E*)-1,5-diphenylpenta-2,4-dien-1-one[13] (119 mg, 0.508 mmol, 3.0 equiv) as the Michael acceptor. After 8 hr the reaction mixture was purified by silica gel column chromatography (1/9 Et_2O/Hexanes) to yield the title product as a white solid (56 mg, 0.106 mmol, 63% yield). ^{1}H NMR (500 MHz, $CDCl_3$) δ 7.98 (d, J = 7.4 Hz, 2H, Ar**H**), 7.70 (d, J = 7.3 Hz, 2H, Ar**H**), 7.40 (t, J = 7.5 Hz, 1H, Ar**H**), 7.50-7.32 (m, 8H, Ar**H**), 7.21 (d, J = 4.3 Hz, 2H, Ar**H**), 7.15 (m, 3H, Ar**H**), 6.35 (d, J = 15.9 Hz, 1H, C=C**H**Ph), 6.03 (dd, J = 8.6 and 15.9 Hz, 1H, **H**C=CHPh), 4.12 (d, J = 5.7 Hz, 1H, NC**H**$CO_2{}^t$Bu), 3.71 (m, 1H, **H**CC=C), 3.42-3.33 (m, 2H, -C**H**$_2$-), 1.38 (s, 9H, C(C**H**$_3$)$_3$). ^{13}C NMR (125 MHz, $CDCl_3$) δ 199.1, 171.4, 170.3, 139.6, 137.4, 137.3, 136.6, 133.0, 132.0, 130.6, 129.8, 129.0, 128.7, 128.7, 128.5, 128.4, 128.2, 128.0, 127.3, 126.3, 81.5, 69.9, 43.2, 40.4, 28.2. IR (thin film, cm^{-1}) 2977, 1726, 1685, 1447, 1283, 1147, 969, 751, 693. $[\alpha]^{20}_D$ = -28.8 (1.0 c, $CHCl_3$). HRMS (FAB+) m/z = 530.2679; calcd for $C_{36}H_{35}NO_3$ $[M+1]^+$ 530.2617. HPLC analysis: Chiralpak OD-H (Hex/EtOH = 97/3, 1.0 mL/min, 254 nm, 23°C), 4.9 min (minor), 6.1 min (major), 92% ee. Employing the same procedure with cyclopropenimine **23**, the isolated in a 45% yield (41 mg, 76 mmol) and the ee obtained was 94%.
Note: other diastereomer and regioisomer are visible in HPLC trace and account for <5% of the material.

[12] Le P.Q.; *Org. Lett.* **2012,** *14*, 6105.
[13] Batovska, D.; Parushev, S.; Stamboliyska, B.; Tsvetkova, I.; Ninova, M.; Najdenski, H. *Eur. J. Med. Chem.* **2009**, *44*, 2211.

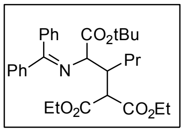

3-(tert-butyl) 1,1-diethyl 3-((diphenylmethylene)amino)-2-propylpropane-1,1,3-tricarboxylate (Table 6, Entry 8). The general procedure was followed using 3-(tert-butyl) 1,1-diethyl 3-((diphenylmethylene)amino)-2-propylpropane-1,1,3-tricarboxylate[14] (108 mg, 0.508 mmol, 3.0 equiv) as the Michael acceptor. After 22 hr the reaction mixture was purified by silica gel column chromatography (1/20 → 1/4 Et_2O/Hexanes) to yield the title product as a yellow oil (89 mg, 0.169 mmol, quantitative yield). A 9:1 diasteromeric ration was determined on the crude proton NMR. ^{1}H NMR (300 MHz, $CDCl_3$) δ 7.65 (m, 2H, Ar**H**), 7.48-7.30 (m, 6H, Ar**H**), 7.15 (m, 2H, Ar**H**), 4.20 (m, 4H, O**CH₂**CH₃), 4.03 (d, J = 4.0 Hz, 1H, **H**C(CO_2Et)$_2$), 3.90 (d, J = 8.0 Hz, 1H, NC**H**CO_2tBu major diasteroisomer), 3.72 (d, J = 8.0 Hz, 1H, NC**H**CO_2tBu minor diastereoisomer), 2.96 (m, 1H, CHC**H**-Pr), 1.45 (s, 9H, C(**CH₃**)$_3$), 1.26 (m, 4H, CH**CH₂CH₂**CH₃), 0.84 (t, J = 7.2, CH_2CH_2**CH₃**). ^{13}C NMR (125 MHz, $CDCl_3$) 170.6, 169.4, 169.0, 139.5, 136.5, 130.2, 129.0, 128.5, 128.3, 127.8, 127.7, 81.3, 66.7, 61.20, 60.9, 53.2, 42.2, 31.7, 28.0, 20.8, 14.0. IR (thin film, cm^{-1}) 1728, 1624, 1446, 1368, 1149, 755, 689. LRMS (APCI+) m/z = 510.76; calcd for $C_{30}H_{40}NO_6$ $[M+1]^+$ 510.65.HPLC analysis: Chiralpak AD-H (Hex/IPA = 98/2, 1.0 mL/min, 254 nm, 23°C), 5.5 min (minor diastereomer), 6.9 (minor diastereomer), 7.4 (major diastereomer) 8.0 min (major diastereomer), 9:1 dr, 95% ee minor diasteroisomer (minor enantiomer not detected) 92% ee major diasteroisomer.

[14] Rousseau, O.; Delaunay, T, Robiette, R. *Synlett*. **2014**, 25, 519.

Michael acceptors with <10% conversion in 72 hr:

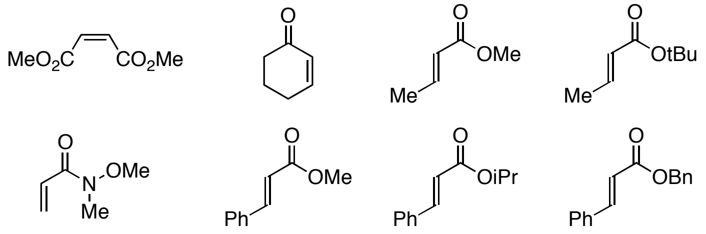

Cyclopropenimine decomposition study (Table 4).

Decomposition as a solid at room temperature rate study: Freshly obtained cyclopropenimine was placed in a vial (250 mg per vial) and stored at room temperature At each time point, a small amount of the solid was removed and its ^{1}H NMR ($CDCl_3$) was used to measure the ratio of cyclopropenimine to the rearranged product. The time required for 50% of the cyclopropepenimine to rearrange is noted as the half-life. For iminoindanol catalyst **36**, no decomposition was noted over a three-month period.

Decomposition in solution rate study: Freshly obtained cyclopropenimine (0.028 mmol) was dissolved in d_8-toluene (0.8 mL, 0.035 M) containing benzyl ether standard (0.007 mmol). This concentration is identical to that of cyclopropenimine in cyclopropenimine-catalyzed Michael reactions. At each time point, ^{1}H NMR was used to measure the ratio of cyclopropenimine to benzyl ether standard. The time required for 50% of the cyclopropepenimine to decompose is noted as the half-life.

The rates of decomposition were found to be identical when *tert*-butyl glycinate benzophenone Schiff base (82.7 mg, 0.280 mmol, 10 equiv relative to cyclopropenimine) was included in the d_8-toluene solution.

entry	catalyst	conditions	$t_{1/2}$
1	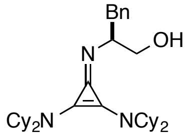 **1**	(a) solid, rt	15 d
		(b) solid –20 °C	8 mo
		(c) HCl salt, rt	>5 y
		(d) 0.035 M PhMe	7 h
2	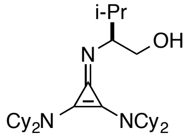 **30**	solid, rt	16 h
3	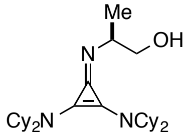 **29**	solid, rt	5 mo
4	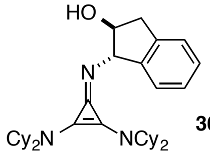 **36**	(a) solid, rt	>5 y
		(b) 0.035 M	36 h
5	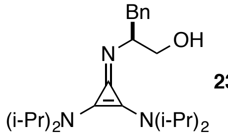 **23**	(a) solid, rt	12 h
		(b) 0.035 M	3 h

Comparison of one-month-old catalysts.

After storing deprotonated cyclopropenimine catalysts **1** and **36** as solids (200 mg each) at 23 °C for one month in a desiccator their activity was reassessed as follows. Cyclopropenimine decomposition solid (9.2 mg, originally 0.0169 mmol, 0.1 equiv; note: mmol and equiv correspond to value of cyclopropenimine prior to decomposition) and *tert*-butyl glycinate benzophenone Schiff base (50 mg, 0.169 mmol, 1.0 equiv) were dissolved in ethyl acetate (0.48 mL, 0.35 M). Methyl acrylate (54 µL, 0.508 mmol, 3.0 equiv) was then added to the vial and the reaction solution was stirred at room temperature. The reaction was monitored by ^{1}H NMR spectroscopy and conversion was determined by comparing *tert*-butyl integrals of the starting material and product. Upon 95% conversion, or after 24 hr if not complete, the reaction solution was concentrated and the crude material subjected to silica gel column chromatography (1/5 Et_2O/Hexanes) to yield pure product. Enantiomeric excesses were determined via chiral HPLC: Chiralpak AD-H (Hex/IPA = 97/3, 1.0 mL/min, 254 nm, 23°C), 6.1 min (minor), 6.7 min (major).

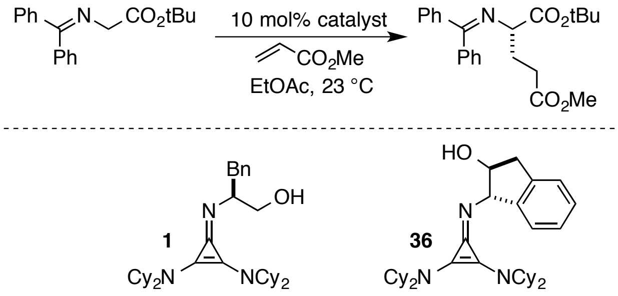

	1	36
**Fresh:**	1 h, 99% yield, 98% ee	2 h, 95% con, 98% ee
**Month old:**	24 h, 72% con, 98% ee	2 h, 99% yield, 97% ee

Product racemization test.

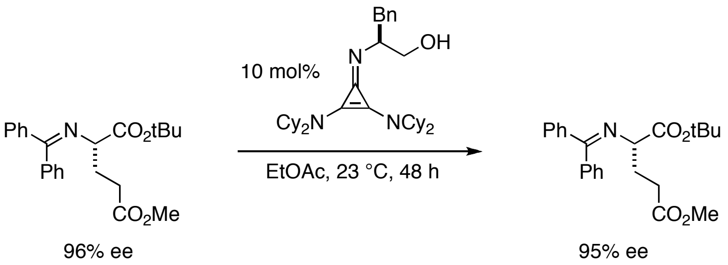

Michael adduct **12a** (50 mg, 0.131 mmol, 1.0 equiv) and cyclopropenimine **1** (7.2 mg, 0.0131 mmol, 0.1 equiv) were dissolved in ethyl acetate (0.37 mL, 0.35 M). The reaction solution was stirred for 48 hr at room temperature after which time the Michael adduct was reisolated via silica gel column chromotagraphy (1/5 Et_2O/Hexanes eluent). Enantiomeric excesses were determined via chiral HPLC: Chiralpak AD-H (Hex/IPA = 97/3, 1.0 mL/min, 254 nm, 23°C), 6.1 min (minor), 6.7 min (major). Original sample: 96% ee; reisolated sample: 95% ee.

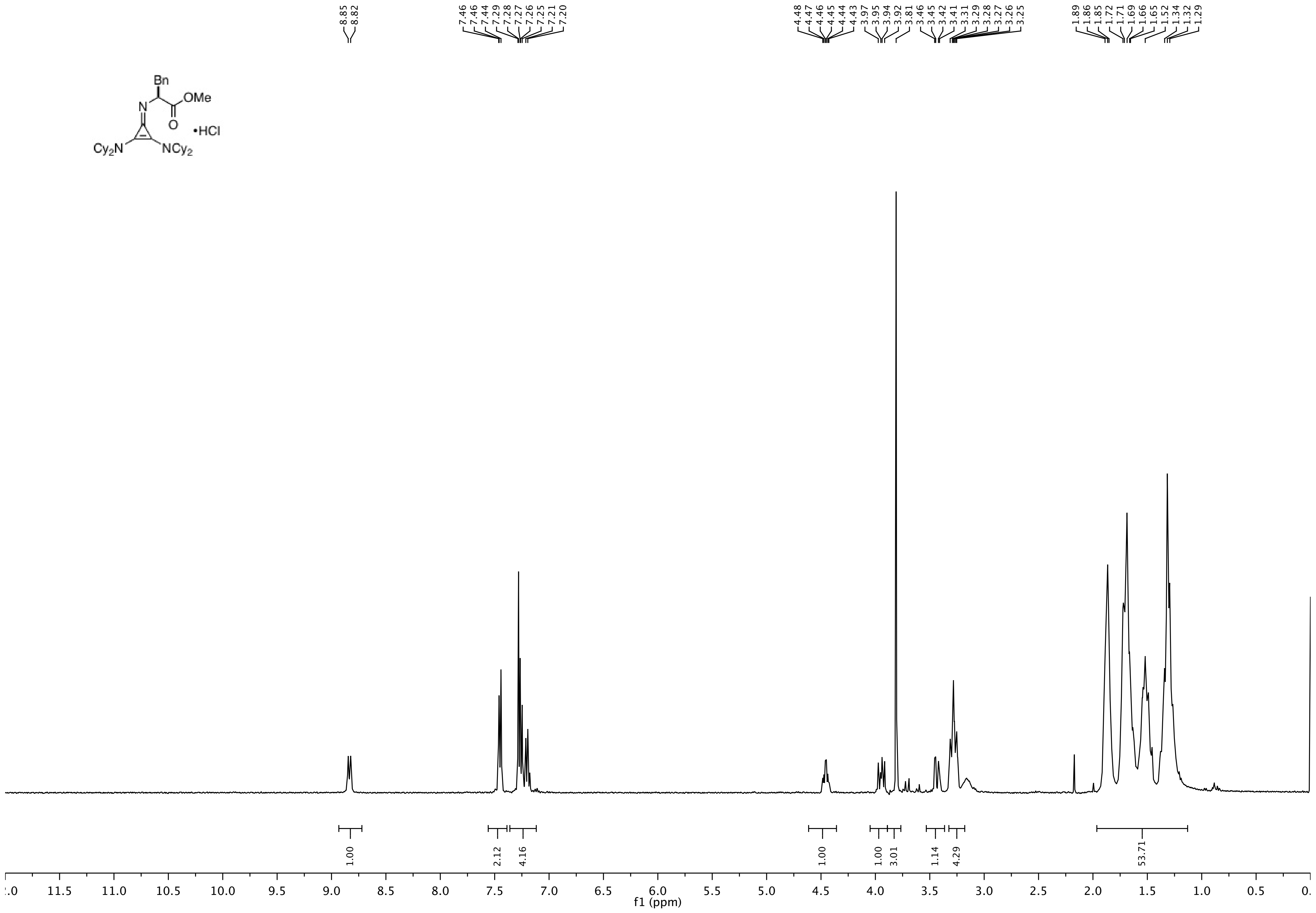

8.85
8.82
7.46
7.46
7.44
7.29
7.28
7.27
7.26
7.25
7.21
7.20
4.48
4.47
4.46
4.45
4.44
4.43
3.97
3.95
3.94
3.92
3.81
3.46
3.45
3.42
3.41
3.31
3.29
3.28
3.27
3.26
3.25
1.89
1.86
1.85
1.72
1.71
1.69
1.66
1.65
1.52
1.34
1.32
1.29
Bn
N
OMe
O
•HCl
Cy2N
NCy2
1.00
2.12
4.16
1.00
1.00
3.01
1.14
4.29
53.71
11.5
11.0
10.5
10.0
9.5
9.0
8.5
8.0
7.5
7.0
6.5
6.0
5.5
5.0
4.5
4.0
3.5
3.0
2.5
2.0
1.5
1.0
0.5
f1 (ppm)

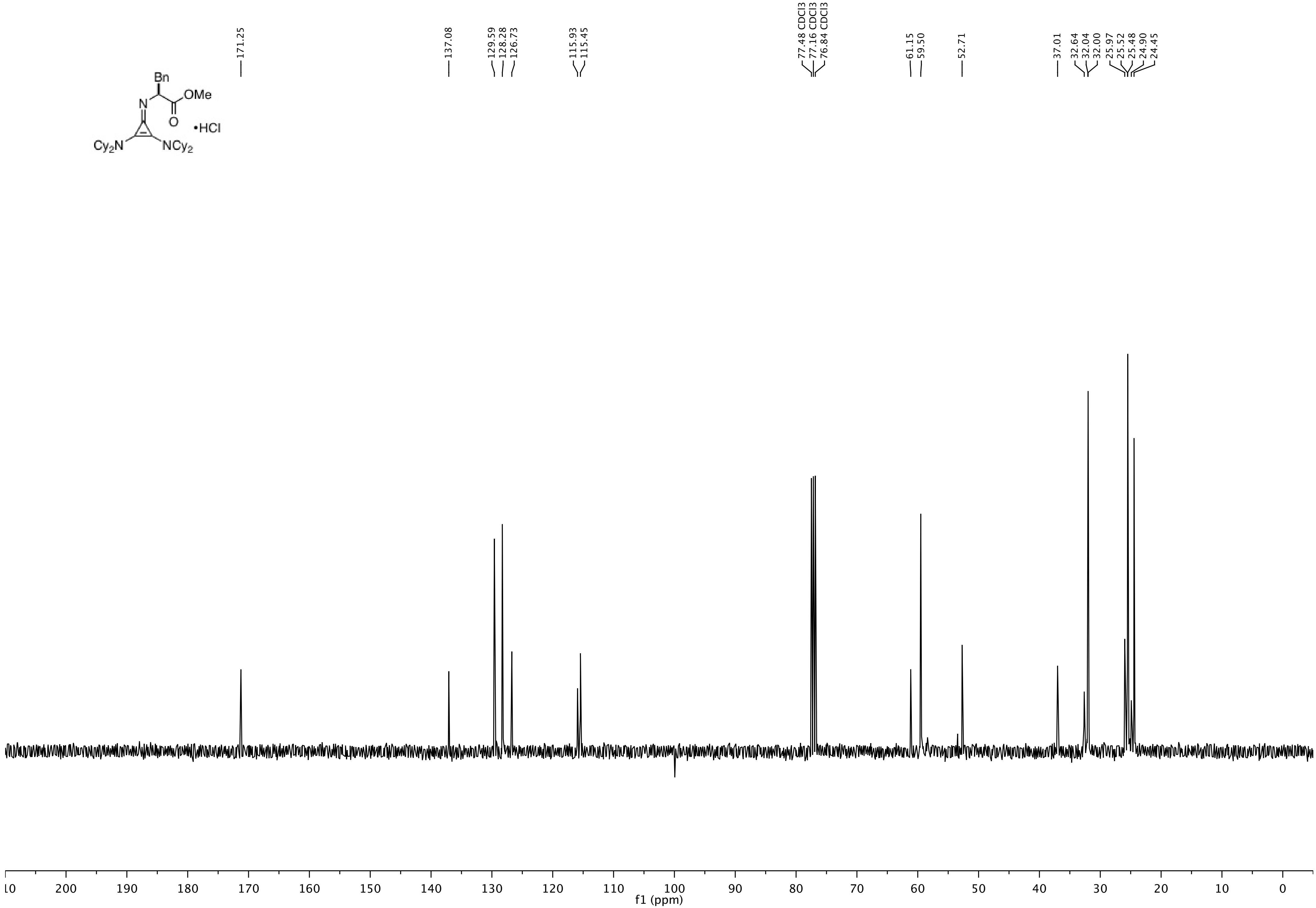

171.25
137.08
129.59
128.28
126.73
115.93
115.45
77.48 CDCl3
77.16 CDCl3
76.84 CDCl3
61.15
59.50
52.71
37.01
32.64
32.04
32.00
25.97
25.52
25.48
24.90
24.45
Bn
OMe
N
O
•HCl
Cy_2N
NCy_2
f1 (ppm)

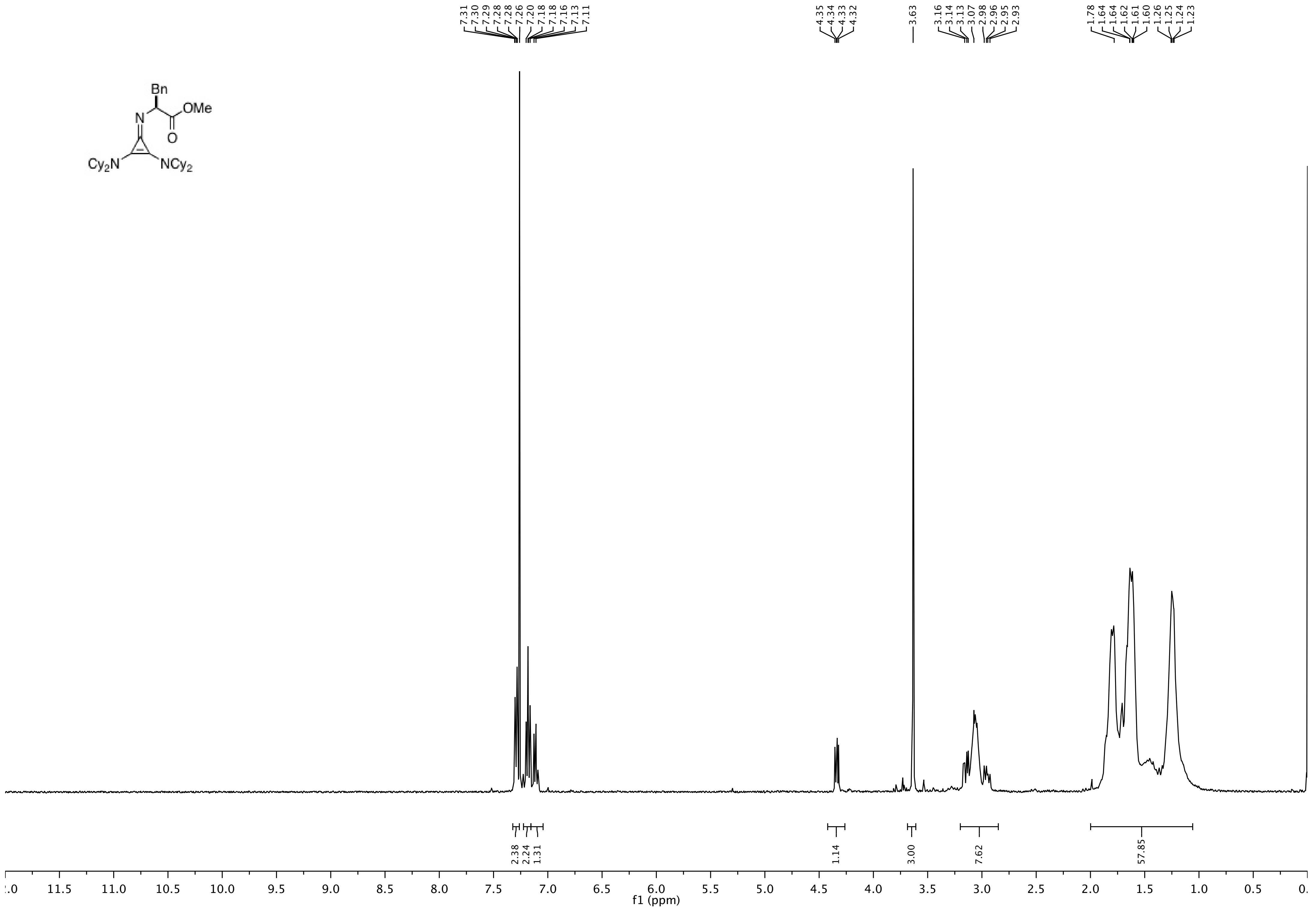

Bn
N
OMe
O
Cy2N
NCy2
7.31 7.30 7.29 7.28 7.28 7.26 7.20 7.18 7.18 7.16 7.13 7.11
4.35 4.34 4.33 4.32
3.63
3.16 3.14 3.13 3.07 2.98 2.96 2.95 2.93
1.78 1.64 1.62 1.61 1.60 1.26 1.25 1.24 1.23
2.38 2.24 1.31
1.14
3.00
7.62
57.85
11.5 11.0 10.5 10.0 9.5 9.0 8.5 8.0 7.5 7.0 6.5 6.0 5.5 5.0 4.5 4.0 3.5 3.0 2.5 2.0 1.5 1.0 0.5
f1 (ppm)

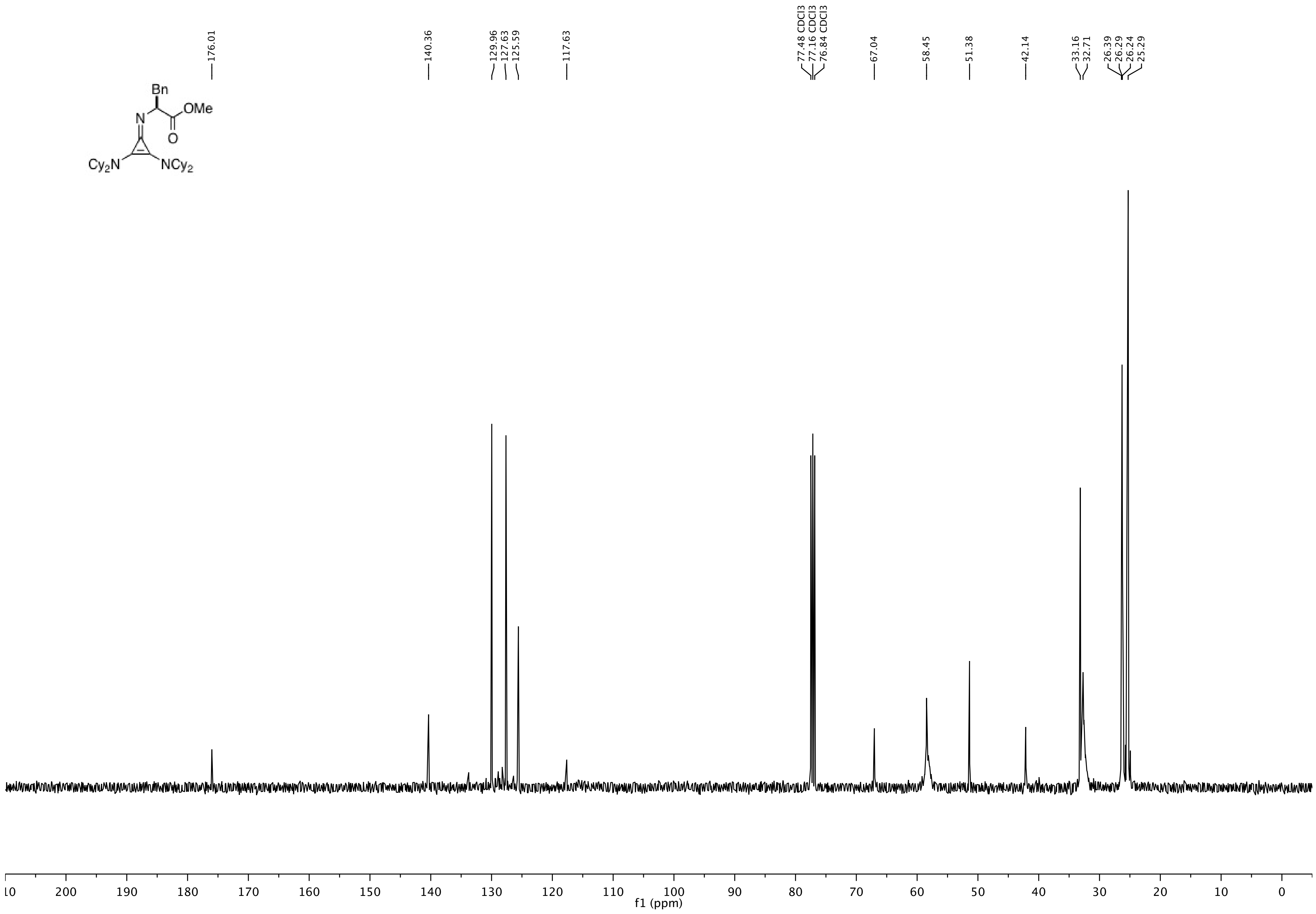
176.01
140.36
129.96
127.63
125.59
117.63
77.48 CDCl3
77.16 CDCl3
76.84 CDCl3
67.04
58.45
51.38
42.14
33.16
32.71
26.39
26.29
26.24
25.29
Bn
OMe
N
O
Cy_2N
NCy_2
200
190
180
170
160
150
140
130
120
110
100
90
80
70
60
50
40
30
20
10
0
f1 (ppm)

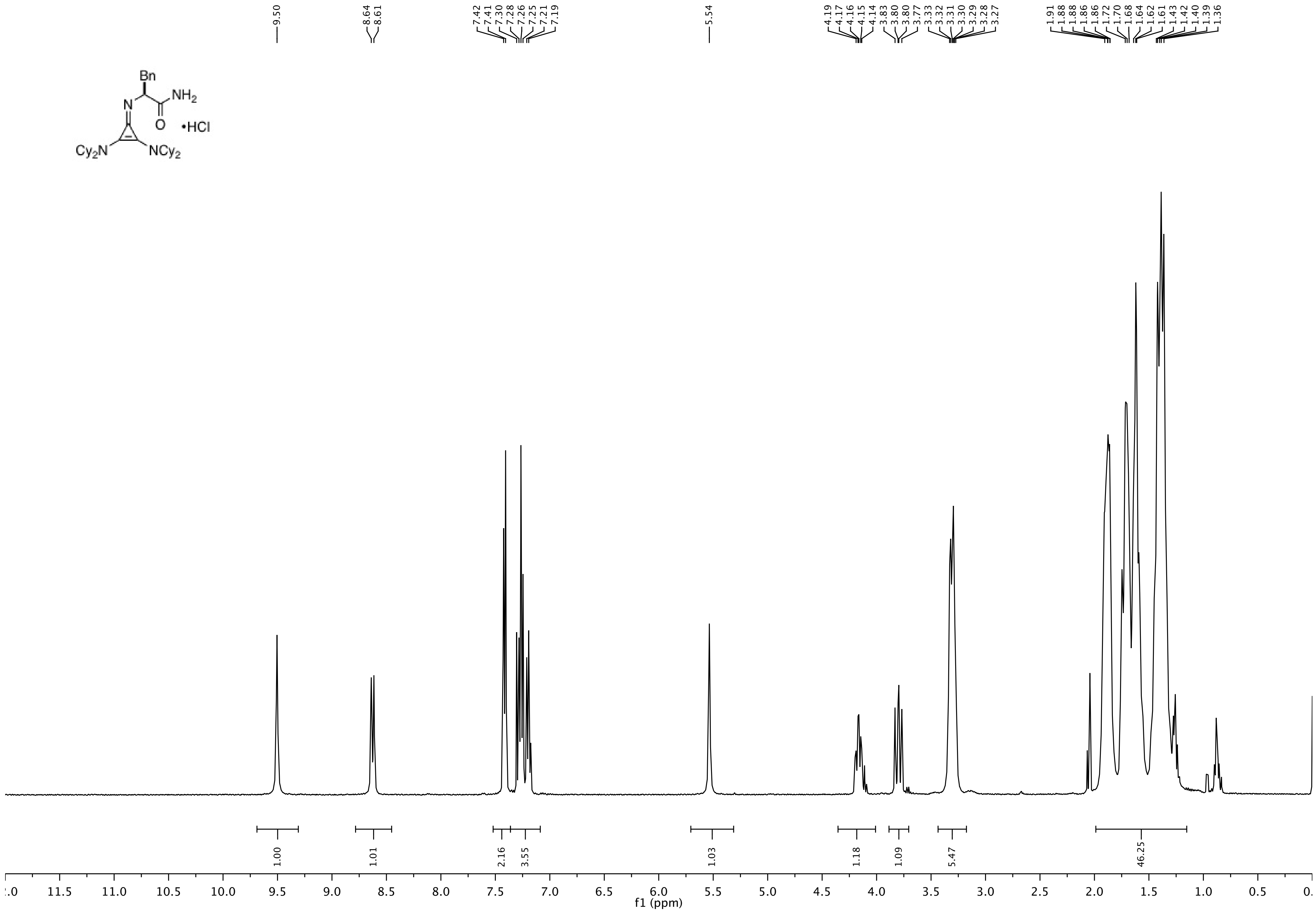

9.50
8.64
8.61
7.42
7.41
7.30
7.28
7.26
7.25
7.21
7.19
5.54
4.19
4.17
4.16
4.15
4.14
3.83
3.80
3.80
3.77
3.33
3.32
3.31
3.30
3.29
3.28
3.27
1.91
1.88
1.88
1.86
1.86
1.72
1.70
1.68
1.64
1.62
1.61
1.43
1.42
1.40
1.39
1.36
Bn
N
NH2
O
•HCl
Cy2N
NCy2
1.00
1.01
2.16
3.55
1.03
1.18
1.09
5.47
46.25
11.5
11.0
10.5
10.0
9.5
9.0
8.5
8.0
7.5
7.0
6.5
6.0
5.5
5.0
4.5
4.0
3.5
3.0
2.5
2.0
1.5
1.0
0.5
f1 (ppm)

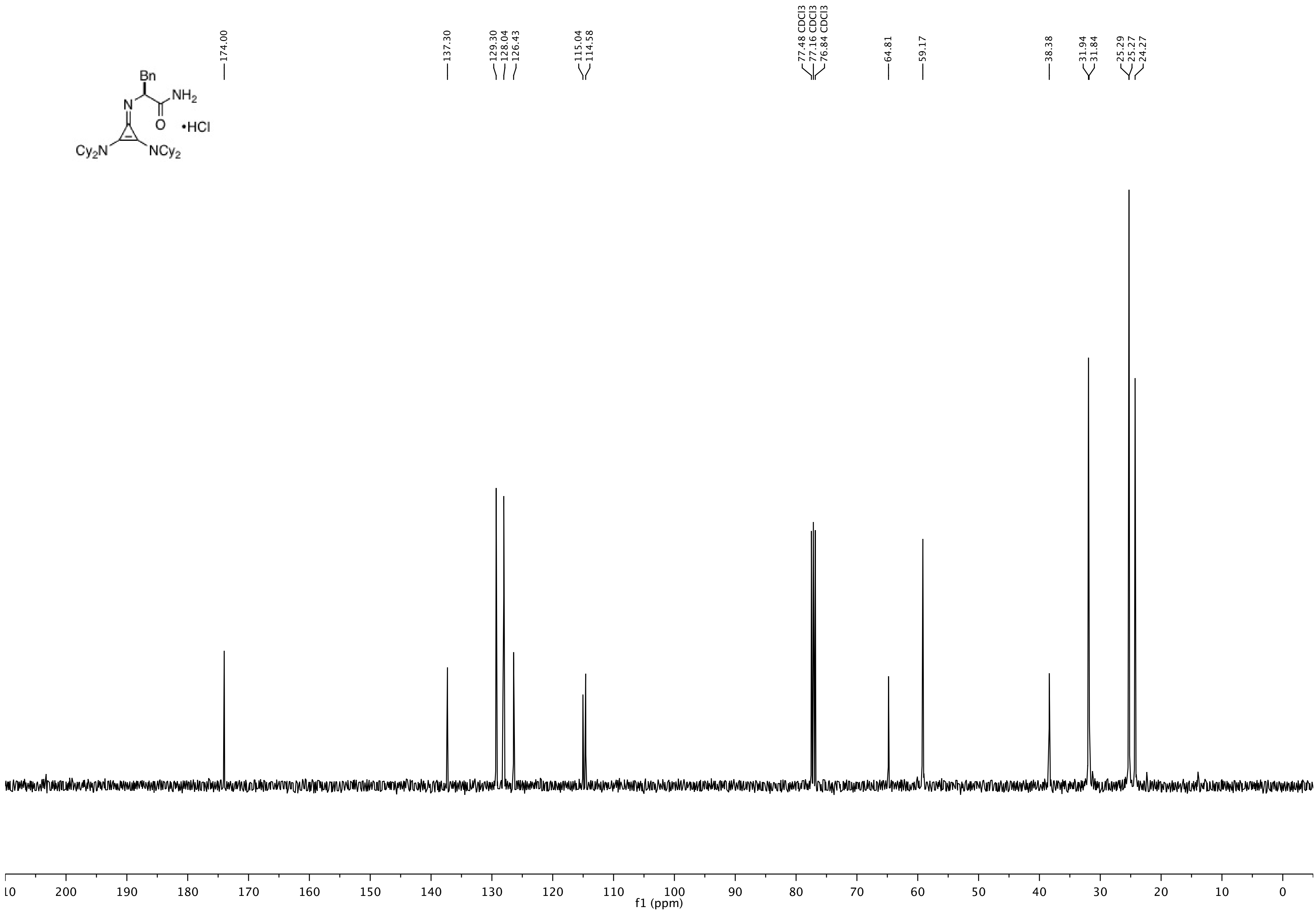

Bn
NH2
N
O
•HCl
Cy2N
NCy2
174.00
137.30
129.30
128.04
126.43
115.04
114.58
77.48 CDCl3
77.16 CDCl3
76.84 CDCl3
64.81
59.17
38.38
31.94
31.84
25.29
25.27
24.27
10
200
190
180
170
160
150
140
130
120
110
100
90
80
70
60
50
40
30
20
10
0
f1 (ppm)

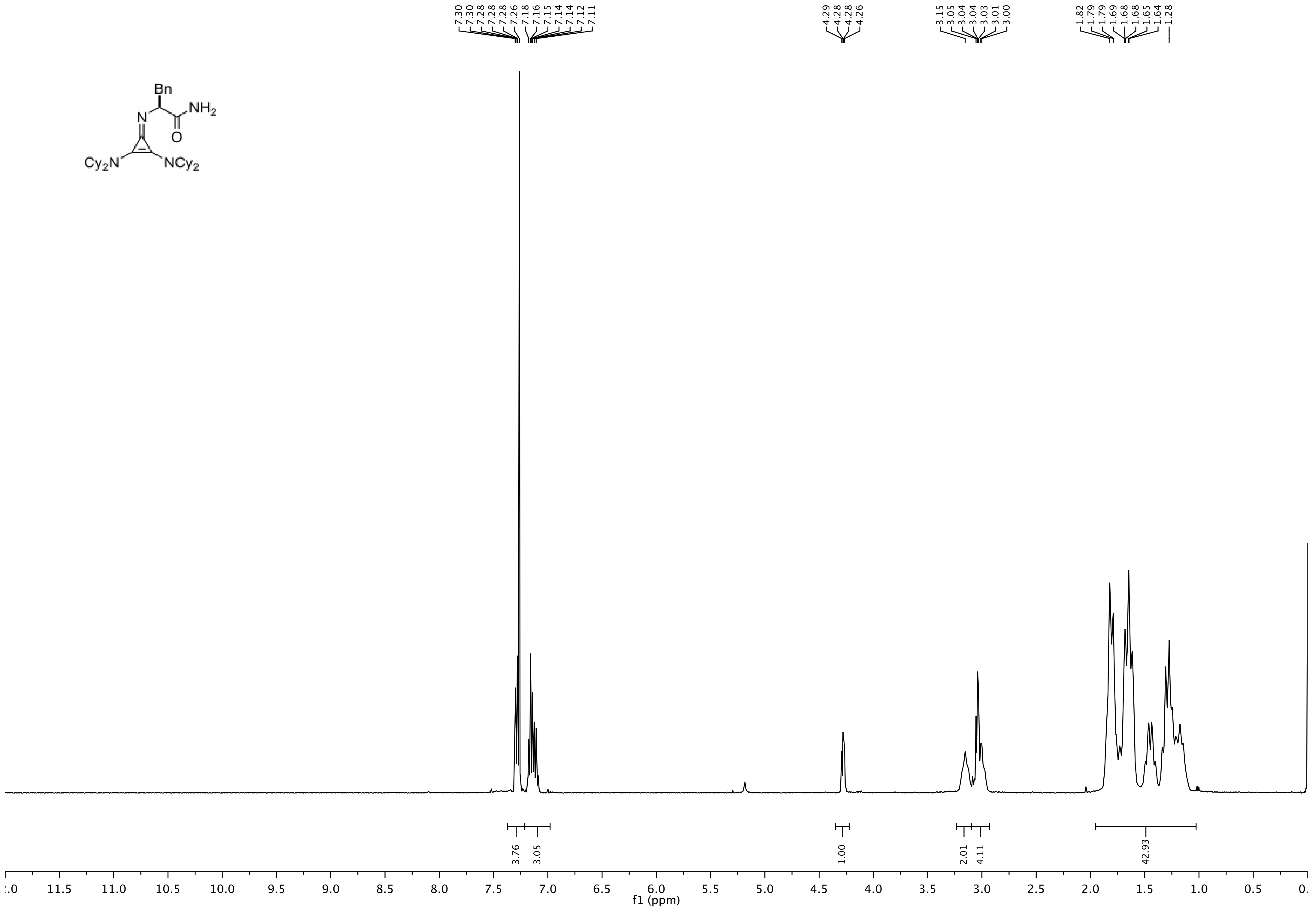
Bn
NH2
N
O
Cy2N
NCy2
7.30
7.30
7.28
7.28
7.28
7.26
7.18
7.16
7.15
7.14
7.14
7.12
7.11
4.29
4.28
4.28
4.26
3.15
3.05
3.04
3.04
3.03
3.01
3.00
1.82
1.79
1.79
1.69
1.68
1.68
1.65
1.64
1.28
3.76
3.05
1.00
2.01
4.11
42.93
11.5
11.0
10.5
10.0
9.5
9.0
8.5
8.0
7.5
7.0
6.5
6.0
5.5
5.0
4.5
4.0
3.5
3.0
2.5
2.0
1.5
1.0
0.5
f1 (ppm)

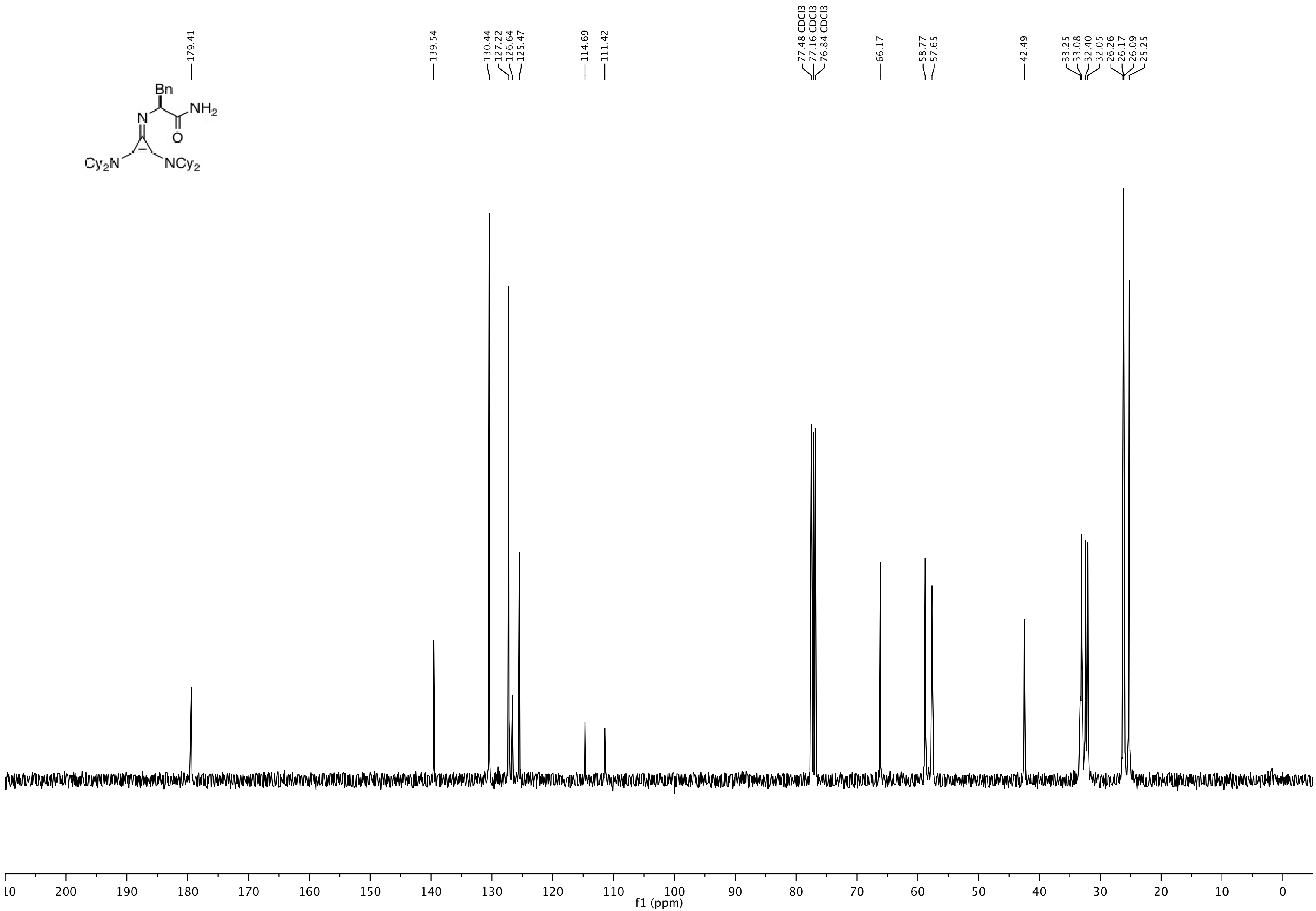

179.41
139.54
130.44
127.22
126.64
125.47
114.69
111.42
77.48 CDCl3
77.16 CDCl3
76.84 CDCl3
66.17
58.77
57.65
42.49
33.25
33.08
32.40
32.05
26.26
26.17
26.09
25.25
Bn
N
NH2
O
Cy2N
NCy2
200
190
180
170
160
150
140
130
120
110
100
90
80
70
60
50
40
30
20
10
0
f1 (ppm)

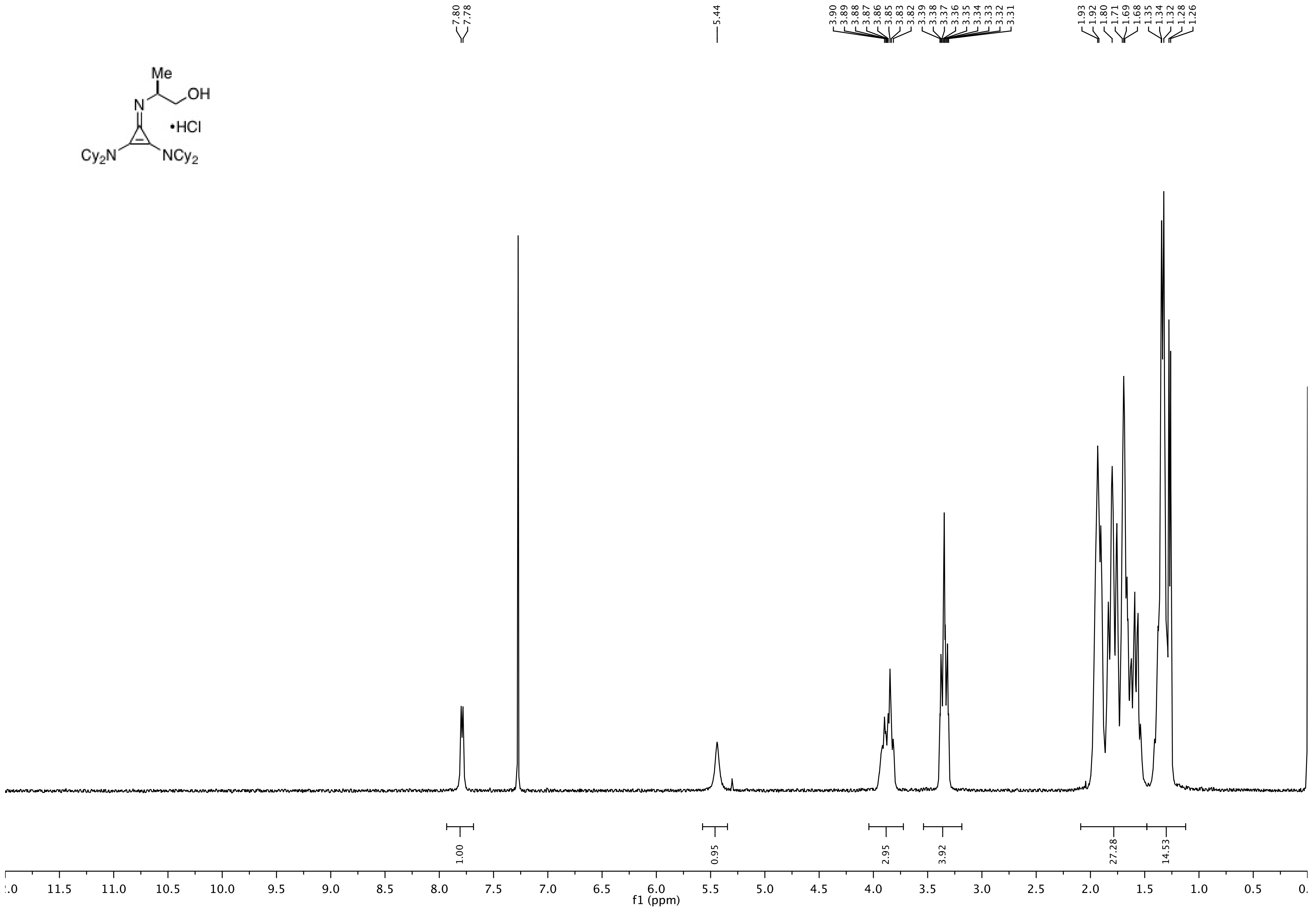
7.80
7.78
5.44
3.90
3.89
3.88
3.87
3.86
3.85
3.83
3.82
3.39
3.38
3.37
3.36
3.35
3.34
3.33
3.32
3.31
1.93
1.92
1.80
1.71
1.69
1.68
1.35
1.34
1.32
1.28
1.26
Me
N
OH
•HCl
Cy2N
NCy2
1.00
0.95
2.95
3.92
27.28
14.53
11.5
11.0
10.5
10.0
9.5
9.0
8.5
8.0
7.5
7.0
6.5
6.0
5.5
5.0
4.5
4.0
3.5
3.0
2.5
2.0
1.5
1.0
0.5
f1 (ppm)

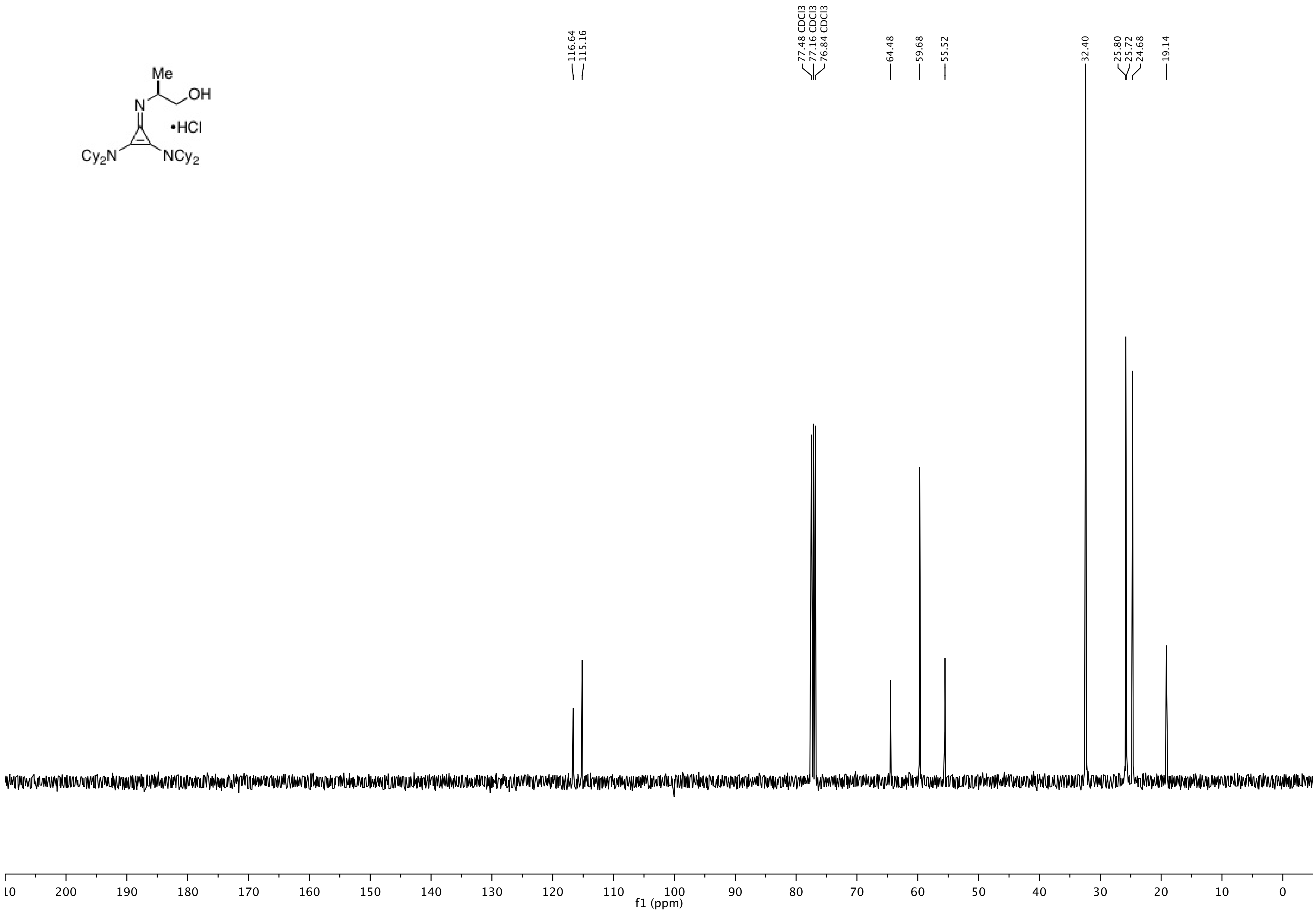
Me
N
OH
•HCl
Cy_2N
NCy_2
116.64
115.16
77.48 CDCl3
77.16 CDCl3
76.84 CDCl3
64.48
59.68
55.52
32.40
25.80
25.72
24.68
19.14
10
200
190
180
170
160
150
140
130
120
110
100
90
80
70
60
50
40
30
20
10
0
f1 (ppm)

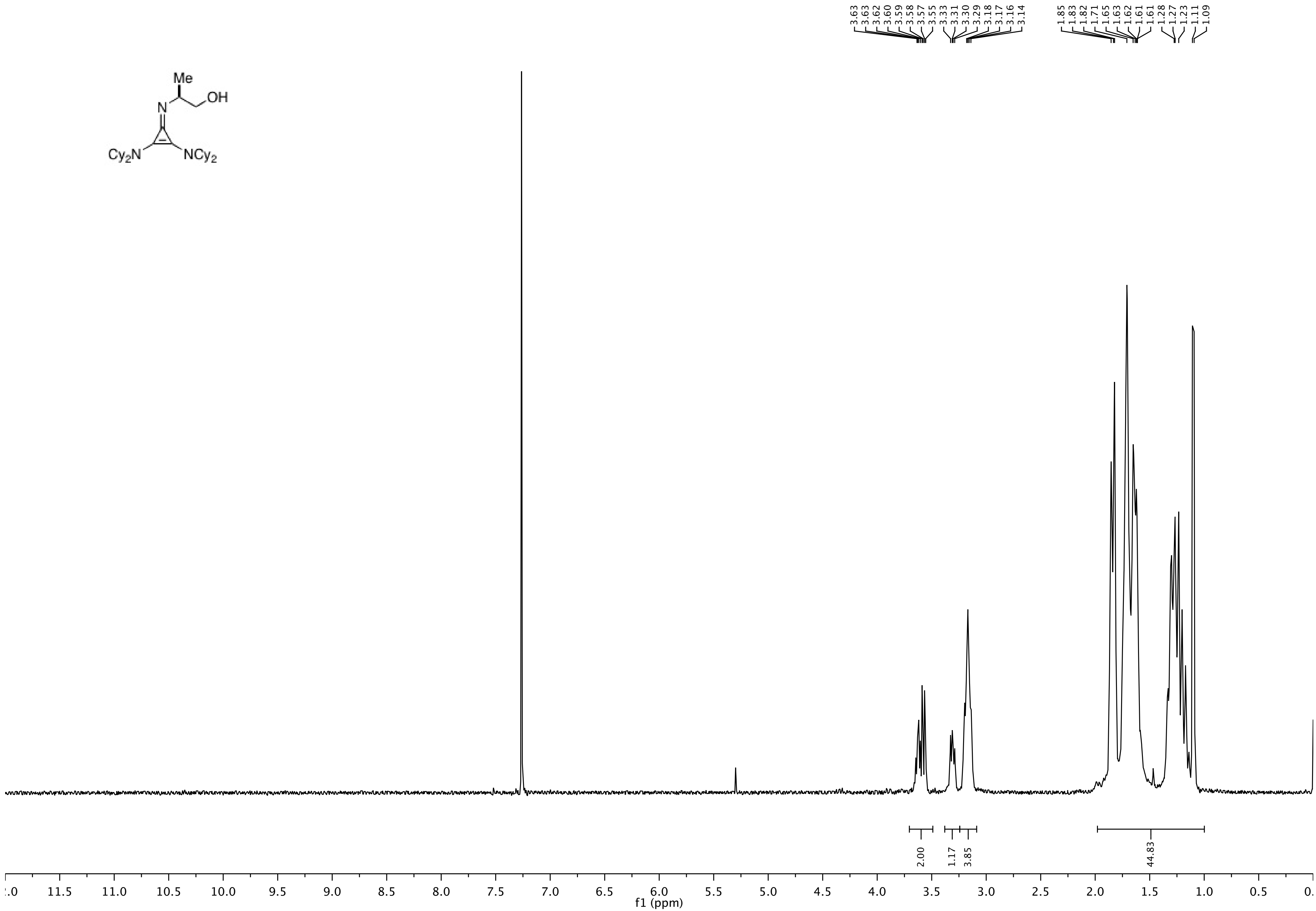
Me
OH
N
Cy2N
NCy2
3.63 3.63 3.62 3.60 3.59 3.58 3.57 3.55 3.33 3.31 3.30 3.29 3.18 3.17 3.16 3.14
1.85 1.83 1.82 1.71 1.65 1.63 1.62 1.61 1.61 1.28 1.27 1.23 1.11 1.09
2.00
1.17
3.85
44.83
11.5 11.0 10.5 10.0 9.5 9.0 8.5 8.0 7.5 7.0 6.5 6.0 5.5 5.0 4.5 4.0 3.5 3.0 2.5 2.0 1.5 1.0 0.5
f1 (ppm)

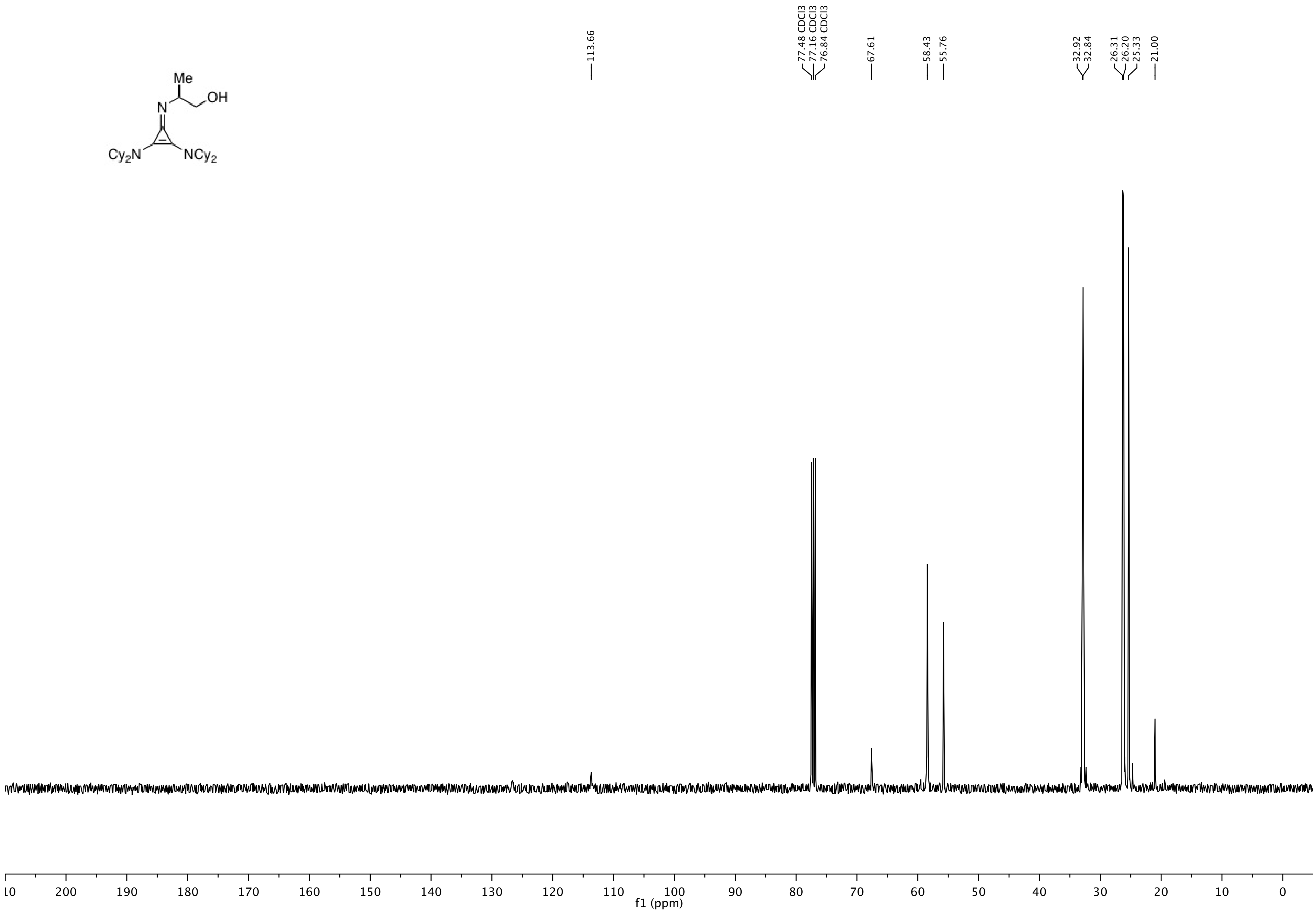

113.66
77.48 CDCl3
77.16 CDCl3
76.84 CDCl3
67.61
58.43
55.76
32.92
32.84
26.31
26.20
25.33
21.00
Me
N
OH
Cy2N
NCy2
10
200
190
180
170
160
150
140
130
120
110
100
90
80
70
60
50
40
30
20
10
0
f1 (ppm)

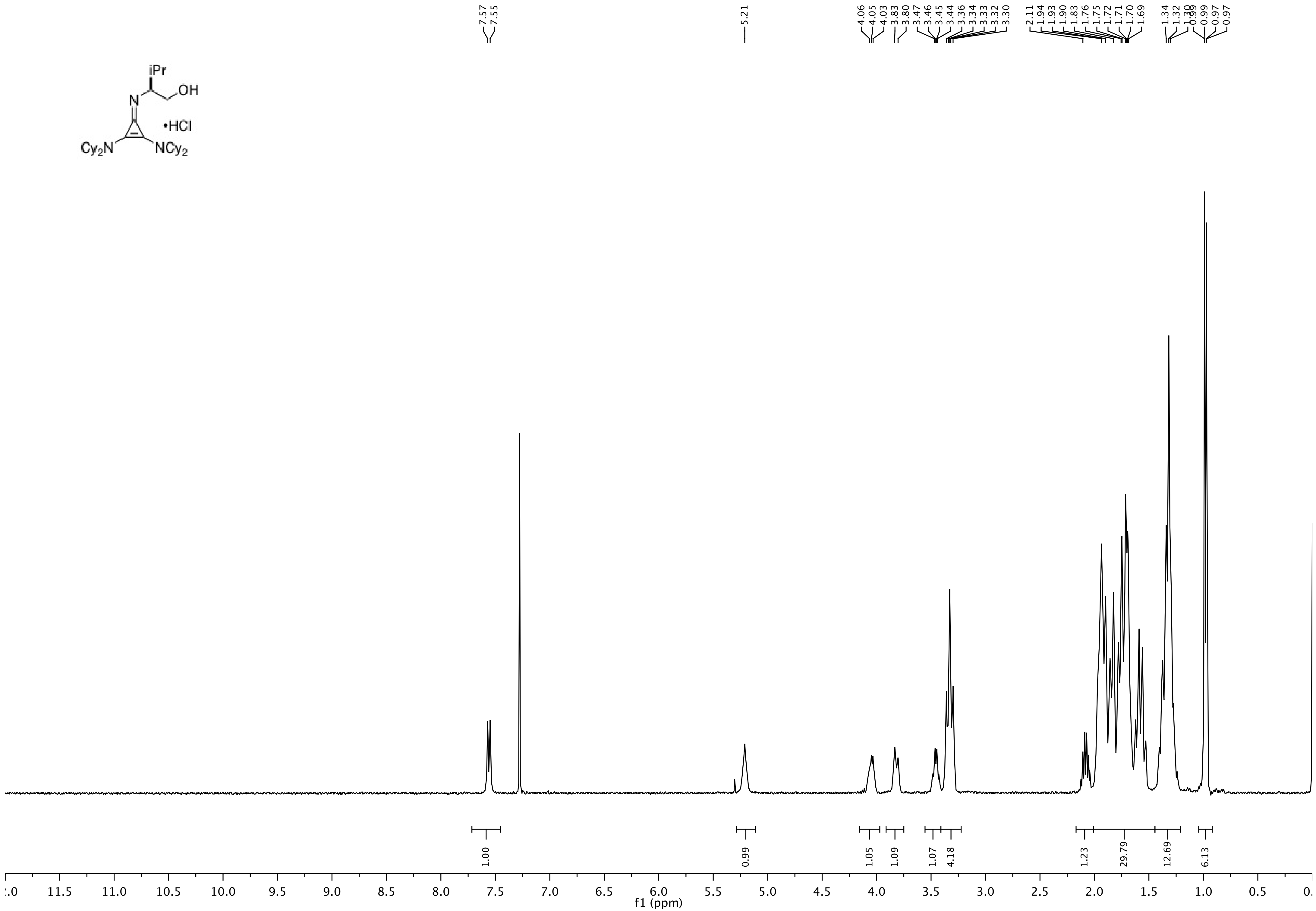

iPr
N
OH
•HCl
Cy2N
NCy2
7.57
7.55
5.21
4.06
4.05
4.03
3.83
3.80
3.47
3.46
3.45
3.44
3.36
3.34
3.33
3.32
3.30
2.11
1.94
1.93
1.90
1.83
1.76
1.75
1.72
1.71
1.70
1.69
1.34
1.32
0.99
0.97
1.00
0.99
1.05
1.09
1.07
4.18
1.23
29.79
12.69
6.13
11.5
11.0
10.5
10.0
9.5
9.0
8.5
8.0
7.5
7.0
6.5
6.0
5.5
5.0
4.5
4.0
3.5
3.0
2.5
2.0
1.5
1.0
0.5
f1 (ppm)

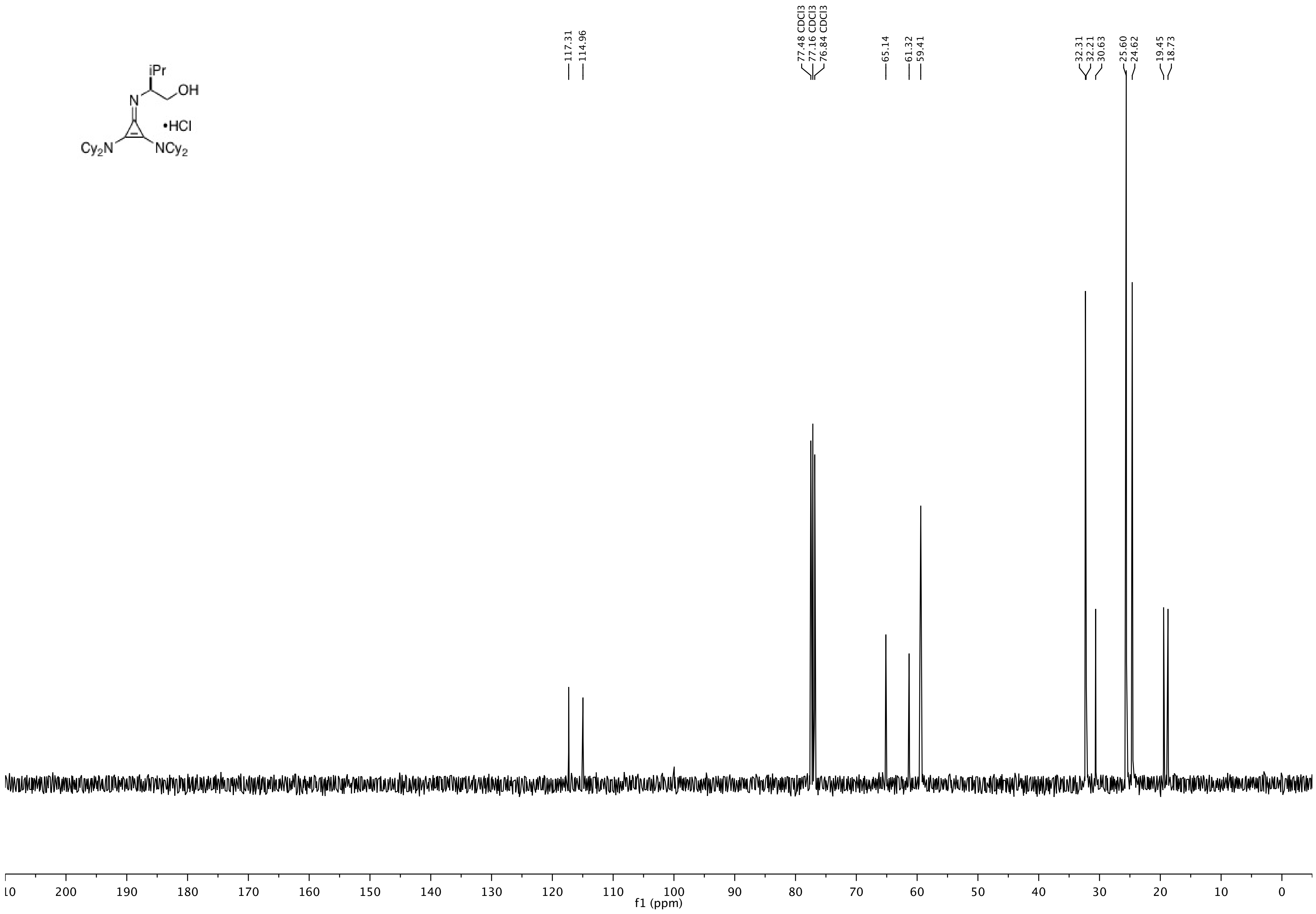

iPr
N
OH
•HCl
Cy2N
NCy2
117.31
114.96
77.48 CDCl3
77.16 CDCl3
76.84 CDCl3
65.14
61.32
59.41
32.31
32.21
30.63
25.60
24.62
19.45
18.73
200 190 180 170 160 150 140 130 120 110 100 90 80 70 60 50 40 30 20 10 0
f1 (ppm)

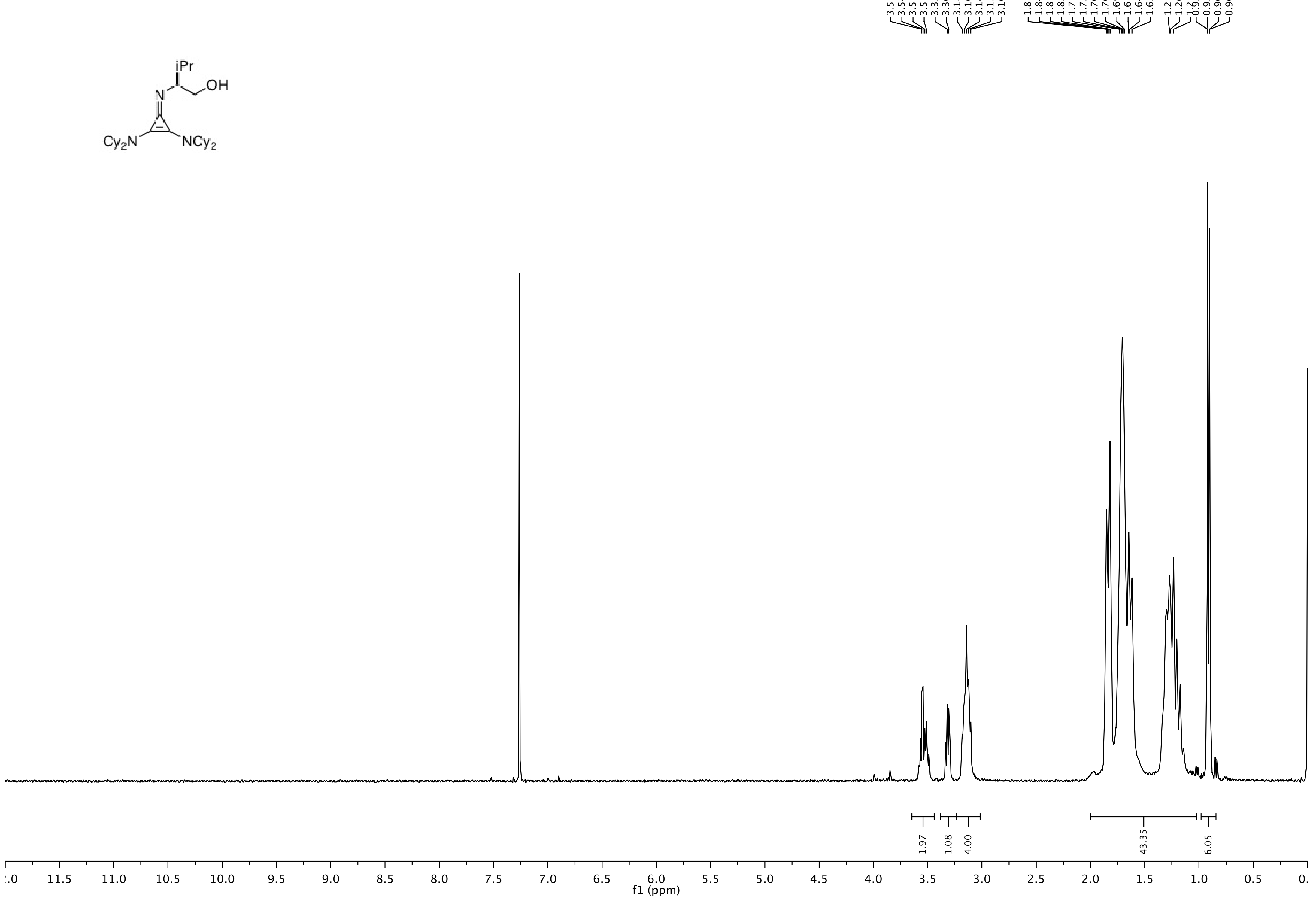

iPr
OH
N
Cy2N
NCy2
3.55 3.54 3.53 3.51 3.32 3.30 3.18 3.16 3.14 3.12 3.10
1.85 1.84 1.83 1.82 1.73 1.72 1.70 1.69 1.65 1.64 1.62
1.27 1.26 1.23 0.92 0.92 0.90 0.90
1.97
1.08
4.00
43.35
6.05
:.0 11.5 11.0 10.5 10.0 9.5 9.0 8.5 8.0 7.5 7.0 6.5 6.0 5.5 5.0 4.5 4.0 3.5 3.0 2.5 2.0 1.5 1.0 0.5 0.
f1 (ppm)

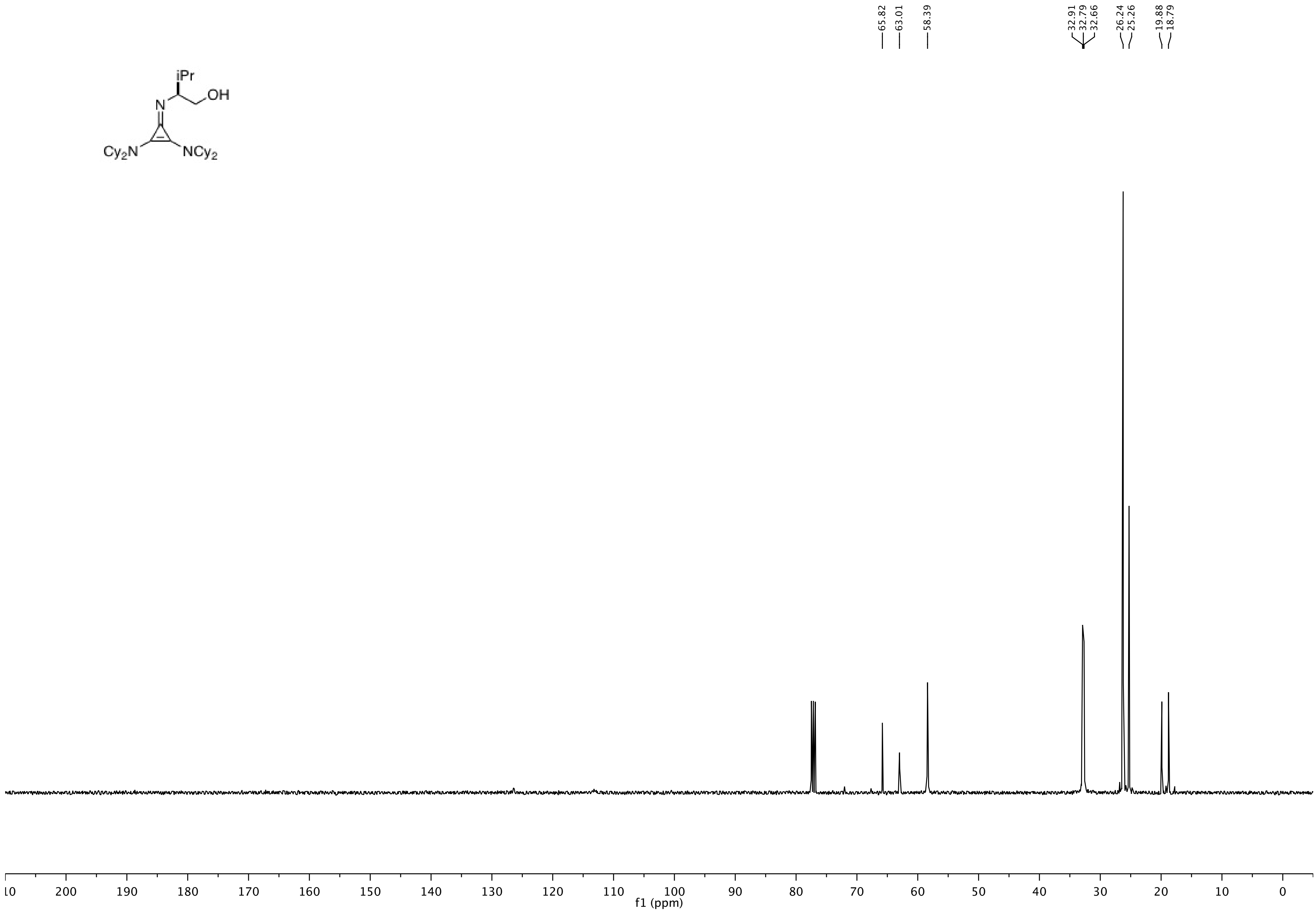

65.82
63.01
58.39
32.91
32.79
32.66
26.24
25.26
19.88
18.79
iPr
N
OH
Cy2N
NCy2
10
200
190
180
170
160
150
140
130
120
110
100
90
80
70
60
50
40
30
20
10
0
f1 (ppm)

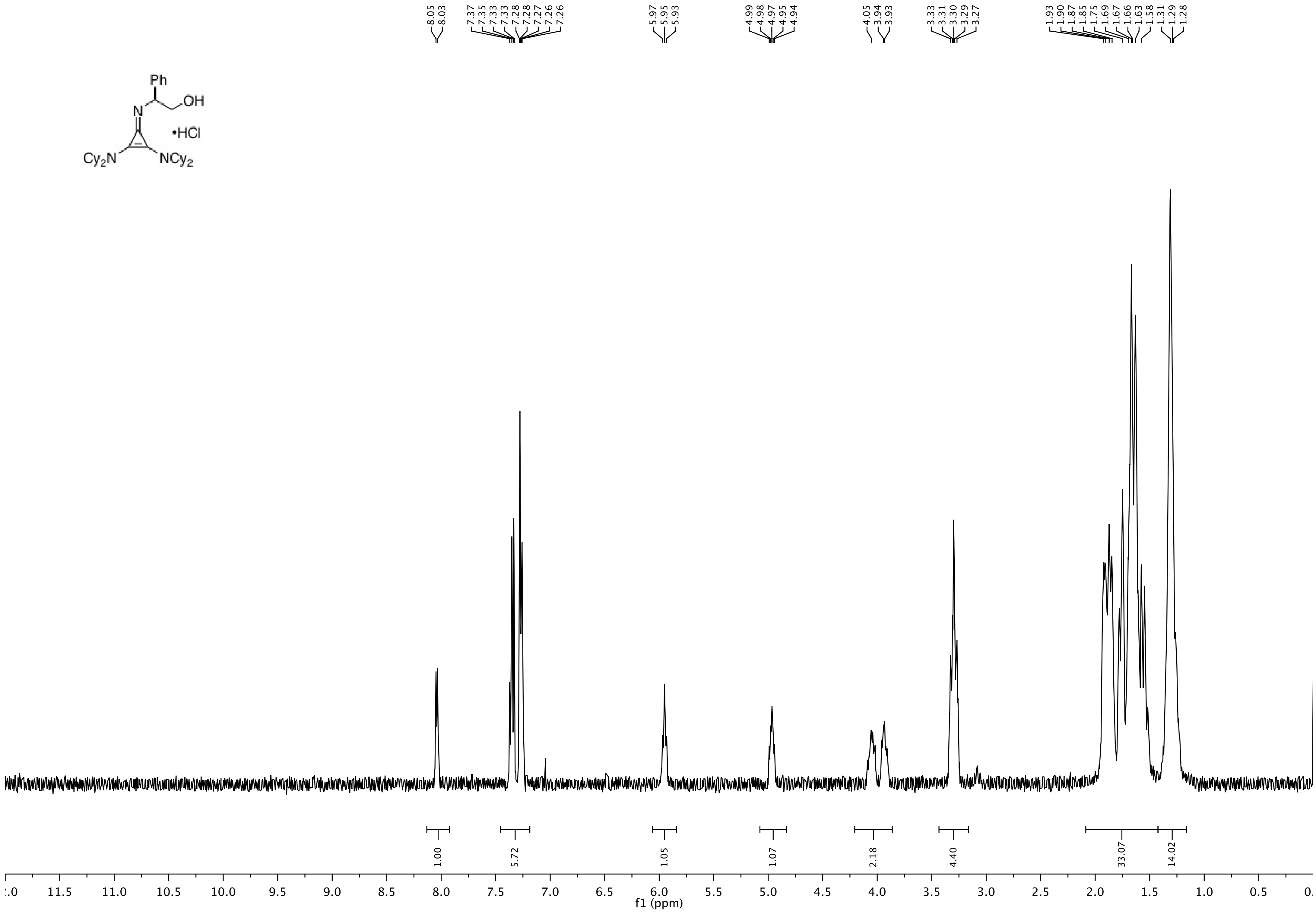

Ph
OH
N
•HCl
Cy2N
NCy2
8.05
8.03
7.37
7.35
7.33
7.33
7.28
7.28
7.27
7.26
7.26
5.97
5.95
5.93
4.99
4.98
4.97
4.95
4.94
4.05
3.94
3.93
3.33
3.31
3.30
3.29
3.27
1.93
1.90
1.87
1.85
1.75
1.69
1.67
1.66
1.63
1.58
1.31
1.29
1.28
1.00
5.72
1.05
1.07
2.18
4.40
33.07
14.02
11.5
11.0
10.5
10.0
9.5
9.0
8.5
8.0
7.5
7.0
6.5
6.0
5.5
5.0
4.5
4.0
3.5
3.0
2.5
2.0
1.5
1.0
0.5
f1 (ppm)

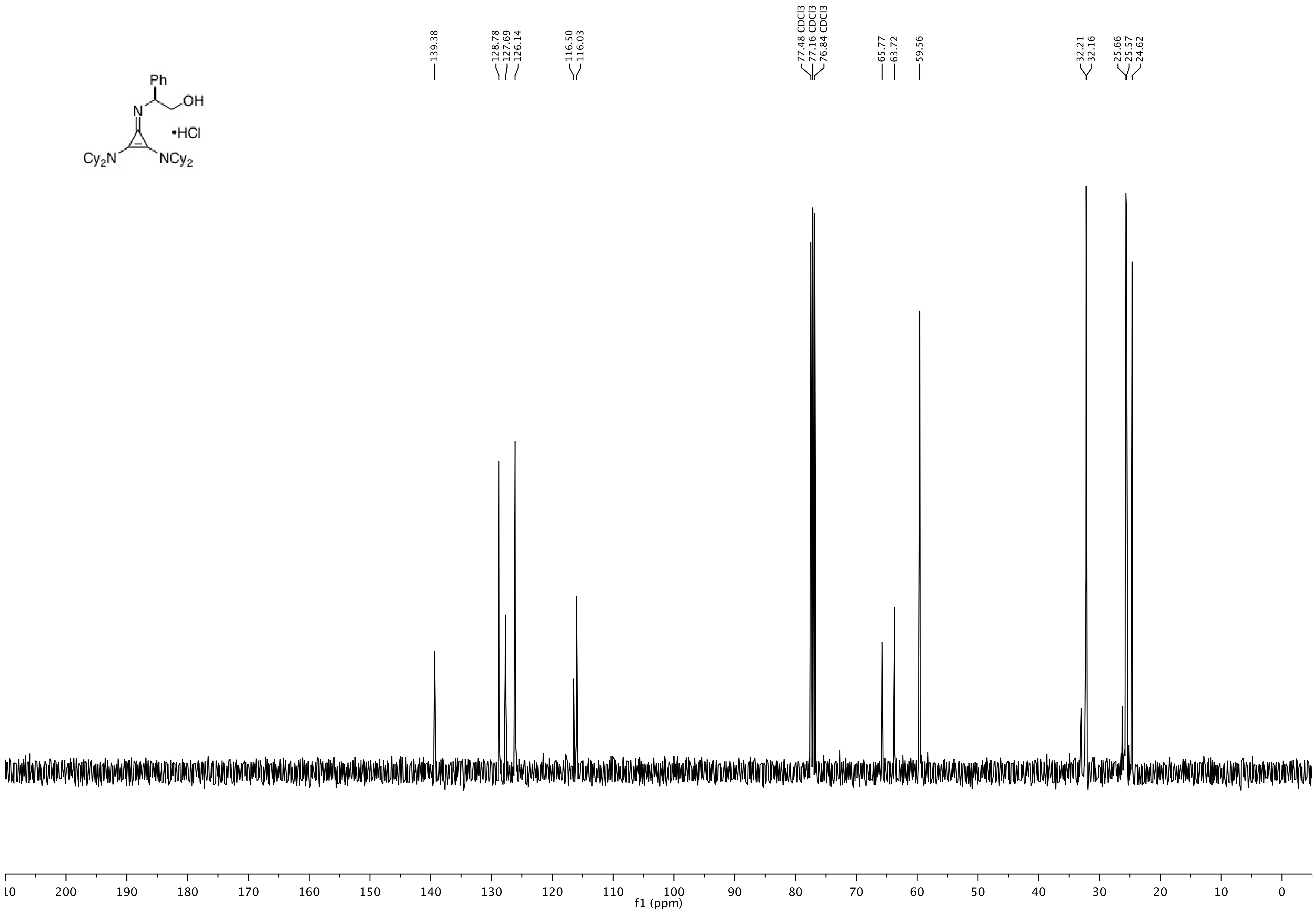

Ph
OH
N
•HCl
Cy2N
NCy2
139.38
128.78
127.69
126.14
116.50
116.03
77.48 CDCl3
77.16 CDCl3
76.84 CDCl3
65.77
63.72
59.56
32.21
32.16
25.66
25.57
24.62
10
200
190
180
170
160
150
140
130
120
110
100
90
80
70
60
50
40
30
20
10
0
f1 (ppm)

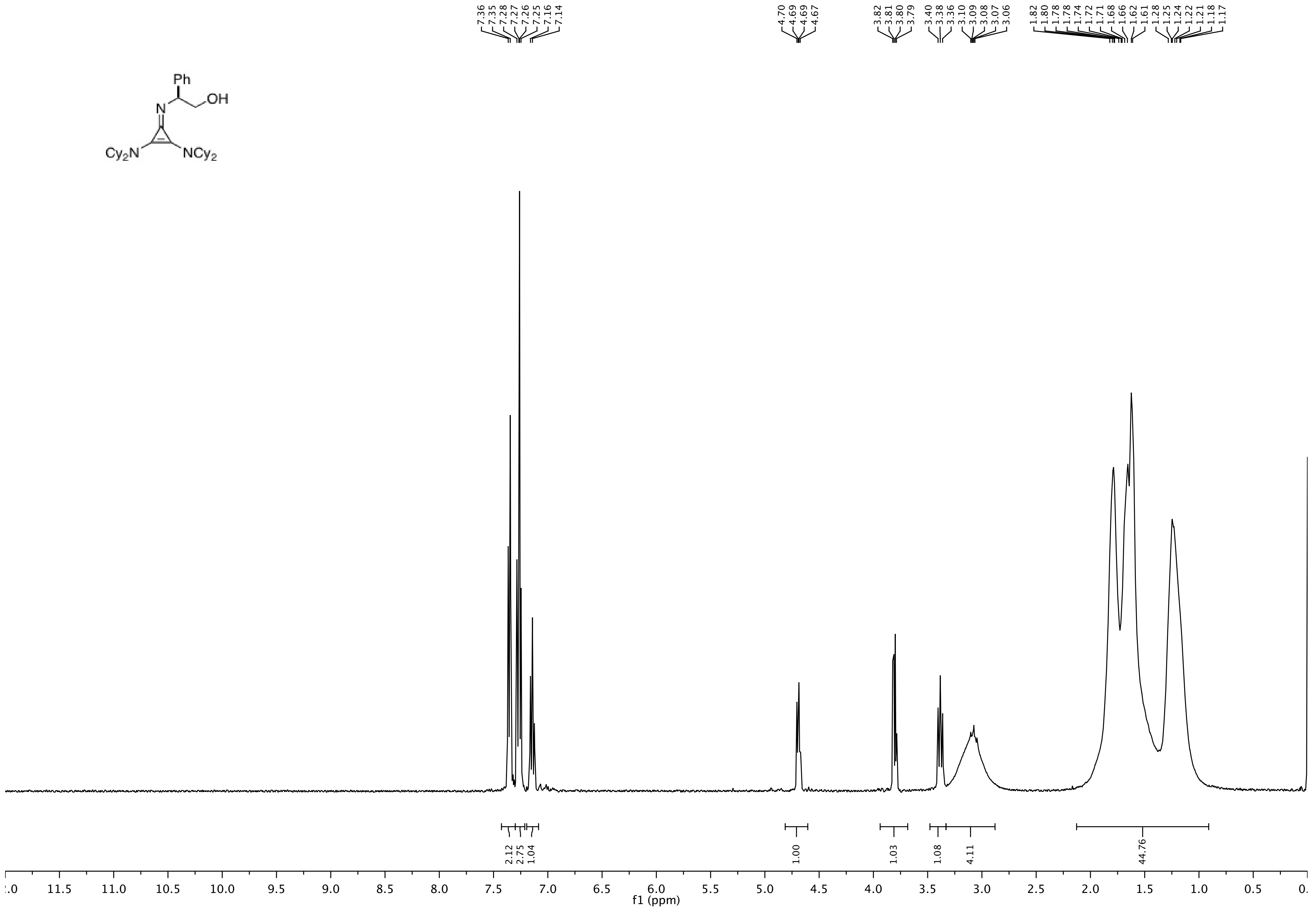

Ph
OH
N
Cy2N
NCy2
7.36
7.35
7.28
7.27
7.26
7.25
7.16
7.14
4.70
4.69
4.69
4.67
3.82
3.81
3.80
3.79
3.40
3.38
3.36
3.10
3.09
3.08
3.07
3.06
1.82
1.80
1.78
1.78
1.74
1.72
1.71
1.68
1.66
1.62
1.61
1.28
1.25
1.24
1.22
1.21
1.18
1.17
2.12
2.75
1.04
1.00
1.03
1.08
4.11
44.76
11.5
11.0
10.5
10.0
9.5
9.0
8.5
8.0
7.5
7.0
6.5
6.0
5.5
5.0
4.5
4.0
3.5
3.0
2.5
2.0
1.5
1.0
0.5
f1 (ppm)

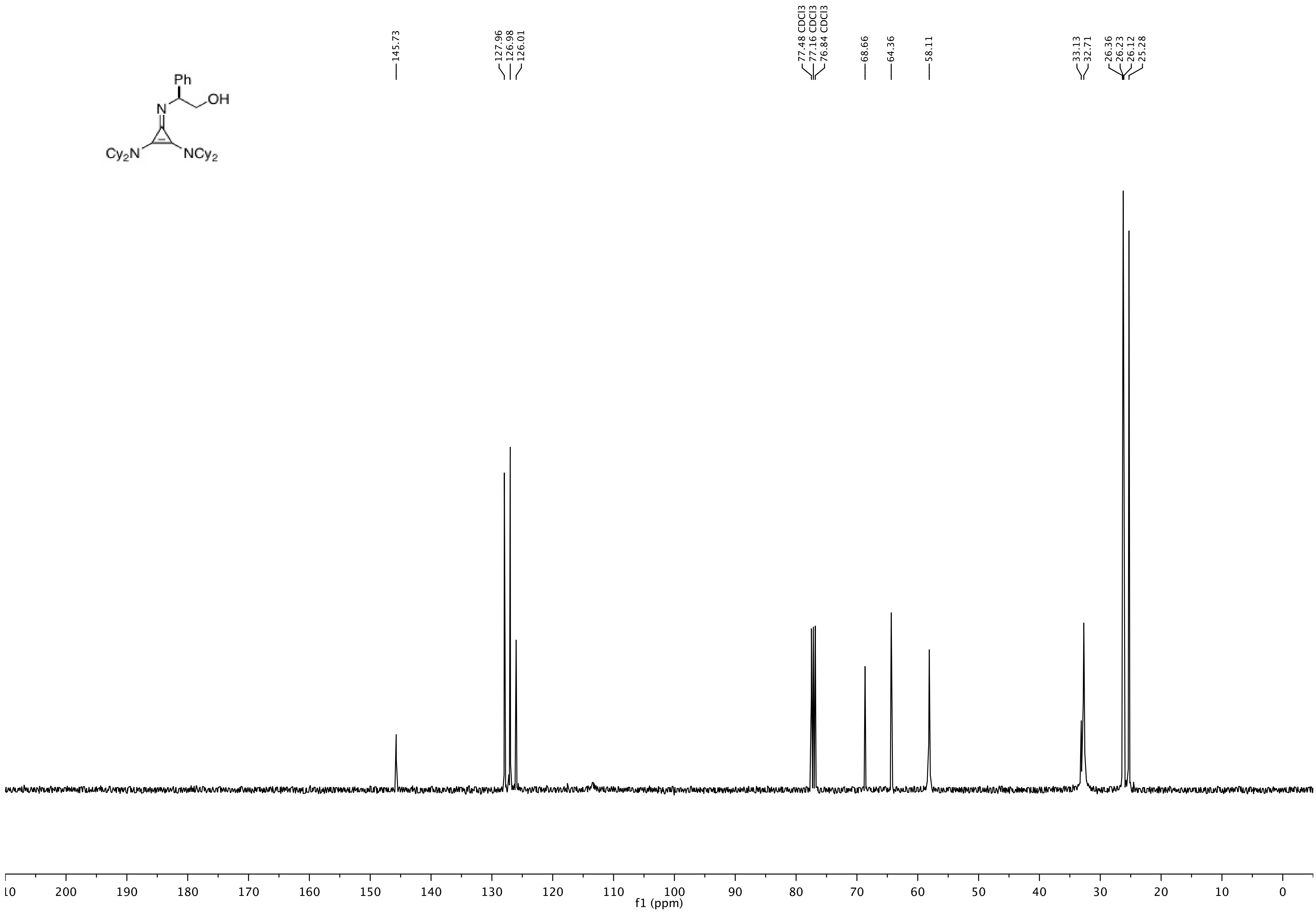

Ph
N
OH
Cy2N
NCy2
145.73
127.96
126.98
126.01
77.48 CDCl3
77.16 CDCl3
76.84 CDCl3
68.66
64.36
58.11
33.13
32.71
26.36
26.23
26.12
25.28
200
190
180
170
160
150
140
130
120
110
100
90
80
70
60
50
40
30
20
10
0
f1 (ppm)

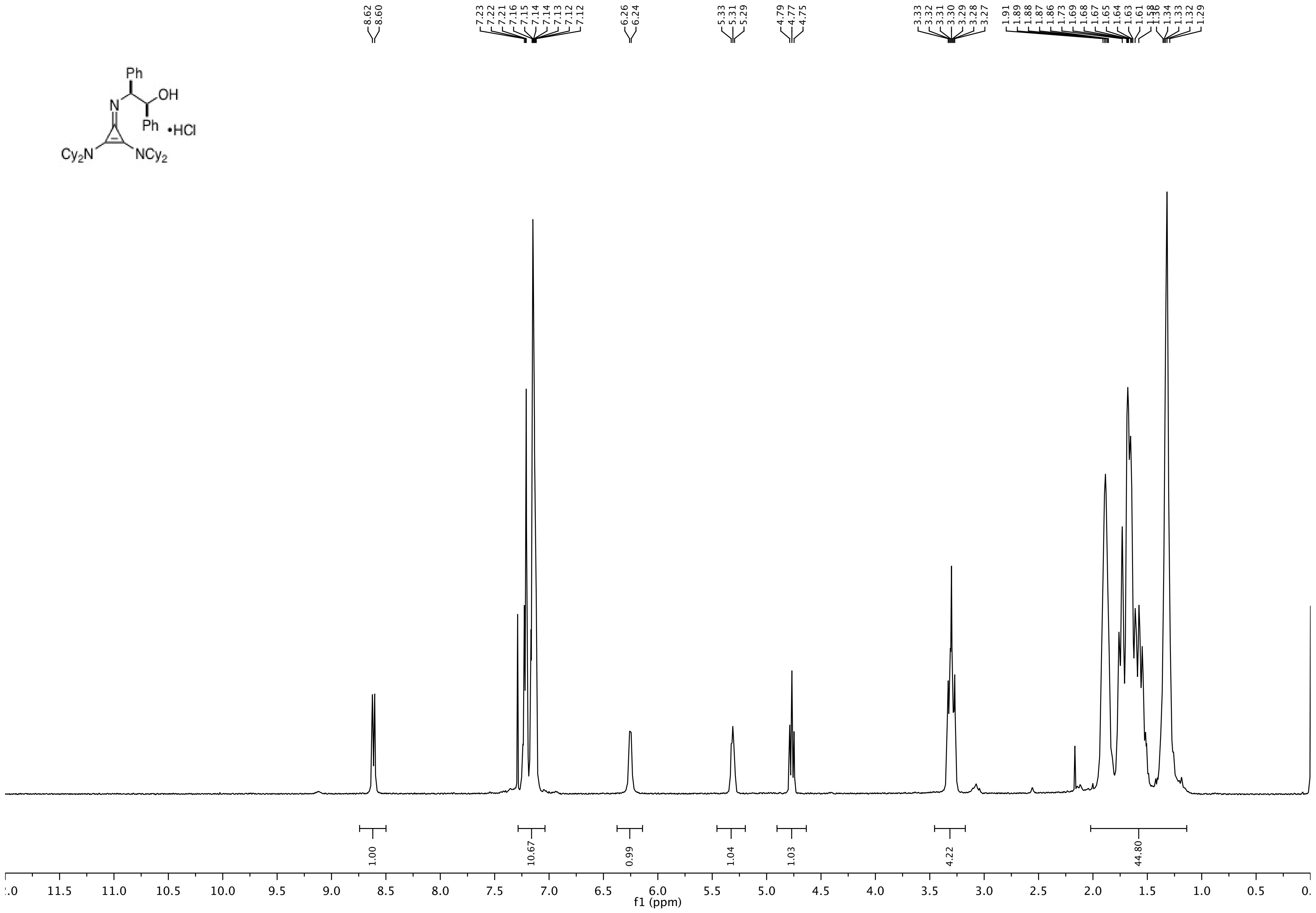

8.62
8.60
7.23
7.22
7.21
7.16
7.15
7.14
7.14
7.13
7.12
7.12
6.26
6.24
5.33
5.31
5.29
4.79
4.77
4.75
3.33
3.32
3.31
3.30
3.29
3.28
3.27
1.91
1.89
1.88
1.87
1.86
1.73
1.69
1.68
1.67
1.65
1.64
1.63
1.61
1.58
1.36
1.34
1.33
1.32
1.29
Ph
OH
N
Ph
•HCl
Cy_2N
NCy_2
1.00
10.67
0.99
1.04
1.03
4.22
44.80
11.5
11.0
10.5
10.0
9.5
9.0
8.5
8.0
7.5
7.0
6.5
6.0
5.5
5.0
4.5
4.0
3.5
3.0
2.5
2.0
1.5
1.0
0.5
f1 (ppm)

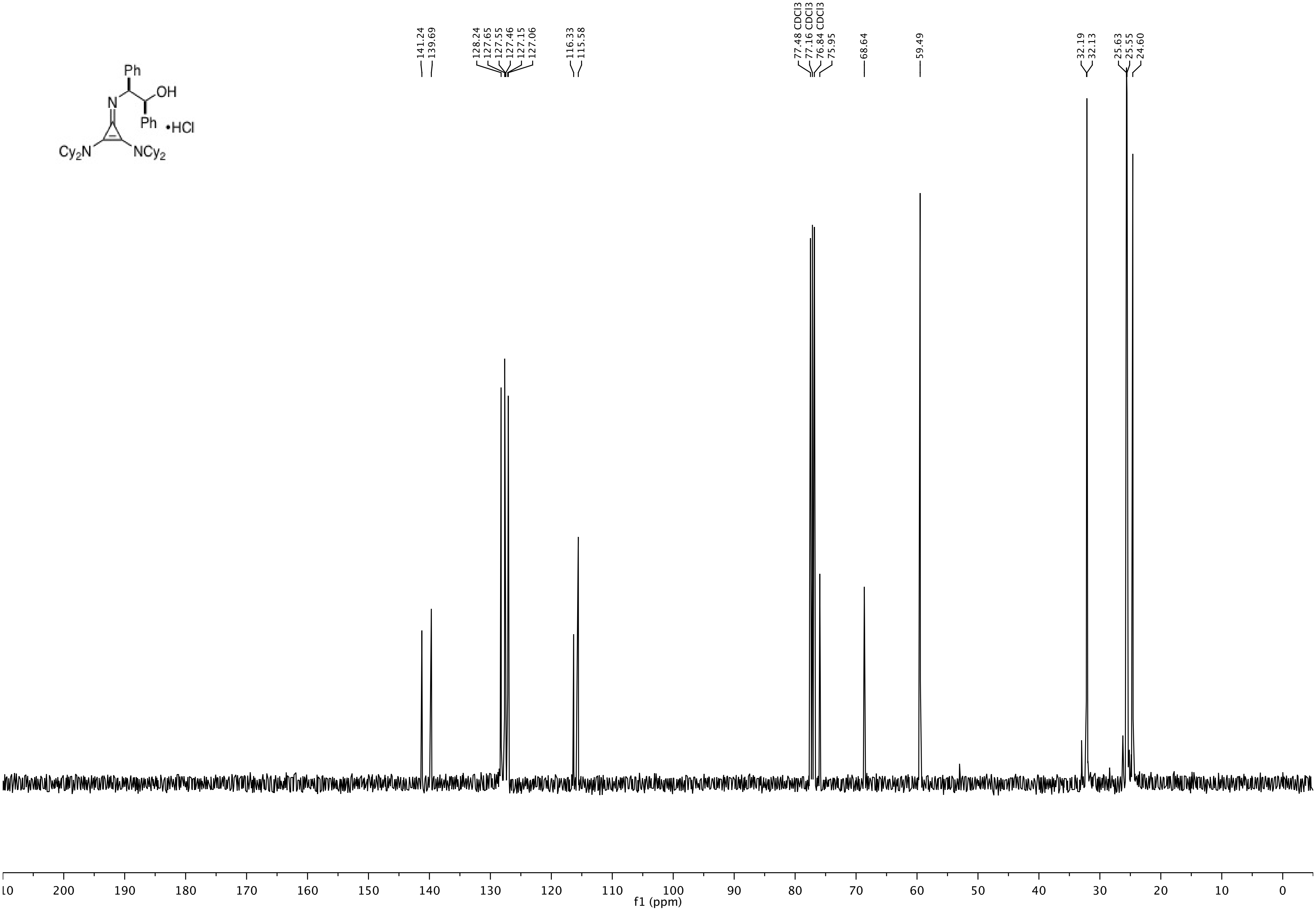
Ph
N
OH
Ph
•HCl
Cy2N
NCy2
141.24
139.69
128.24
127.65
127.55
127.46
127.15
127.06
116.33
115.58
77.48 CDCl3
77.16 CDCl3
76.84 CDCl3
75.95
68.64
59.49
32.19
32.13
25.63
25.55
24.60
10
200
190
180
170
160
150
140
130
120
110
100
90
80
70
60
50
40
30
20
10
0
f1 (ppm)

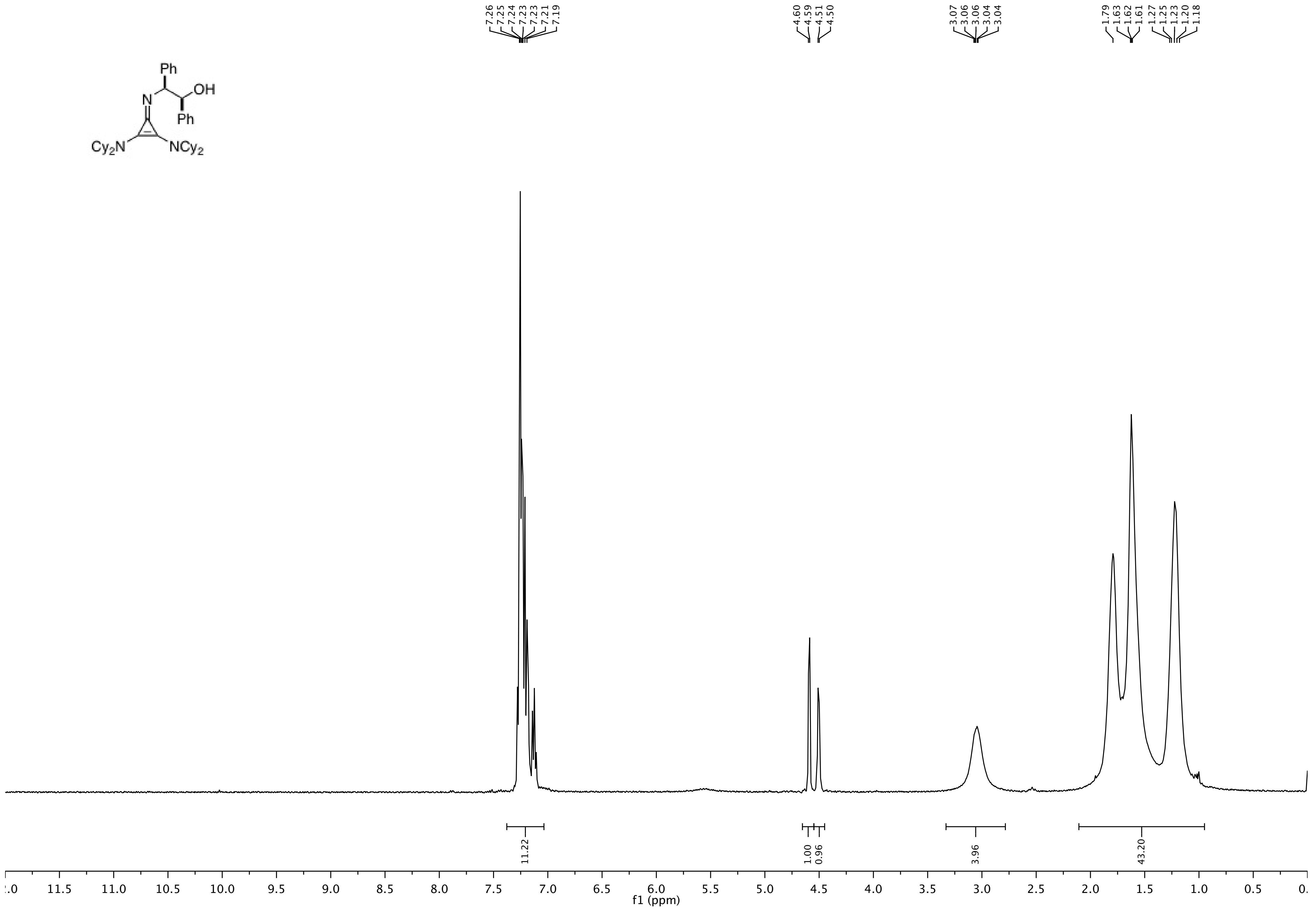
Ph
OH
N
Ph
Cy2N
NCy2
7.26 7.25 7.24 7.23 7.23 7.21 7.19
4.60 4.59 4.51 4.50
3.07 3.06 3.06 3.04 3.04
1.79 1.63 1.62 1.61 1.27 1.25 1.23 1.20 1.18
11.22
1.00 0.96
3.96
43.20
11.5 11.0 10.5 10.0 9.5 9.0 8.5 8.0 7.5 7.0 6.5 6.0 5.5 5.0 4.5 4.0 3.5 3.0 2.5 2.0 1.5 1.0 0.5
f1 (ppm)

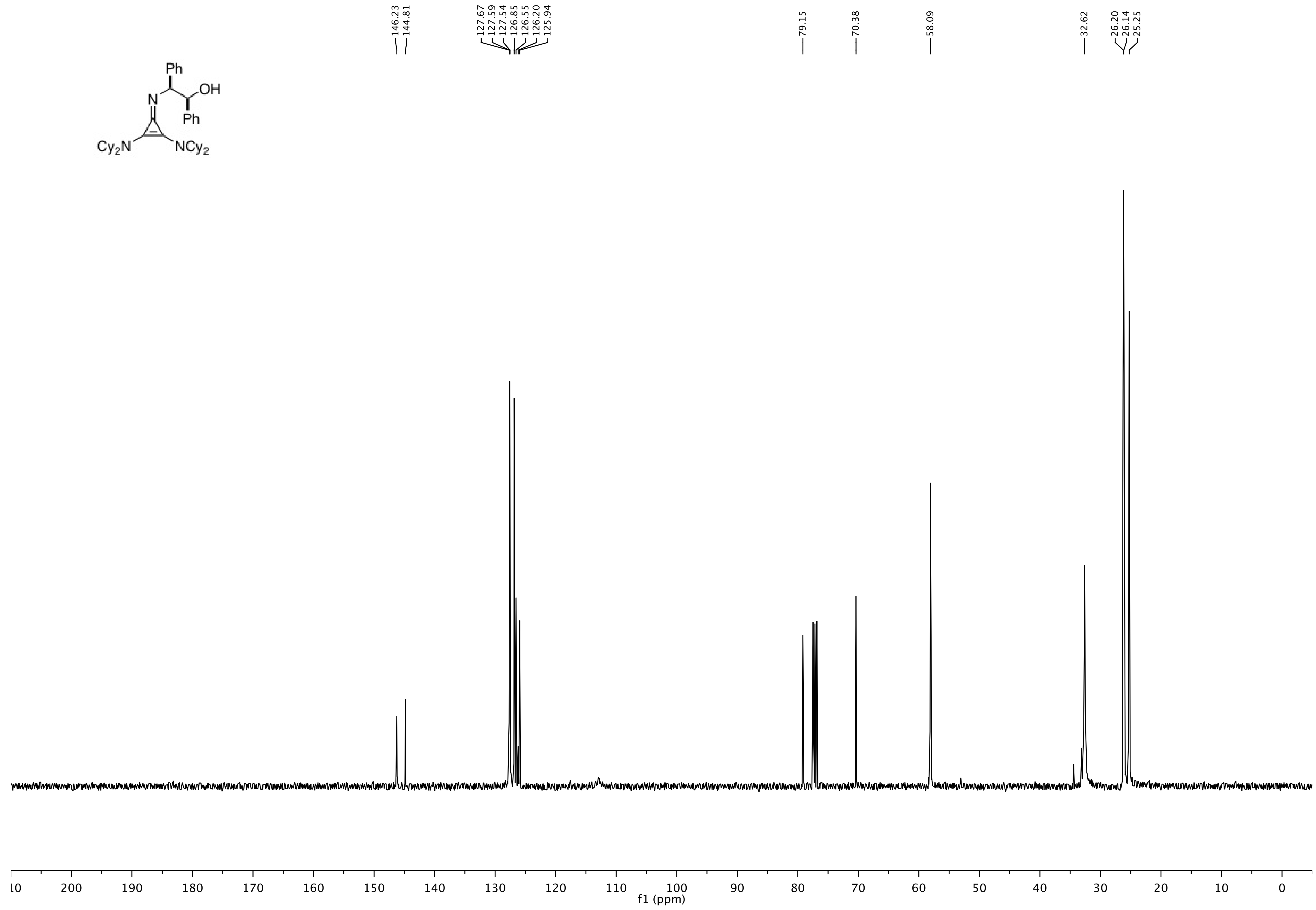

146.23
144.81
127.67
127.59
127.54
126.85
126.55
126.20
125.94
79.15
70.38
58.09
32.62
26.20
26.14
25.25
Ph
OH
N
Ph
Cy2N
NCy2
200
190
180
170
160
150
140
130
120
110
100
90
80
70
60
50
40
30
20
10
0
f1 (ppm)

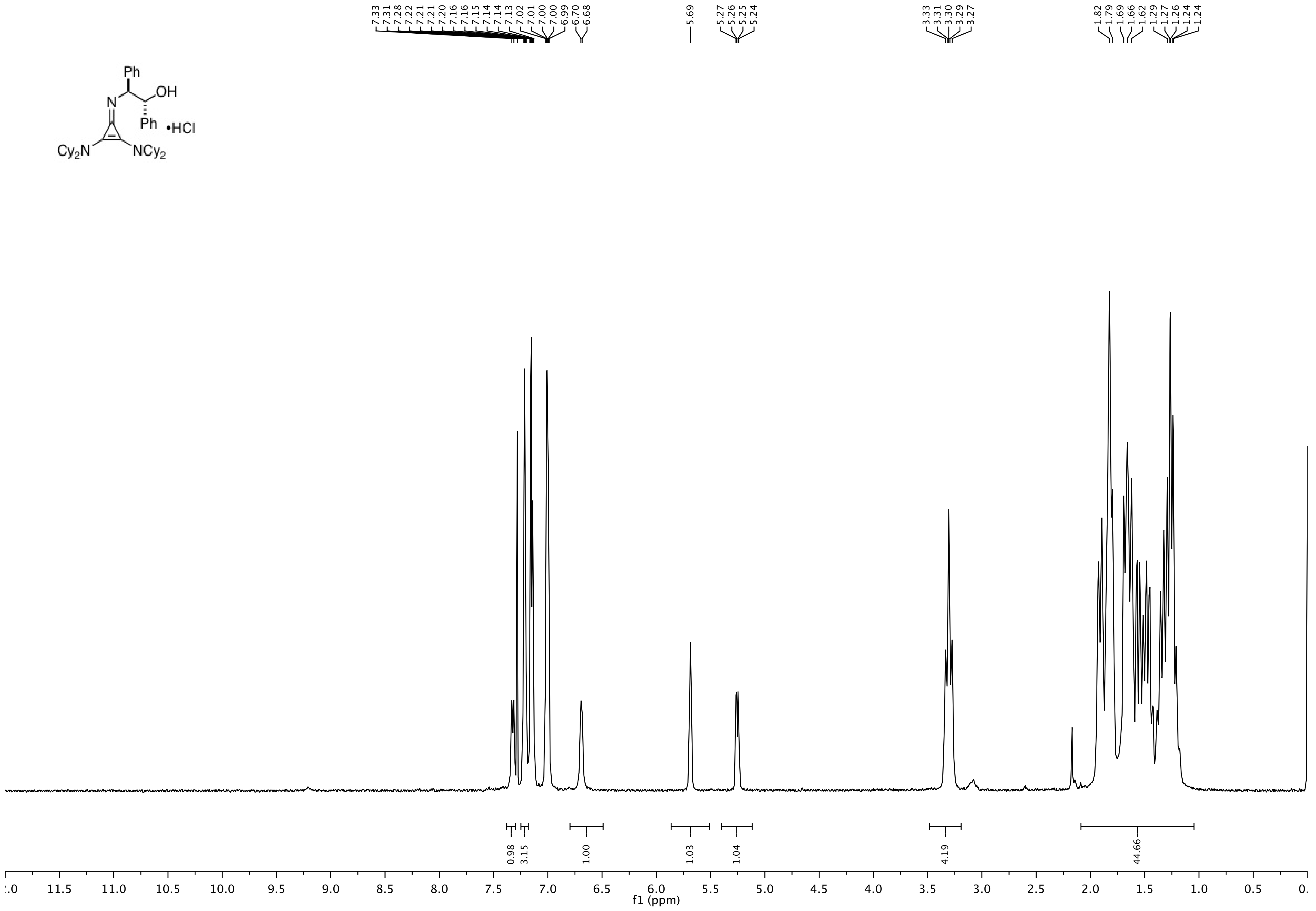
7.33 7.31 7.28 7.22 7.21 7.21 7.20 7.16 7.15 7.14 7.14 7.13 7.02 7.01 7.00 7.00 6.99 6.70 6.68
5.69
5.27 5.26 5.25 5.24
3.33 3.31 3.30 3.29 3.27
1.82 1.79 1.69 1.66 1.62 1.29 1.27 1.26 1.24 1.24
Ph
OH
N
Ph
•HCl
Cy2N
NCy2
0.98 3.15 1.00 1.03 1.04 4.19 44.66
11.5 11.0 10.5 10.0 9.5 9.0 8.5 8.0 7.5 7.0 6.5 6.0 5.5 5.0 4.5 4.0 3.5 3.0 2.5 2.0 1.5 1.0 0.5
f1 (ppm)

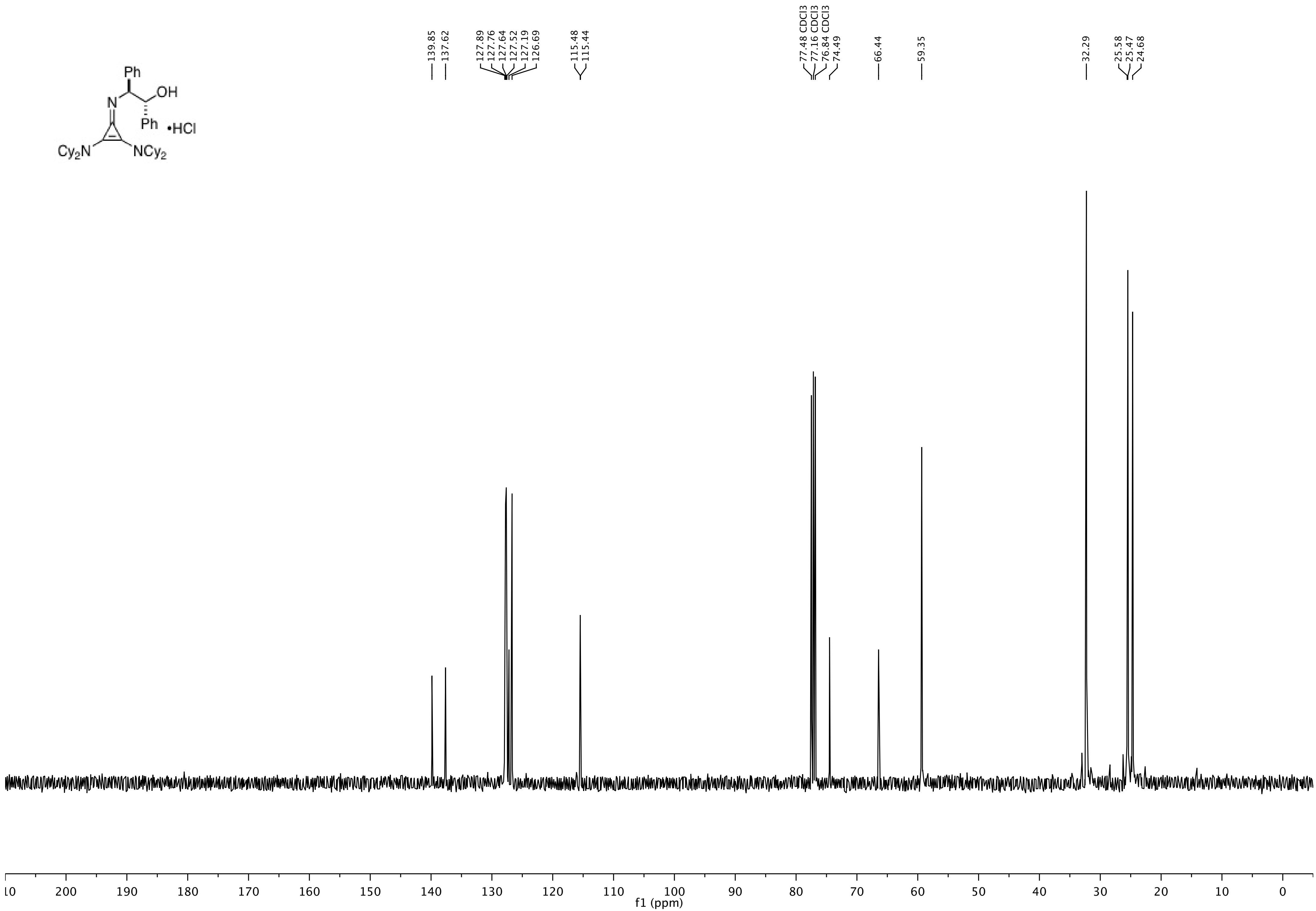

Ph
OH
N
Ph
•HCl
Cy2N
NCy2
139.85
137.62
127.89
127.76
127.64
127.52
127.19
126.69
115.48
115.44
77.48 CDCl3
77.16 CDCl3
76.84 CDCl3
74.49
66.44
59.35
32.29
25.58
25.47
24.68
10
200
190
180
170
160
150
140
130
120
110
100
90
80
70
60
50
40
30
20
10
0
f1 (ppm)

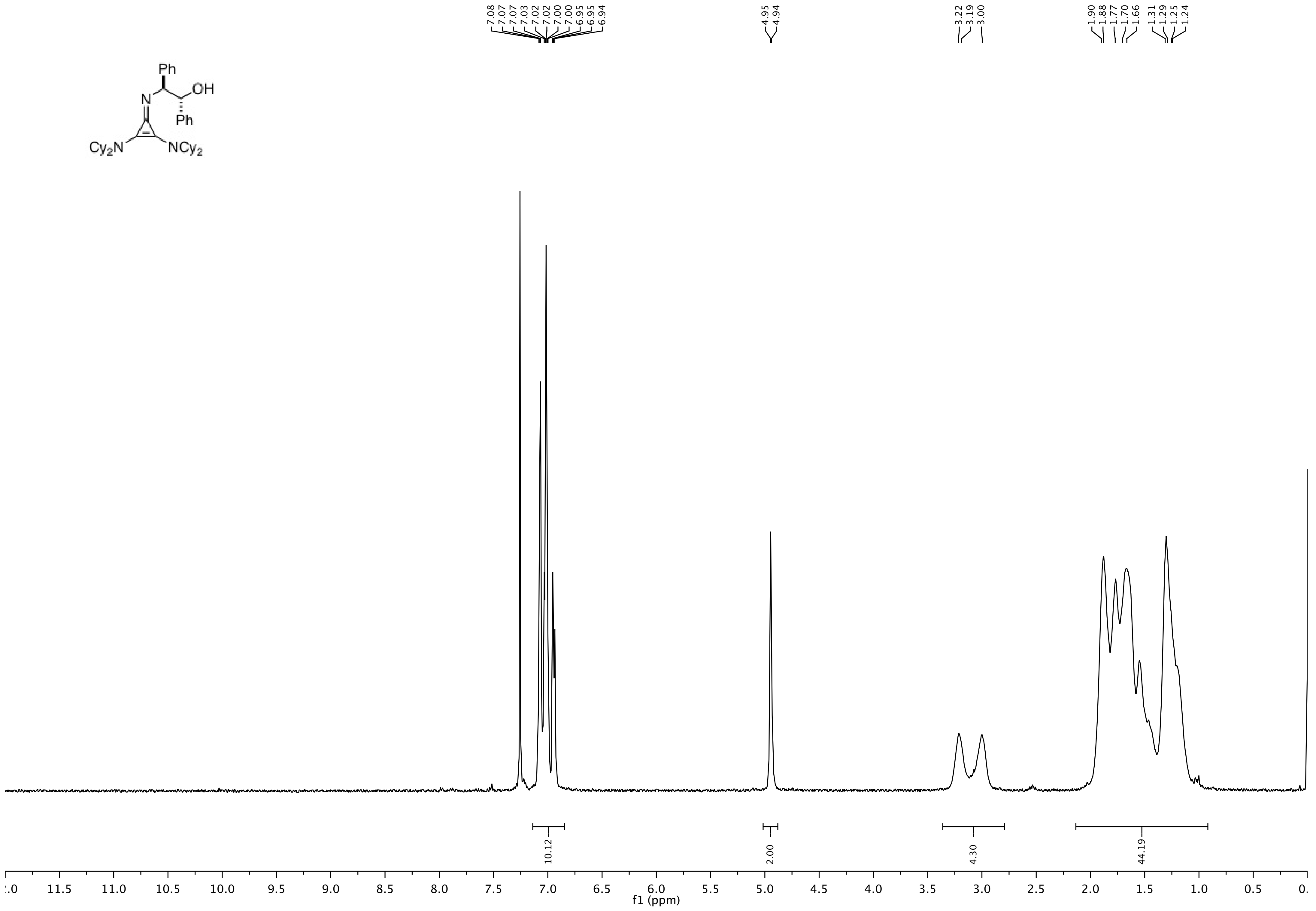
Ph
OH
N
Ph
Cy2N
NCy2
7.08
7.07
7.07
7.03
7.02
7.02
7.00
7.00
6.95
6.95
6.94
4.95
4.94
3.22
3.19
3.00
1.90
1.88
1.77
1.70
1.66
1.31
1.29
1.25
1.24
10.12
2.00
4.30
44.19
11.5
11.0
10.5
10.0
9.5
9.0
8.5
8.0
7.5
7.0
6.5
6.0
5.5
5.0
4.5
4.0
3.5
3.0
2.5
2.0
1.5
1.0
0.5
f1 (ppm)

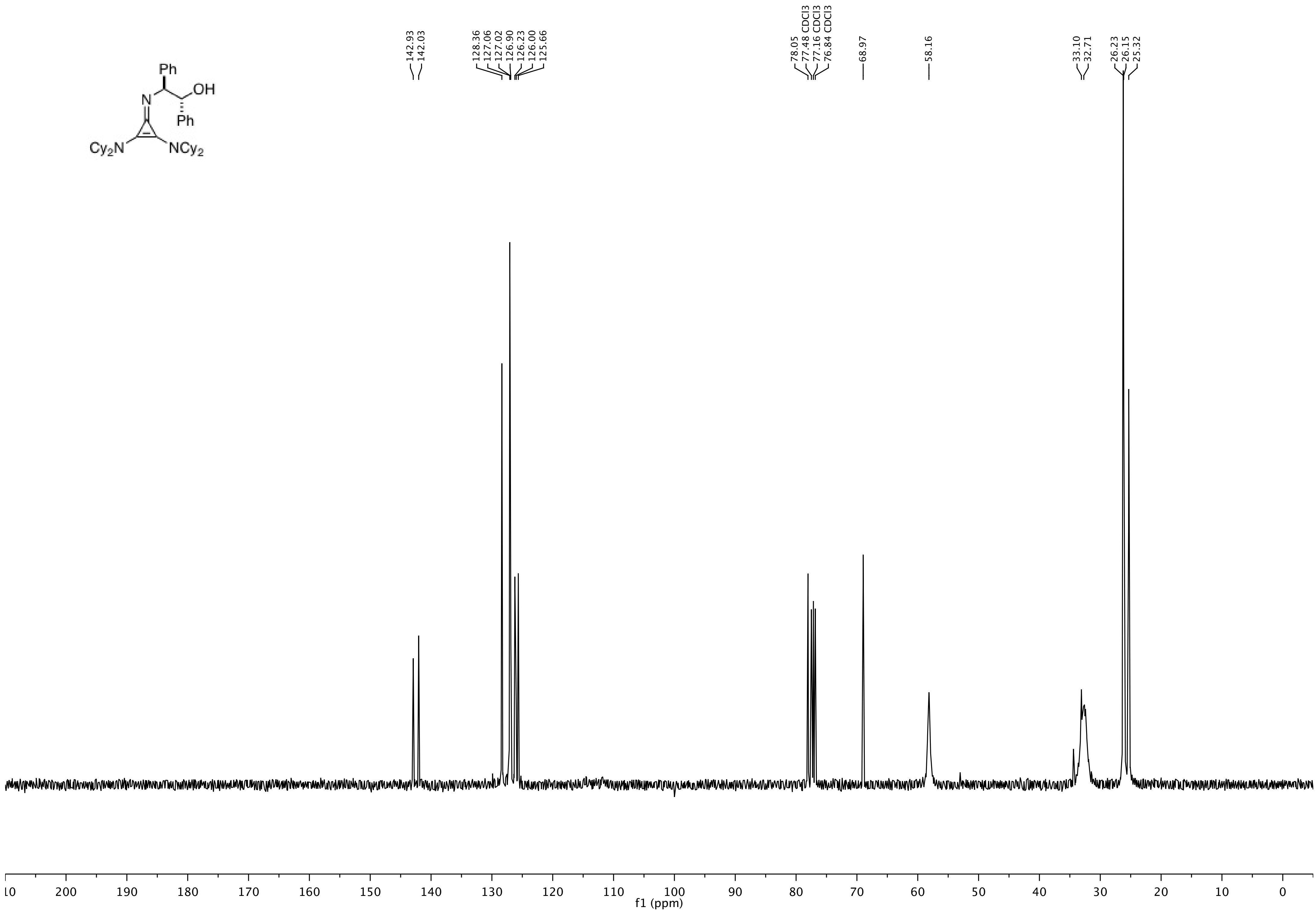

142.93
142.03
128.36
127.06
127.02
126.90
126.23
126.00
125.66
78.05
77.48 CDCl3
77.16 CDCl3
76.84 CDCl3
68.97
58.16
33.10
32.71
26.23
26.15
25.32
10
200
190
180
170
160
150
140
130
120
110
100
90
80
70
60
50
40
30
20
10
0
f1 (ppm)

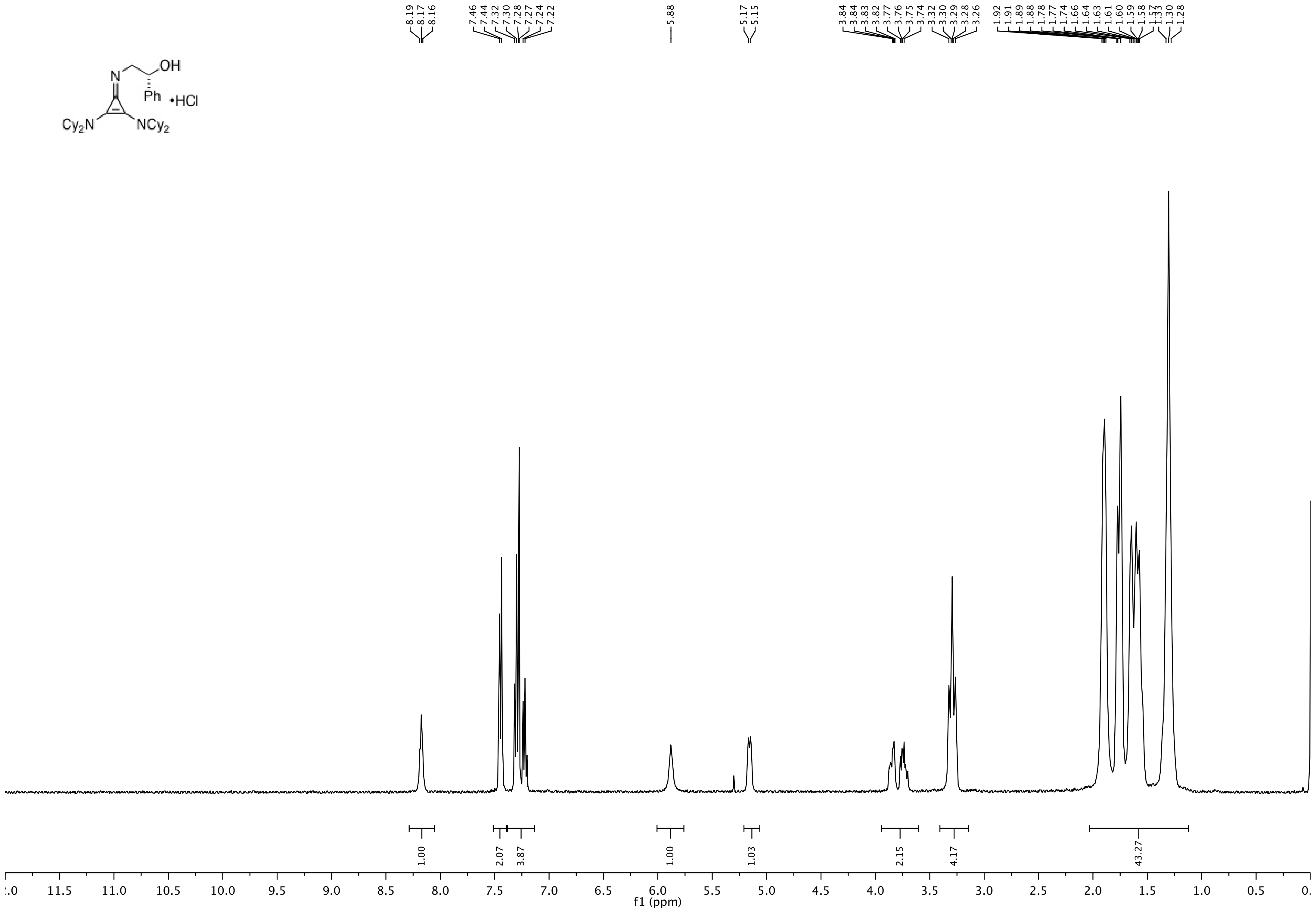

8.19
8.17
8.16
7.46
7.44
7.32
7.30
7.28
7.27
7.24
7.22
5.88
5.17
5.15
3.84
3.84
3.83
3.82
3.77
3.76
3.75
3.74
3.32
3.30
3.29
3.28
3.26
1.92
1.91
1.89
1.88
1.78
1.77
1.74
1.66
1.64
1.63
1.61
1.60
1.59
1.58
1.57
1.33
1.30
1.28
OH
N
Ph
•HCl
Cy_2N
NCy_2
1.00
2.07
3.87
1.00
1.03
2.15
4.17
43.27
11.5
11.0
10.5
10.0
9.5
9.0
8.5
8.0
7.5
7.0
6.5
6.0
5.5
5.0
4.5
4.0
3.5
3.0
2.5
2.0
1.5
1.0
0.5
f1 (ppm)

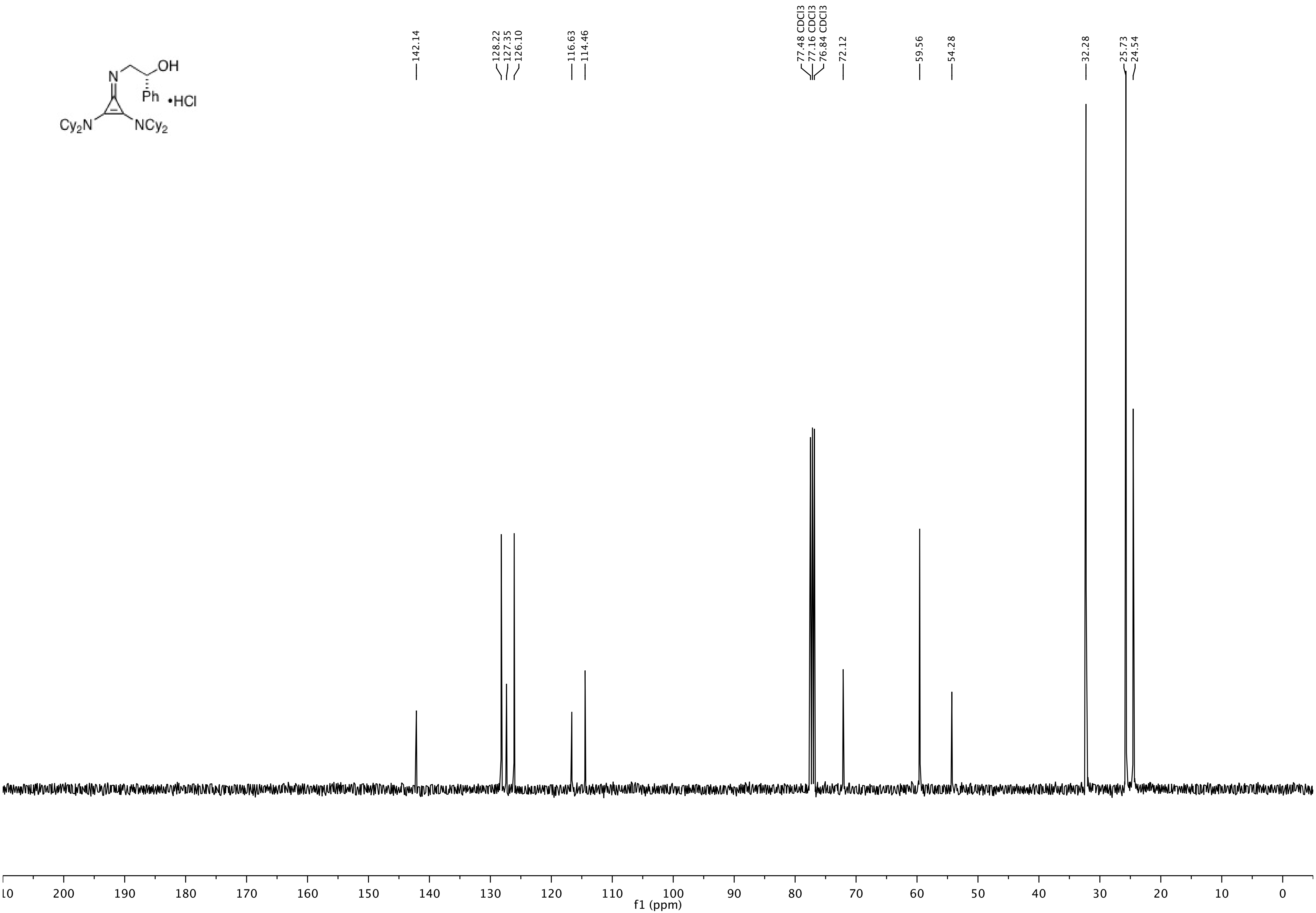

OH
N
Ph
•HCl
Cy2N
NCy2
142.14
128.22
127.35
126.10
116.63
114.46
77.48 CDCl3
77.16 CDCl3
76.84 CDCl3
72.12
59.56
54.28
32.28
25.73
24.54
200 190 180 170 160 150 140 130 120 110 100 90 80 70 60 50 40 30 20 10 0
f1 (ppm)

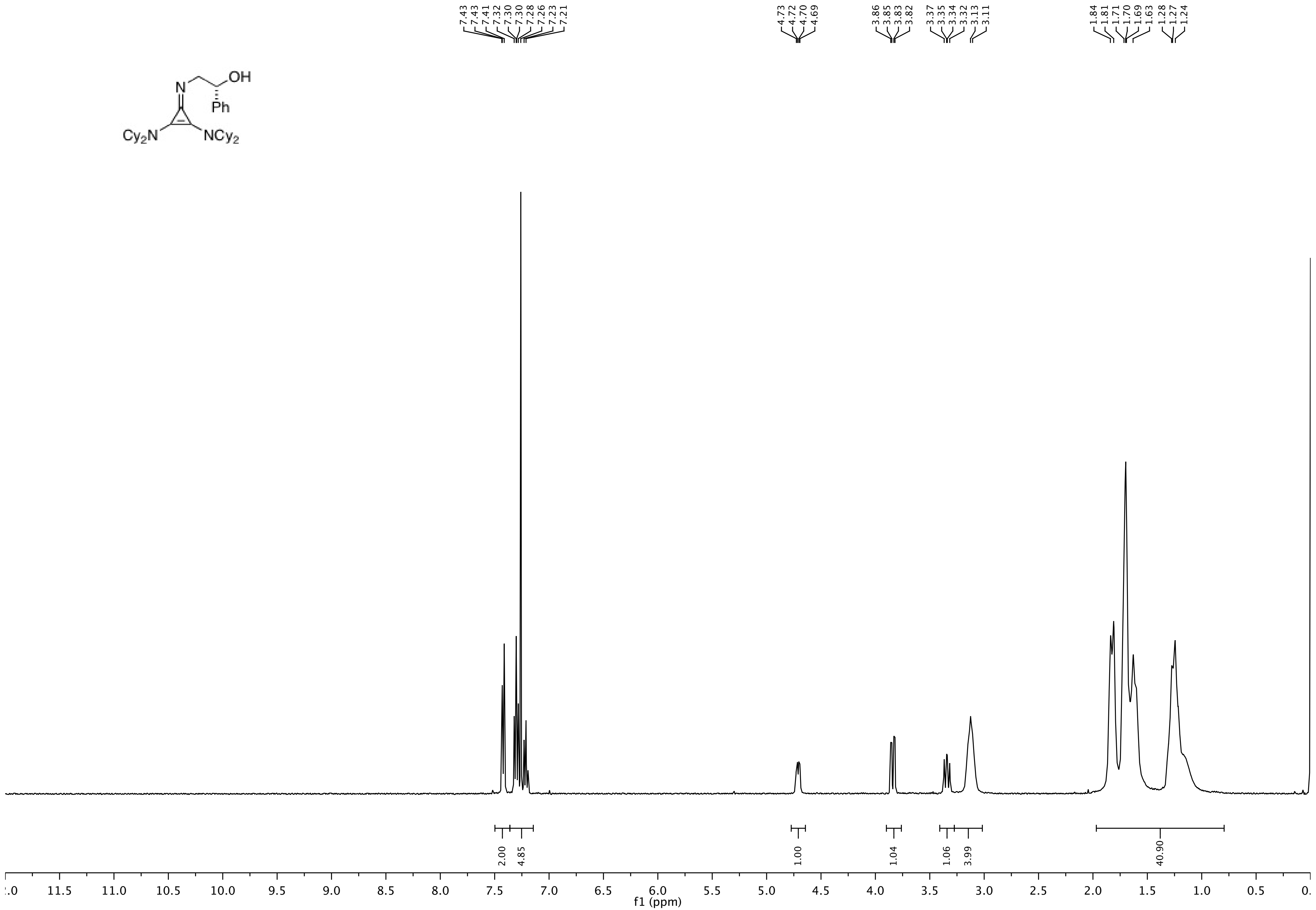

7.43
7.43
7.41
7.32
7.30
7.30
7.28
7.26
7.23
7.21
4.73
4.72
4.70
4.69
3.86
3.85
3.83
3.82
3.37
3.35
3.34
3.32
3.13
3.11
1.84
1.81
1.71
1.70
1.69
1.63
1.28
1.27
1.24
OH
N
Ph
Cy2N
NCy2
2.00
4.85
1.00
1.04
1.06
3.99
40.90
11.5
11.0
10.5
10.0
9.5
9.0
8.5
8.0
7.5
7.0
6.5
6.0
5.5
5.0
4.5
4.0
3.5
3.0
2.5
2.0
1.5
1.0
0.5
f1 (ppm)

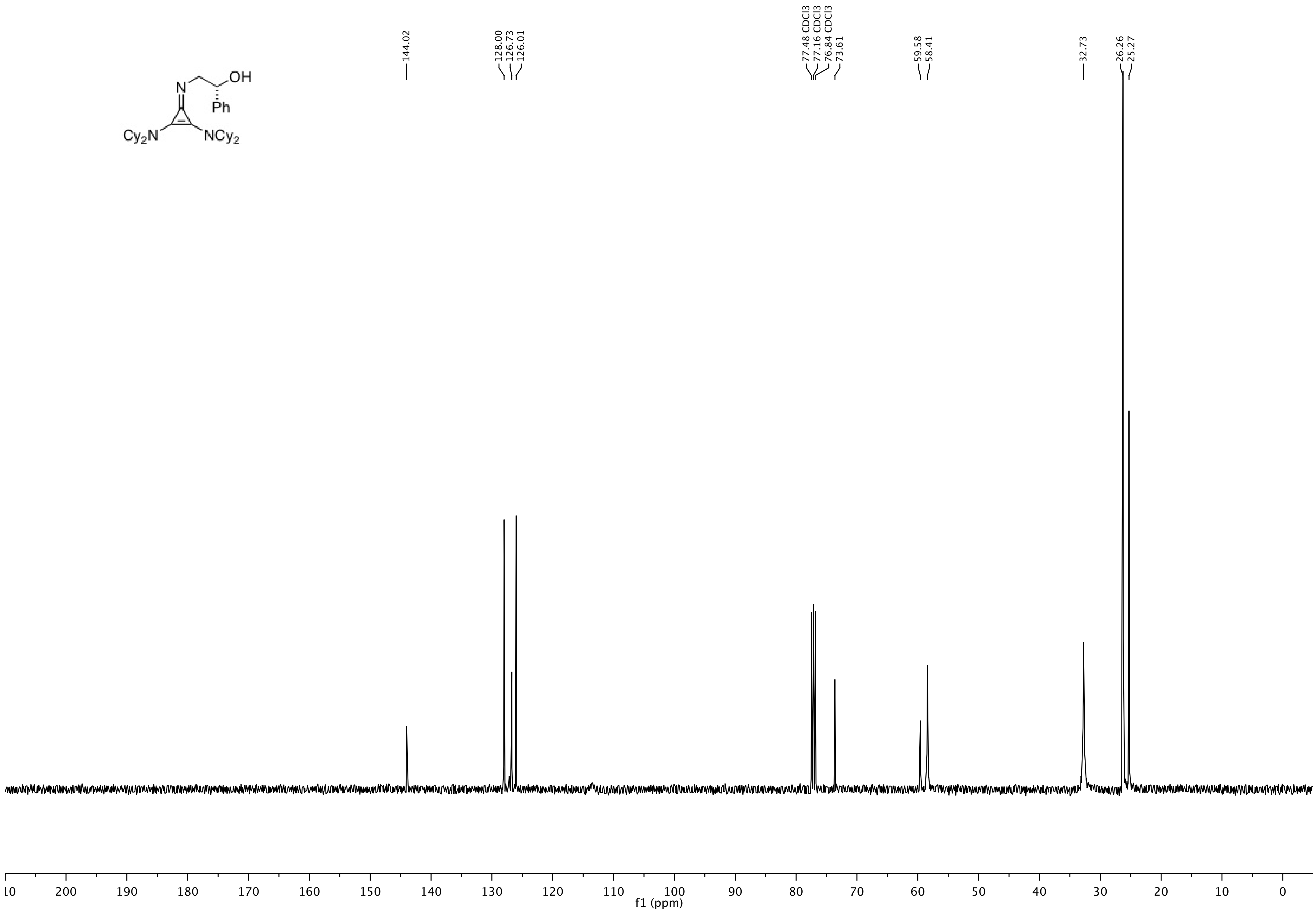
144.02
128.00
126.73
126.01
77.48 CDCl3
77.16 CDCl3
76.84 CDCl3
73.61
59.58
58.41
32.73
26.26
25.27
OH
N
Ph
Cy2N
NCy2
10 200 190 180 170 160 150 140 130 120 110 100 90 80 70 60 50 40 30 20 10 0
f1 (ppm)

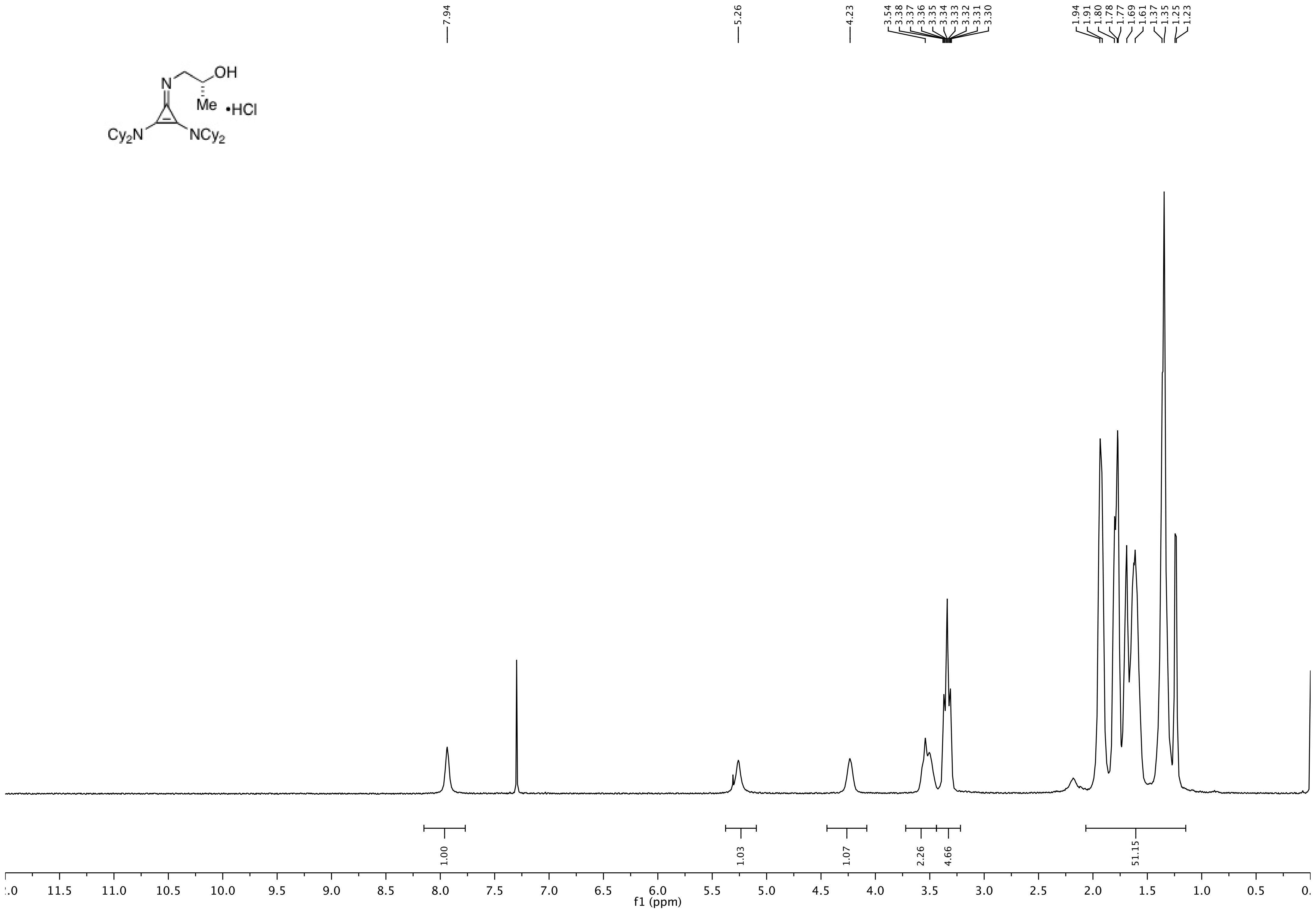
7.94
5.26
4.23
3.54
3.38
3.37
3.36
3.35
3.34
3.33
3.32
3.31
3.30
1.94
1.91
1.80
1.78
1.77
1.69
1.61
1.37
1.35
1.25
1.23
OH
N
Me
•HCl
Cy_2N
NCy_2
1.00
1.03
1.07
2.26
4.66
51.15
11.5
11.0
10.5
10.0
9.5
9.0
8.5
8.0
7.5
7.0
6.5
6.0
5.5
5.0
4.5
4.0
3.5
3.0
2.5
2.0
1.5
1.0
0.5
f1 (ppm)

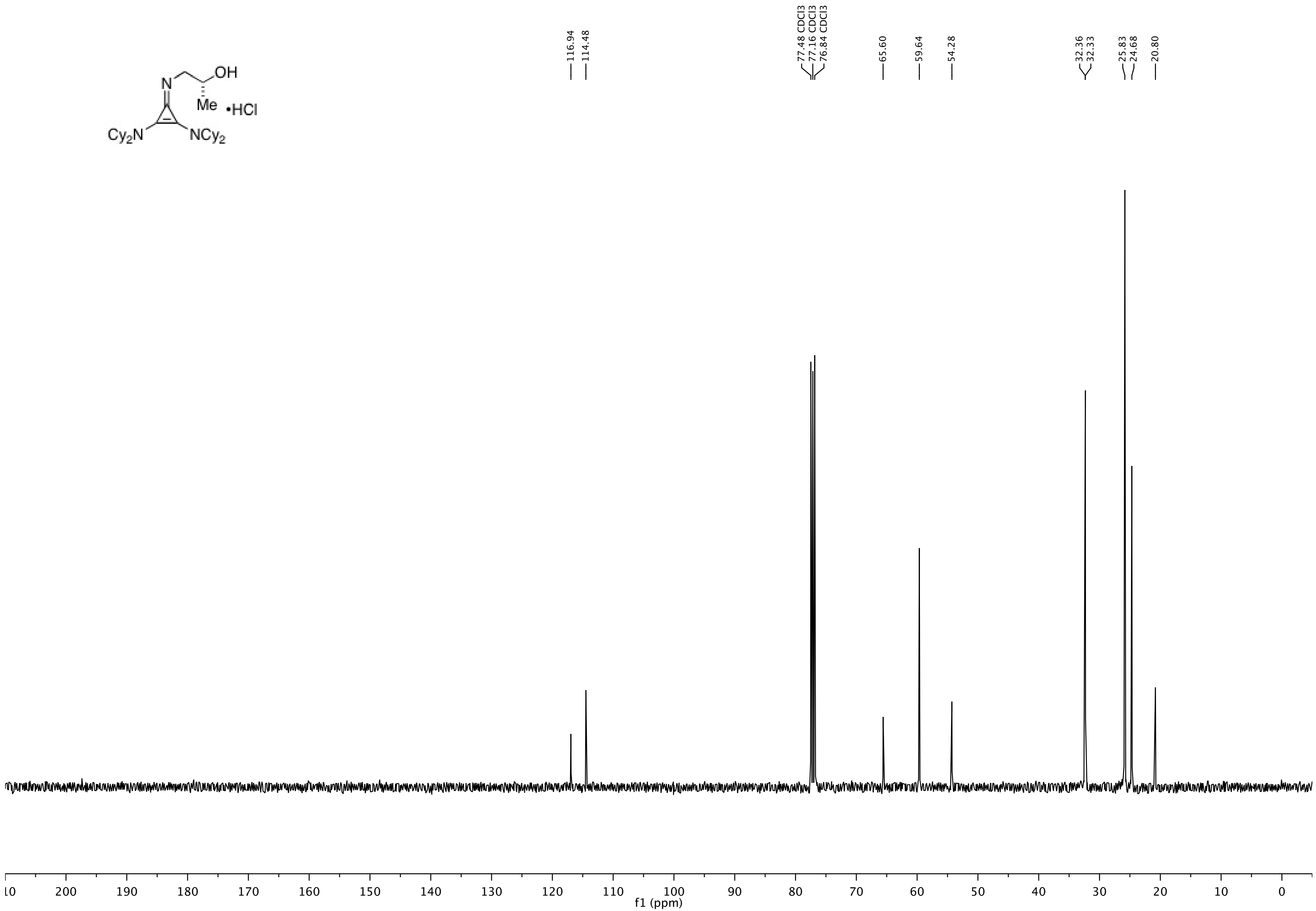
116.94
114.48
77.48 CDCl3
77.16 CDCl3
76.84 CDCl3
65.60
59.64
54.28
32.36
32.33
25.83
24.68
20.80
OH
N
Me
•HCl
Cy2N
NCy2
10
200
190
180
170
160
150
140
130
120
110
100
90
80
70
60
50
40
30
20
10
0
f1 (ppm)

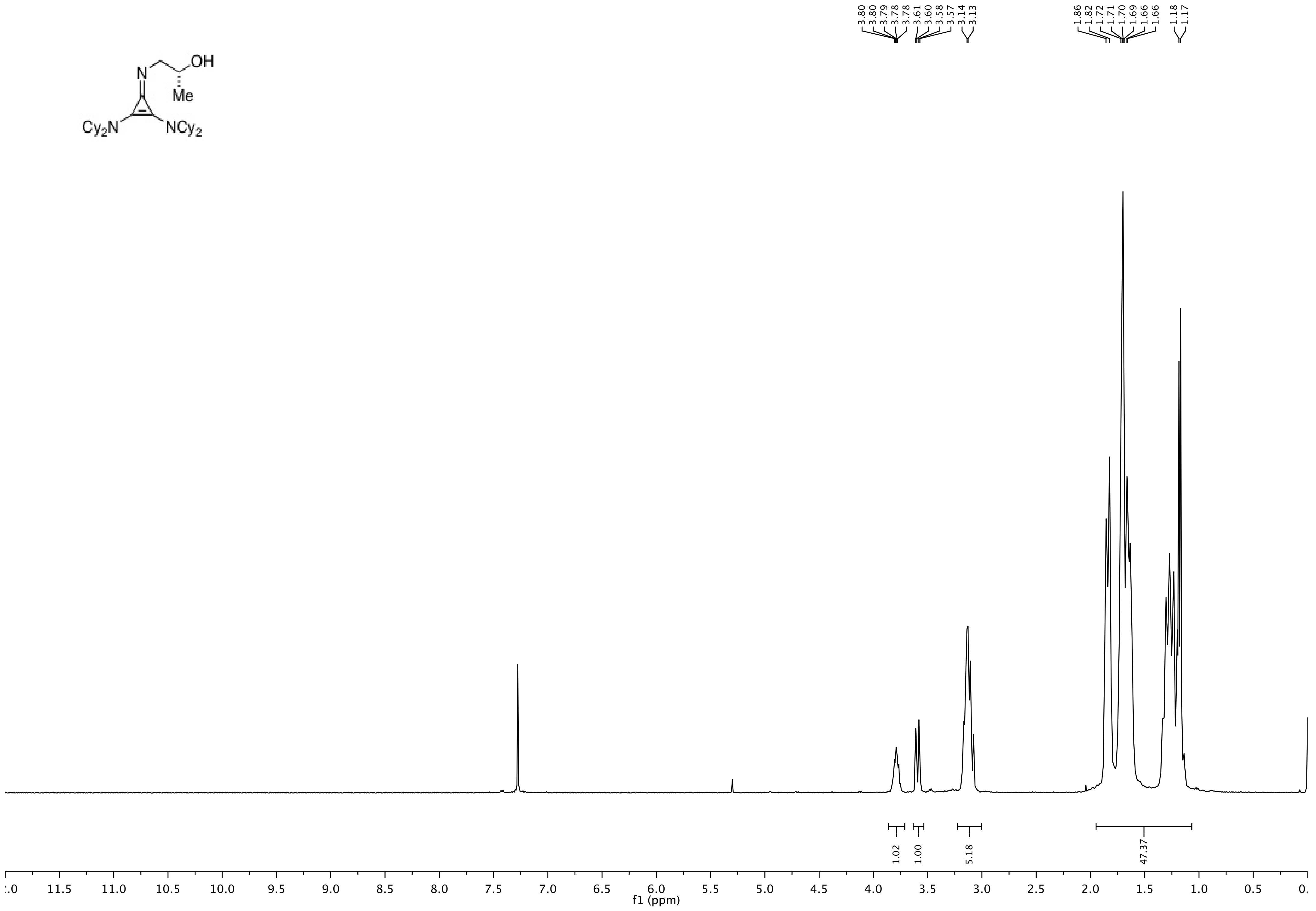

OH
N
Me
Cy2N
NCy2
3.80
3.80
3.79
3.78
3.78
3.61
3.60
3.58
3.57
3.14
3.13
1.86
1.82
1.72
1.71
1.70
1.69
1.66
1.66
1.18
1.17
1.02
1.00
5.18
47.37
11.5
11.0
10.5
10.0
9.5
9.0
8.5
8.0
7.5
7.0
6.5
6.0
5.5
5.0
4.5
4.0
3.5
3.0
2.5
2.0
1.5
1.0
0.5
f1 (ppm)

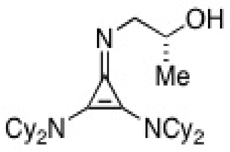
N
OH
Me
Cy_2N
NCy_2

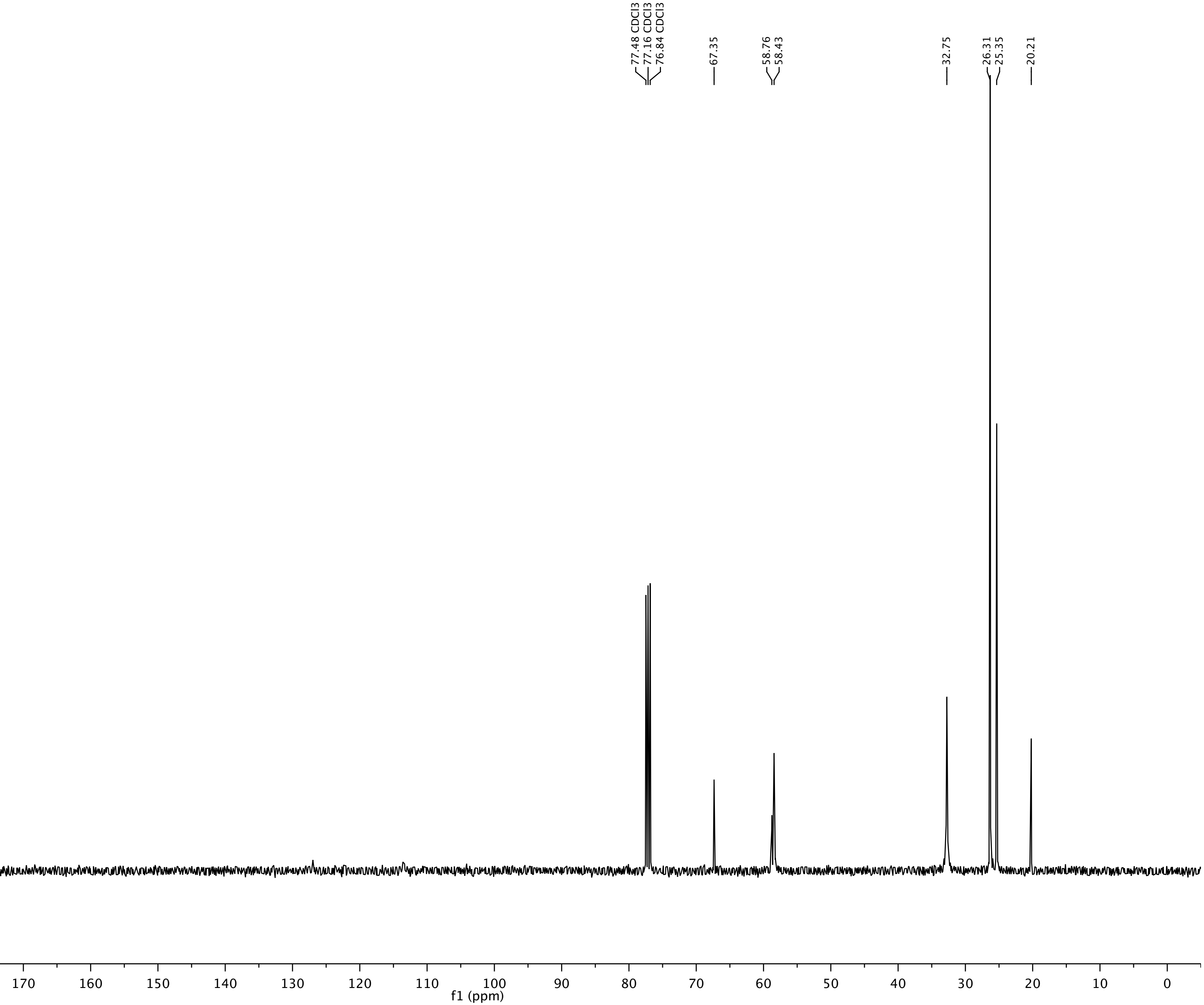
77.48 CDCl3
77.16 CDCl3
76.84 CDCl3
67.35
58.76
58.43
32.75
26.31
25.35
20.21
200
190
180
170
160
150
140
130
120
110
100
90
80
70
60
50
40
30
20
10
0
f1 (ppm)

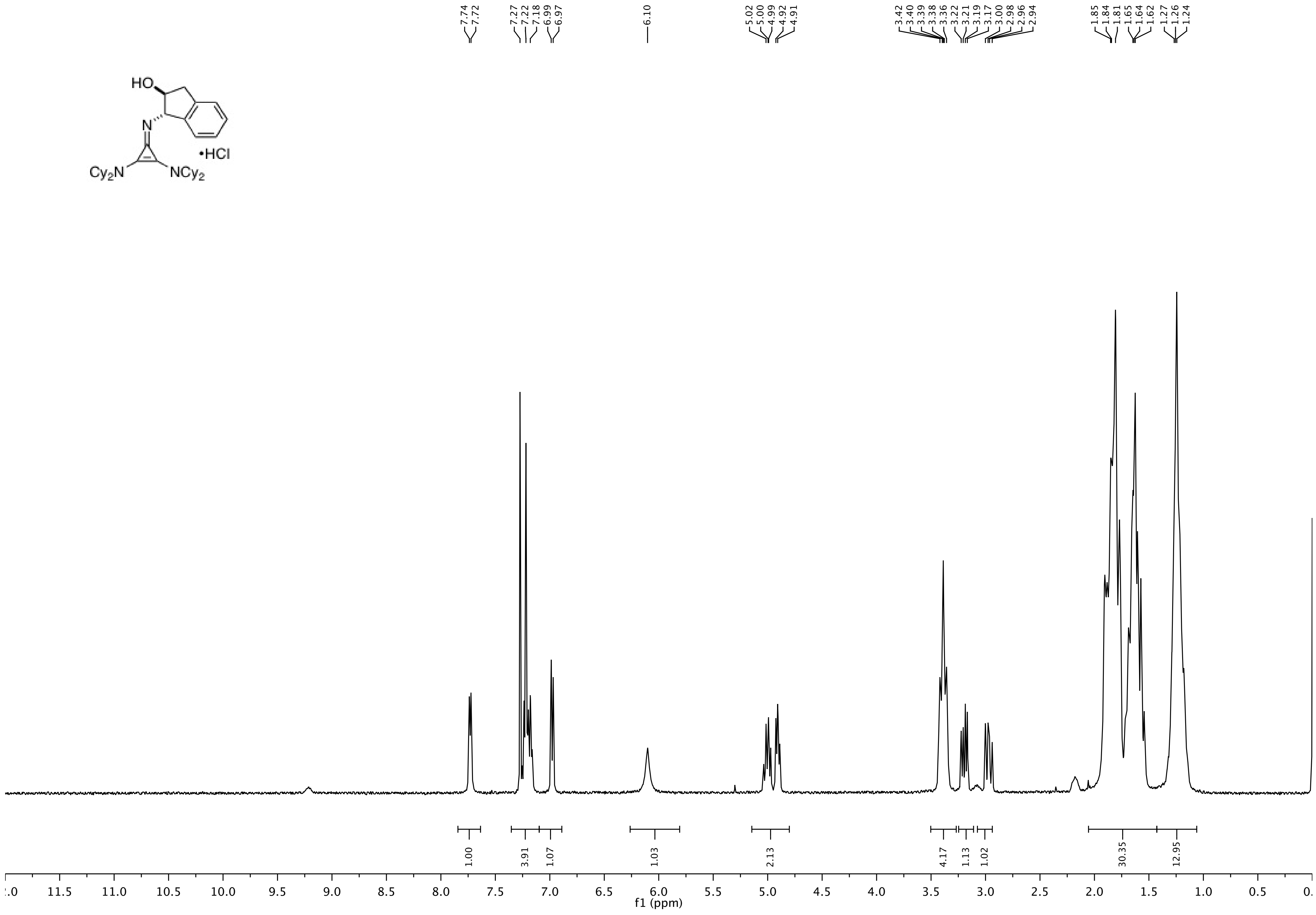
HO
N
Cy2N
NCy2
•HCl
7.74
7.72
7.27
7.22
7.18
6.99
6.97
6.10
5.02
5.00
4.99
4.92
4.91
3.42
3.40
3.39
3.38
3.36
3.22
3.21
3.19
3.17
3.00
2.98
2.96
2.94
1.85
1.84
1.81
1.65
1.64
1.62
1.27
1.26
1.24
1.00
3.91
1.07
1.03
2.13
4.17
1.13
1.02
30.35
12.95
11.5
11.0
10.5
10.0
9.5
9.0
8.5
8.0
7.5
7.0
6.5
6.0
5.5
5.0
4.5
4.0
3.5
3.0
2.5
2.0
1.5
1.0
0.5
f1 (ppm)

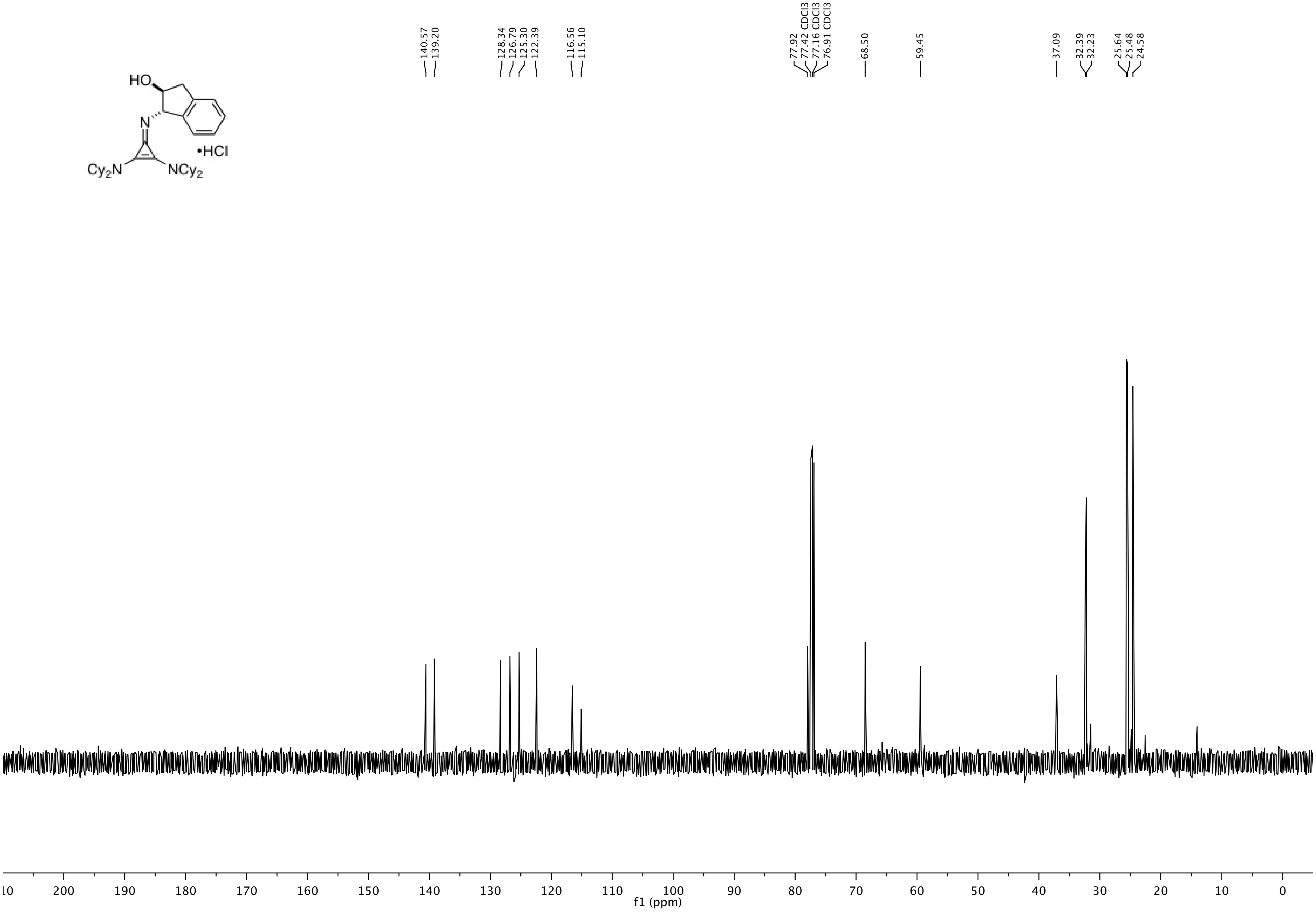
HO
N
Cy2N
NCy2
•HCl
140.57
139.20
128.34
126.79
125.30
122.39
116.56
115.10
77.92
77.42 CDCl3
77.16 CDCl3
76.91 CDCl3
68.50
59.45
37.09
32.39
32.23
25.64
25.48
24.58
10
200
190
180
170
160
150
140
130
120
110
100
90
80
70
60
50
40
30
20
10
0
f1 (ppm)

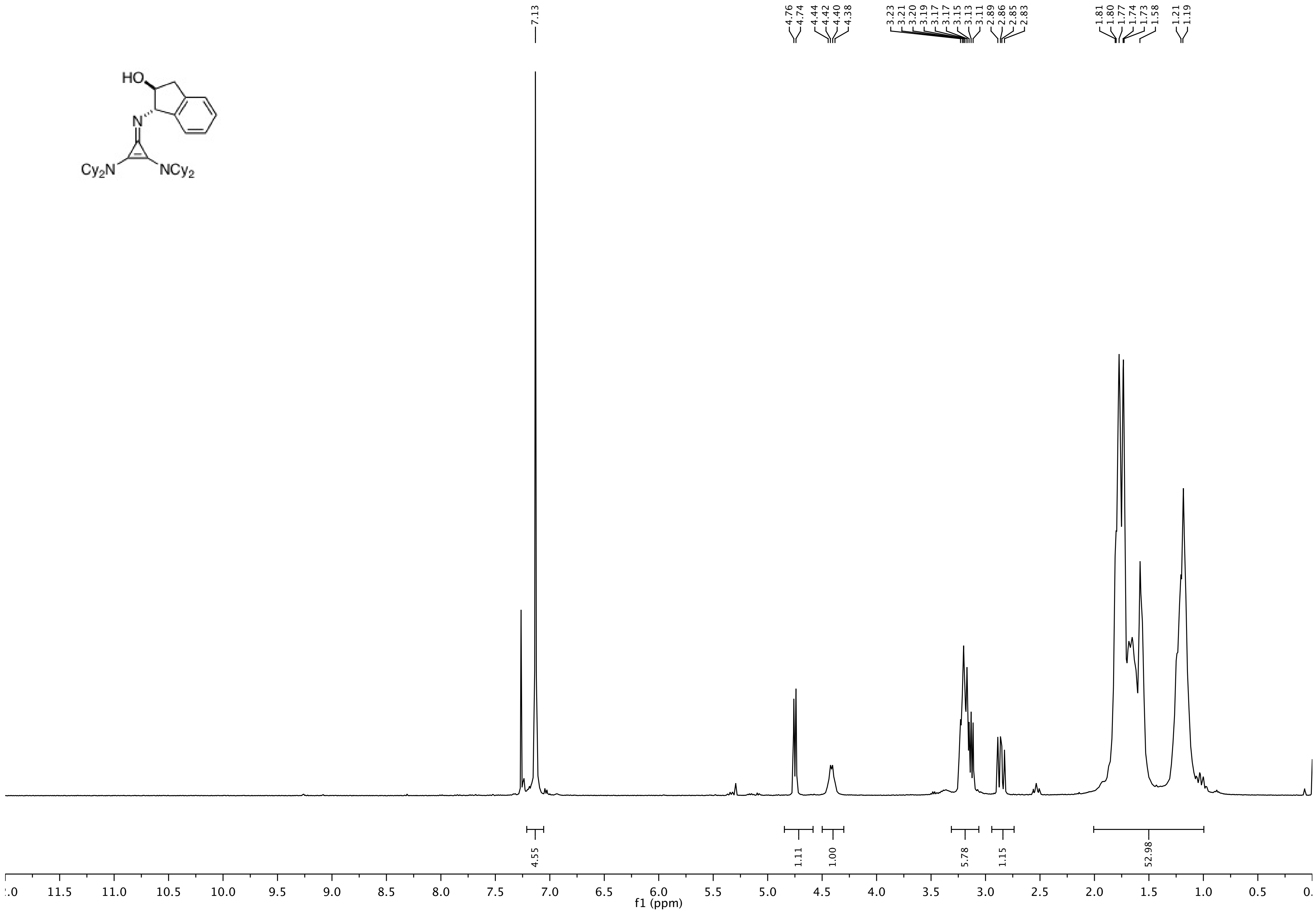
HO
N
Cy2N
NCy2
7.13
4.76
4.74
4.44
4.42
4.40
4.38
3.23
3.21
3.20
3.19
3.17
3.17
3.15
3.13
3.11
2.89
2.86
2.85
2.83
1.81
1.80
1.77
1.74
1.73
1.58
1.21
1.19
4.55
1.11
1.00
5.78
1.15
52.98
11.5
11.0
10.5
10.0
9.5
9.0
8.5
8.0
7.5
7.0
6.5
6.0
5.5
5.0
4.5
4.0
3.5
3.0
2.5
2.0
1.5
1.0
0.5
f1 (ppm)

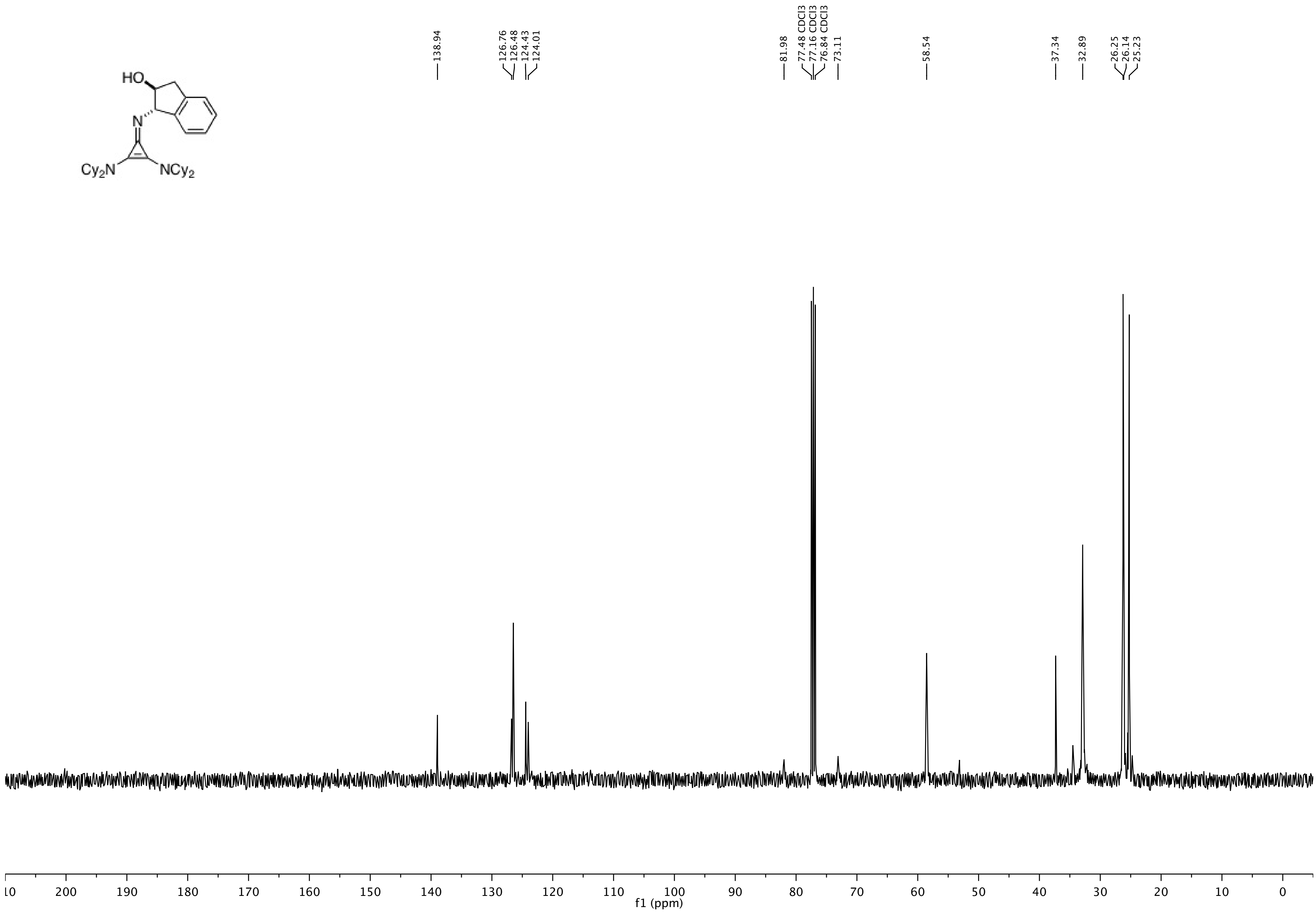

HO
N
Cy2N
NCy2
138.94
126.76
126.48
124.43
124.01
81.98
77.48 CDCl3
77.16 CDCl3
76.84 CDCl3
73.11
58.54
37.34
32.89
26.25
26.14
25.23
f1 (ppm)

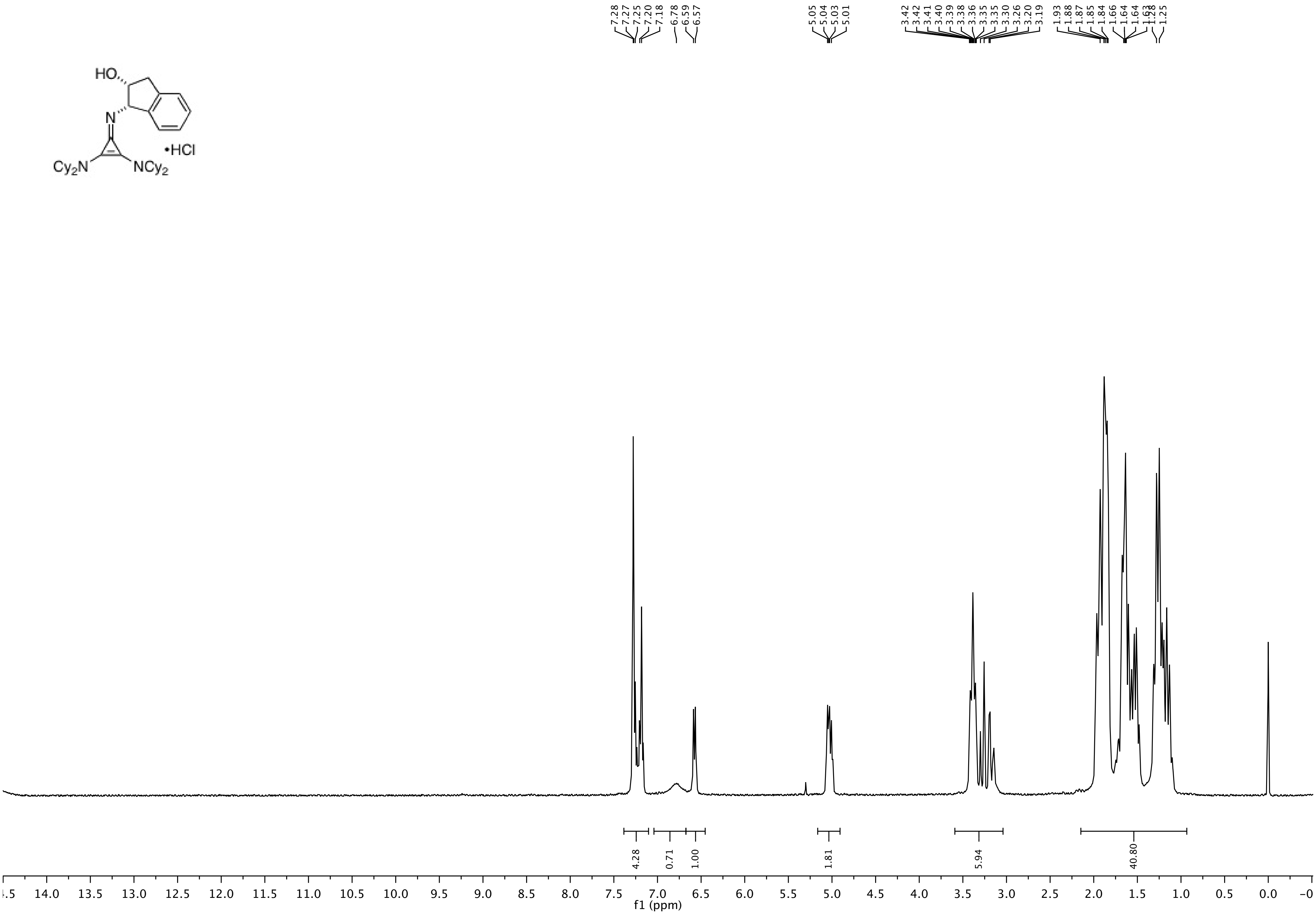
HO
N
Cy2N
NCy2
•HCl
7.28 7.27 7.25 7.20 7.18 6.78 6.59 6.57
5.05 5.04 5.03 5.01
3.42 3.42 3.41 3.40 3.39 3.38 3.36 3.35 3.35 3.30 3.26 3.20 3.19
1.93 1.88 1.87 1.85 1.84 1.66 1.64 1.64 1.63 1.28 1.25
4.28 0.71 1.00 1.81 5.94 40.80
f1 (ppm)

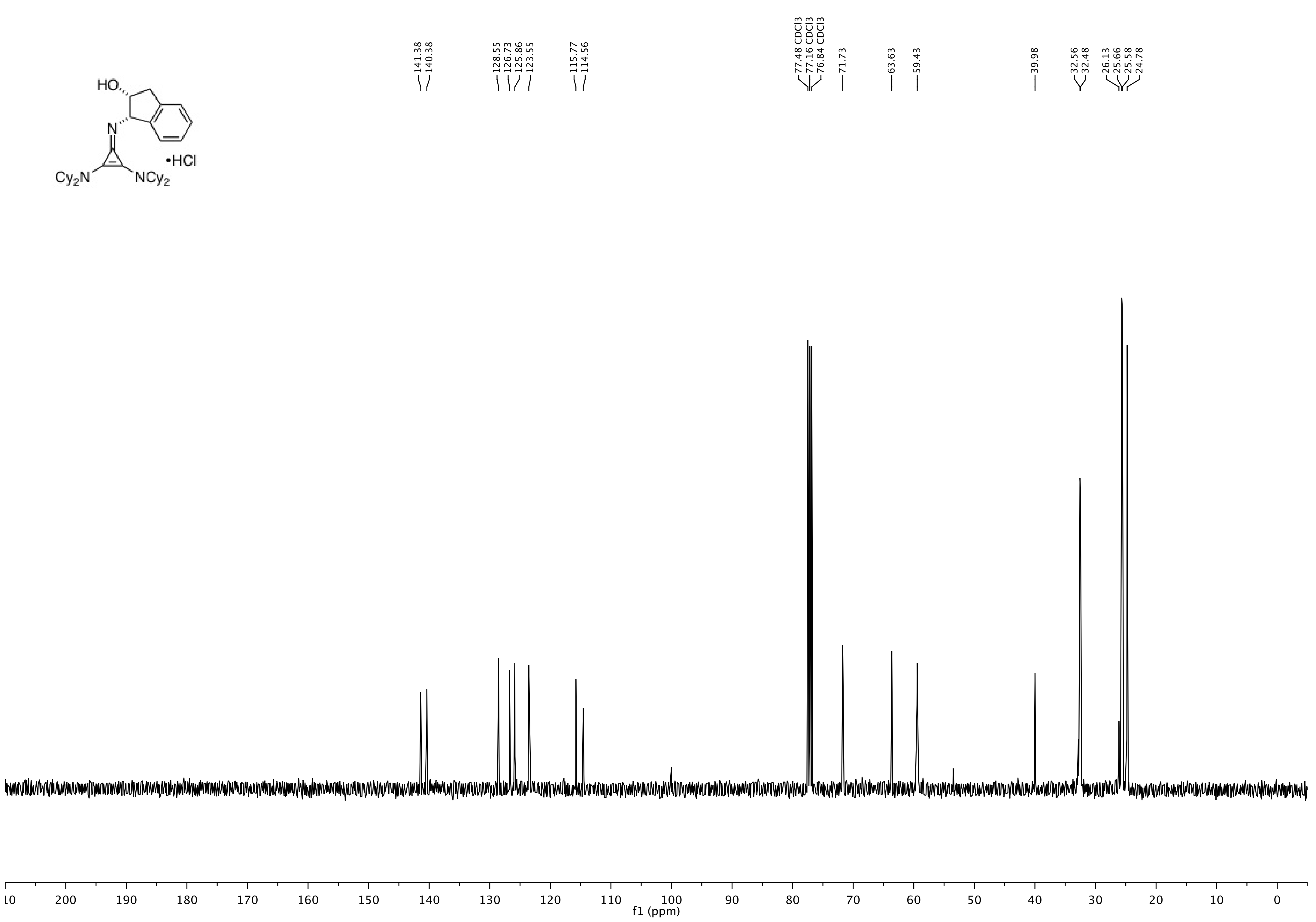

HO
N
•HCl
Cy2N
NCy2
141.38
140.38
128.55
126.73
125.86
123.55
115.77
114.56
77.48 CDCl3
77.16 CDCl3
76.84 CDCl3
71.73
63.63
59.43
39.98
32.56
32.48
26.13
25.66
25.58
24.78
10
200
190
180
170
160
150
140
130
120
110
100
90
80
70
60
50
40
30
20
10
0
f1 (ppm)

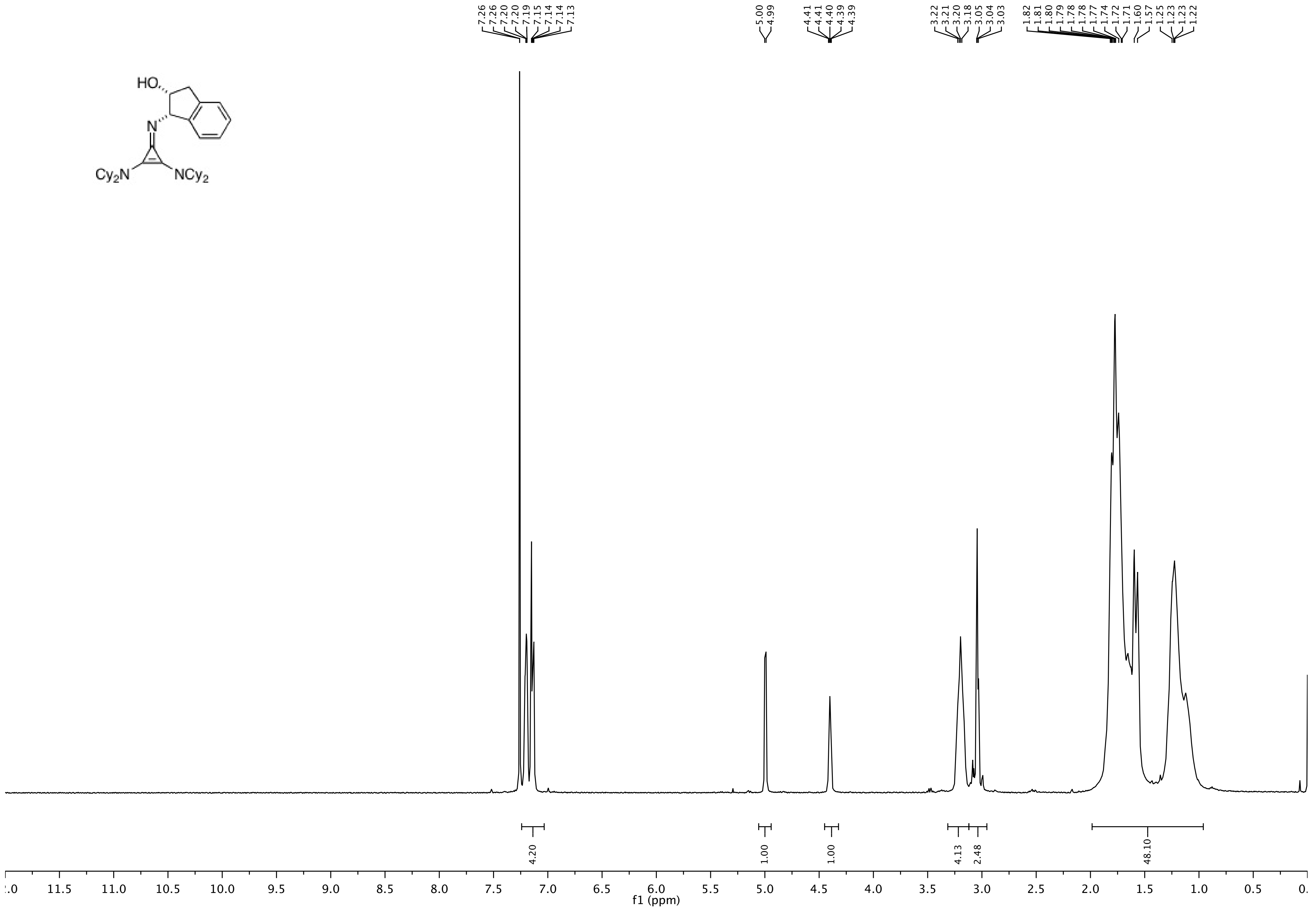

7.26
7.26
7.20
7.20
7.19
7.15
7.14
7.14
7.13
5.00
4.99
4.41
4.41
4.40
4.39
4.39
3.22
3.21
3.20
3.18
3.05
3.04
3.03
1.82
1.81
1.80
1.79
1.78
1.78
1.77
1.74
1.72
1.71
1.60
1.57
1.25
1.23
1.23
1.22
HO
N
Cy2N
NCy2
4.20
1.00
1.00
4.13
2.48
48.10
11.5
11.0
10.5
10.0
9.5
9.0
8.5
8.0
7.5
7.0
6.5
6.0
5.5
5.0
4.5
4.0
3.5
3.0
2.5
2.0
1.5
1.0
0.5
f1 (ppm)

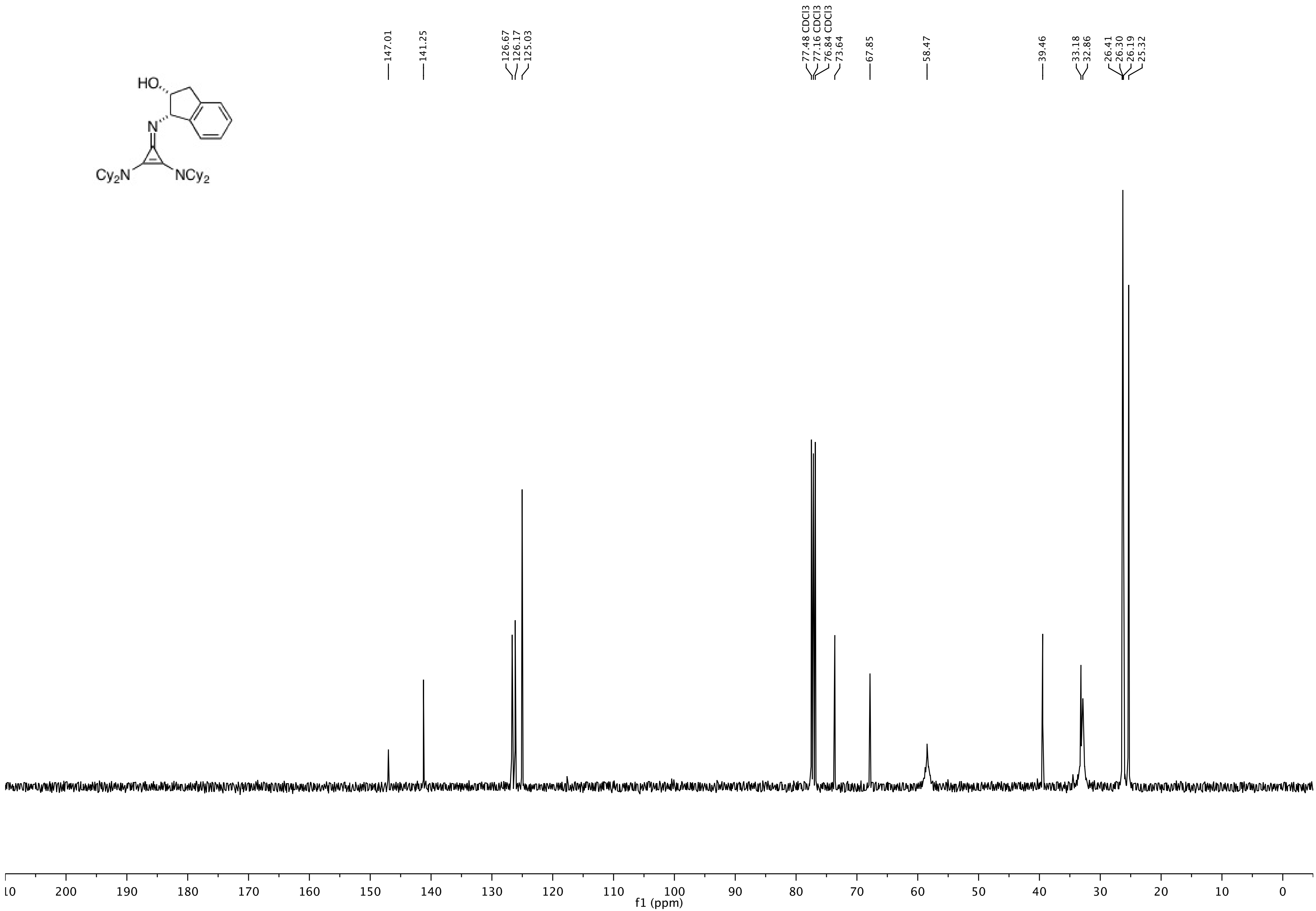
147.01
141.25
126.67
126.17
125.03
77.48 CDCl3
77.16 CDCl3
76.84 CDCl3
73.64
67.85
58.47
39.46
33.18
32.86
26.41
26.30
26.19
25.32
HO
N
Cy2N
NCy2
200
190
180
170
160
150
140
130
120
110
100
90
80
70
60
50
40
30
20
10
0
f1 (ppm)

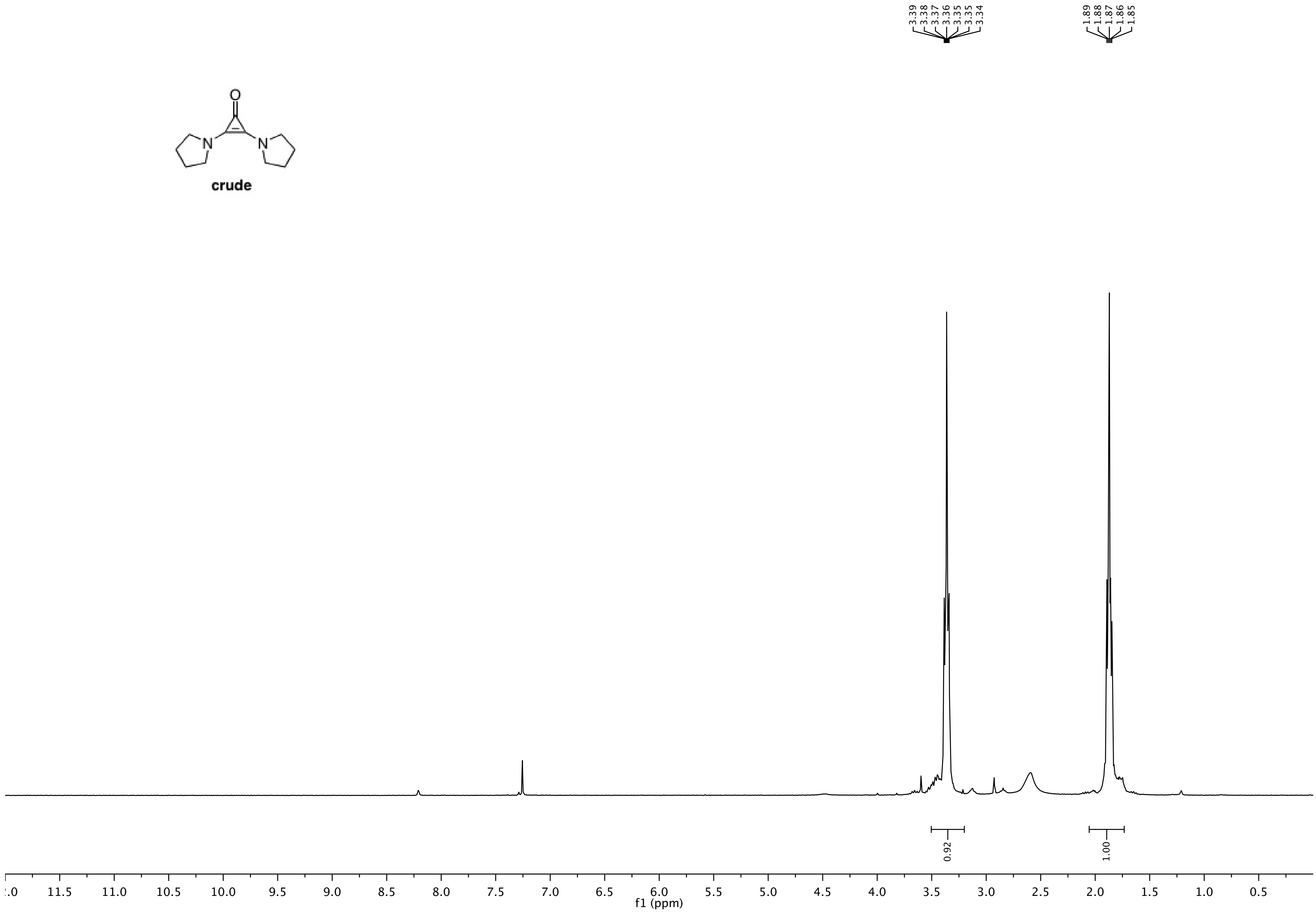

crude
3.39 3.38 3.37 3.36 3.35 3.35 3.34
1.89 1.88 1.87 1.86 1.85
0.92
1.00
11.5 11.0 10.5 10.0 9.5 9.0 8.5 8.0 7.5 7.0 6.5 6.0 5.5 5.0 4.5 4.0 3.5 3.0 2.5 2.0 1.5 1.0 0.5
f1 (ppm)

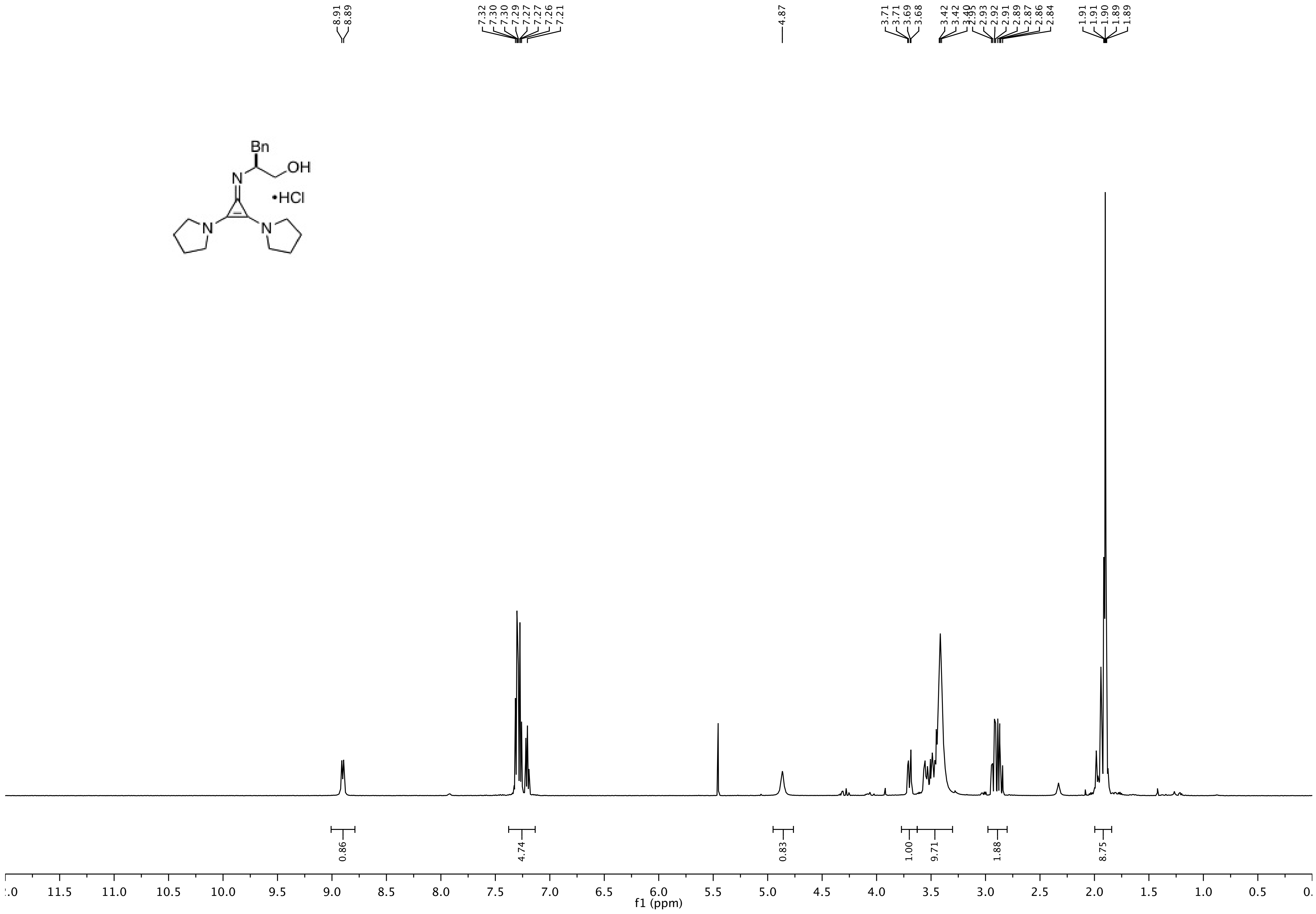

8.91
8.89
7.32
7.30
7.30
7.29
7.27
7.27
7.26
7.21
4.87
3.71
3.71
3.69
3.68
3.42
3.42
3.40
2.95
2.93
2.92
2.91
2.89
2.87
2.86
2.84
1.91
1.91
1.90
1.89
1.89
Bn
OH
N
•HCl
N
N
0.86
4.74
0.83
1.00
9.71
1.88
8.75
11.5
11.0
10.5
10.0
9.5
9.0
8.5
8.0
7.5
7.0
6.5
6.0
5.5
5.0
4.5
4.0
3.5
3.0
2.5
2.0
1.5
1.0
0.5
f1 (ppm)

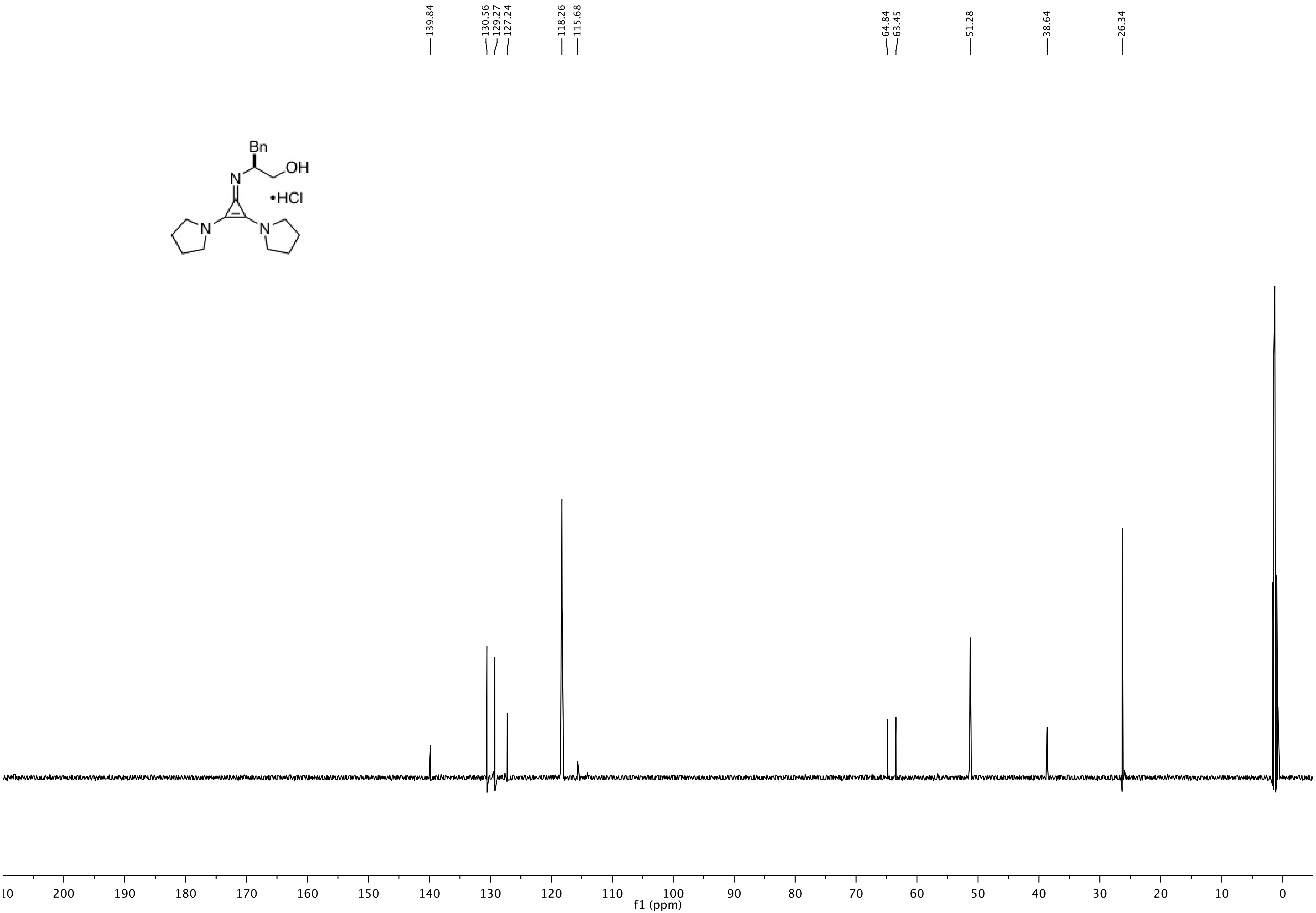

139.84
130.56
129.27
127.24
118.26
115.68
64.84
63.45
51.28
38.64
26.34
Bn
OH
N
•HCl
N
N
f1 (ppm)

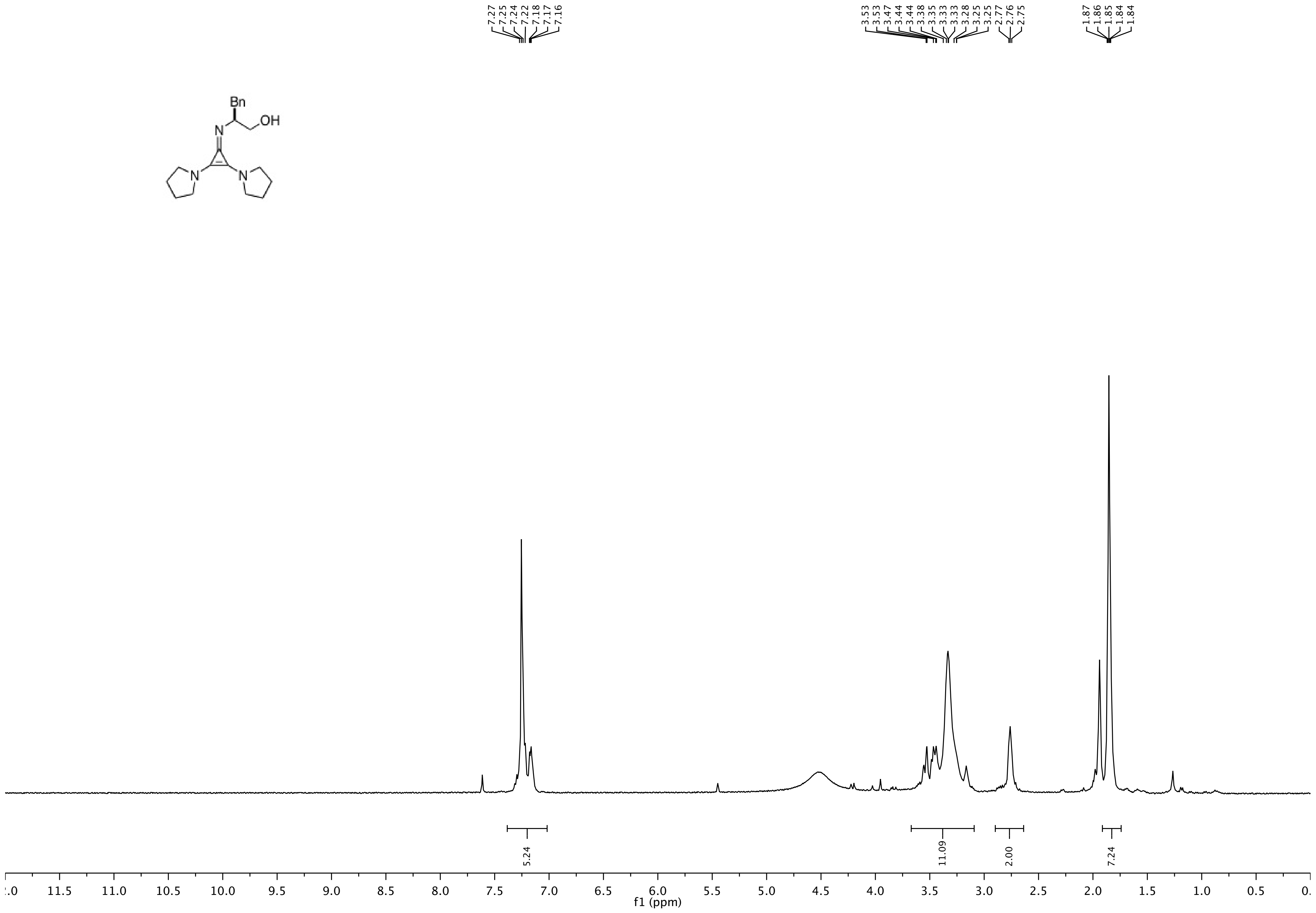

7.27 7.25 7.24 7.22 7.18 7.17 7.16
3.53 3.53 3.47 3.44 3.44 3.38 3.35 3.33 3.33 3.28 3.25 3.25 2.77 2.76 2.75
1.87 1.86 1.85 1.84 1.84
Bn
OH
N
5.24
11.09
2.00
7.24
11.5 11.0 10.5 10.0 9.5 9.0 8.5 8.0 7.5 7.0 6.5 6.0 5.5 5.0 4.5 4.0 3.5 3.0 2.5 2.0 1.5 1.0 0.5
f1 (ppm)

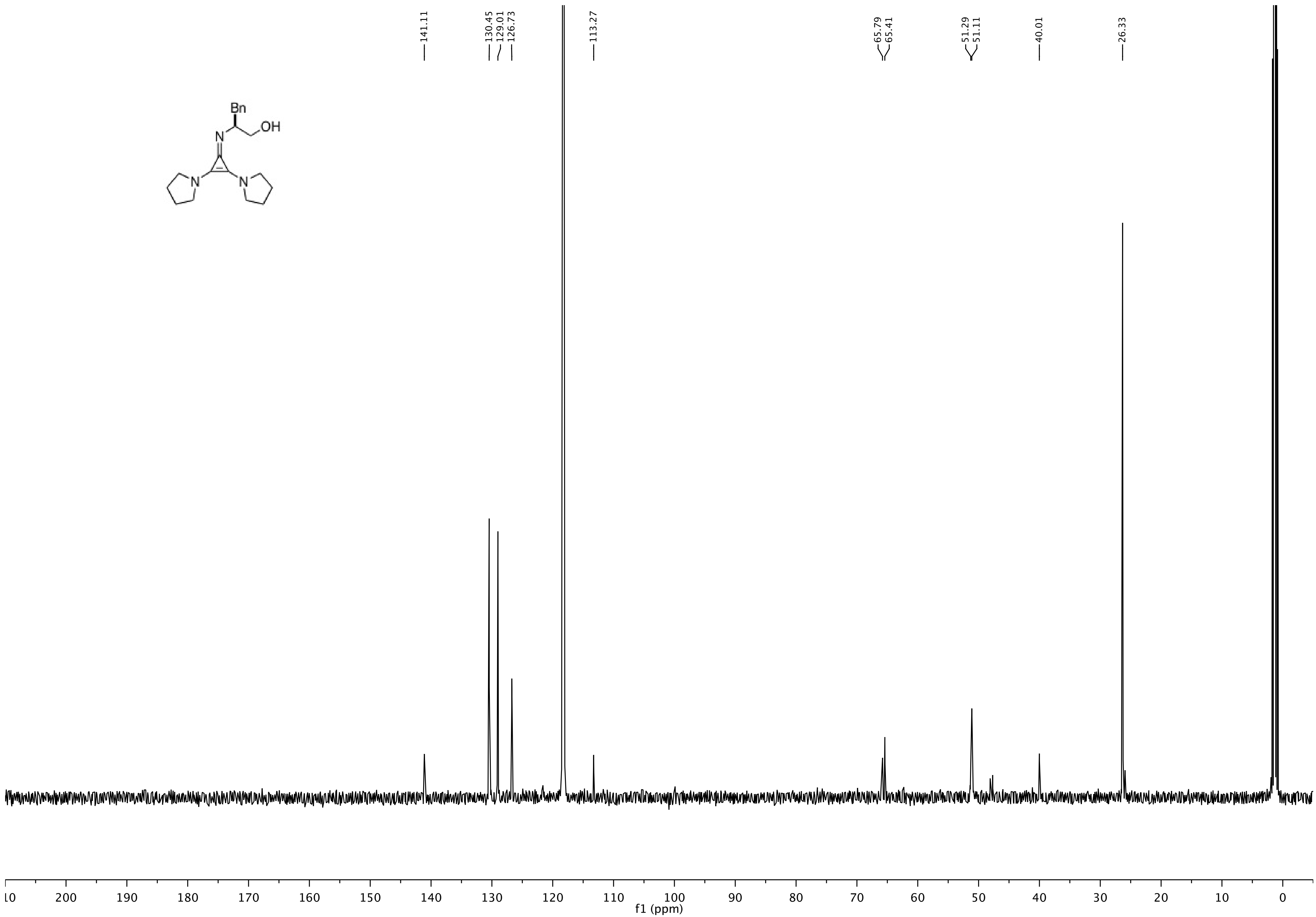

141.11
130.45
129.01
126.73
113.27
65.79
65.41
51.29
51.11
40.01
26.33
Bn
OH
N
210
200
190
180
170
160
150
140
130
120
110
100
90
80
70
60
50
40
30
20
10
0
f1 (ppm)

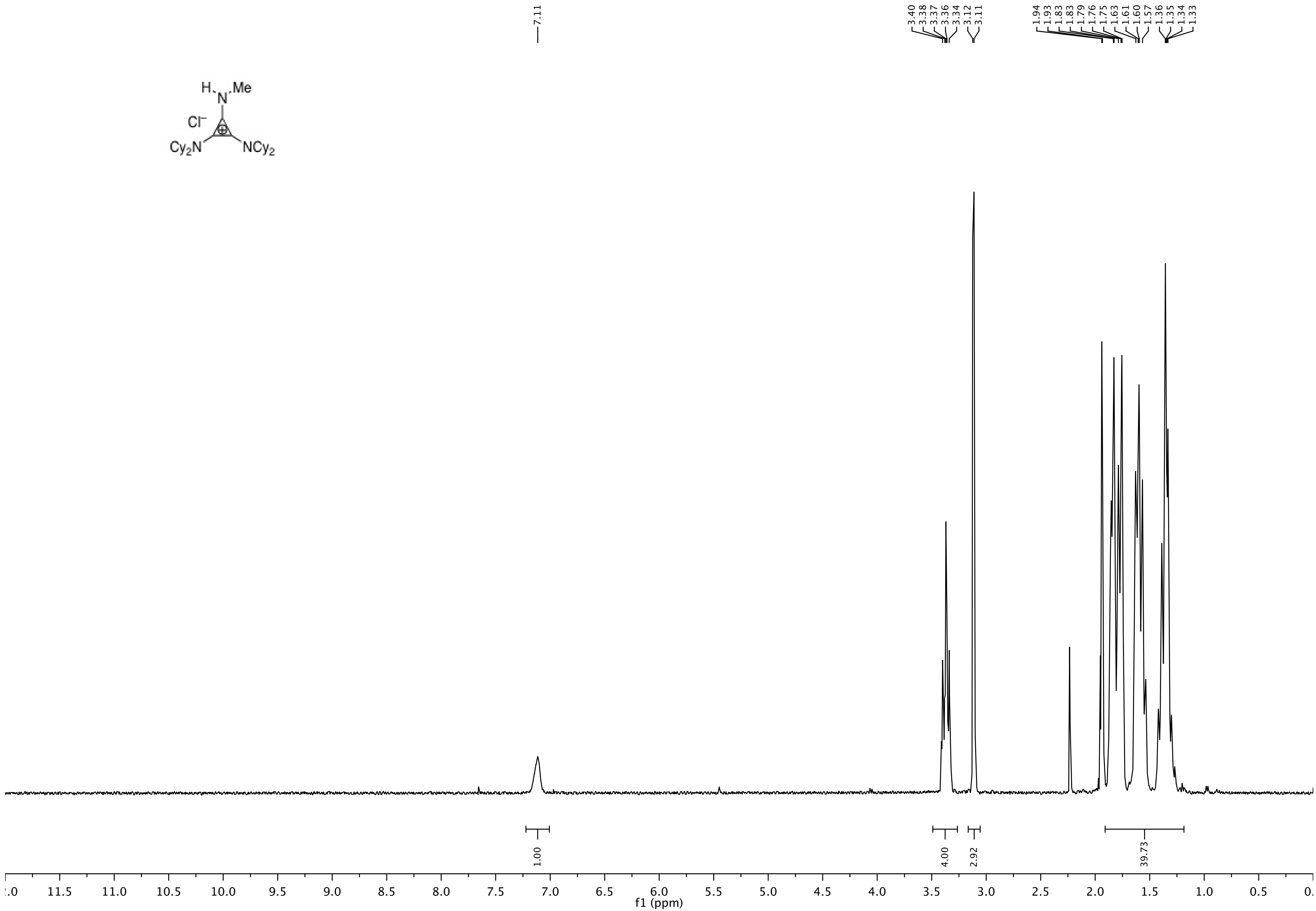

7.11
3.40
3.38
3.37
3.36
3.34
3.12
3.11
1.94
1.93
1.83
1.83
1.79
1.76
1.75
1.63
1.61
1.60
1.57
1.36
1.35
1.34
1.33
H
N
Me
Cl⁻
Cy2N
NCy2
1.00
4.00
2.92
39.73
11.5
11.0
10.5
10.0
9.5
9.0
8.5
8.0
7.5
7.0
6.5
6.0
5.5
5.0
4.5
4.0
3.5
3.0
2.5
2.0
1.5
1.0
0.5
f1 (ppm)

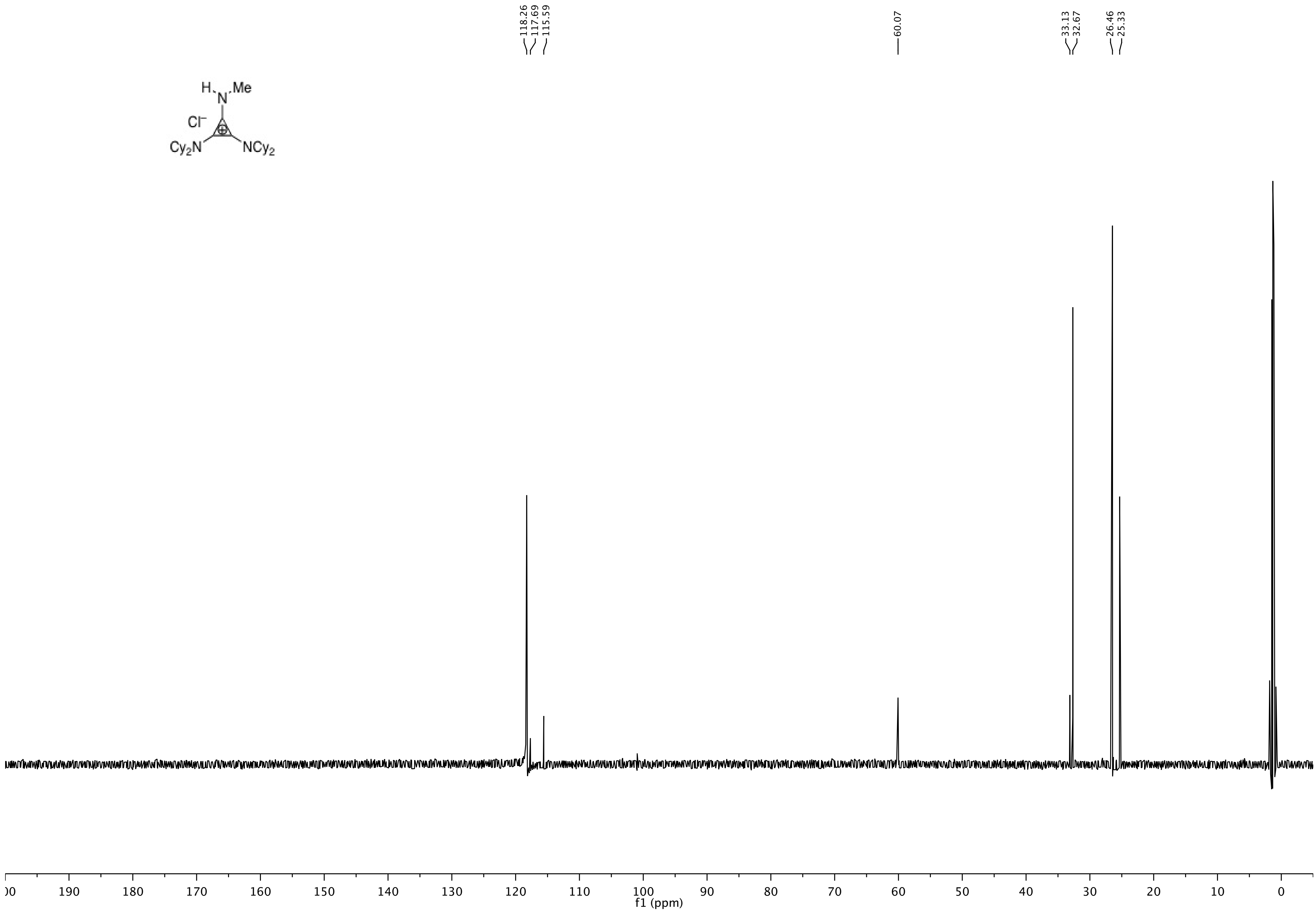
118.26
117.69
115.59
60.07
33.13
32.67
26.46
25.33
H
N
Me
Cl⁻
Cy2N
NCy2
00
190
180
170
160
150
140
130
120
110
100
90
80
70
60
50
40
30
20
10
0
f1 (ppm)

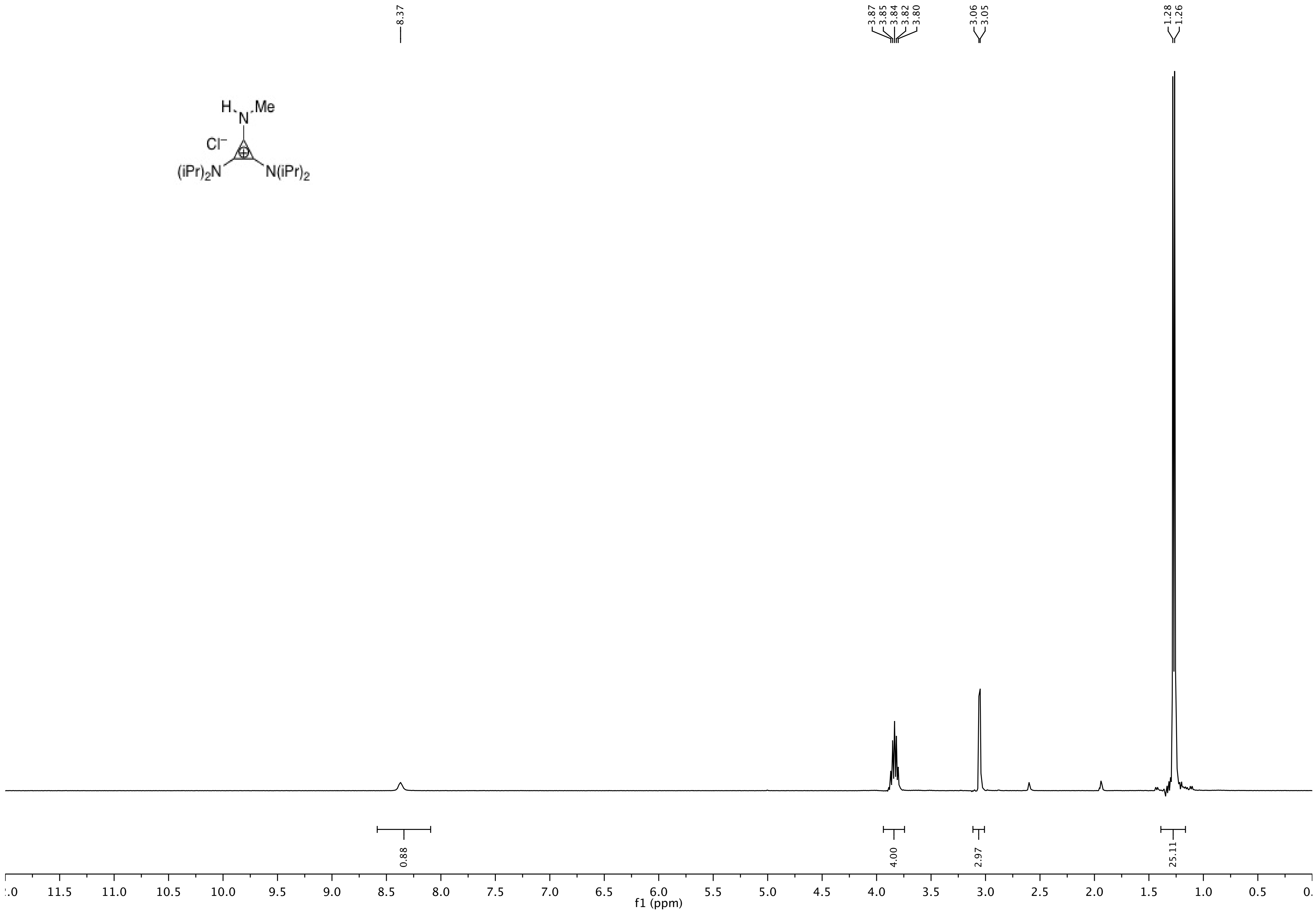

8.37
3.87
3.85
3.84
3.82
3.80
3.06
3.05
1.28
1.26
H
N
Me
Cl⁻
(iPr)2N
N(iPr)2
0.88
4.00
2.97
25.11
11.5 11.0 10.5 10.0 9.5 9.0 8.5 8.0 7.5 7.0 6.5 6.0 5.5 5.0 4.5 4.0 3.5 3.0 2.5 2.0 1.5 1.0 0.5
f1 (ppm)

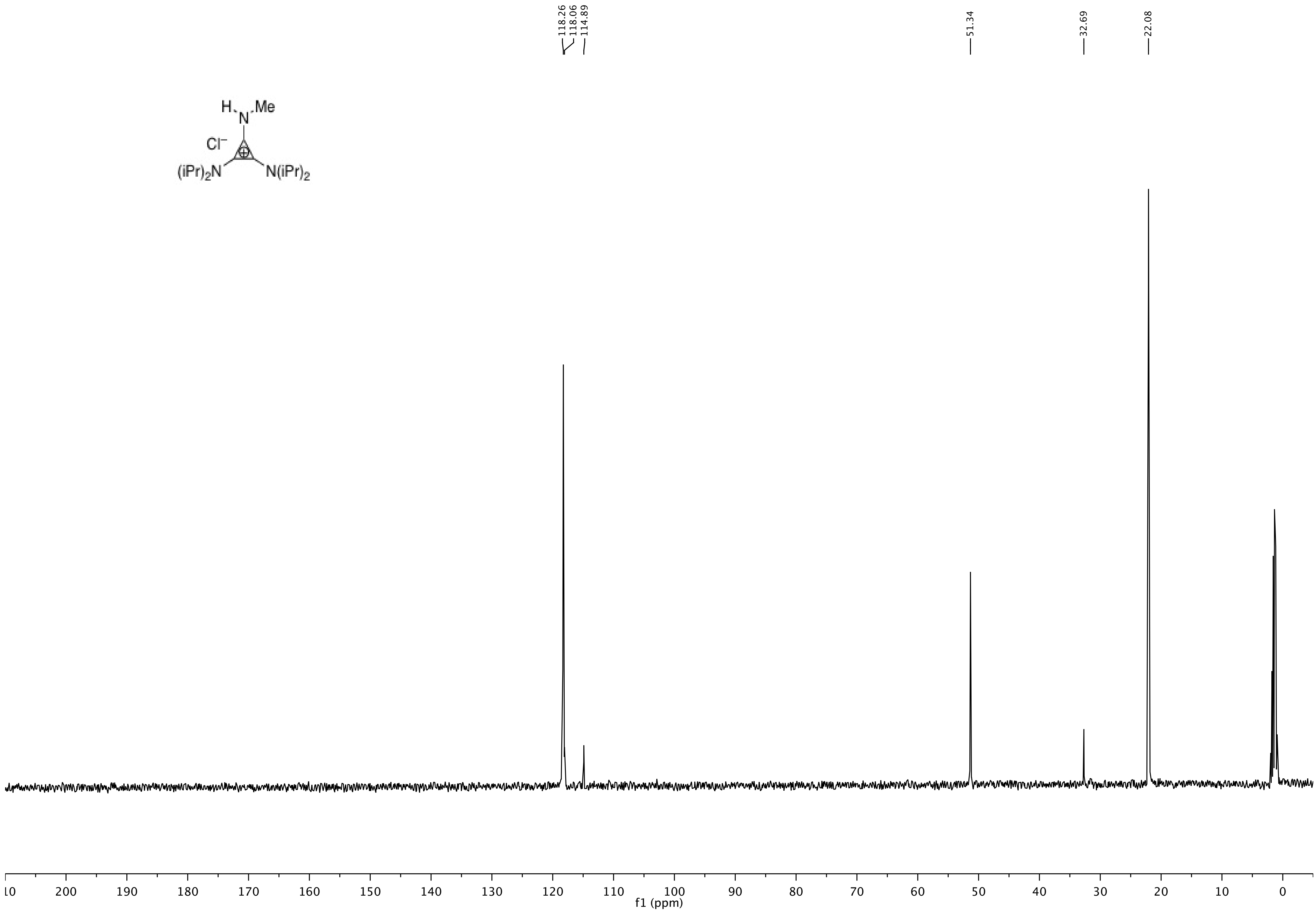

118.26
118.06
114.89
51.34
32.69
22.08
H
N
Me
Cl⁻
(iPr)2N
N(iPr)2
10
200
190
180
170
160
150
140
130
120
110
100
90
80
70
60
50
40
30
20
10
0
f1 (ppm)

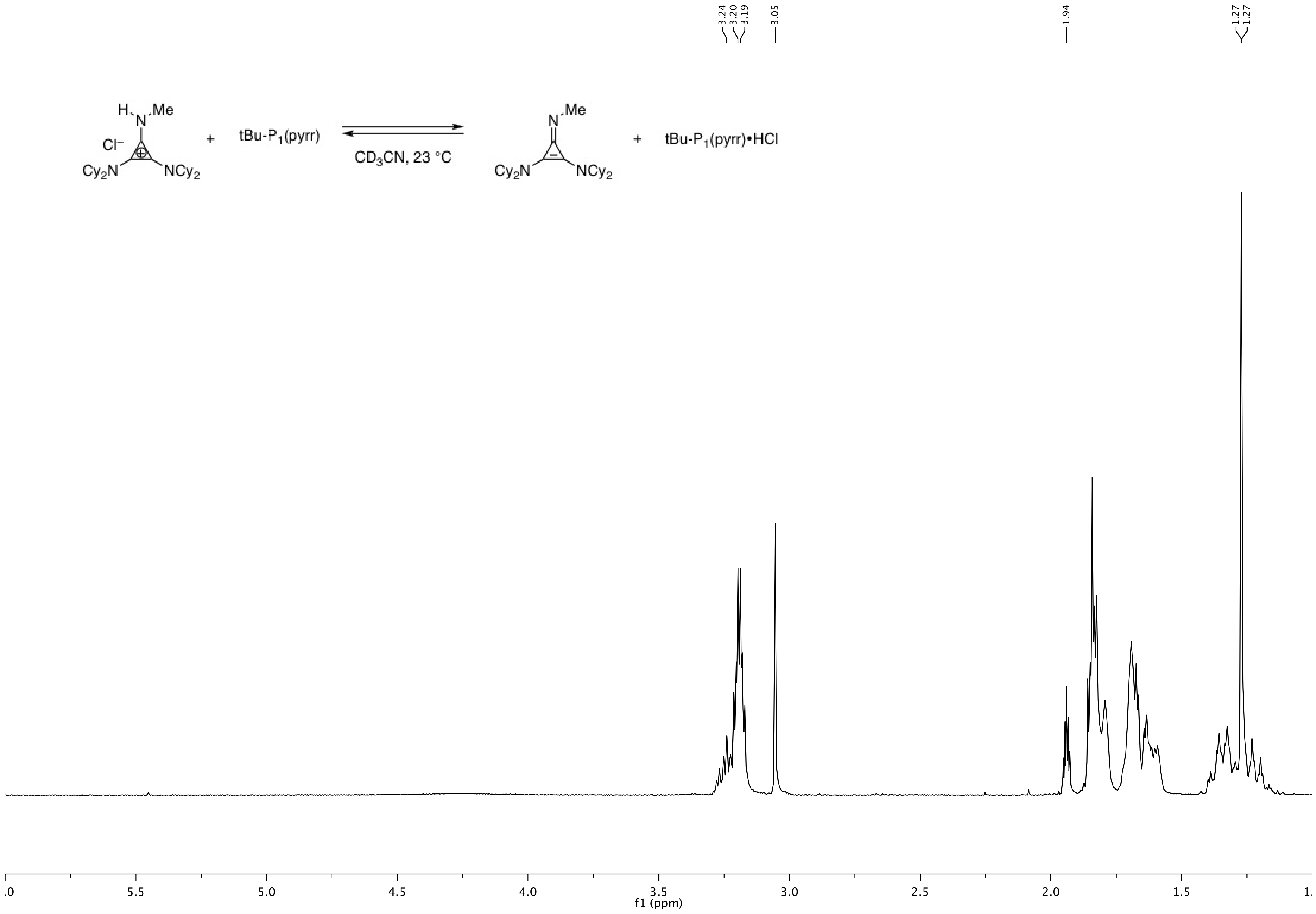

3.24
3.20
3.19
3.05
1.94
1.27
1.27
H
N
Me
Cl⁻
Cy2N
NCy2
+
tBu-P1(pyrr)
CD3CN, 23 °C
N
Me
Cy2N
NCy2
+
tBu-P1(pyrr)•HCl
.0
5.5
5.0
4.5
4.0
3.5
3.0
2.5
2.0
1.5
1.
f1 (ppm)

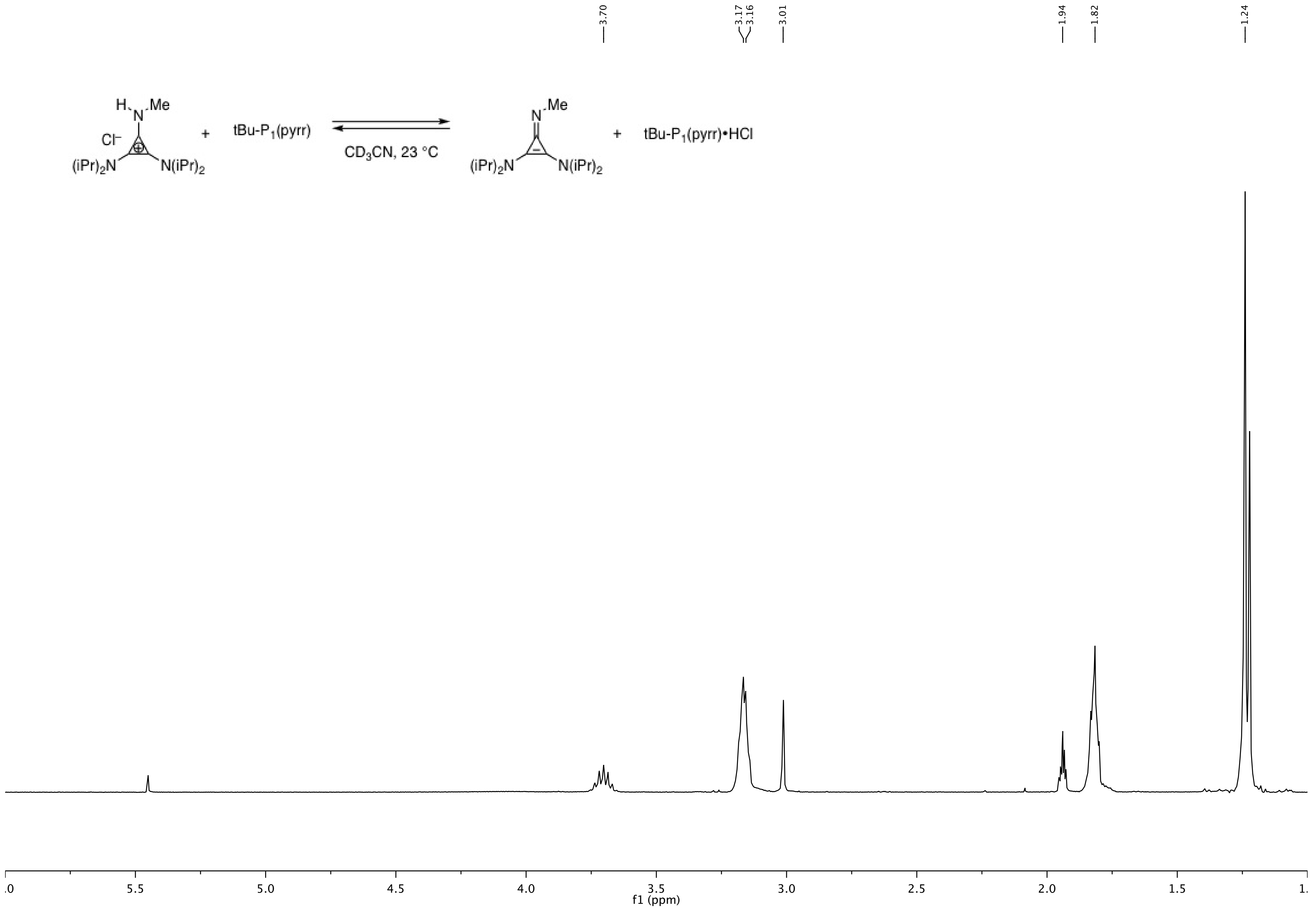

H
N
Me
Cl⁻
(iPr)2N
N(iPr)2
+
tBu-P1(pyrr)
CD3CN, 23 °C
N
Me
(iPr)2N
N(iPr)2
+
tBu-P1(pyrr)•HCl
3.70
3.17
3.16
3.01
1.94
1.82
1.24
.0
5.5
5.0
4.5
4.0
3.5
3.0
2.5
2.0
1.5
1.
f1 (ppm)

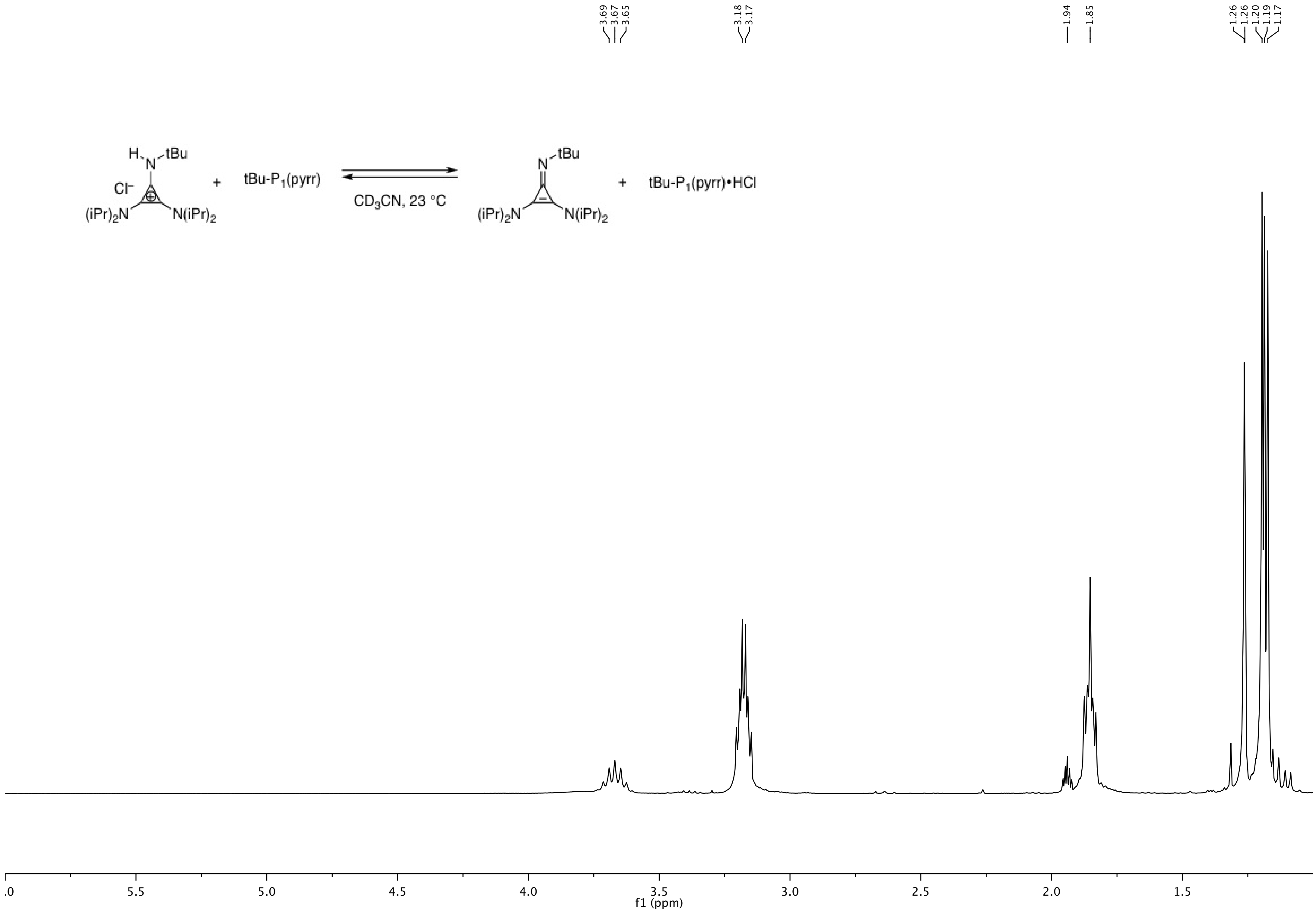
3.69
3.67
3.65
3.18
3.17
1.94
1.85
1.26
1.26
1.20
1.19
1.17
H
N
tBu
Cl⁻
(iPr)2N
N(iPr)2
+
tBu-P1(pyrr)
CD3CN, 23 °C
N
tBu
(iPr)2N
N(iPr)2
+
tBu-P1(pyrr)•HCl
.0
5.5
5.0
4.5
4.0
3.5
3.0
2.5
2.0
1.5
f1 (ppm)

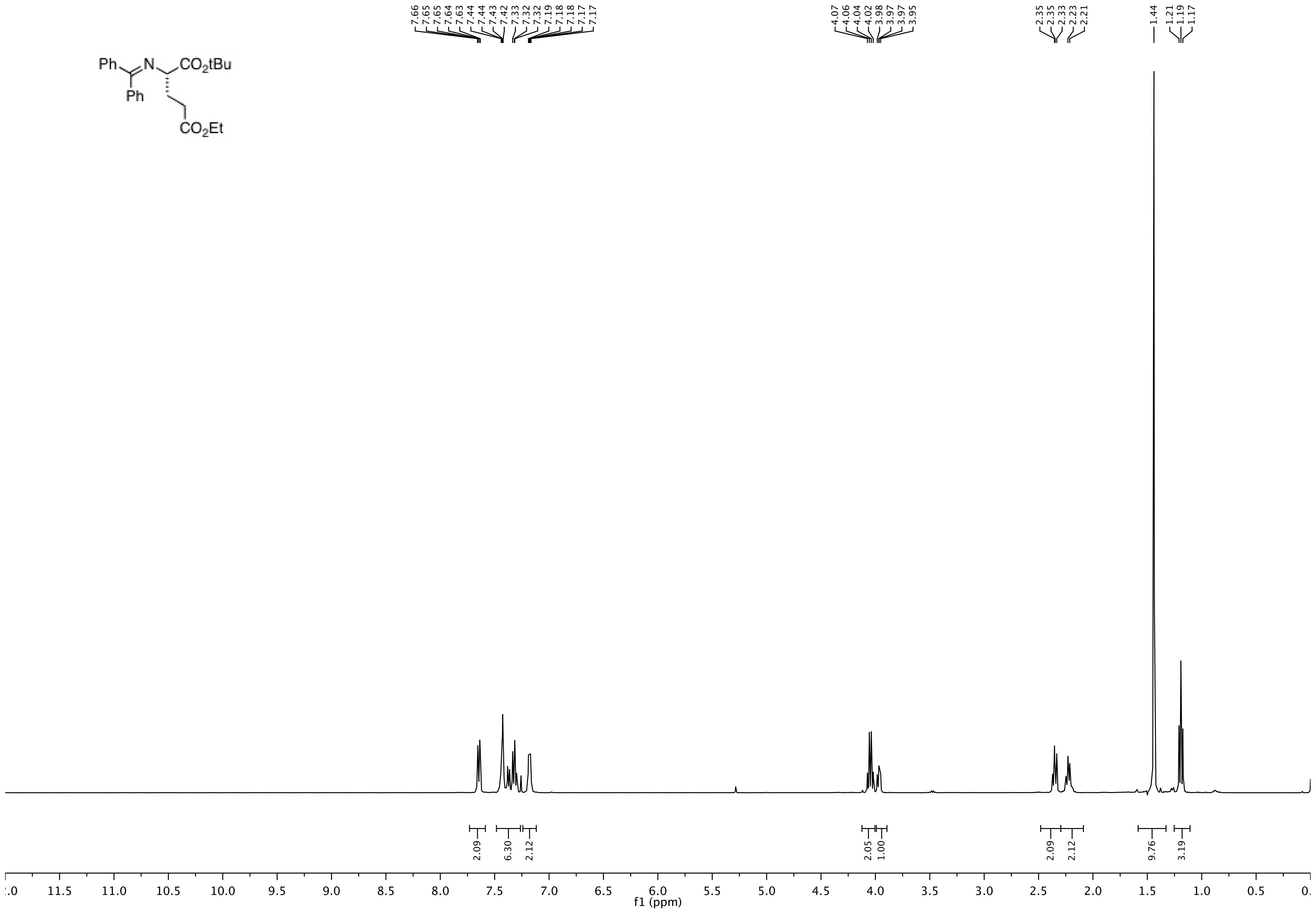
Ph
N
CO_2tBu
Ph
CO_2Et
7.66
7.65
7.65
7.64
7.63
7.44
7.44
7.43
7.42
7.33
7.32
7.32
7.19
7.18
7.18
7.17
7.17
4.07
4.06
4.04
4.02
3.98
3.97
3.97
3.95
2.35
2.35
2.33
2.23
2.21
1.44
1.21
1.19
1.17
2.09
6.30
2.12
2.05
1.00
2.09
2.12
9.76
3.19
f1 (ppm)

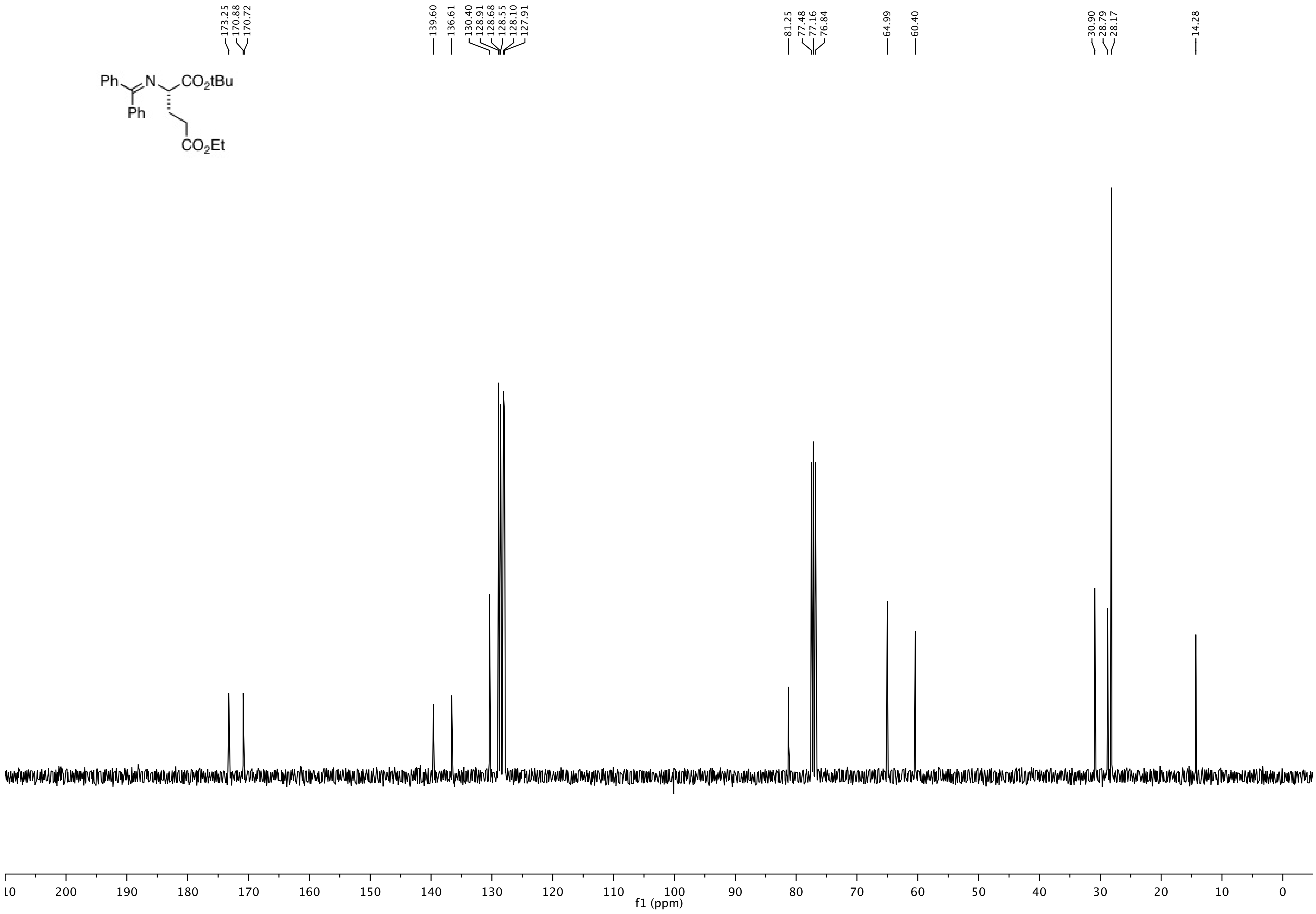

173.25
170.88
170.72
139.60
136.61
130.40
128.91
128.68
128.55
128.10
127.91
81.25
77.48
77.16
76.84
64.99
60.40
30.90
28.79
28.17
14.28
Ph
N
CO2tBu
Ph
CO2Et
200
190
180
170
160
150
140
130
120
110
100
90
80
70
60
50
40
30
20
10
0
f1 (ppm)

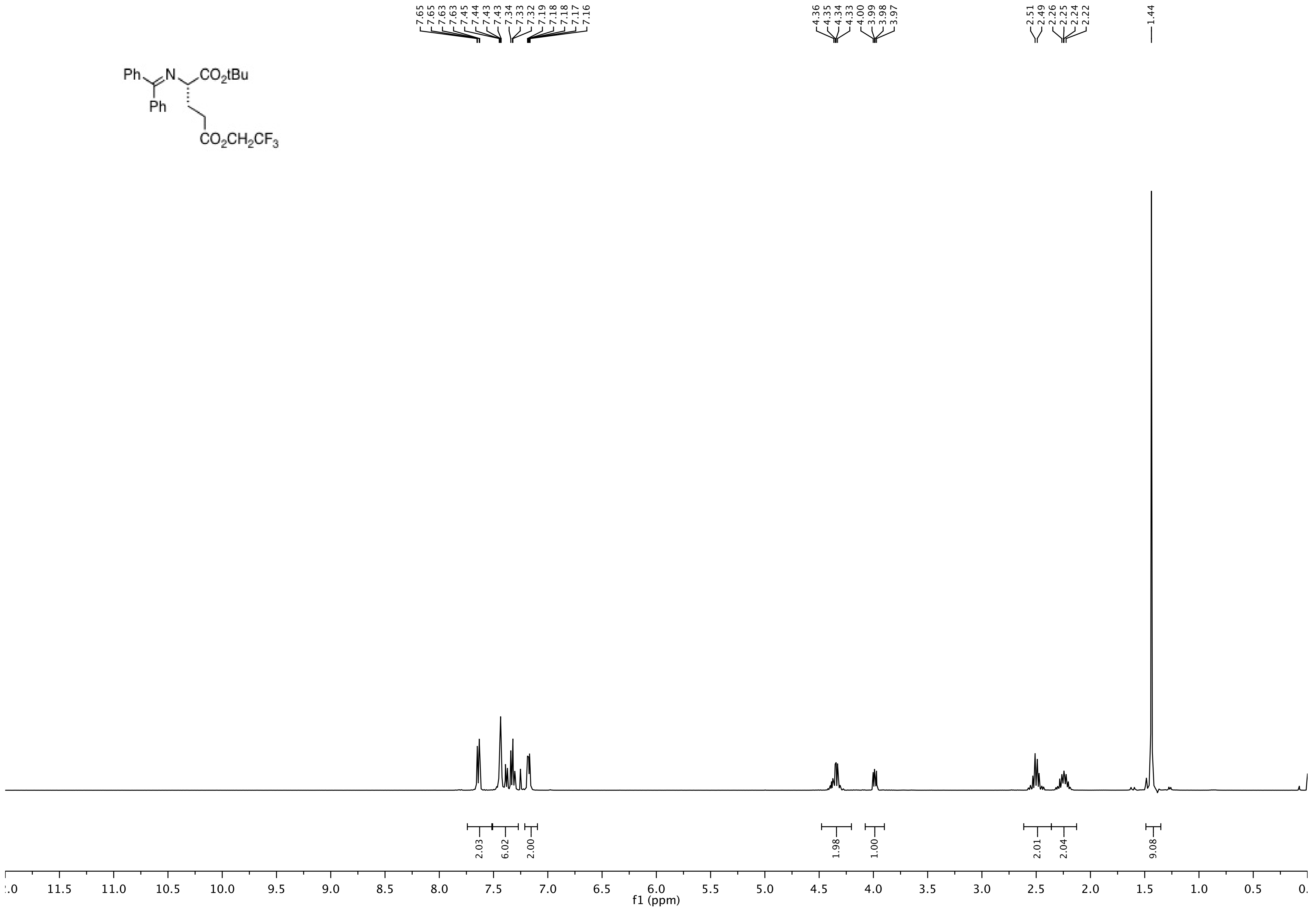

Ph
N
CO2tBu
Ph
CO2CH2CF3
7.65 7.65 7.63 7.63 7.45 7.44 7.43 7.43 7.34 7.33 7.32 7.19 7.18 7.18 7.17 7.16
4.36 4.35 4.34 4.33 4.00 3.99 3.98 3.97
2.51 2.49 2.26 2.25 2.24 2.22
1.44
2.03 6.02 2.00 1.98 1.00 2.01 2.04 9.08
11.5 11.0 10.5 10.0 9.5 9.0 8.5 8.0 7.5 7.0 6.5 6.0 5.5 5.0 4.5 4.0 3.5 3.0 2.5 2.0 1.5 1.0 0.5
f1 (ppm)

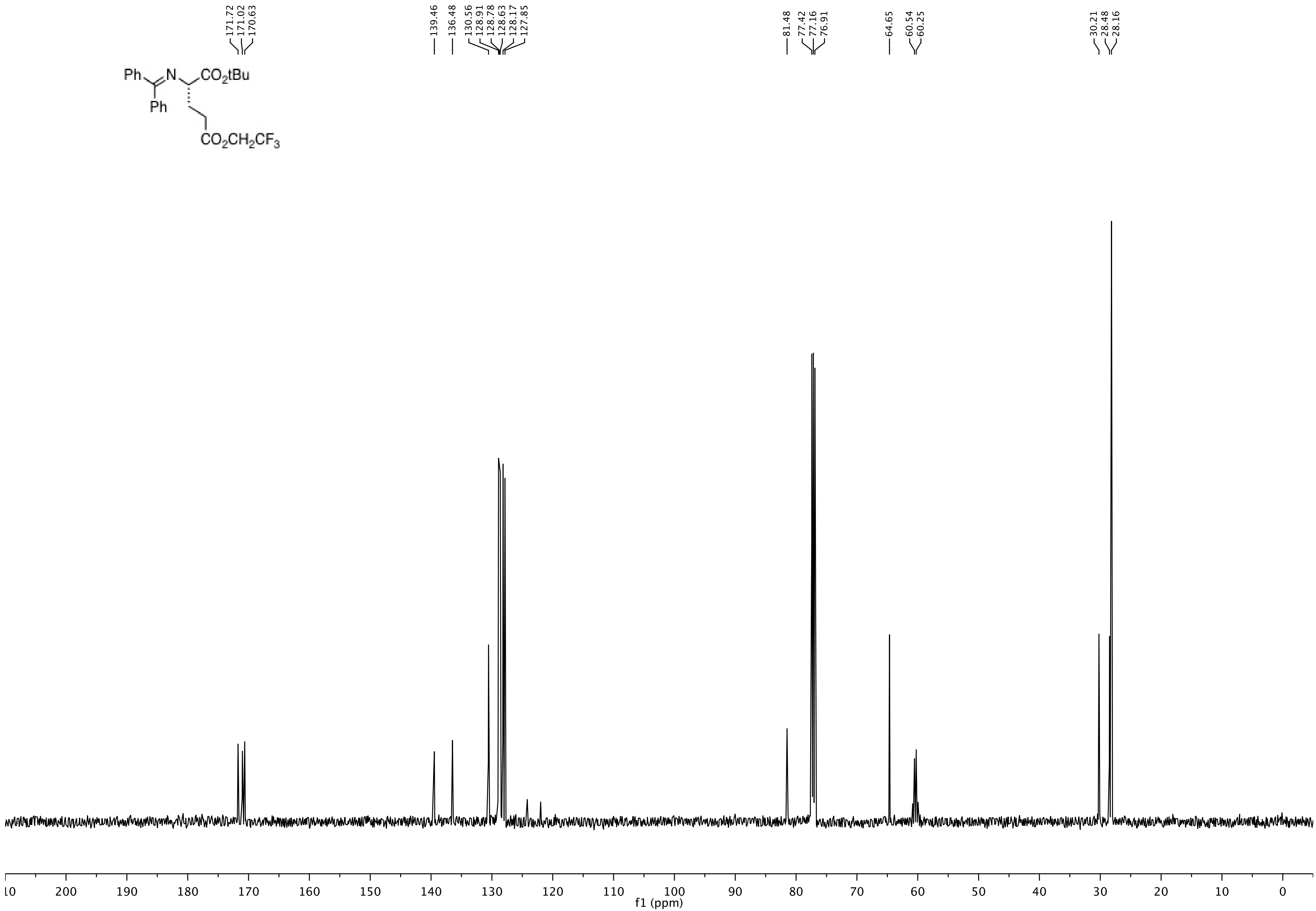
171.72
171.02
170.63
139.46
136.48
130.56
128.91
128.78
128.63
128.17
127.85
81.48
77.42
77.16
76.91
64.65
60.54
60.25
30.21
28.48
28.16
Ph
N
CO2tBu
Ph
CO2CH2CF3
f1 (ppm)

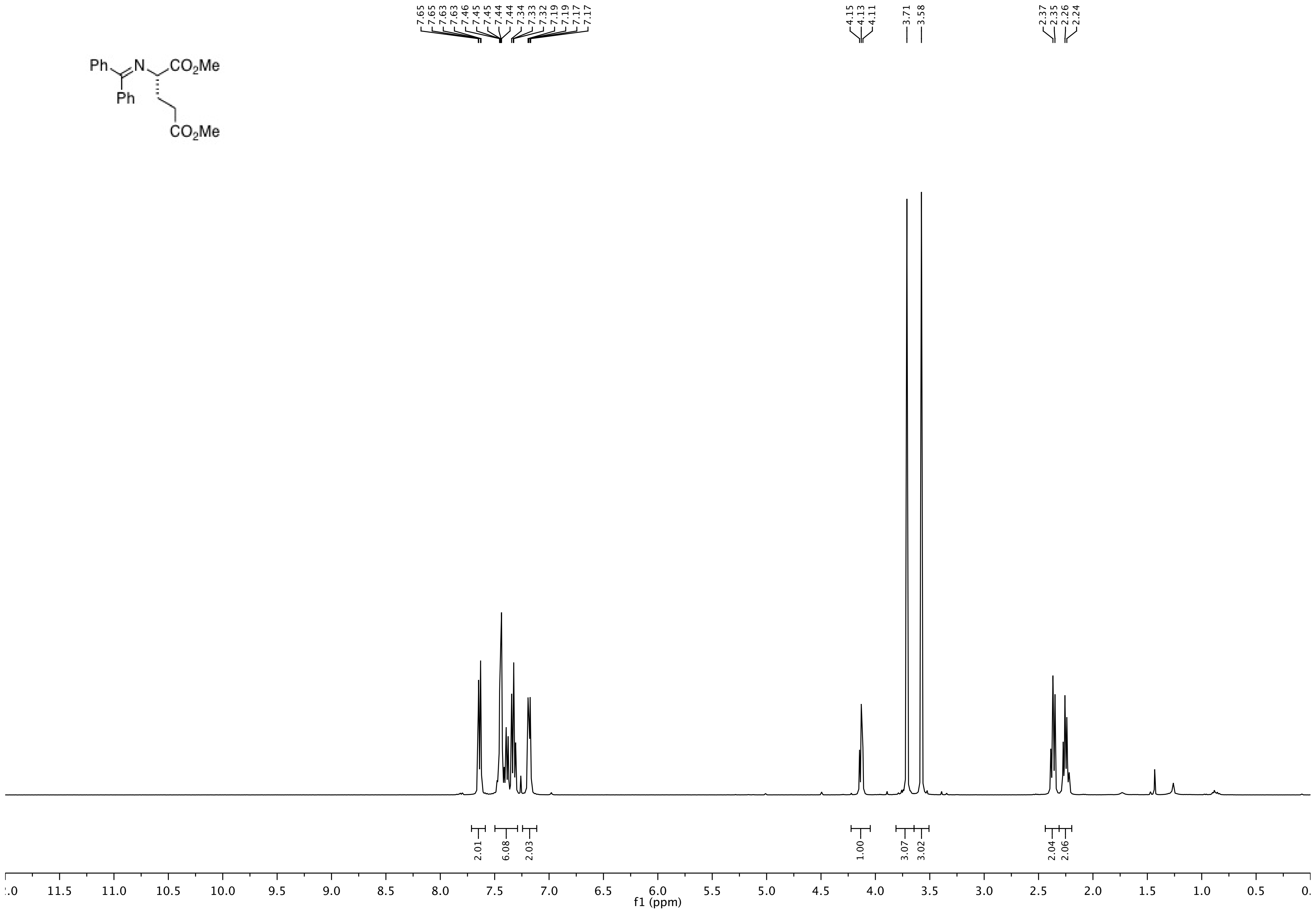

Ph
N
CO2Me
Ph
CO2Me
7.65
7.65
7.63
7.63
7.46
7.45
7.45
7.44
7.44
7.34
7.33
7.32
7.19
7.19
7.17
7.17
4.15
4.13
4.11
3.71
3.58
2.37
2.35
2.26
2.24
2.01
6.08
2.03
1.00
3.07
3.02
2.04
2.06
11.5
11.0
10.5
10.0
9.5
9.0
8.5
8.0
7.5
7.0
6.5
6.0
5.5
5.0
4.5
4.0
3.5
3.0
2.5
2.0
1.5
1.0
0.5
f1 (ppm)

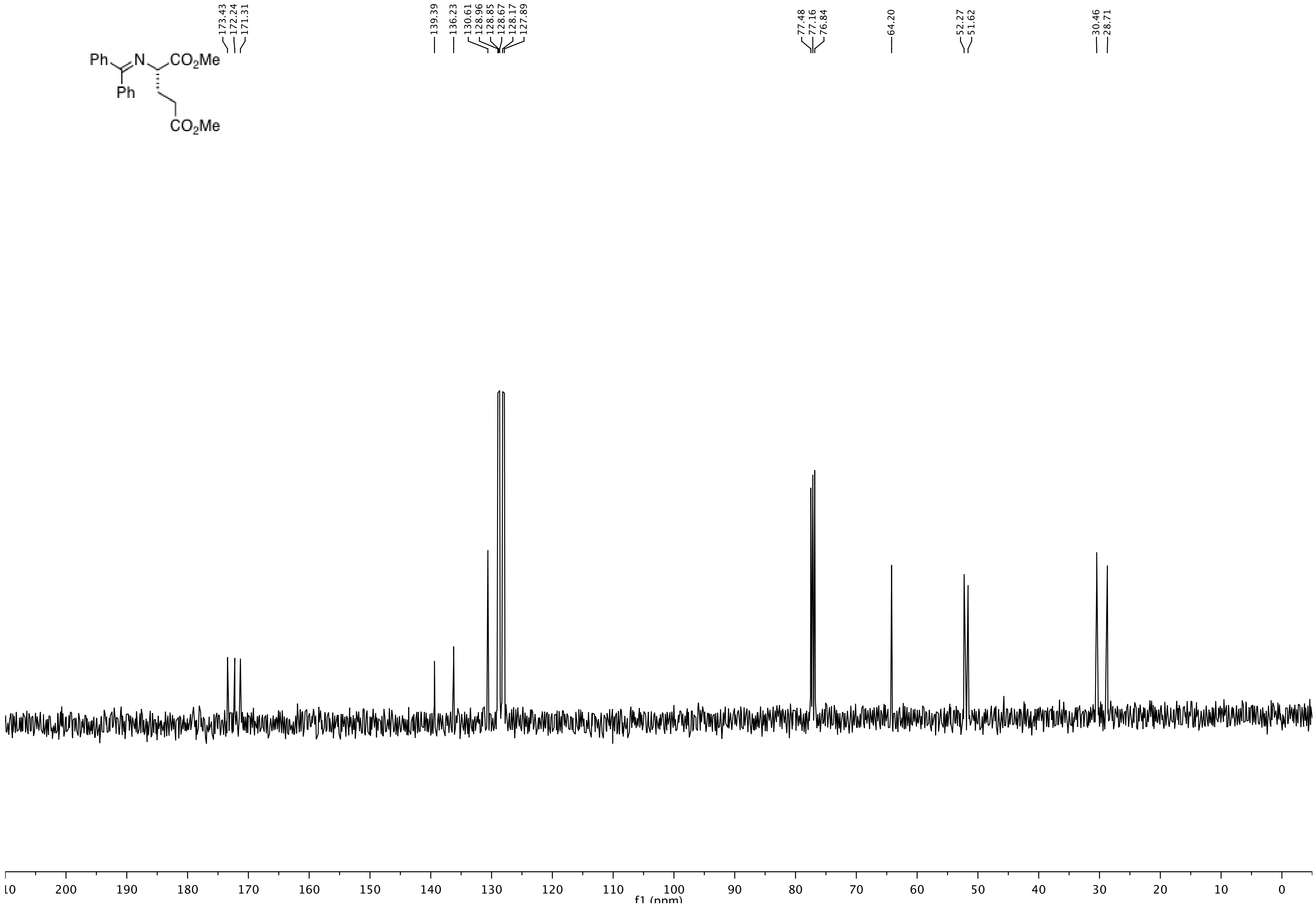

Ph
N
CO2Me
Ph
CO2Me
173.43
172.24
171.31
139.39
136.23
130.61
128.96
128.85
128.67
128.17
127.89
77.48
77.16
76.84
64.20
52.27
51.62
30.46
28.71
200
190
180
170
160
150
140
130
120
110
100
90
80
70
60
50
40
30
20
10
0
f1 (ppm)

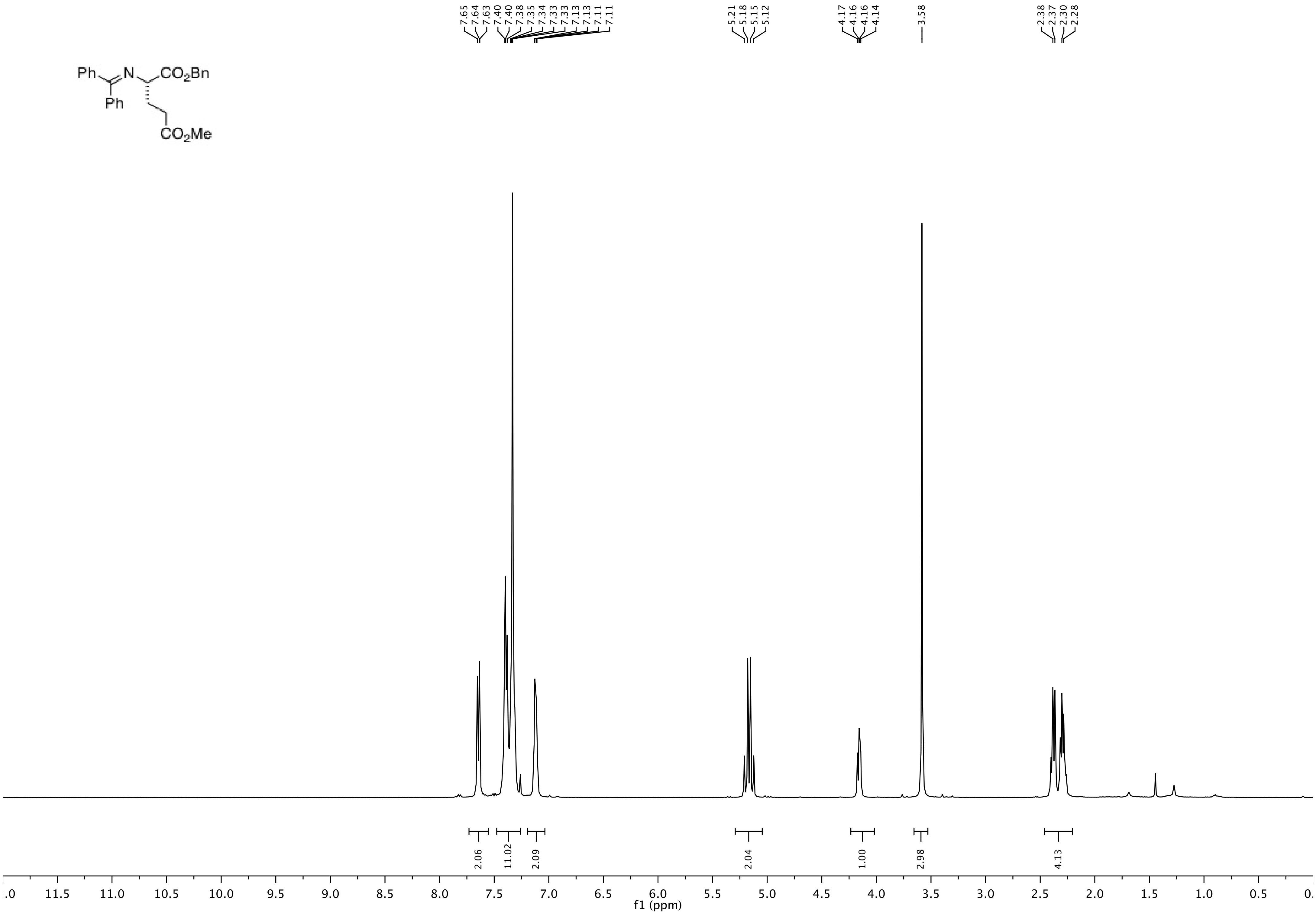
Ph N CO2Bn
Ph
CO2Me
7.65 7.64 7.63 7.40 7.40 7.38 7.35 7.34 7.33 7.33 7.13 7.13 7.11 7.11
5.21 5.18 5.15 5.12
4.17 4.16 4.16 4.14
3.58
2.38 2.37 2.30 2.28
2.06 11.02 2.09 2.04 1.00 2.98 4.13
11.5 11.0 10.5 10.0 9.5 9.0 8.5 8.0 7.5 7.0 6.5 6.0 5.5 5.0 4.5 4.0 3.5 3.0 2.5 2.0 1.5 1.0 0.5
f1 (ppm)

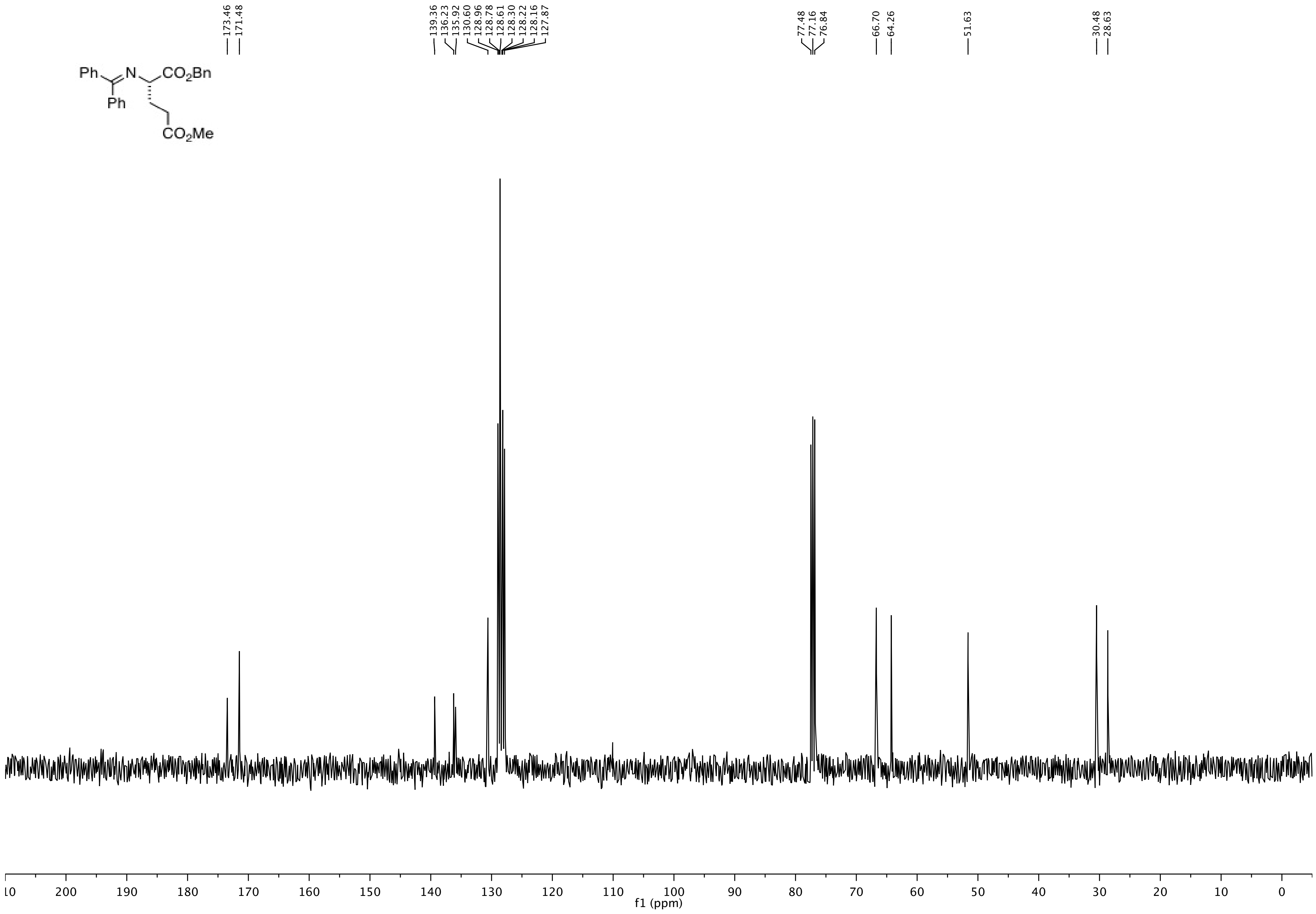
173.46
171.48
139.36
136.23
135.92
130.60
128.96
128.78
128.61
128.30
128.22
128.16
127.87
77.48
77.16
76.84
66.70
64.26
51.63
30.48
28.63
Ph
N
CO2Bn
Ph
CO2Me
200
190
180
170
160
150
140
130
120
110
100
90
80
70
60
50
40
30
20
10
0
f1 (ppm)

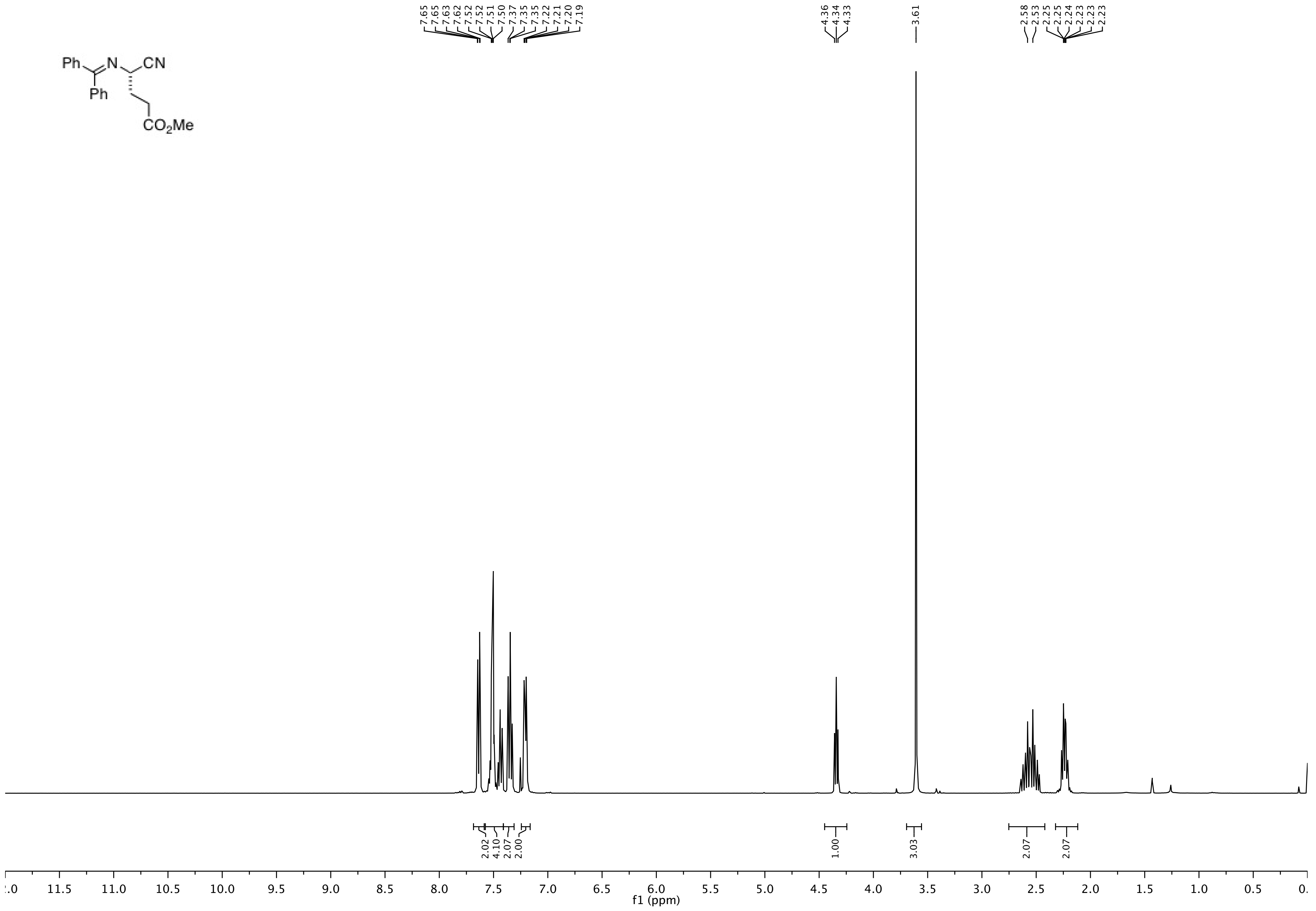
Ph
N
CN
Ph
CO2Me
7.65 7.65 7.63 7.62 7.52 7.52 7.51 7.50 7.37 7.35 7.35 7.22 7.21 7.20 7.19
4.36 4.34 4.33
3.61
2.58 2.53 2.25 2.25 2.24 2.23 2.23 2.23
2.02 4.10 2.07 2.00
1.00
3.03
2.07
2.07
11.5 11.0 10.5 10.0 9.5 9.0 8.5 8.0 7.5 7.0 6.5 6.0 5.5 5.0 4.5 4.0 3.5 3.0 2.5 2.0 1.5 1.0 0.5
f1 (ppm)

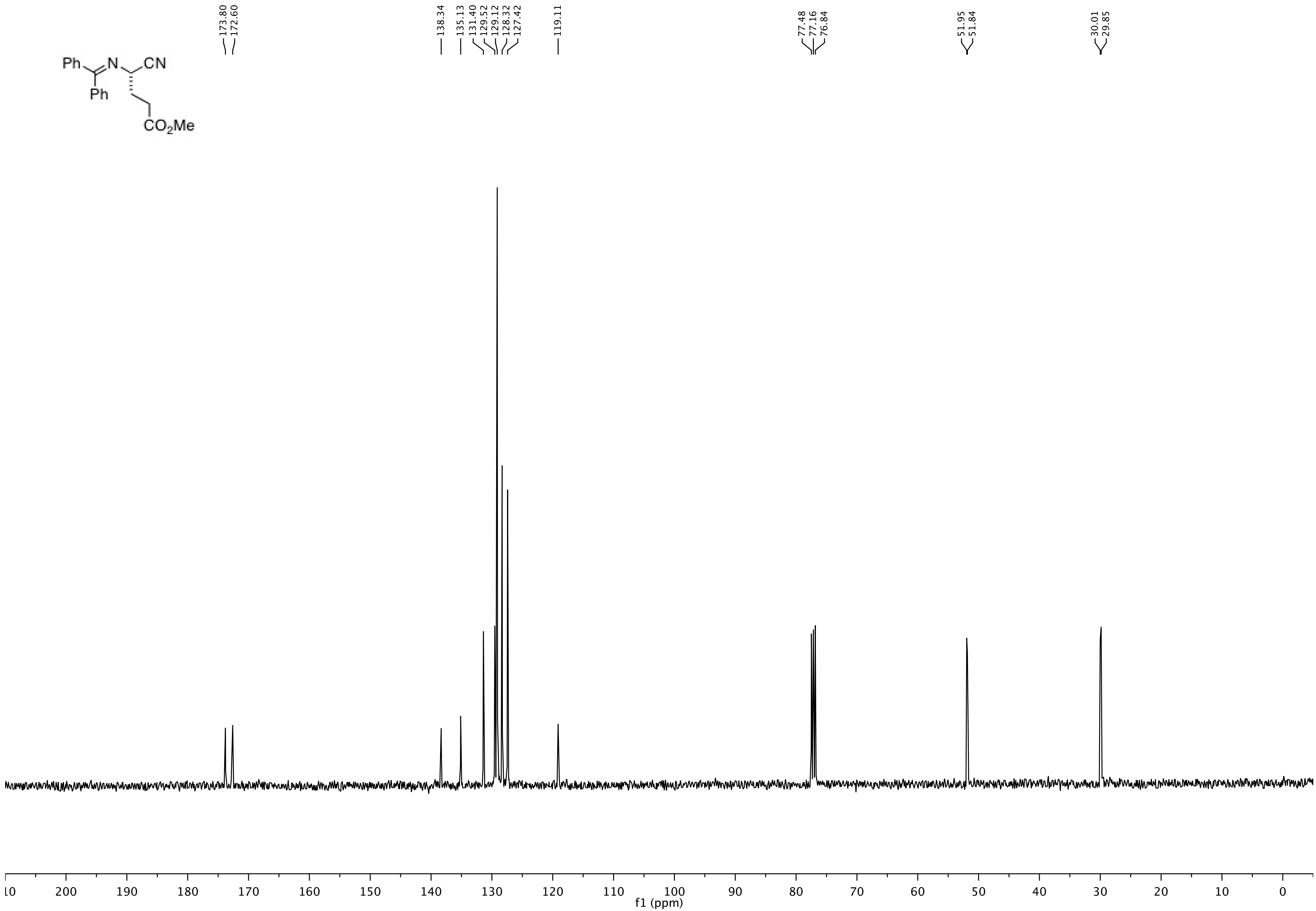

173.80
172.60
138.34
135.13
131.40
129.52
129.12
128.32
127.42
119.11
77.48
77.16
76.84
51.95
51.84
30.01
29.85
Ph
N
CN
Ph
CO_2Me
10
200
190
180
170
160
150
140
130
120
110
100
90
80
70
60
50
40
30
20
10
0
f1 (ppm)

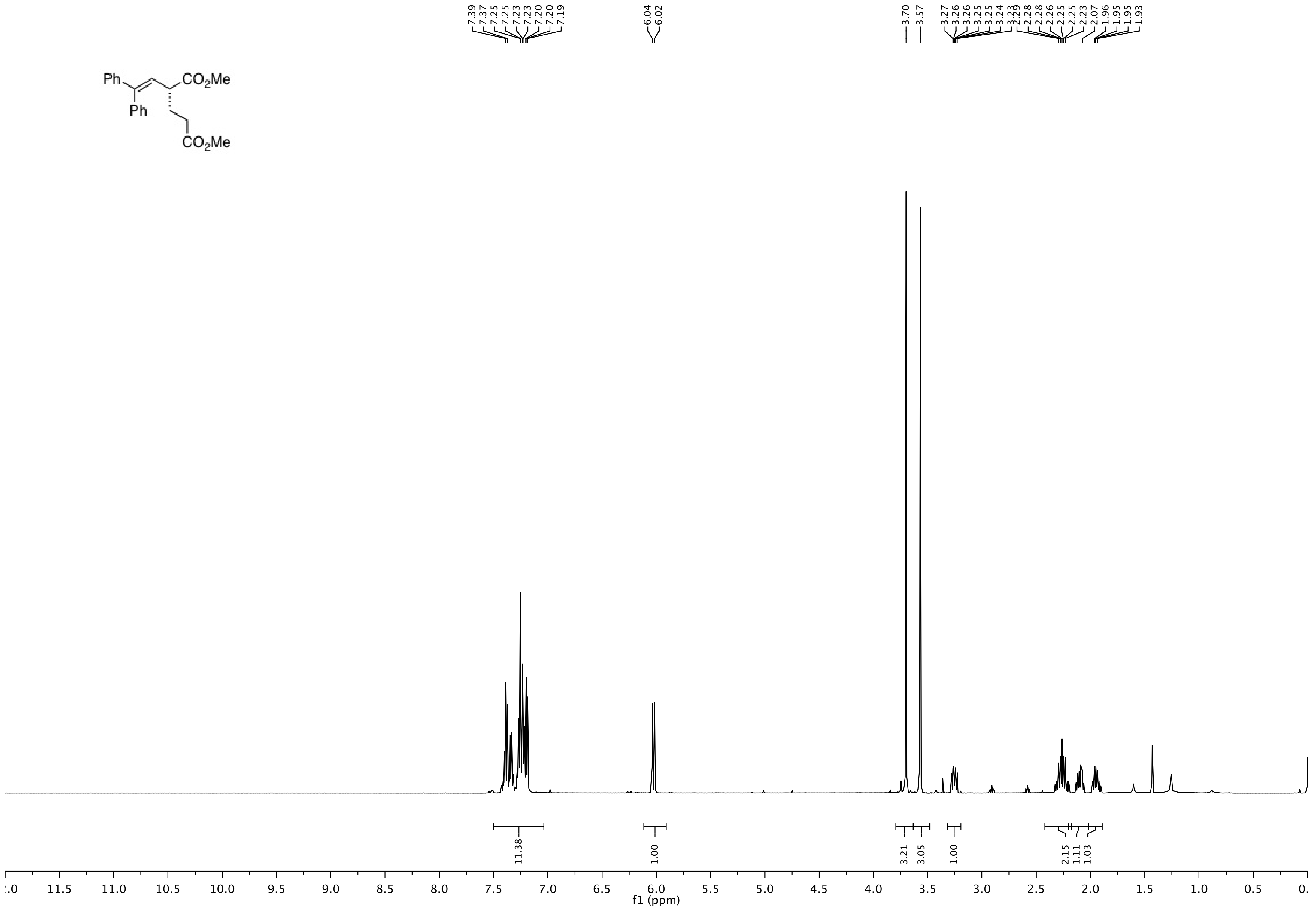

Ph
Ph
CO2Me
CO2Me
7.39
7.37
7.25
7.25
7.23
7.23
7.20
7.20
7.19
6.04
6.02
3.70
3.57
3.27
3.26
3.25
3.25
3.24
3.23
2.29
2.28
2.28
2.26
2.25
2.25
2.23
2.07
1.96
1.95
1.95
1.93
11.38
1.00
3.21
3.05
1.00
2.15
1.11
1.03
11.5
11.0
10.5
10.0
9.5
9.0
8.5
8.0
7.5
7.0
6.5
6.0
5.5
5.0
4.5
4.0
3.5
3.0
2.5
2.0
1.5
1.0
0.5
f1 (ppm)

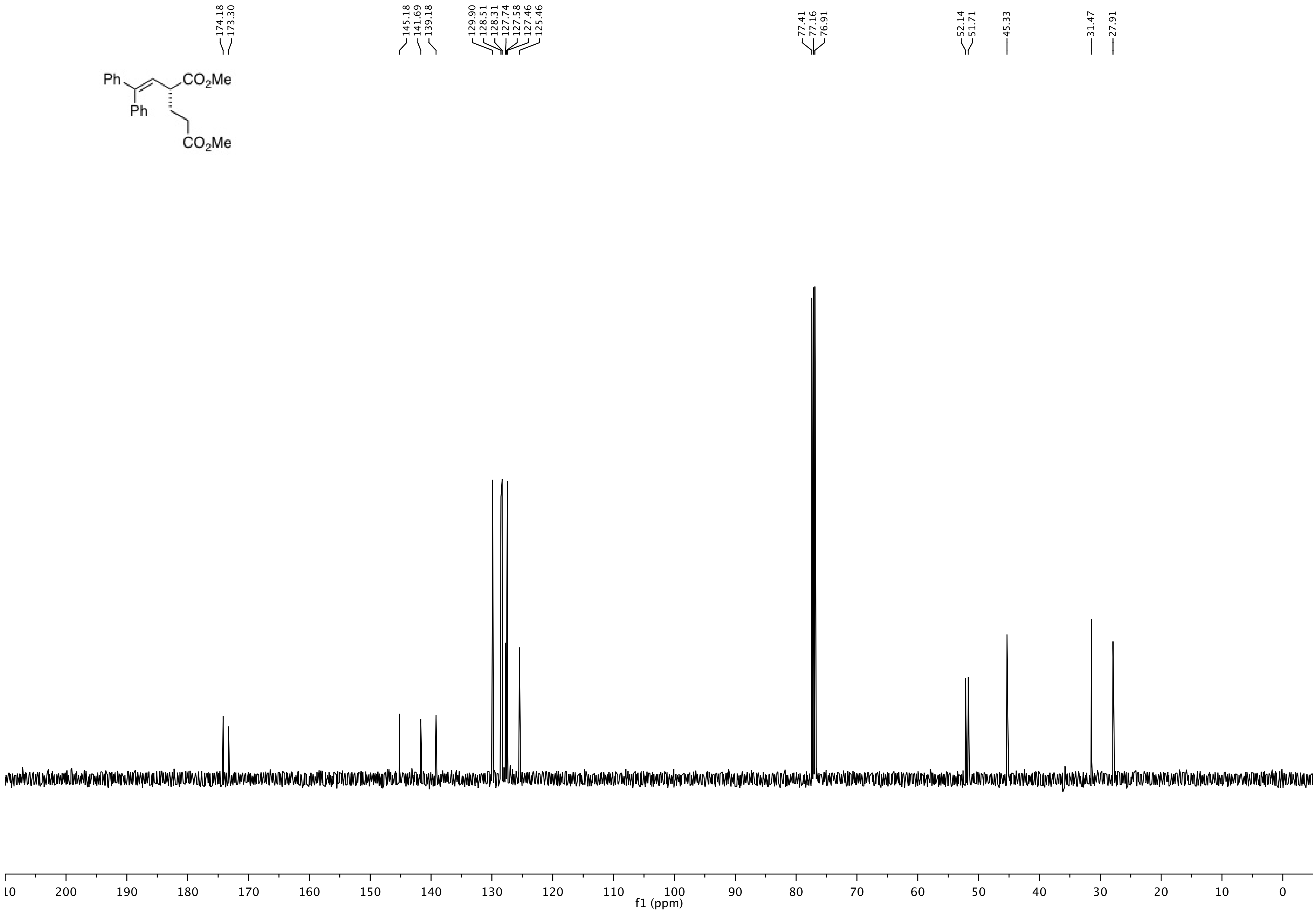

174.18
173.30
145.18
141.69
139.18
129.90
128.51
128.31
127.74
127.58
127.46
125.46
77.41
77.16
76.91
52.14
51.71
45.33
31.47
27.91
Ph
Ph
CO2Me
CO2Me
f1 (ppm)

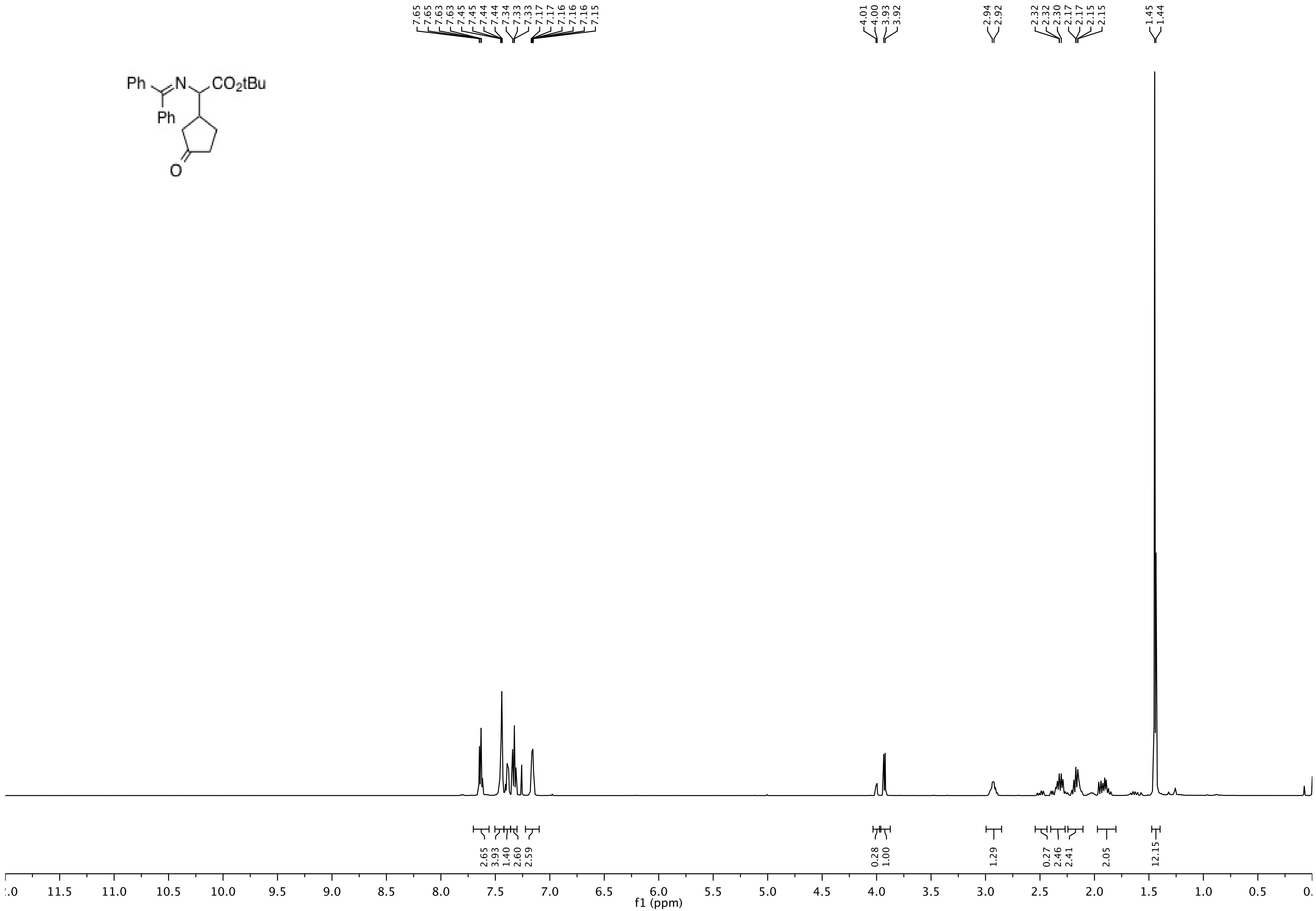

7.65
7.65
7.63
7.63
7.45
7.45
7.44
7.44
7.34
7.33
7.33
7.17
7.17
7.16
7.16
7.15
4.01
4.00
3.93
3.92
2.94
2.92
2.32
2.32
2.30
2.17
2.17
2.15
2.15
1.45
1.44
Ph
N
CO2tBu
Ph
O
2.65
3.93
1.40
2.60
2.59
0.28
1.00
1.29
0.27
2.46
2.41
2.05
12.15
11.5
11.0
10.5
10.0
9.5
9.0
8.5
8.0
7.5
7.0
6.5
6.0
5.5
5.0
4.5
4.0
3.5
3.0
2.5
2.0
1.5
1.0
0.5
f1 (ppm)

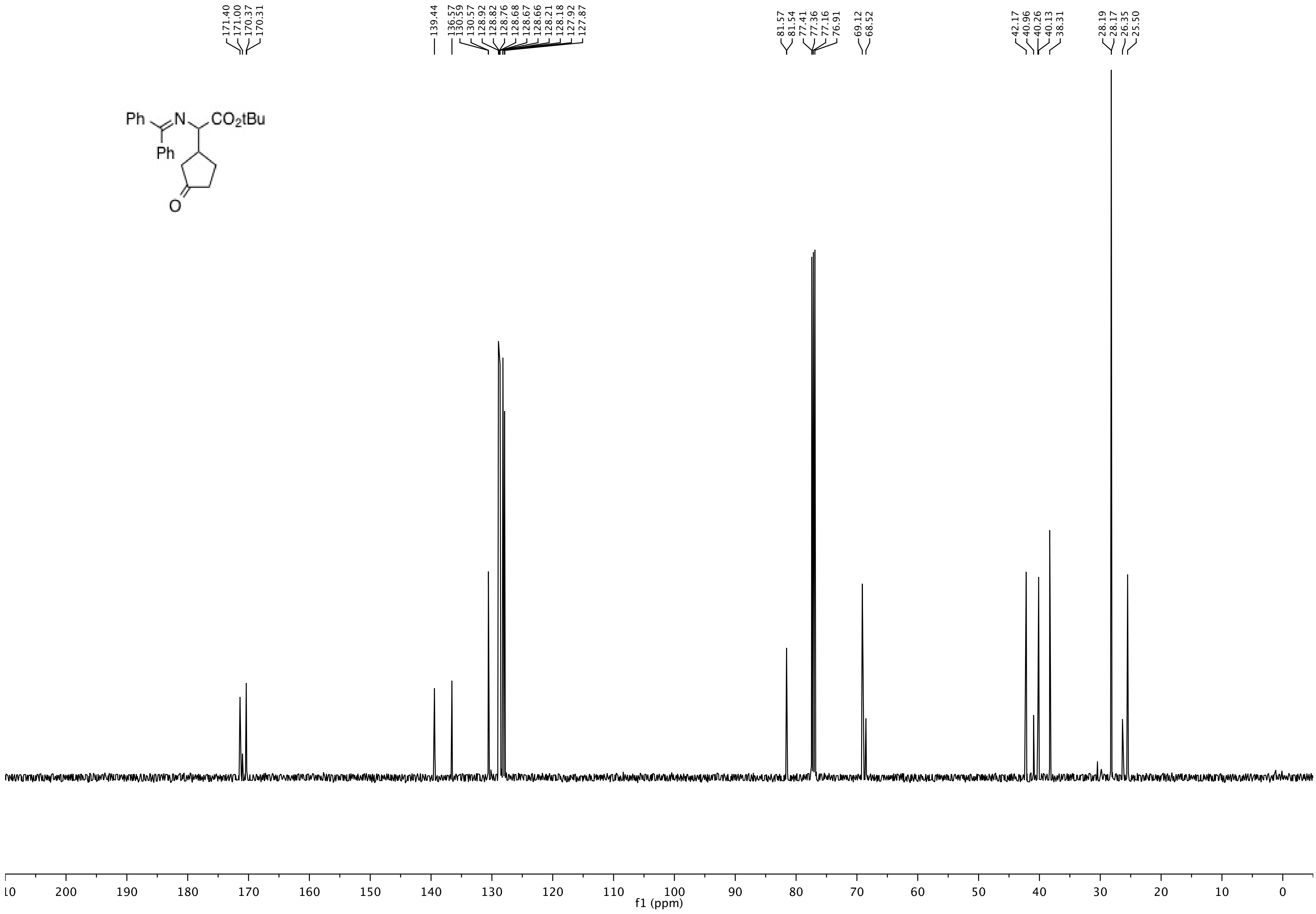
171.40
171.00
170.37
170.31
139.44
136.57
130.59
130.57
128.92
128.82
128.76
128.68
128.67
128.66
128.21
128.18
127.92
127.87
81.57
81.54
77.41
77.36
77.16
76.91
69.12
68.52
42.17
40.96
40.26
40.13
38.31
28.19
28.17
26.35
25.50
Ph
N
CO_2tBu
Ph
O
200
190
180
170
160
150
140
130
120
110
100
90
80
70
60
50
40
30
20
10
0
f1 (ppm)

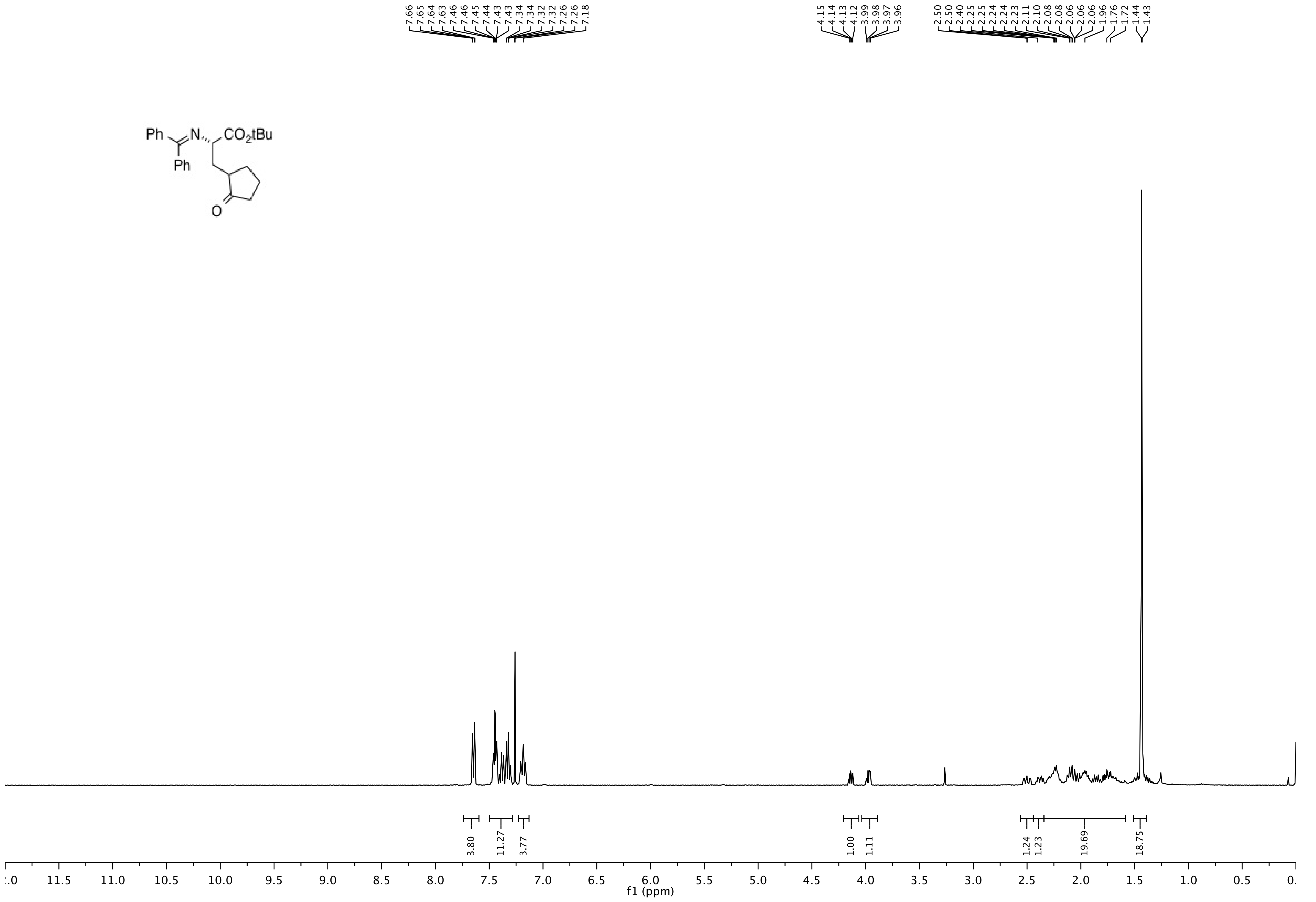

7.66
7.65
7.64
7.63
7.46
7.45
7.44
7.43
7.43
7.34
7.34
7.32
7.32
7.26
7.26
7.18
4.15
4.14
4.13
4.12
3.99
3.98
3.97
3.96
2.50
2.50
2.40
2.25
2.25
2.24
2.24
2.23
2.11
2.10
2.08
2.08
2.06
2.06
2.06
1.96
1.76
1.72
1.44
1.43
Ph
N
CO2tBu
Ph
O
3.80
11.27
3.77
1.00
1.11
1.24
1.23
19.69
18.75
11.5
11.0
10.5
10.0
9.5
9.0
8.5
8.0
7.5
7.0
6.5
6.0
5.5
5.0
4.5
4.0
3.5
3.0
2.5
2.0
1.5
1.0
0.5
f1 (ppm)

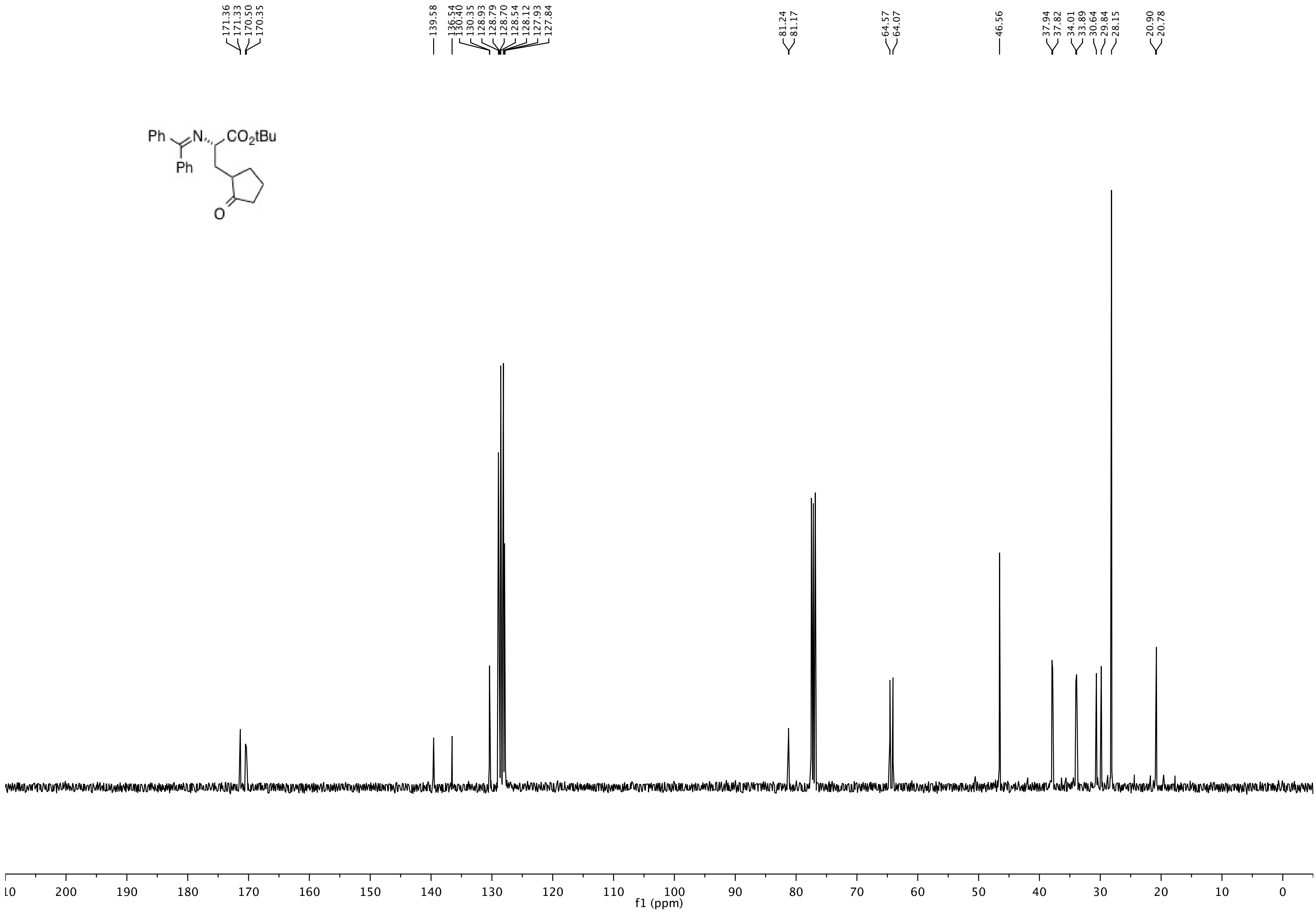
171.36
171.33
170.50
170.35
139.58
136.54
130.40
130.35
128.93
128.79
128.70
128.54
128.12
127.93
127.84
81.24
81.17
64.57
64.07
46.56
37.94
37.82
34.01
33.89
30.64
29.84
28.15
20.90
20.78
Ph
N
CO_2tBu
Ph
O
200
190
180
170
160
150
140
130
120
110
100
90
80
70
60
50
40
30
20
10
0
f1 (ppm)

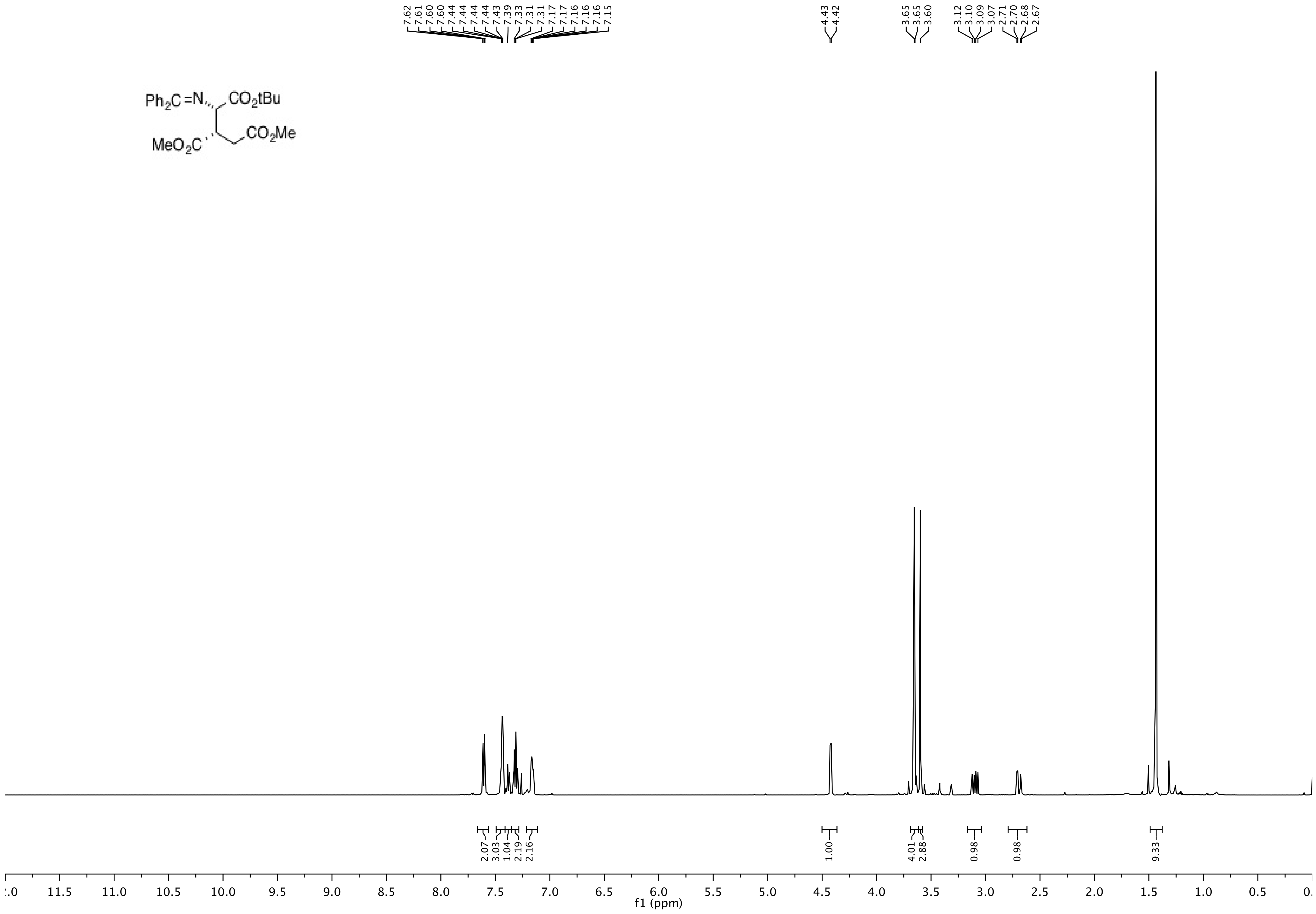

Ph2C=N CO2tBu
MeO2C CO2Me
7.62 7.61 7.60 7.44 7.44 7.44 7.44 7.43 7.39 7.33 7.31 7.31 7.17 7.17 7.16 7.16 7.15
4.43 4.42
3.65 3.65 3.60
3.12 3.10 3.09 3.07 2.71 2.70 2.68 2.67
2.07 3.03 1.04 2.19 2.16
1.00
4.01 2.88
0.98
0.98
9.33
11.5 11.0 10.5 10.0 9.5 9.0 8.5 8.0 7.5 7.0 6.5 6.0 5.5 5.0 4.5 4.0 3.5 3.0 2.5 2.0 1.5 1.0 0.5
f1 (ppm)

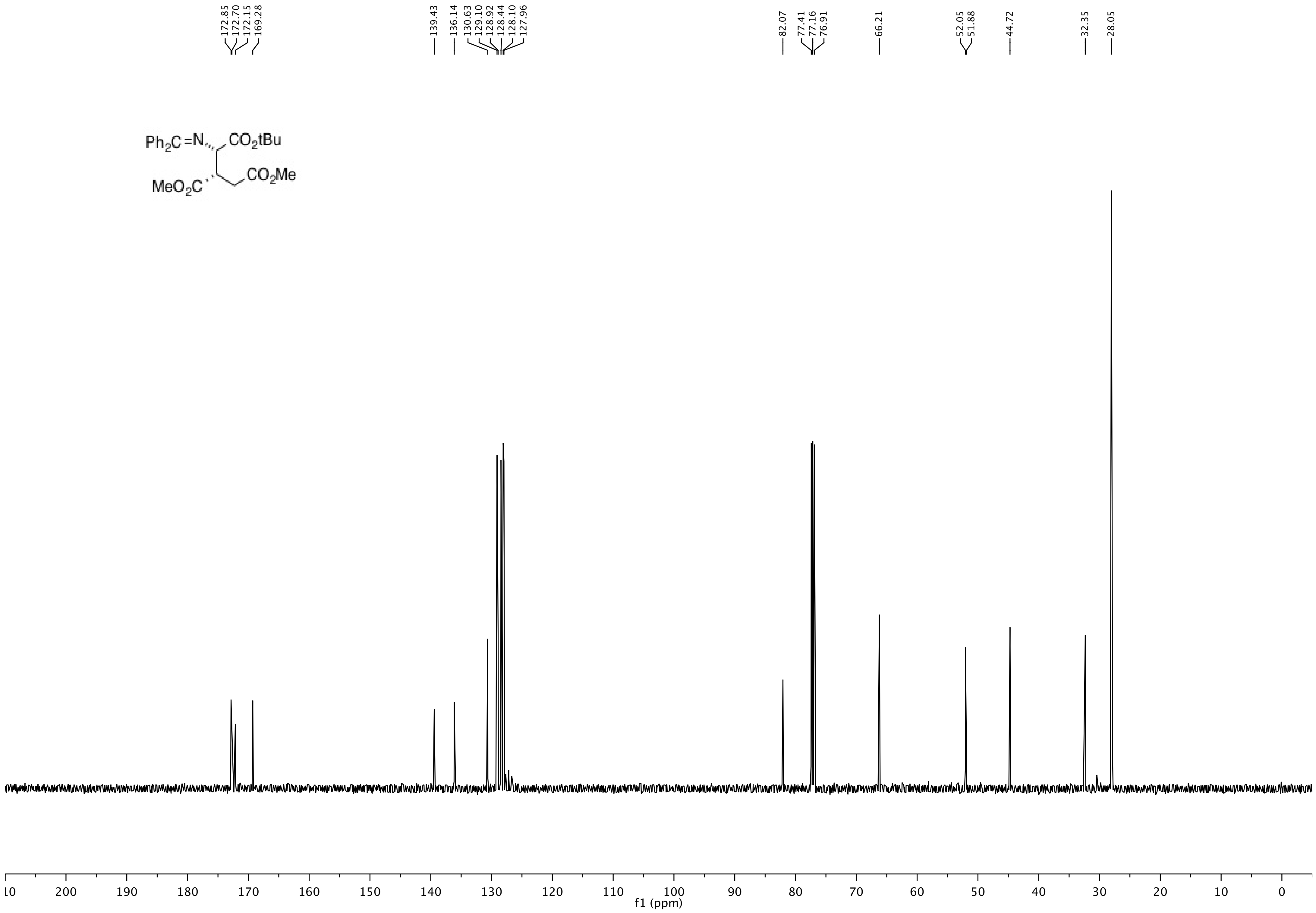
172.85
172.70
172.15
169.28
139.43
136.14
130.63
129.10
128.92
128.44
128.10
127.96
82.07
77.41
77.16
76.91
66.21
52.05
51.88
44.72
32.35
28.05
Ph2C=N CO2tBu
MeO2C CO2Me
200
190
180
170
160
150
140
130
120
110
100
90
80
70
60
50
40
30
20
10
0
f1 (ppm)

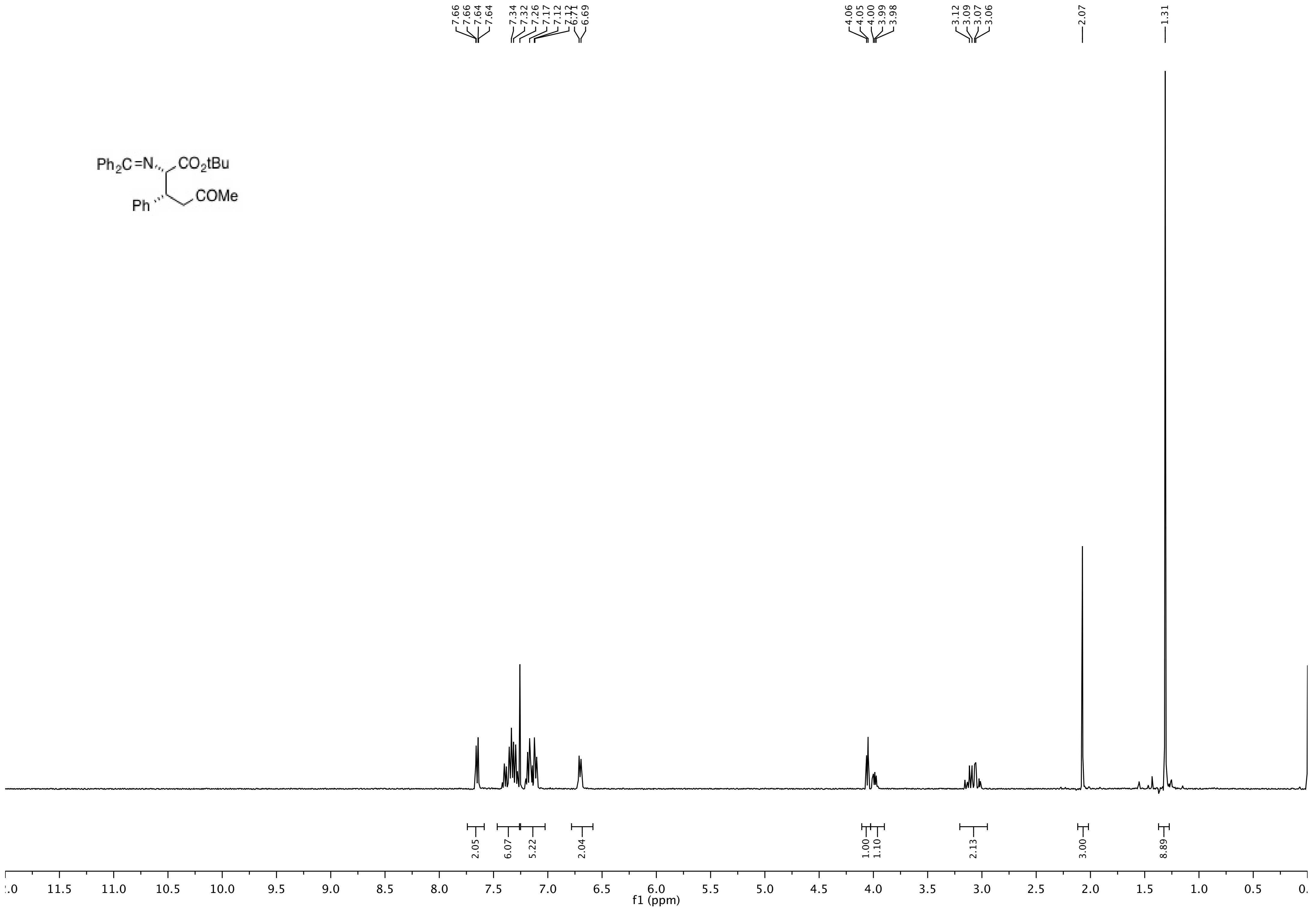

7.66
7.66
7.64
7.64
7.34
7.32
7.26
7.17
7.12
7.12
7.11
6.69
4.06
4.05
4.00
3.99
3.98
3.12
3.09
3.07
3.06
2.07
1.31
Ph2C=N CO2tBu
Ph COMe
2.05
6.07
5.22
2.04
1.00
1.10
2.13
3.00
8.89
11.5
11.0
10.5
10.0
9.5
9.0
8.5
8.0
7.5
7.0
6.5
6.0
5.5
5.0
4.5
4.0
3.5
3.0
2.5
2.0
1.5
1.0
0.5
f1 (ppm)

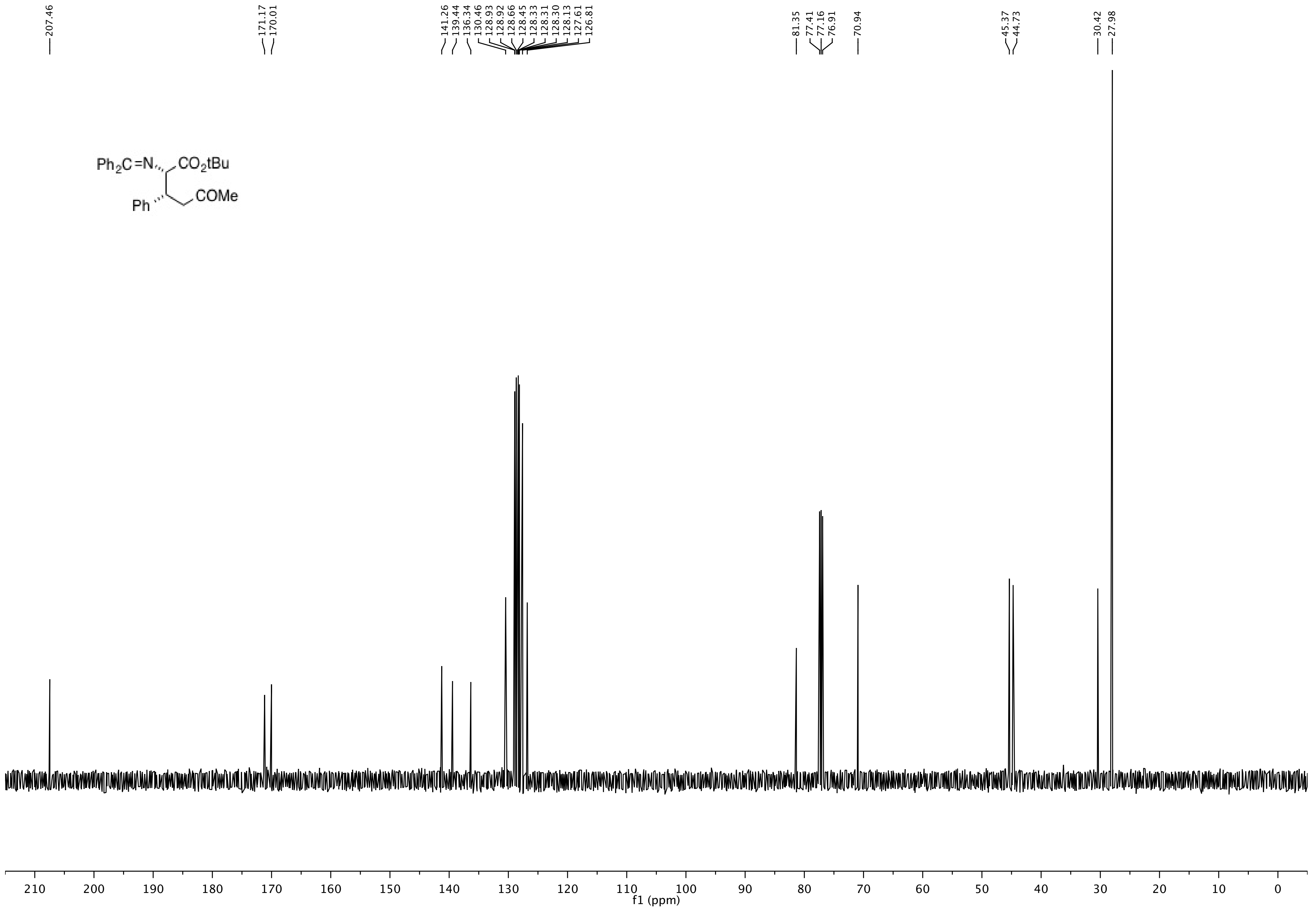
207.46
171.17
170.01
141.26
139.44
136.34
130.46
128.93
128.92
128.66
128.45
128.33
128.31
128.30
128.13
127.61
126.81
81.35
77.41
77.16
76.91
70.94
45.37
44.73
30.42
27.98
Ph2C=N
CO2tBu
Ph
COMe
210
200
190
180
170
160
150
140
130
120
110
100
90
80
70
60
50
40
30
20
10
0
f1 (ppm)

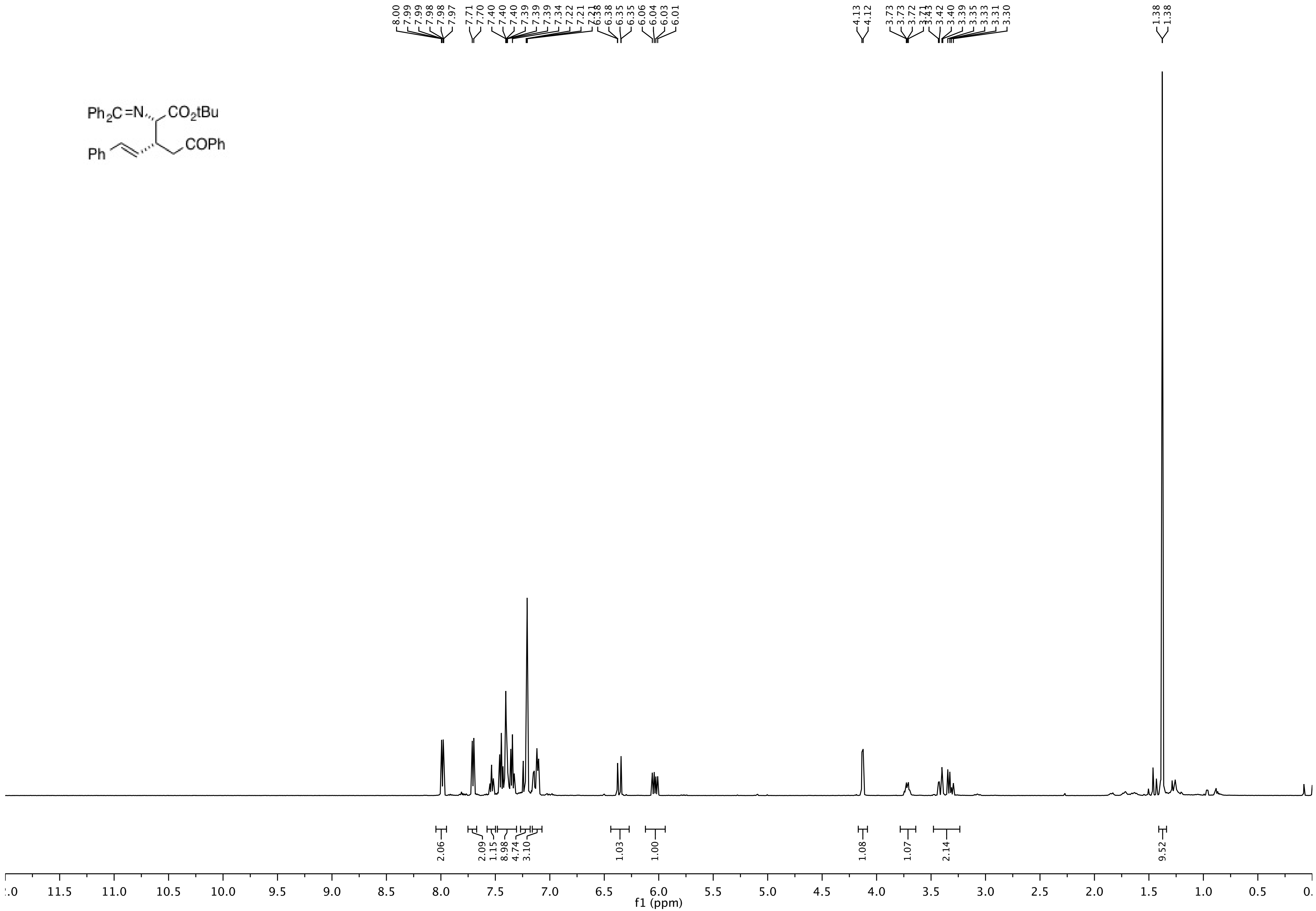

8.00 7.99 7.99 7.98 7.98 7.97 7.71 7.70 7.40 7.40 7.40 7.39 7.39 7.39 7.34 7.22 7.21 7.21 6.38 6.38 6.35 6.35 6.06 6.04 6.03 6.01
4.13 4.12 3.73 3.73 3.72 3.71 3.43 3.42 3.40 3.39 3.35 3.33 3.31 3.30
1.38 1.38
Ph2C=N CO2tBu
Ph COPh
2.06 2.09 1.15 8.98 4.74 3.10 1.03 1.00 1.08 1.07 2.14 9.52
11.5 11.0 10.5 10.0 9.5 9.0 8.5 8.0 7.5 7.0 6.5 6.0 5.5 5.0 4.5 4.0 3.5 3.0 2.5 2.0 1.5 1.0 0.5
f1 (ppm)

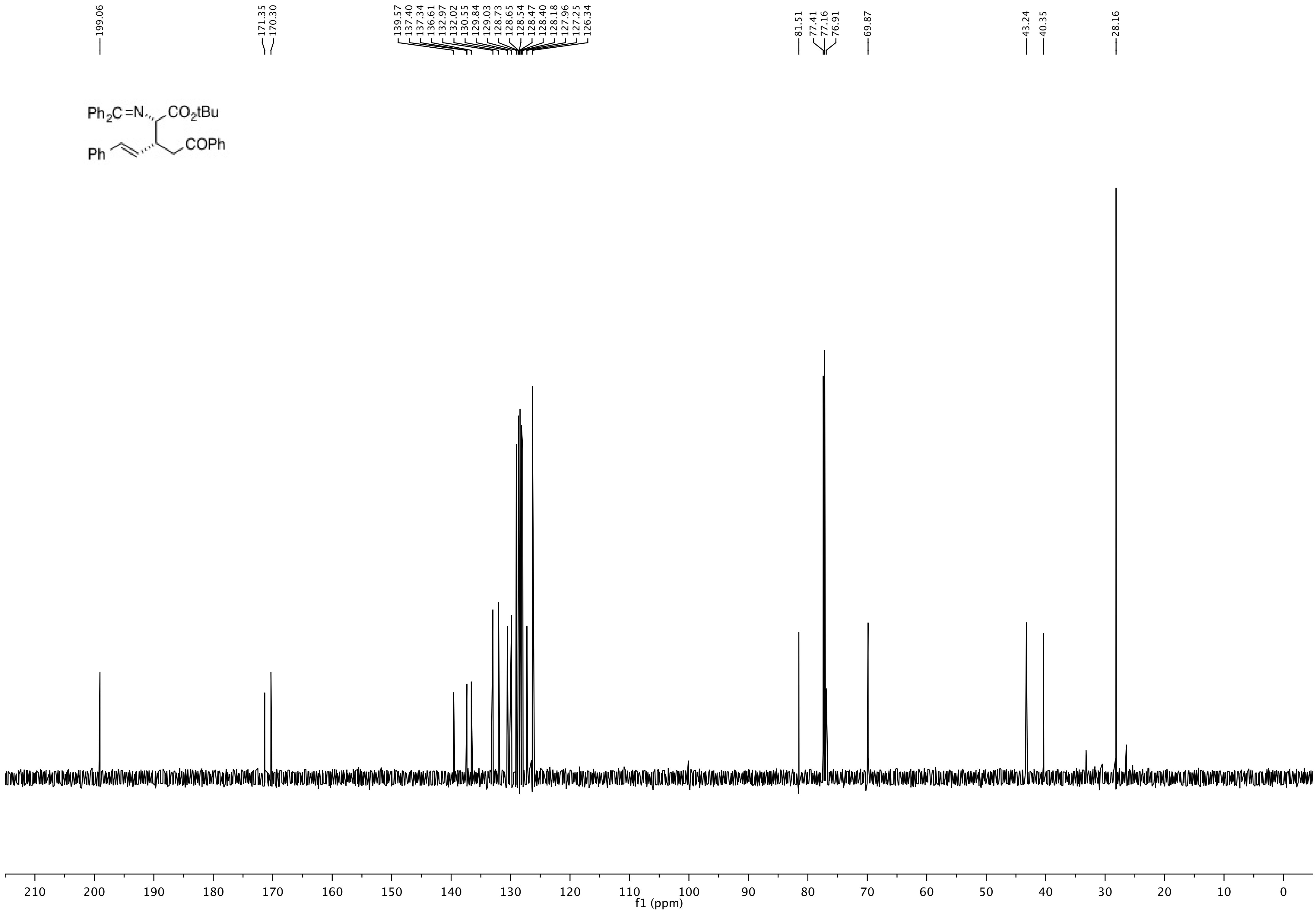
199.06
171.35
170.30
139.57
137.40
137.34
136.61
132.97
132.02
130.55
129.84
129.03
128.73
128.65
128.54
128.47
128.40
128.18
127.96
127.25
126.34
81.51
77.41
77.16
76.91
69.87
43.24
40.35
28.16
Ph2C=N CO2tBu
Ph COPh
210 200 190 180 170 160 150 140 130 120 110 100 90 80 70 60 50 40 30 20 10 0
f1 (ppm)

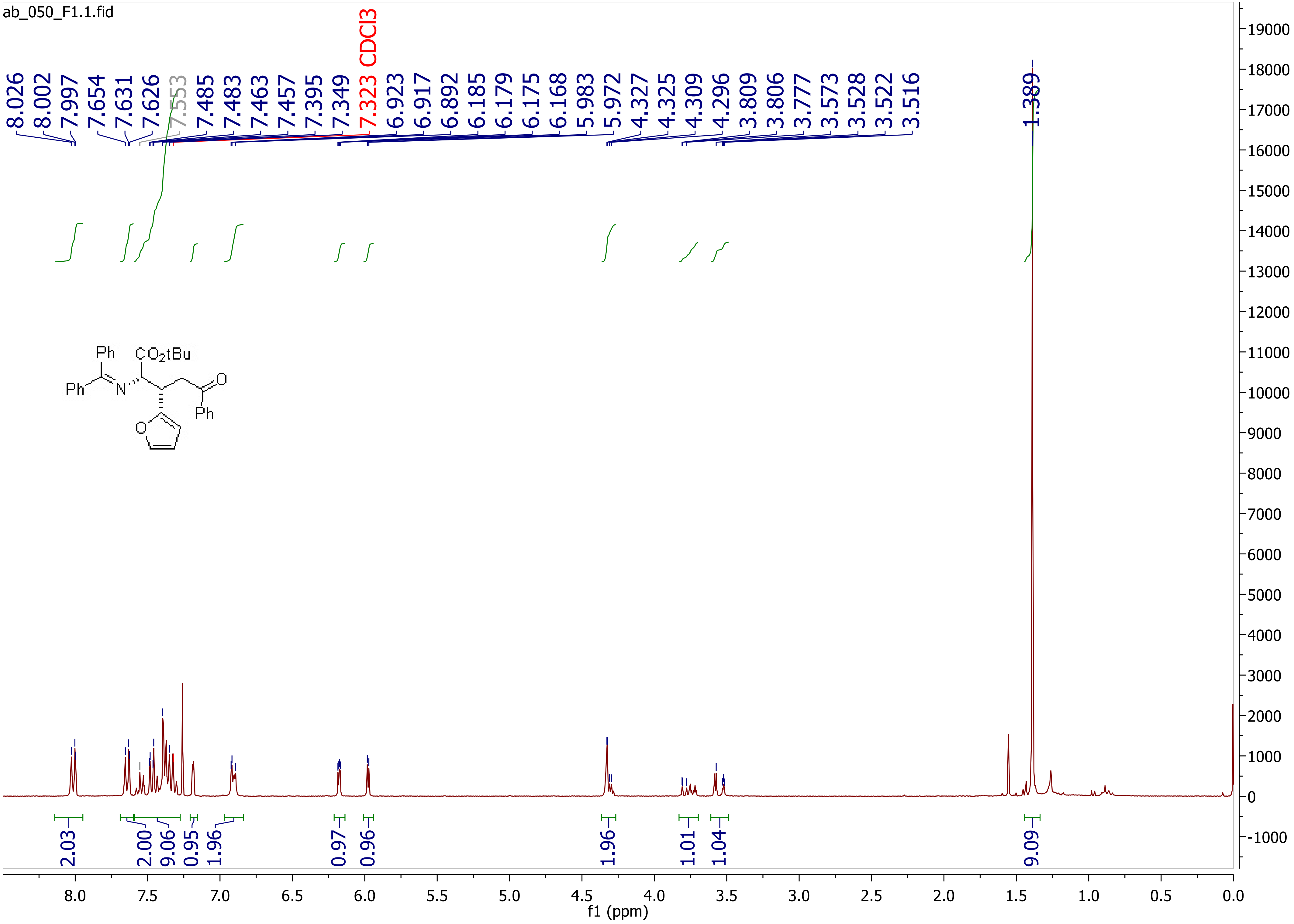

ab_050_F1.1.fid
CDCl3
8.026
8.002
7.997
7.654
7.631
7.626
7.553
7.485
7.483
7.463
7.457
7.395
7.349
7.323
6.923
6.917
6.892
6.185
6.179
6.175
6.168
5.983
5.972
4.327
4.325
4.309
4.296
3.809
3.806
3.777
3.573
3.528
3.522
3.516
1.389
Ph
Ph
N
CO2tBu
O
Ph
O
2.03
2.00
9.06
0.95
1.96
0.97
0.96
1.96
1.01
1.04
9.09
f1 (ppm)

ab_050_F1checkC.1.fid
proton

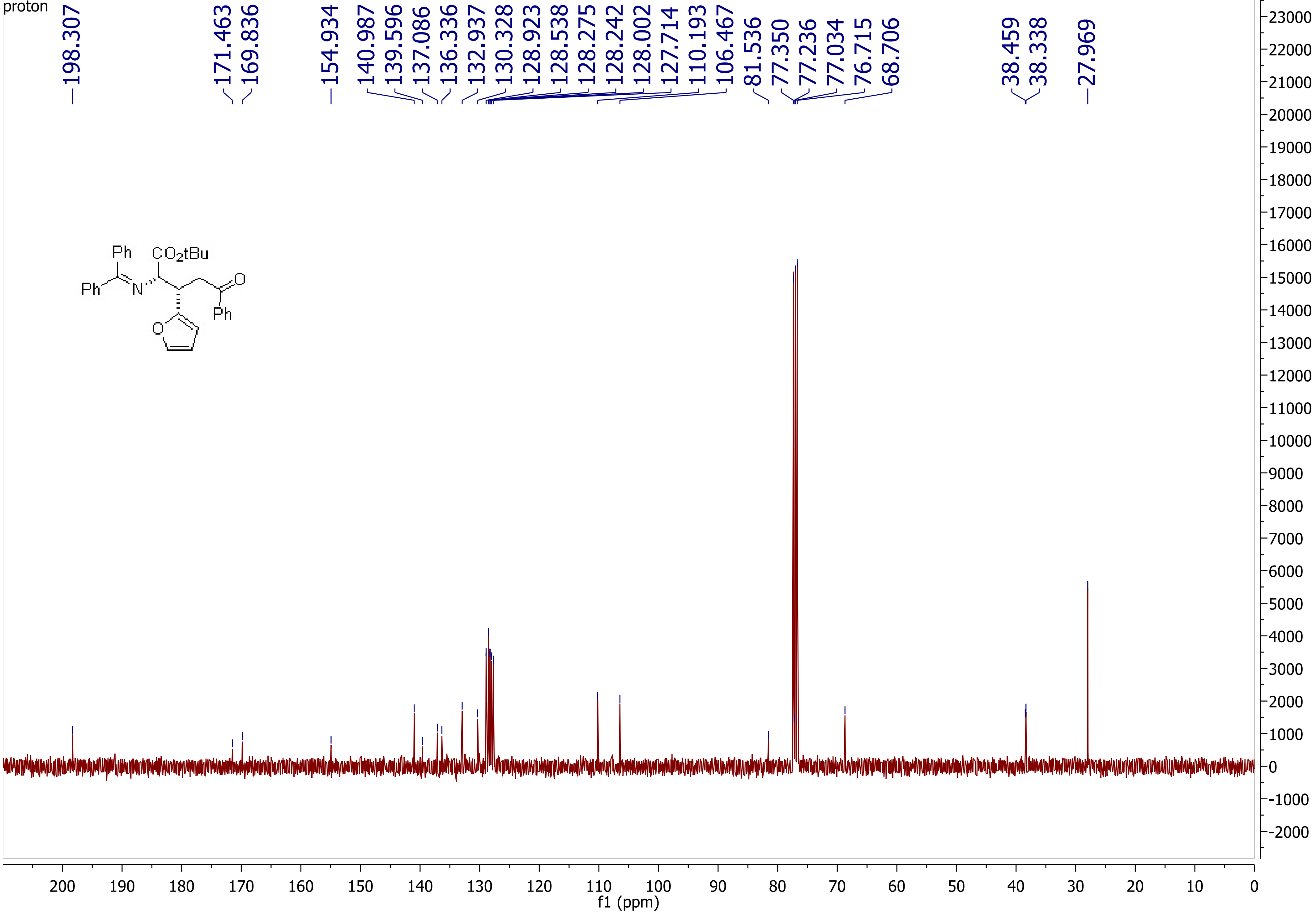

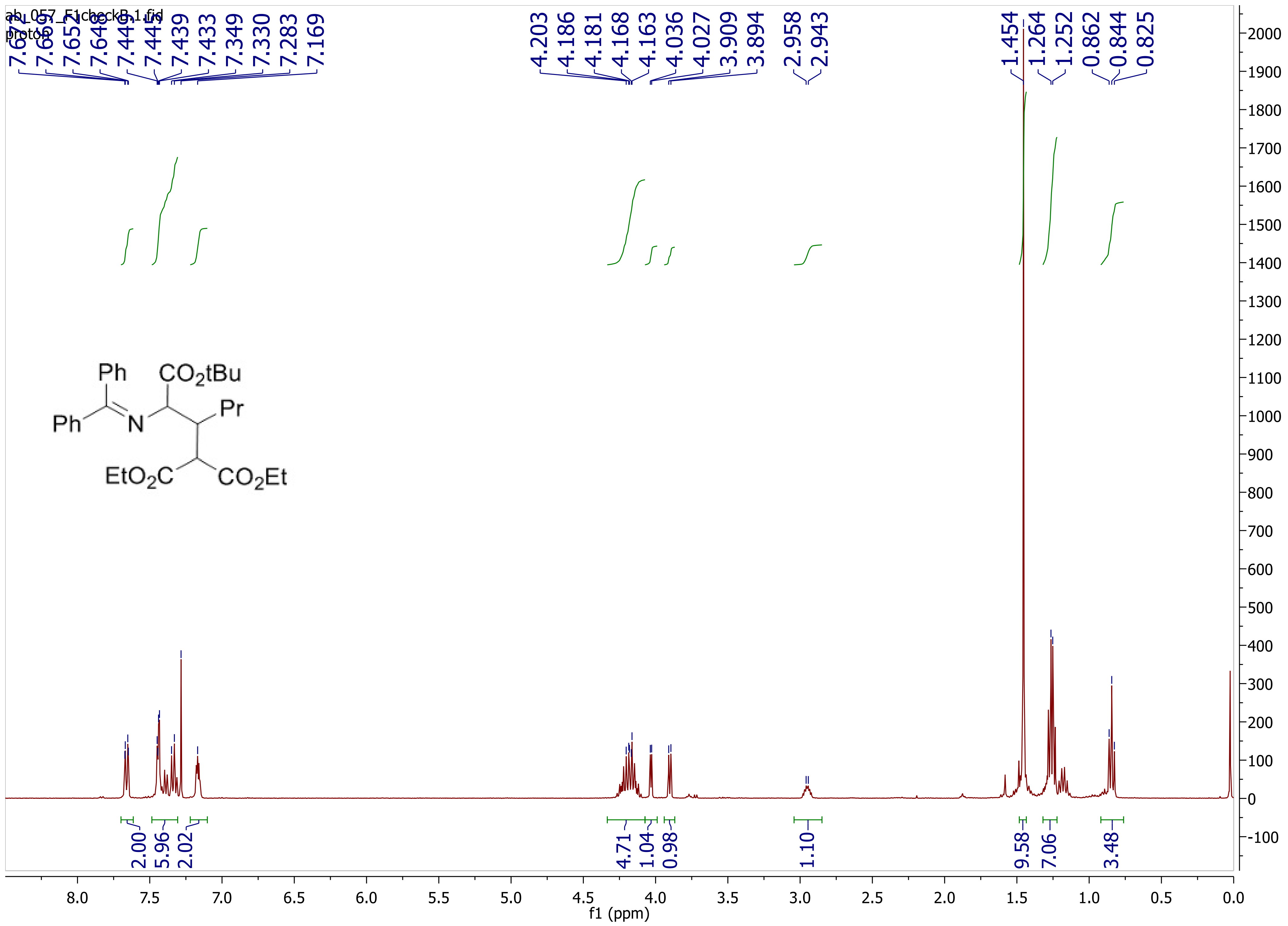

ab_057_F1checkB.1.fid
proton
7.652
7.648
7.445
7.439
7.433
7.349
7.330
7.283
7.169
4.203
4.186
4.181
4.168
4.163
4.036
4.027
3.909
3.894
2.958
2.943
1.454
1.264
1.252
0.862
0.844
0.825
Ph
CO2tBu
Pr
Ph
N
EtO2C
CO2Et
2.00
5.96
2.02
4.71
1.04
0.98
1.10
9.58
7.06
3.48
f1 (ppm)

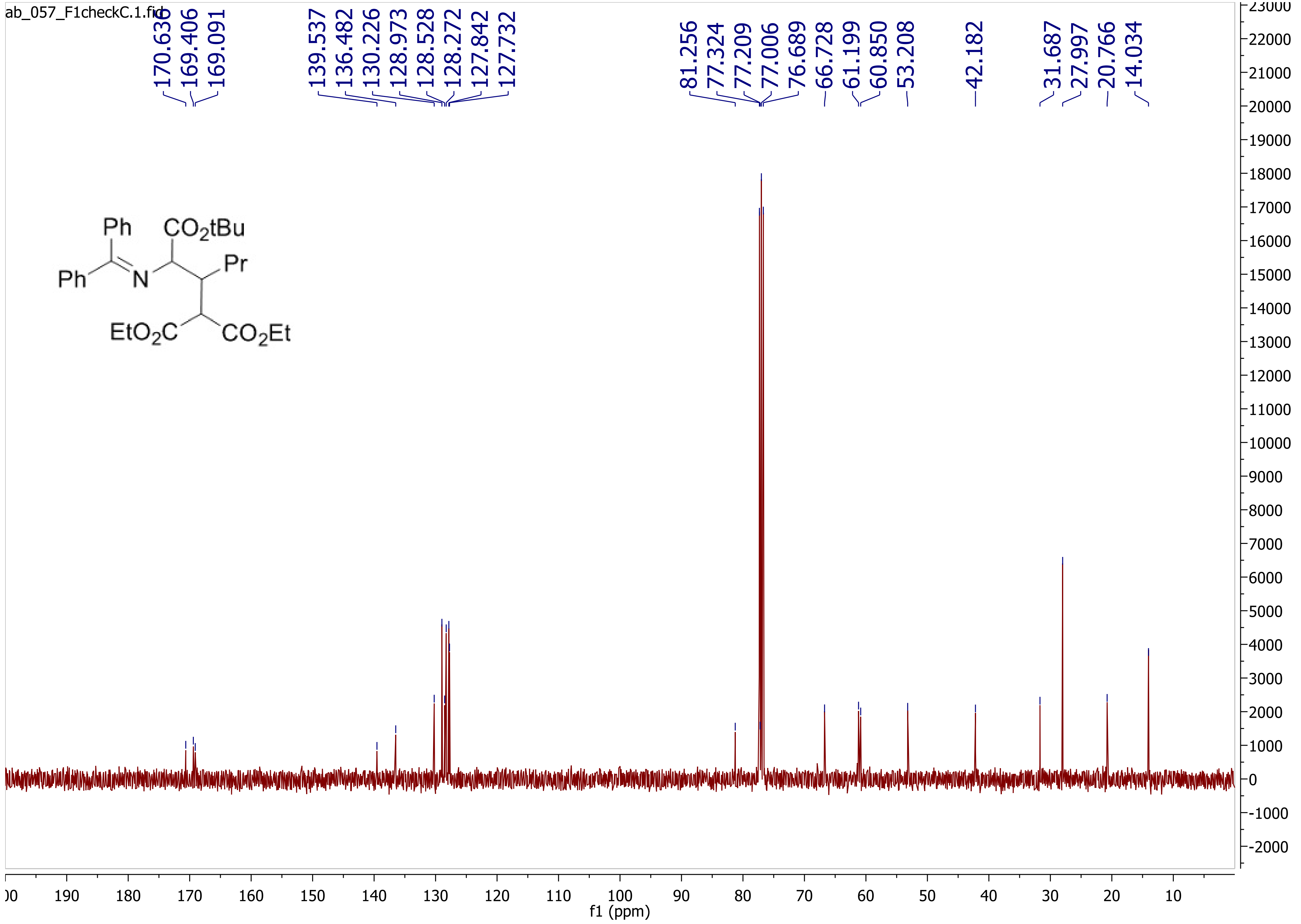
ab_057_F1checkC.1.fid
170.636
169.406
169.091
139.537
136.482
130.226
128.973
128.528
128.272
127.842
127.732
81.256
77.324
77.209
77.006
76.689
66.728
61.199
60.850
53.208
42.182
31.687
27.997
20.766
14.034
Ph
CO2tBu
Pr
Ph
N
EtO2C
CO2Et
f1 (ppm)

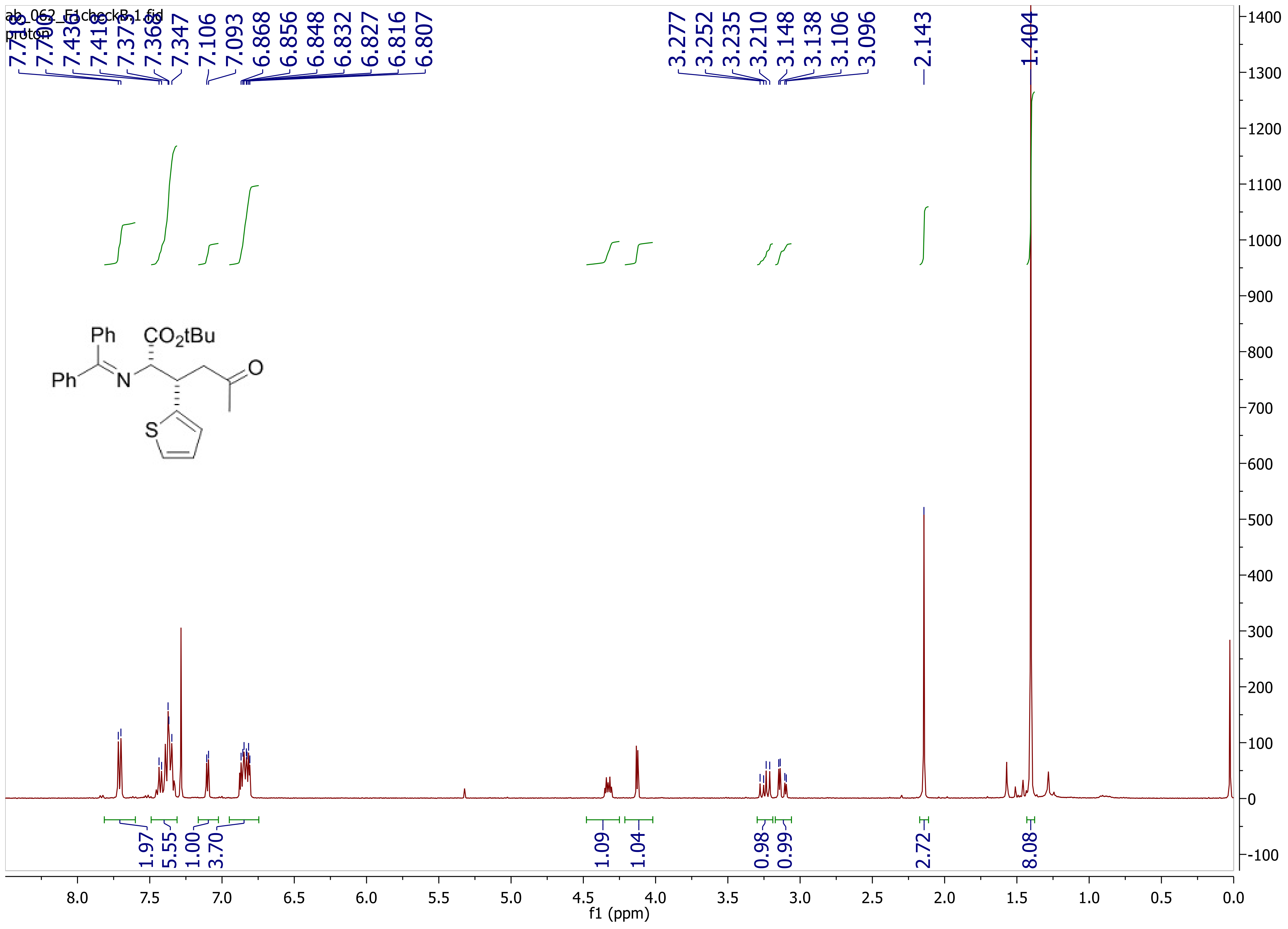

ab_062_F1checkB.1.fid
proton
7.718
7.700
7.436
7.418
7.373
7.368
7.347
7.106
7.093
6.868
6.856
6.848
6.832
6.827
6.816
6.807
3.277
3.252
3.235
3.210
3.148
3.138
3.106
3.096
2.143
1.404
Ph
CO2tBu
Ph
N
O
S
1.97
5.55
1.00
3.70
1.09
1.04
0.98
0.99
2.72
8.08
f1 (ppm)

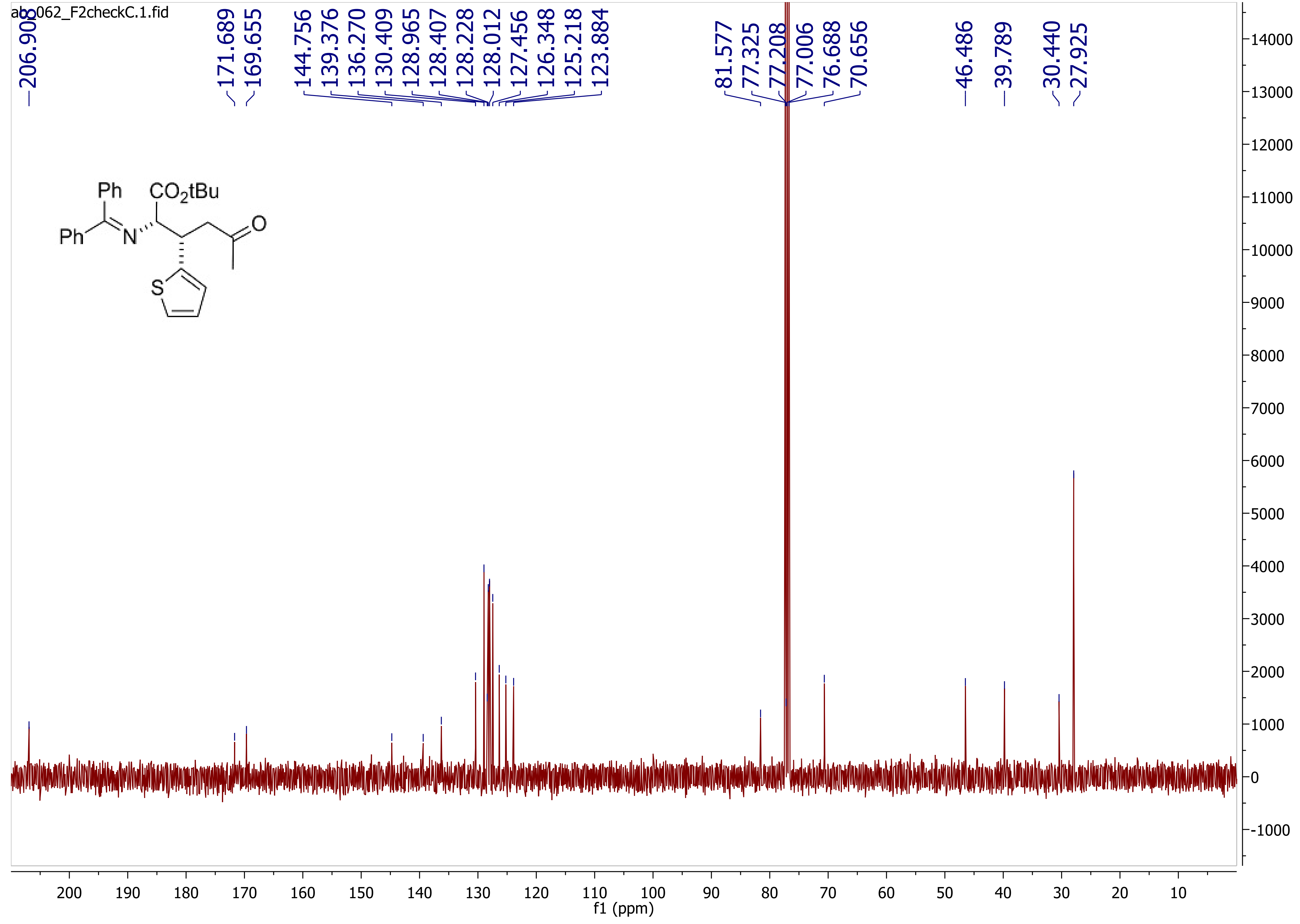
ab_062_F2checkC.1.fid
206.908
171.689
169.655
144.756
139.376
136.270
130.409
128.965
128.407
128.228
128.012
127.456
126.348
125.218
123.884
81.577
77.325
77.208
77.006
76.688
70.656
46.486
39.789
30.440
27.925
Ph
Ph
N
CO2tBu
O
S
f1 (ppm)

Note: For each entry, the top HPLC trace is a racemic sample that was prepared using standard conditions and a cyclopropenimine catalyst lacking a chiral substituent.

Equation 5, **19**. Hexanes/iPrOH 97/3, 1 mL/min, 254 nm, OD-H.

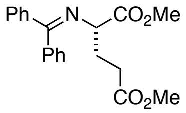

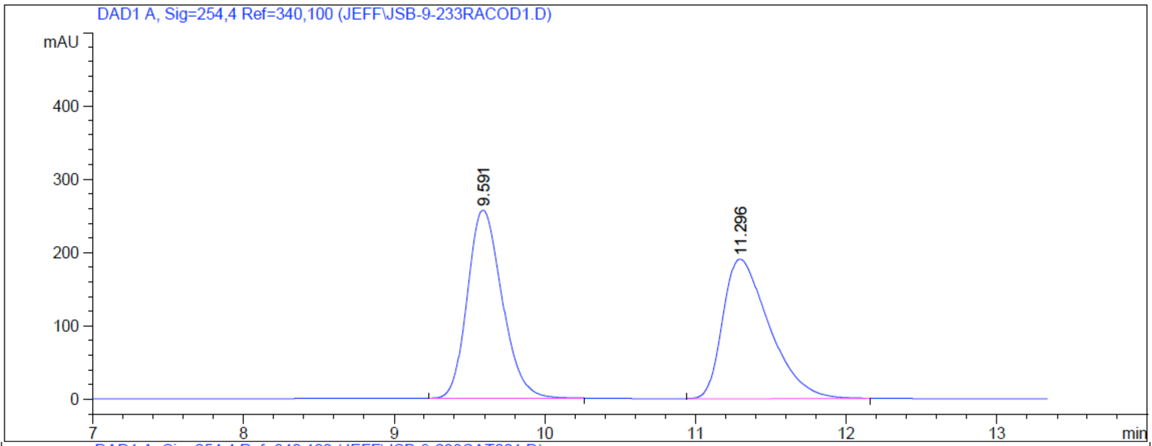

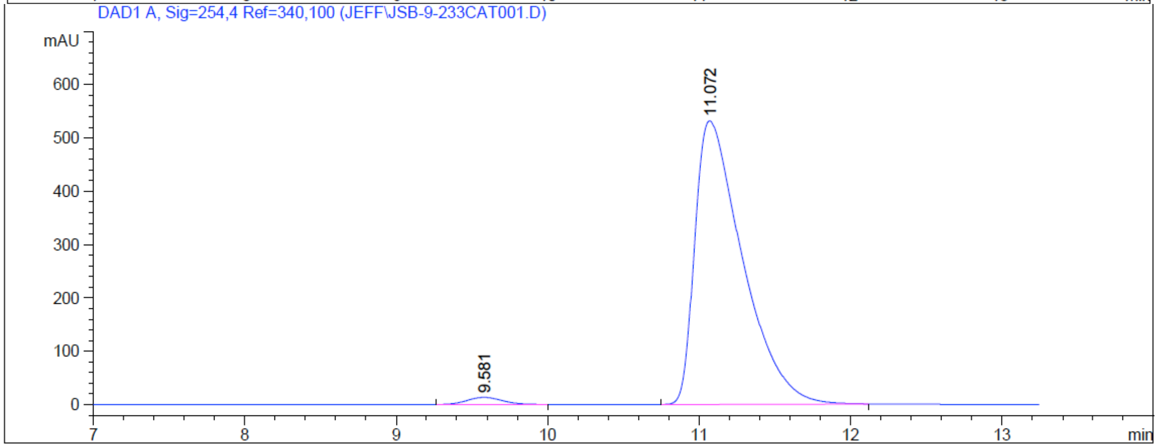

Peak #	RetTime [min]	Sig	Type	Area [mAU*s]	Height [mAU]	Area %
1	9.581	1	BB	220.26395	13.59522	1.8606
2	11.072	1	BB	1.16180e4	531.61310	98.1394

Equation 5, **20**. Hexanes/iPrOH 97/3, 1 mL/min, 254 nm, OD-H.

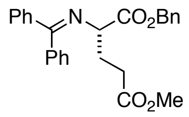

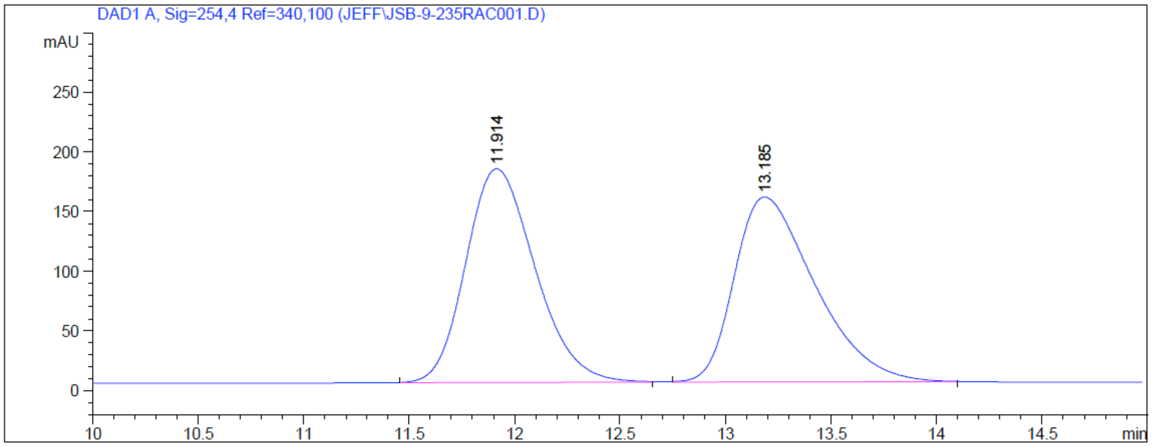

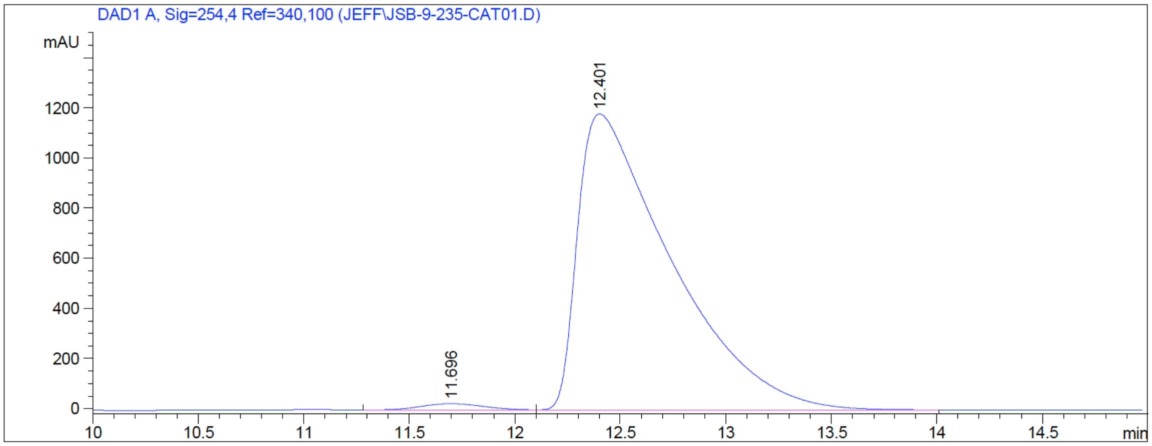

Peak #	RetTime [min]	Sig	Type	Area [mAU*s]	Height [mAU]	Area %
1	11.696	1	VV	541.52197	25.60269	1.4679
2	12.401	1	VB	3.63491e4	1180.74292	98.5321

Equation 5, **22**. Hexanes/iPrOH 98/2, 0.8 mL/min, 254 nm, OD-H.

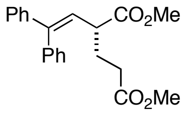

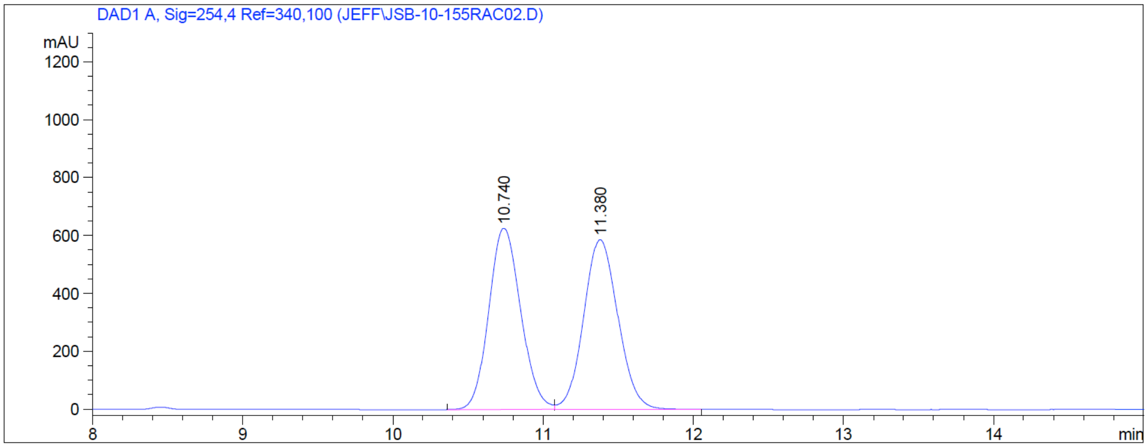

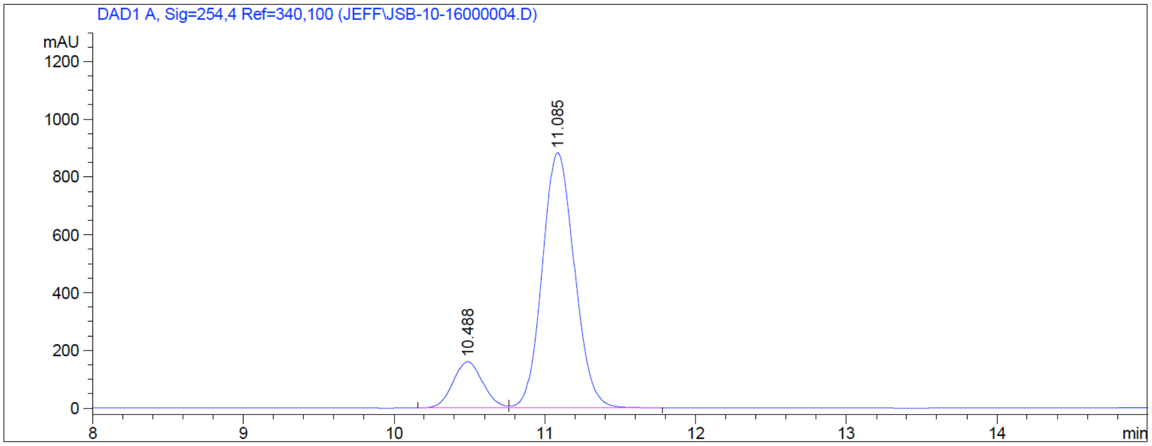

Peak #	RetTime [min]	Sig	Type	Area [mAU*s]	Height [mAU]	Area %
1	10.488	1	BV	2213.77783	160.14494	14.4041
2	11.085	1	VB	1.31553e4	882.94867	85.5959

Equation 5, **21**. Hexanes/iPrOH 98.5/1.5, 0.7 mL/min, 254 nm, OD-H.

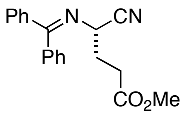

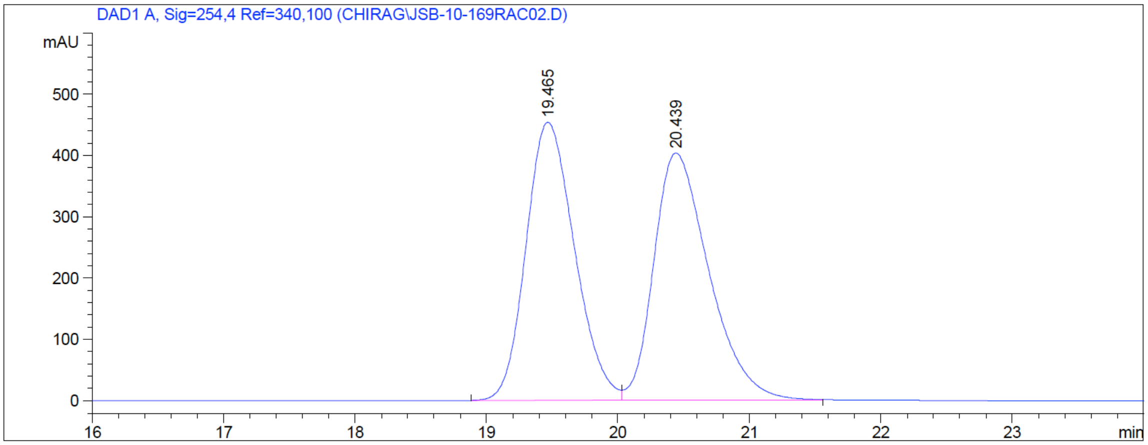

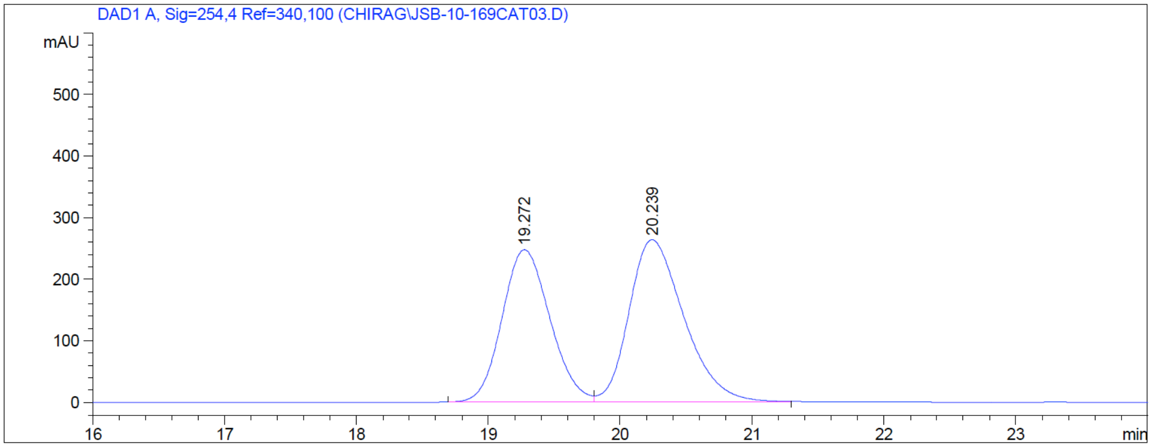

Peak #	RetTime [min]	Sig	Type	Area [mAU*s]	Height [mAU]	Area %
1	19.272	1	BV	6234.42676	246.89293	45.0987
2	20.239	1	VB	7589.53662	262.84201	54.9013

Table 2, entry 1. Hexanes/iPrOH 97/3, 1 mL/min, 254 nm, AD-H.

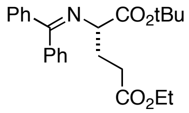

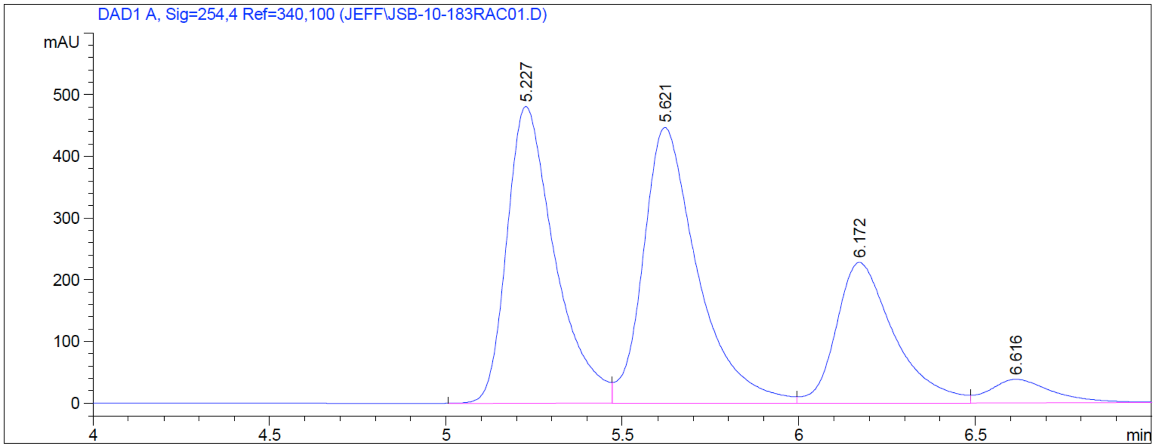

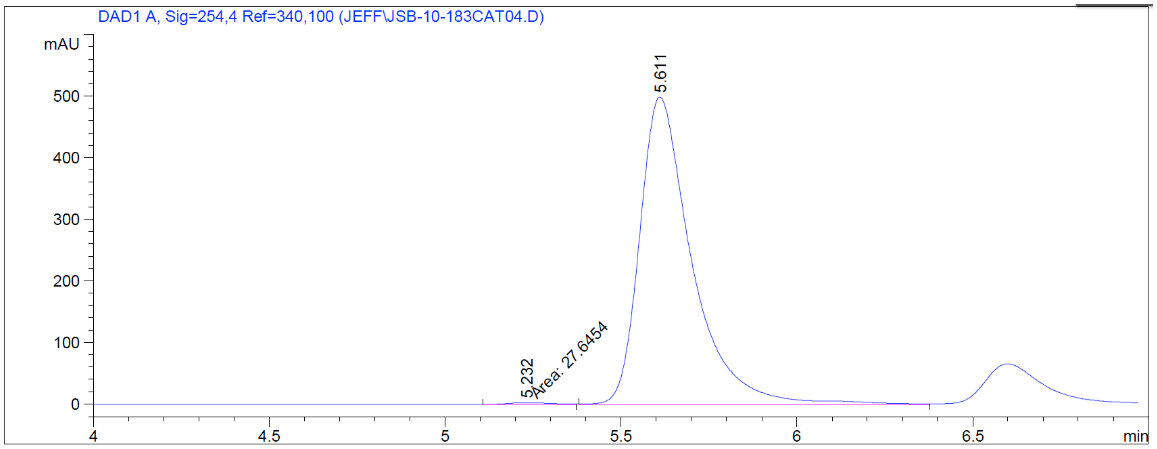

Peak #	RetTime [min]	Sig	Type	Area [mAU*s]	Height [mAU]	Area %
1	5.232	1	MM T	27.64539	3.09858	0.5306
2	5.611	1	VV	5183.00488	499.06174	99.4694

Table 2, entry 3. Hexanes/iPrOH 98.5/1.5, 1 mL/min, 254 nm, OD-H.

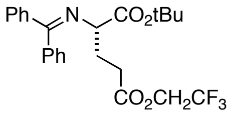

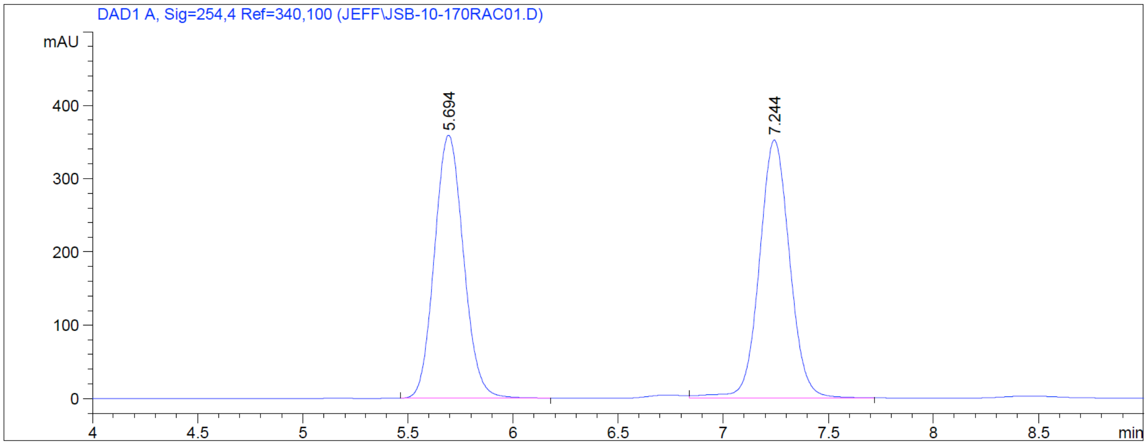

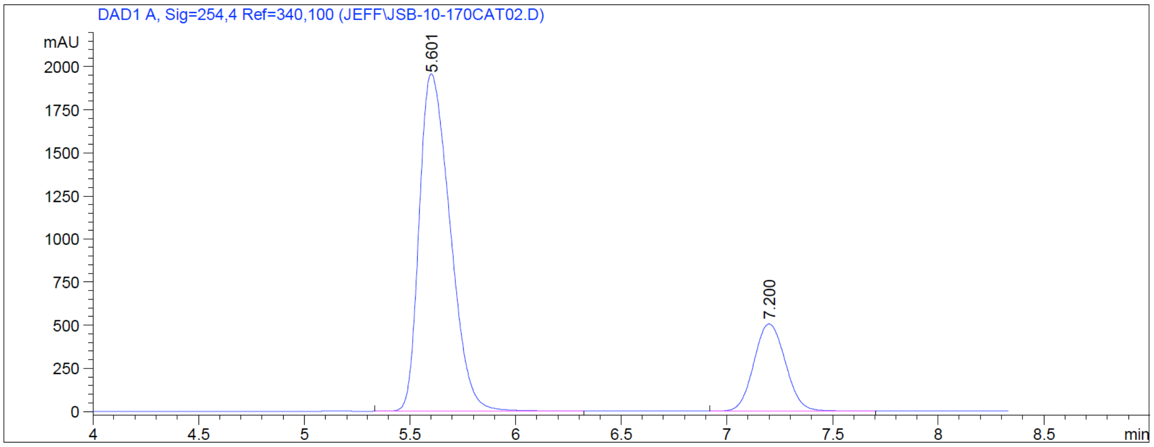

Peak #	RetTime [min]	Sig	Type	Area [mAU*s]	Height [mAU]	Area %
1	5.601	1	BB	1.98404e4	1958.64746	79.0668
2	7.200	1	BB	5252.81689	506.48874	20.9332

Table 3, entry 1. Hexanes/EtOH 98/2, 1 mL/min, 254 nm, AD-H.

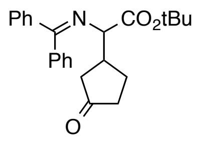

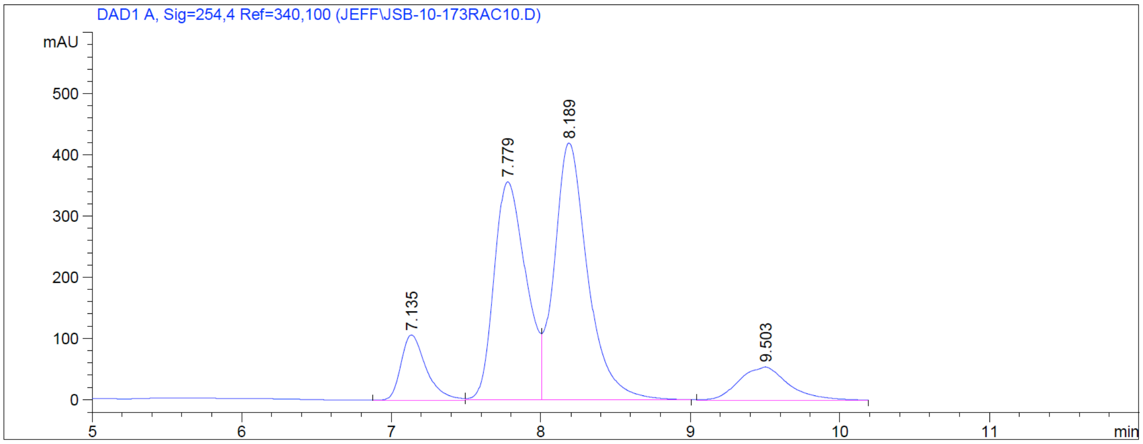

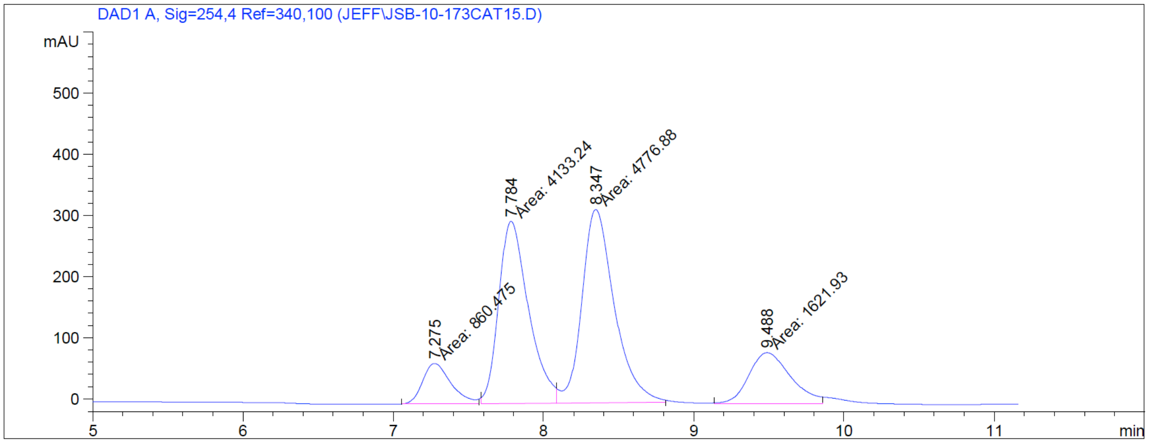

Peak #	RetTime [min]	Sig	Type	Area [mAU*s]	Height [mAU]	Area %
1	7.275	1	MM T	860.47479	65.83994	7.5530
2	7.784	1	MF T	4133.23828	297.99585	36.2803
3	8.347	1	FM T	4776.87842	315.63742	41.9299
4	9.488	1	MM T	1621.93311	83.56573	14.2368

Table 3, entry 2. Hexanes/iPrOH 96/4, 1 mL/min, 254 nm, AD-H.

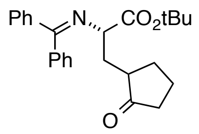

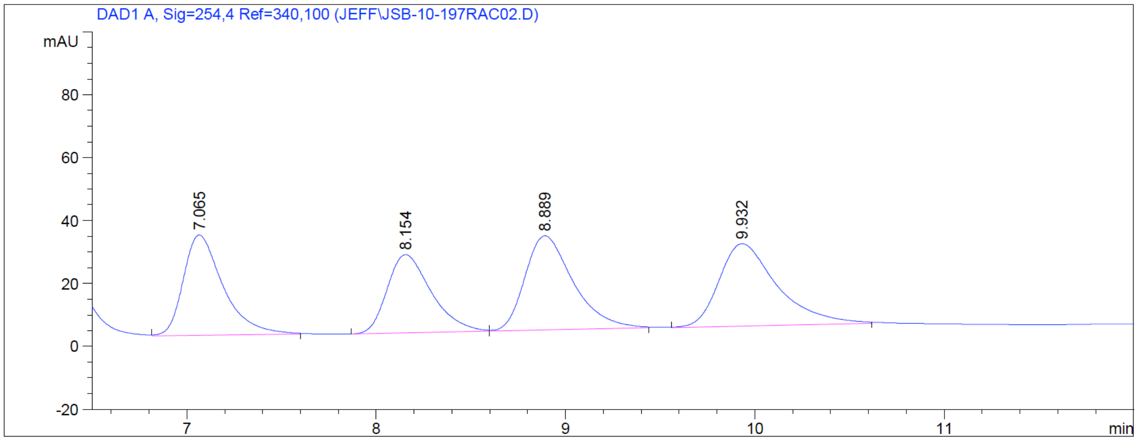

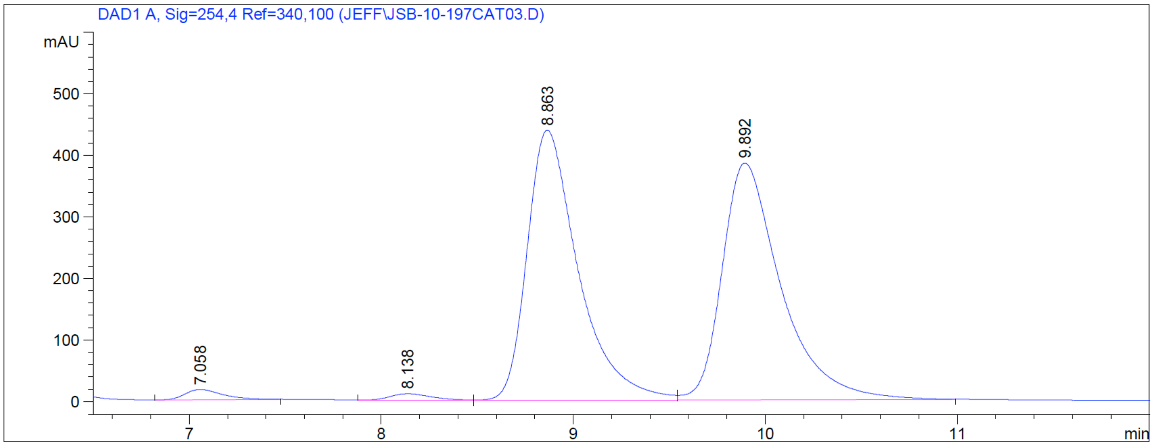

Peak #	RetTime [min]	Sig	Type	Area [mAU*s]	Height [mAU]	Area %
1	7.058	1	BB	234.12263	16.82588	1.4056
2	8.138	1	BV	145.43689	10.15079	0.8731
3	8.863	1	VV	8092.19971	438.02695	48.5815
4	9.892	1	VB	8185.20020	384.25171	49.1398

Table 6, entry 1: Hexanes/iPrOH 98/2, 1 mL/min, 254 nm, AD-H

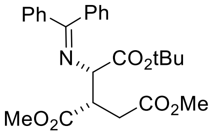

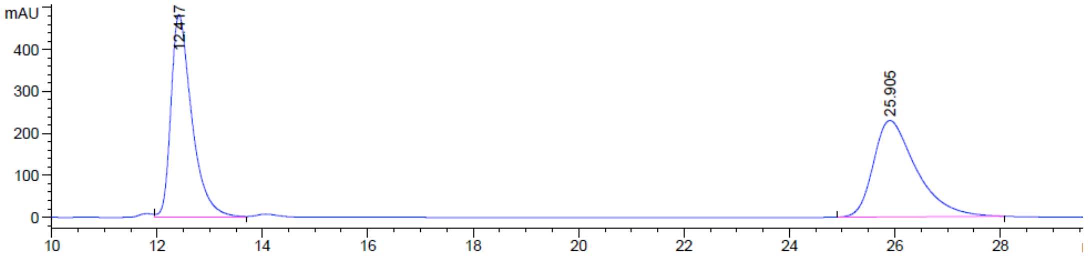

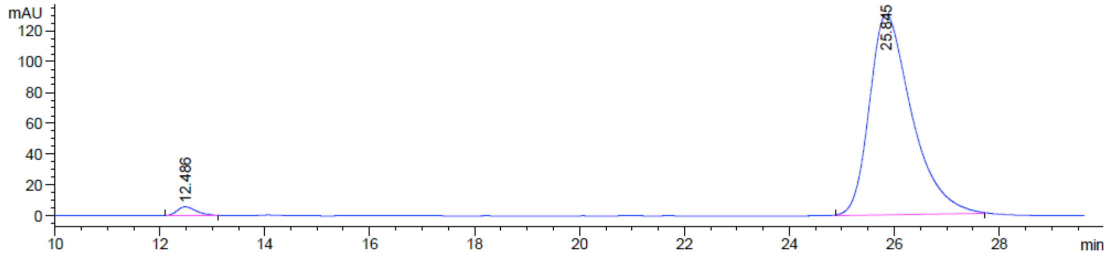

Peak #	RetTime [min]	Type	Width [min]	Area [mAU*s]	Height [mAU]	Area %
1	12.486	BB	0.3758	144.56180	5.64371	1.9442
2	25.845	BB	0.8407	7291.11523	130.12016	98.0558

Table 6, entry 3: Hexanes/iPrOH 95/5, 1 mL/min, 254 nm, AD-H

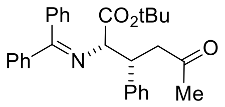

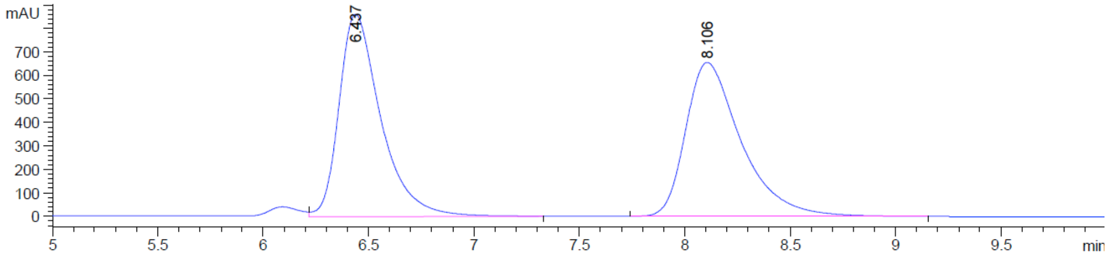

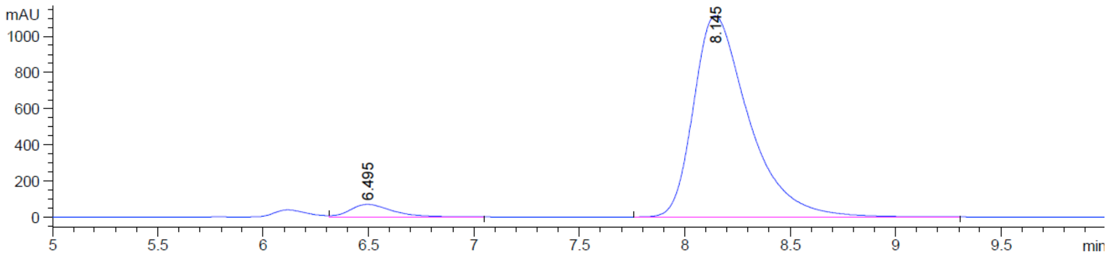

Peak #	RetTime [min]	Type	Width [min]	Area [mAU*s]	Height [mAU]	Area %
1	6.495	VB	0.2097	973.36182	69.81744	4.5746
2	8.145	BB	0.2747	2.03041e4	1113.64990	95.4254

Table 6 entry 4: Hexanes/iPrOH 95/5, 1 mL/min, 254 nm, AD-H

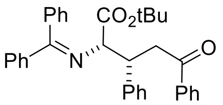

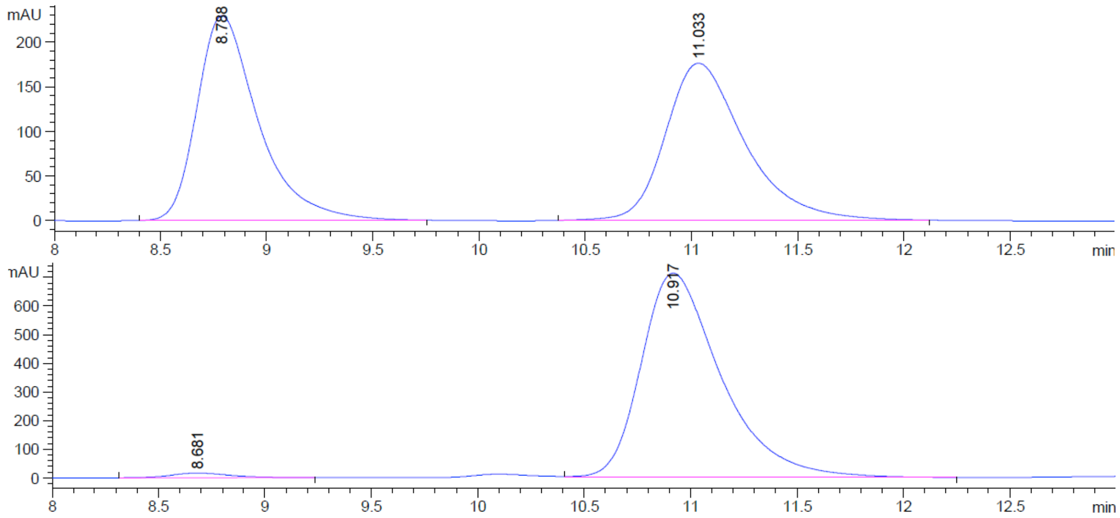

Peak #	RetTime [min]	Type	Width [min]	Area [mAU*s]	Height [mAU]	Area %
1	8.681	BB	0.3025	319.17154	15.94729	1.6749
2	10.917	VB	0.3952	1.87366e4	713.91718	98.3251

Table 6, entry 5: Hexanes/iPrOH 95/5, 1 mL/min, 254 nm, AD-H

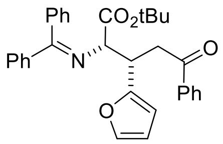

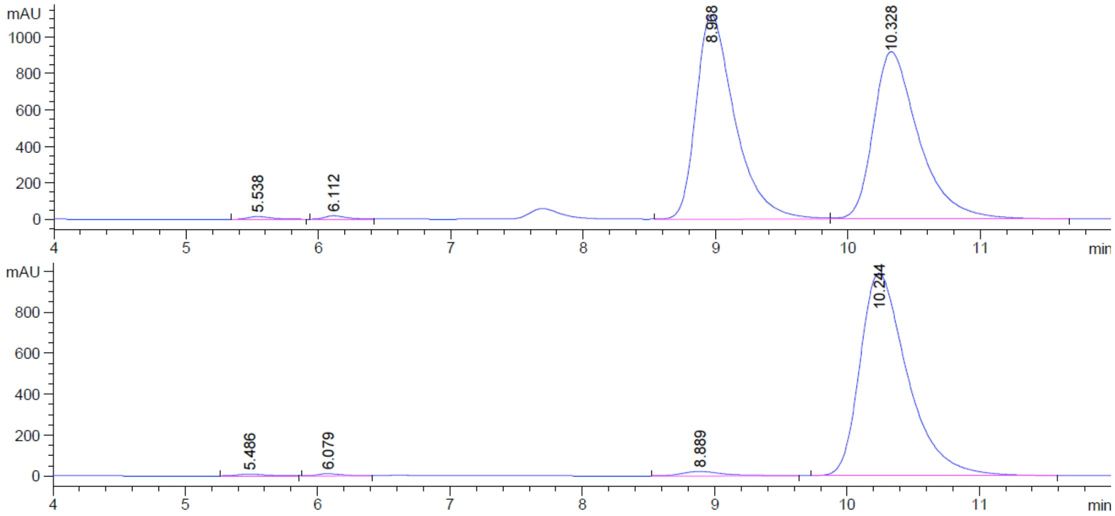

Peak #	RetTime [min]	Type	Width [min]	Area [mAU*s]	Height [mAU]	Area %
1	3.109	BB	0.1299	115.94562	13.02428	0.4532
2	5.486	BB	0.2140	122.80331	8.84298	0.4800
3	6.079	BB	0.1869	128.54597	10.49434	0.5024
4	8.889	BB	0.3304	479.36398	21.81064	1.8736
5	10.244	BB	0.3777	2.47386e4	989.43939	96.6908

Table 6, entry 6: Hexanes/iPrOH 95/5, 1 mL/min, 254 nm, AD-H

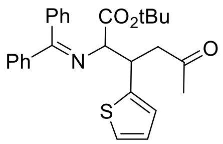

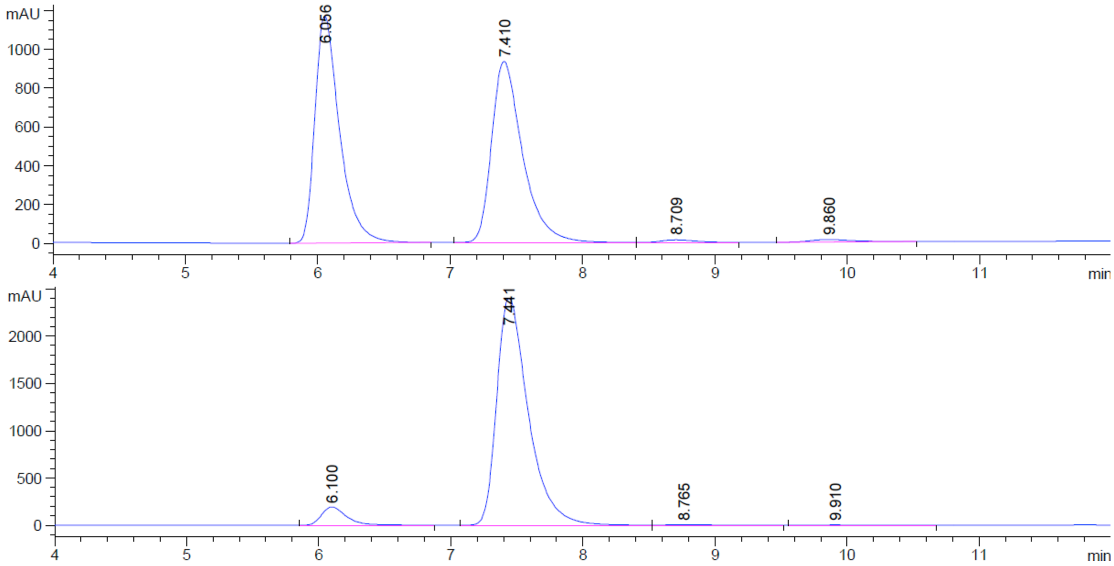

Peak #	RetTime [min]	Type	Width [min]	Area [mAU*s]	Height [mAU]	Area %
1	6.100	BB	0.2017	2606.30786	195.26425	6.0833
2	7.441	BB	0.2503	3.99973e4	2401.12329	93.3561
3	8.765	BB	0.3759	157.62022	6.13147	0.3679
4	9.910	BB	0.3574	82.58051	3.42343	0.1927

Table 6, entry 7. Hexanes/EtOH 97/3, 1 mL/min, 254 nm, OD-H (using catalyst **1**)

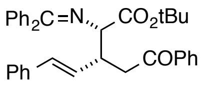

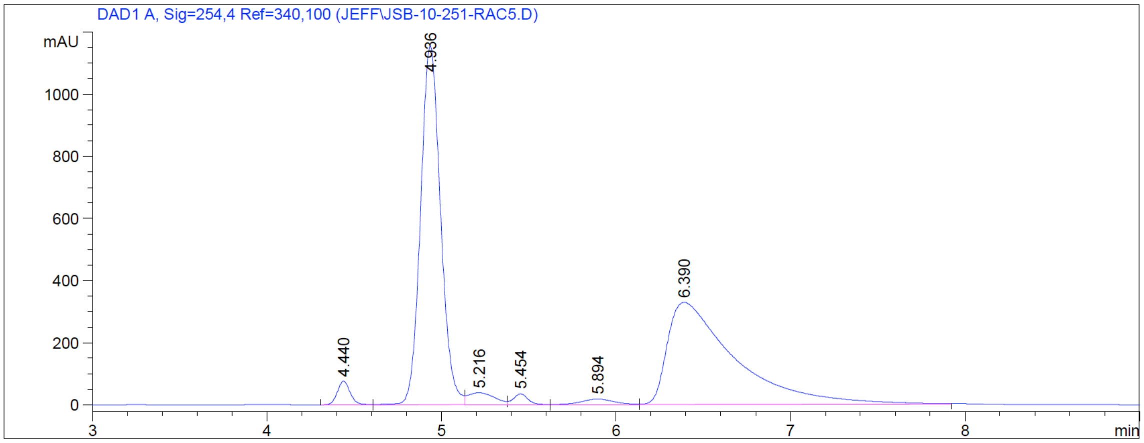

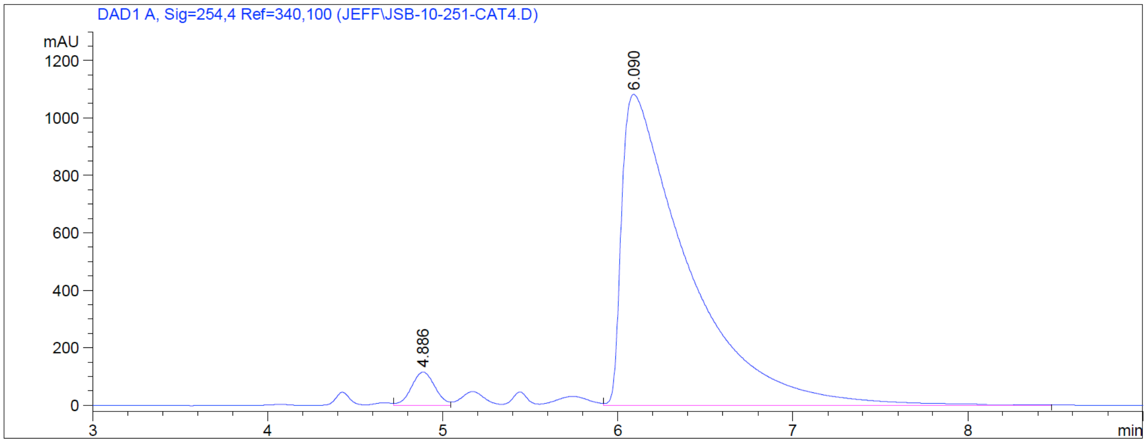

Peak #	RetTime [min]	Sig	Type	Area [mAU*s]	Height [mAU]	Area %
1	4.886	1	VV	1093.96570	116.45546	3.7026
2	6.090	1	VB	2.84522e4	1083.16150	96.2974

Table 6, entry 7. Hexanes/EtOH 97/3, 1 mL/min, 254 nm, OD-H (using catalyst **1**)

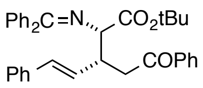

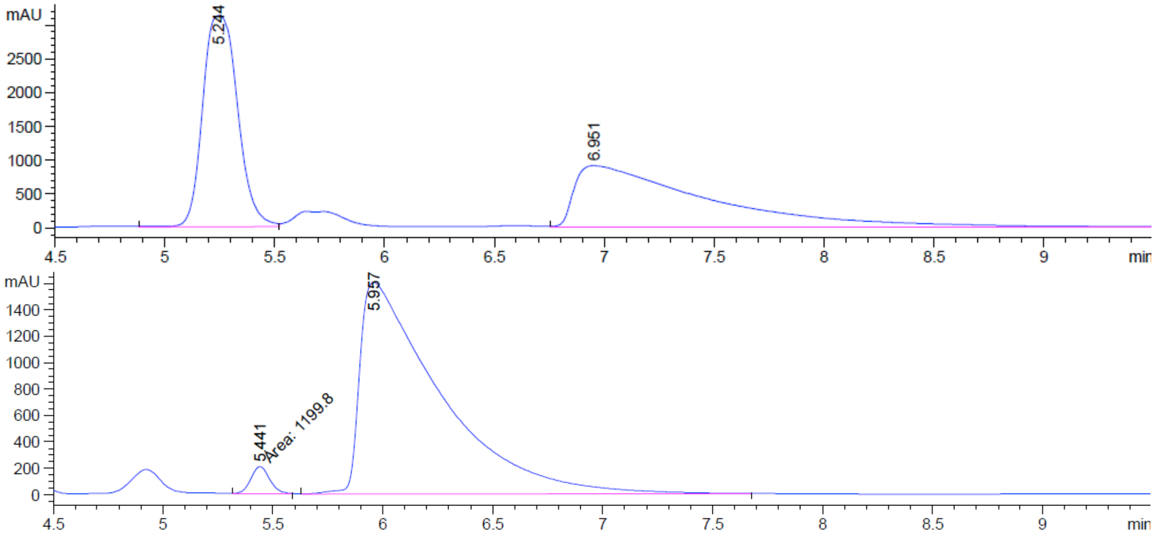

Peak #	RetTime [min]	Type	Width [min]	Area [mAU*s]	Height [mAU]	Area %
1	5.441	MM T	0.0978	1199.80334	204.56404	2.8510
2	5.957	VB	0.3492	4.08831e4	1604.62085	97.1490

Table 6 entry 8: Hexanes/iPrOH 98/2, 1 mL/min, 254 nm, AD-H

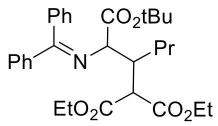

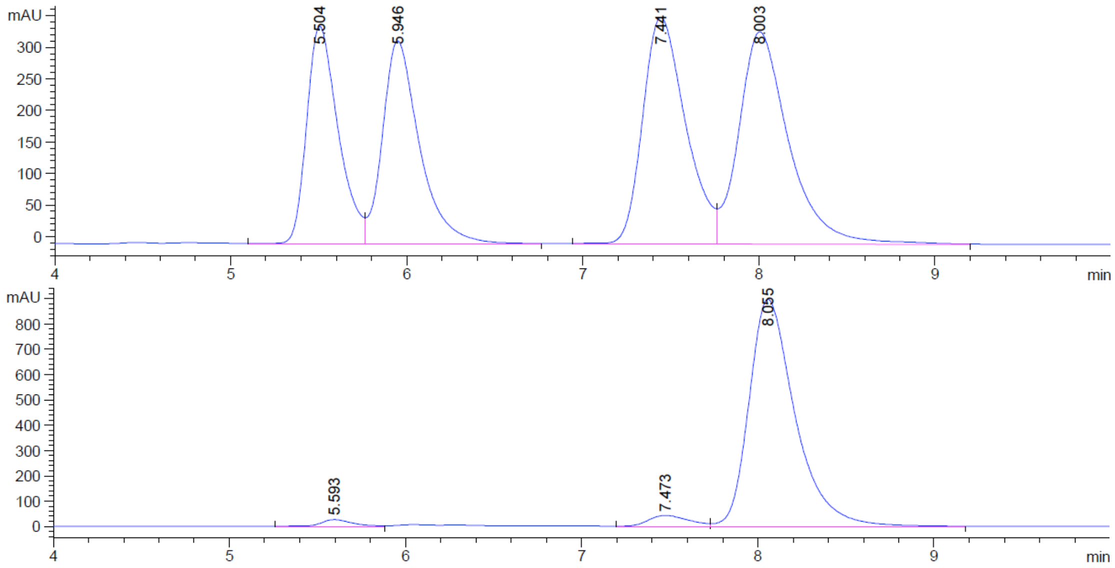

Peak #	RetTime [min]	Type	Width [min]	Area [mAU*s]	Height [mAU]	Area %
1	5.593	BV	0.1824	327.70663	26.84514	1.8945
2	7.473	BV	0.2449	671.32690	42.35588	3.8810
3	8.055	VB	0.2740	1.62990e4	896.84387	94.2246
